# Lung endothelial PEAR1 induces tumor cell dormancy

**DOI:** 10.1186/s12943-025-02488-3

**Published:** 2025-11-03

**Authors:** Kenneth Anthony Roquid, Adriana Vucetic, Elena Dyukova, Mika J. Hanssen, Haaglim Cho, Rémy Bonnavion, Kenny Mattonet, Mario Looso, Miloslav Sanda, Boris Strilic, Stefan Offermanns

**Affiliations:** 1 Department of Pharmacology, Max Planck Institute for Heart and Lung Research, Ludwigstr. 43, Bad Nauheim, 61231 Germany; 2 Max Planck Institute for Heart and Lung Research, Biomolecular Mass Spectrometry, Ludwigstr. 43, Bad Nauheim, 61231 Germany; 3 Center for Molecular Medicine, Goethe University Frankfurt, Theodor-Stern-Kai 7, Frankfurt, 60590 Germany; 4 Frankfurt Cancer Institute (FCI), Goethe University Frankfurt, Frankfurt, Germany; 5 Cardiopulmonary Institute (CPI), Bad Nauheim, Germany; 6 Max Planck Institute for Heart and Lung Research, Imaging Platform, Ludwigstr. 43, Bad Nauheim, 61231 Germany; 7 Max Planck Institute for Heart and Lung Research, Bioinformatics, Ludwigstr. 43, Bad Nauheim, 61231 Germany; 8 German Center for Cardiovascular Research (DZHK), Rhine-Main site, Frankfurt, Bad Nauheim, Germany; 9 Faculty of Biochemistry and Molecular Medicine, University of Oulu, Kontikangas Campus, Aapistie 7A, Oulu, 90220 Finland; 10 Department of Cardiovascular Therapeutics, TRON - Translational Oncology at the University Medical Center of Johannes Gutenberg University gGmbH, Freiligrathstrasse 12, Mainz, 55131 Germany

**Keywords:** Dormancy, Endothelial, PEAR1, LOXL2, CTSD, p27, Metastasis

## Abstract

In many cancer patients, distant metastases develop after years of dormancy. Understanding how disseminated tumor cells (DTCs), which are often found in proximity to the microvasculature, remain dormant and what regulates their reactivation is one of the major challenges in tumor biology. In a screen for endothelial secreted and plasma membrane proteins able to regulate tumor cell dormancy, we identified the transmembrane protein platelet and endothelial aggregation receptor 1 (PEAR1). Human and murine endothelial cells lacking PEAR1 lost the ability to promote dormancy of different tumor cells, and the extracellular part of PEAR1 was able to rescue this effect. Similarly, in mice lacking PEAR1 in endothelial cells, tumor cell dormancy in the lung was reduced and tumor metastasis was increased. We found that PEAR1 induces tumor cell dormancy by binding lysyl oxidase like 2 (LOXL2) and cathepsin D (CTSD), which both inhibit tumor cell dormancy and promote tumor growth and metastasis. Tumor cells with suppressed CTSD expression showed increased dormancy and decreased metastatic potential in vivo. Our data identify a mechanism underlying tumor cell dormancy and suggest CTSD and LOXL2 as targets for approaches to promote dormancy.

**Supplementary Information:**

The online version contains supplementary material available at 10.1186/s12943-025-02488-3.

## Introduction

Minimal residual disease (MRD) resulting from the existence of dormant tumor cells poses a significant challenge to achieving complete remission in cancer patients [1]. Dormant tumor cells can persist asymptomatically and remain undetected for long periods after treatment by employing conserved mechanisms of cell survival and cell cycle arrest [2–5]. Tumor cell dormancy is a collective term for cells in a reversible state of cell cycle arrest [6–8]. Because of their capacity to reactivate and re-enter the cell cycle, tumor cells undergoing cellular dormancy are particularly significant in the context of MRD and metastatic relapse [2]. Tumor cell dormancy is sustained by a variety of factors and is characterized by elevated levels of the cyclin-dependent kinase inhibitor p27, which inhibits progression from G_0_ to G_1_ and from G_1_ to S phase of the cell cycle [8, 9].

Cellular tumor dormancy is believed to depend on a local microenvironment, the dormancy niche [1, 10, 11]. Despite recent progress in identifying cell-autonomous and cell-extrinsic factors, which regulate tumor cell dormancy locally [8, 12], the molecular and cellular mechanisms of cellular tumor dormancy are still poorly understood. Studies in various animal models indicate that dormant tumor cells are often found in the perivascular space near endothelial cells [13, 14], and endothelial cells appear to serve as a crucial source of dormancy mediators [15, 16]. A pioneering study explored the interaction of endothelial and tumor cells in this context and identified thrombospondin-1 (THBS1) as an endothelial-derived mediator of tumor dormancy [13]. The goal of the current study was to identify and to characterize novel endothelial factors that can induce cellular tumor dormancy *in vitro* and *in vivo*.

## Results

### Endothelial cells induce tumor cell dormancy *in vitro*

**Fig. 1:**
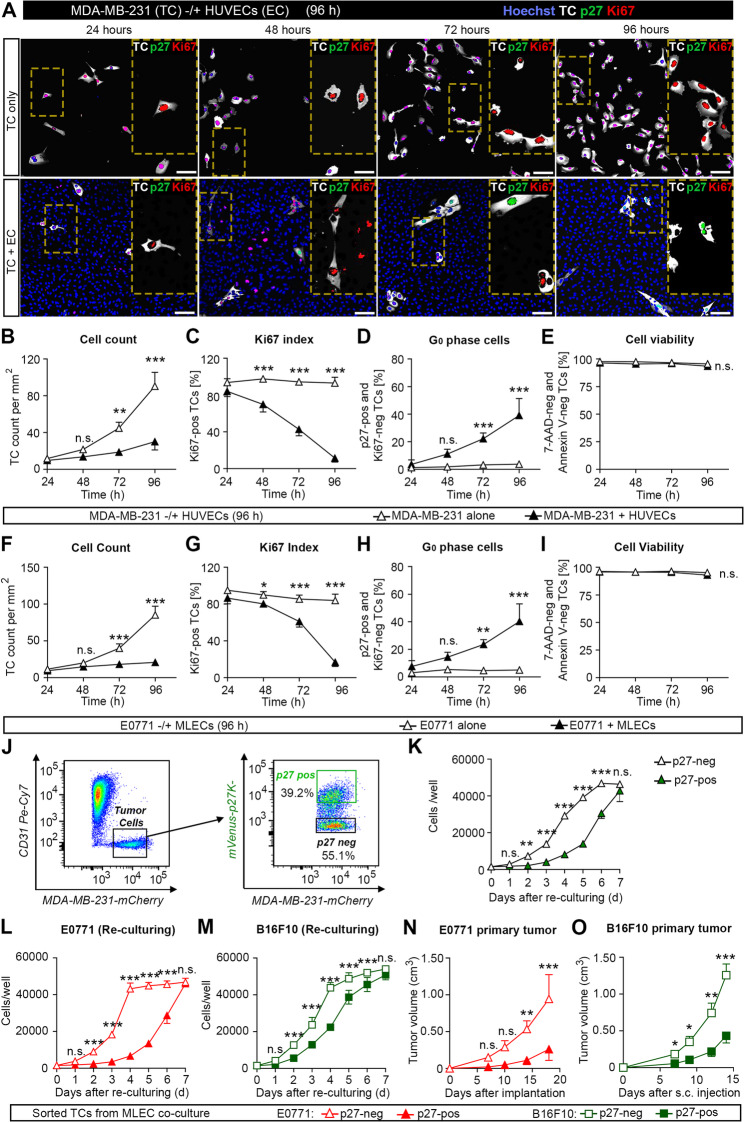
Endothelial cells induce tumor cell dormancy *in vitro*. **A**-**E**, Human MDA-MB-231 breast cancer cells co-expressing mCherry-luciferase and mVenus-p27K^−^ were cultured alone or together with HUVECs for 96 h in a direct co-culture system. Shown in (A) is a representative immunohistochemical staining with Hoechst 33,342 and with antibodies directed against mCherry to detect tumor cells (TC), against mVenus to detect the mVenus-p27K^−^ fusion protein (p27) and against Ki67 to detect proliferating cells with magnified areas indicated by a box with dashed lines. (B-E) show the quantification of tumor cell count (B), percentage of Ki67-positive cells (C), percentage of p27-positive and Ki67-negative cells (D) as well as percentage of live cells as indicated by 7-AAD and annexin V negativity (E) after 1–4 days of culture (*n* = 3 independent experiments). Scale bars: 100 μm. **F**-**I**, E0771 mouse tumor cells co-expressing mCherry-luciferase and mVenus-p27K^−^ were cultured alone or together with mouse lung endothelial cells (MLECs) for 4 days. Shown are the quantifications of the tumor cell number (F), the percentage of Ki67-positive (F), the percentage of p27-positive and Ki67-negative cells (H), and the percentage of alive, 7-AAD-negative and annexin V-negative cells (I) after 1–4 days of culture (*n* = 3 independent experiments). **J**, **K**, MDA-MB-231 tumor cells co-cultured with HUVECs were sorted to obtain cells positive and negative for mVenus-p27K^−^, and both cell fractions were cultured again in the absence of endothelial cells. Shown is the sorting strategy (J) as well as the cell number per well (K) during 7 days of culture (*n* = 3 independent experiments). **L-O**, E0771 and B16F10 mouse tumor cells co-expressing mCherry-luciferase and mVenus-p27K^−^ and co-cultured with MLECs for 4 days were sorted to obtain cells negative or positive for mVenus-p27K^−^. Both cell fractions were then cultured alone for 7 days, and cells per well were counted (L, M), or both fractions were injected orthotopically into the mammary fat pad of wild-type mice (N) or subcutaneously into wild-type animals (O), and tumor growth was monitored for 18 and 14 days, respectively (*n* = 4 independent experiments (L, M); *n* = 4 mice per group (N, O)). Shown are mean values ± S.E.M.; ^*^, *P* ≤ 0.05; ^**^, *P* ≤ 0.01; ^***^, *P* ≤ 0.001; n.s., non-significant (2-way ANOVA with Bonferroni’s post-hoc test (B-I, K-L, N); 2-way ANOVA repeated measures with Bonferroni's post-hoc test (M, O))

To recapitulate endothelial-cell-induced tumor cell dormancy *in vitro* and to identify endothelial proteins, which can induce tumor cell dormancy, we established a 2D co-culture assay wherein primary human umbilical vein endothelial cells (HUVECs) were cultured with human tumor cells expressing a mutant reporter of p27 (mVenus-p27K^−^) to detect tumor cells that have entered the G0 phase of the cell cycle [17]. Tumor cells negative for Ki67 and positive for mVenus-p27K^−^ were defined as dormant. We then compared the growth of MDA-MB-231 breast cancer cells seeded in the absence or presence of HUVECs over the course of 96 h. Whereas MDA-MB-231 cells proliferated extensively when cultured alone, growth of tumor cells co-cultured with HUVECs was markedly suppressed as indicated by the decrease in tumor cell count and percentage of Ki67-positive tumor cells as well as by an increase in p27-positive cells while cell viability as detected with 7-amino actinomycin D (7-AAD) was unchanged (Fig. 1A-E). Tumor cell dormancy was further indicated by an increased ratio of phosphorylated p38 and phosphorylated ERK1/2 as well as by increased *NR2F1* expression (Suppl. Figure 1 A and B). The same effects were seen when human PC3 prostate cancer cells and MEWO melanoma cells were co-cultured with HUVECs (Suppl. Figure 2A-H). Similarly, mouse E0771 breast cancer cells, RM1 prostate cancer cells, and B16F10 melanoma cells, when co-cultured with mouse lung endothelial cells (MLECs), showed decreased proliferation and an increased fraction of p27-positive cells (Fig. 1F-I and Suppl. Figure 2I-P). In all tested tumor cells cocultured with endothelial cells, we observed 30–50% of cells which were both Ki67- and p27-negative (Suppl. Figure 3). These cells are non-proliferating cells that are not dormant.

To determine whether tumor cells that had become dormant upon co-culturing with HUVECs are reactivatable *in vitro*, we re-cultured sorted mVenus-p27K^−^-positive MDA-MB-231 cancer cells in the absence of endothelial cells. The p27-positive MDA-MB-231 cells showed a delayed onset of growth compared to p27-negative cells but started to proliferate with a 48 h delay (Fig. 1J and K). Also, mVenus-p27K^−^-positive E0771 mouse breast cancer cells and B16 melanoma cells isolated from co-cultures with MLECs started to proliferate *in vitro* when cultured alone (Fig. 1L and M) and showed primary tumor growth *in vivo* (Fig. 1N and O) suggesting that dormant tumor cells can be reactivated.

### Identification of PEAR1 and CCL2 as endothelial-derived dormancy mediators

**Fig. 2:**
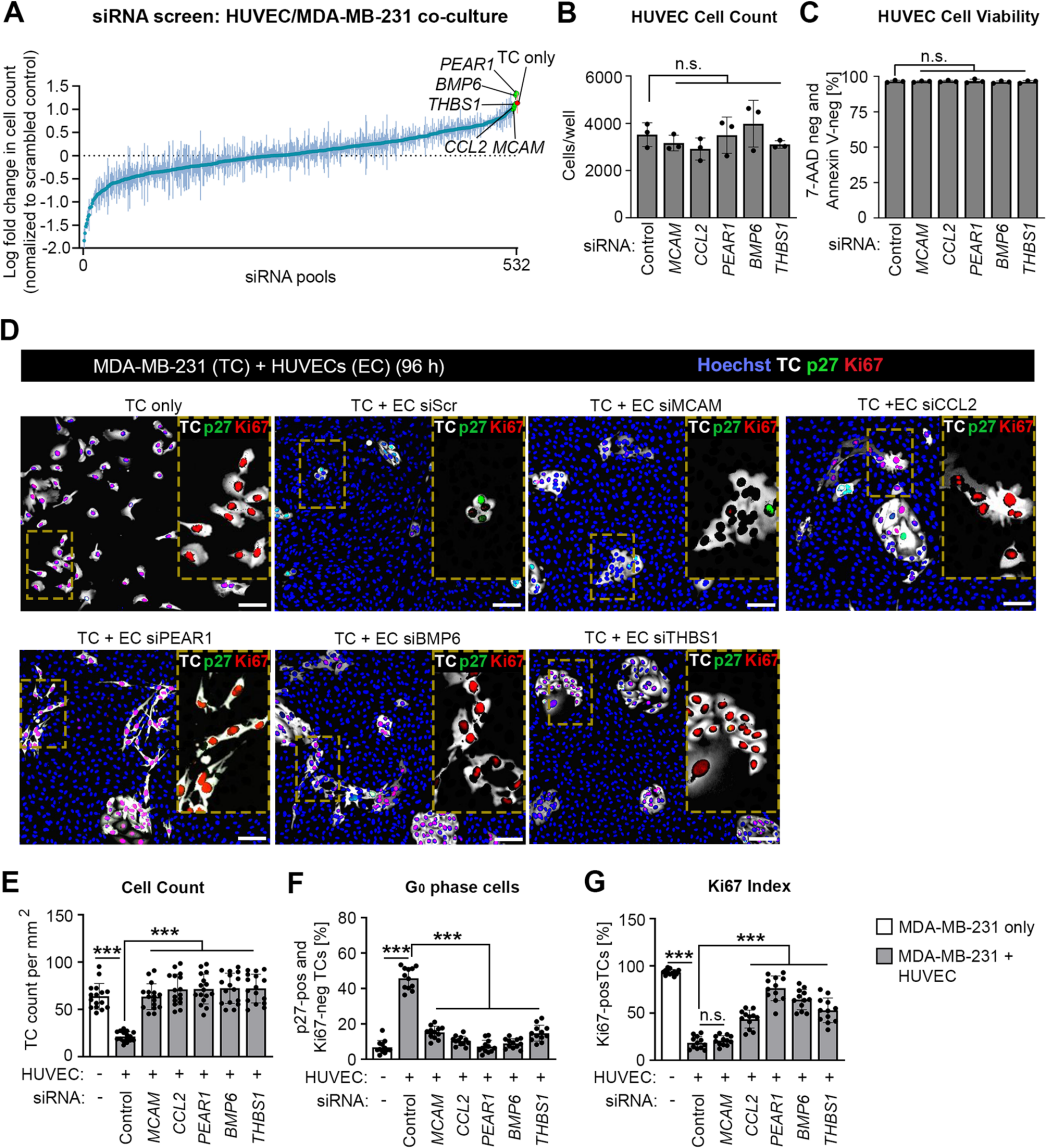
Identification of PEAR1 and CCL2 as mediators of endothelium-induced tumor cell dormancy. **A**, HUVECs were transfected with control siRNA or siRNAs against RNAs encoding surface receptors and secreted proteins highly expressed in HUVECs. Thereafter, cells were co-cultured with MDA-MB-231 cancer cells for 96 h and numbers of tumor cells were determined. Shown is the ratio of the cell count of MDA-MB-231 cells co-cultured with HUVECs transfected with a control siRNA and with each of the individual siRNA. The plot shows the ranked average ratios of 4 independent experiments. **B**, **C**, HUVECs were transfected with control siRNA or siRNAs directed against *MCAM*, *CCL2*, *THBS1*, *BMP6*, and *PEAR1*. 24 h later, cells were counted (B) or the percentage of 7-AAD-negative and annexin V-negative, alive cells was determined (C) (*n* = 3 independent experiments). **D**-**G**, MDA-MB-231 human tumor cells co-expressing mCherry-luciferase and mVenus-p27K^−^ were cultured alone or together with HUVECs transfected with control siRNA or siRNAs directed against *MCAM*, *CCL2*, *THBS1*, *BMP6*, and *PEAR1*, and cells were analyzed 4 days later. Shown in (D) are representative immunohistochemical images stained with Hoechst 33,342 and with antibodies directed against mCherry (TC), mVenus-p27K^−^ (p27) and Ki67 with magnified areas indicated by a box with dashed lines. Bar diagrams show the quantification of cell numbers (E), percentage of p27-positive and Ki67-negative (F) and of Ki67-positive tumor cells (G) (*n* = 16 (E) and 12 (F and G) independent experiments). Scale bars: 100 μm. Shown are mean values ± S.D. (A) or S.E.M. (B, C, E-G); ^***^, *P* ≤ 0.001; n.s., non-significant (one-way ANOVA and Tukey’s multiple comparison test)

To identify surface or secreted proteins of endothelial cells which are responsible for inducing tumor cell dormancy, we employed an siRNA screen targeting 532 RNAs encoding transmembrane and secreted proteins highly expressed in HUVECs. We identified PEAR1, bone morphogenetic protein 6 (BMP6), THBS1, CC-chemokine ligand 2 (CCL2), and melanoma cell adhesion molecule (MCAM) as potential mediators since siRNA-mediated knock-down of these proteins reversed the HUVEC-induced decrease in MDA-MB-231 cell count by at least 1 log-fold change (Fig. 2A and Suppl. Table 1) but had no effect on basal proliferation and cell death of HUVECs (Fig. 2B and C). Of these 5 candidates, PEAR1, BMP6, THBS1 and CCL2 could be validated as their knock-down in HUVECs allowed tumor cells to regain proliferation with an increased Ki67 index while having a decreased percentage of p27-positive and Ki67-negative tumor cells (Fig. 2D-G and Suppl. Figure 4 A). Knock-down of MCAM in contrast did not lead to an increased Ki67 index (Fig. 2D and G). As THBS1 and BMPs are already known to promote tumor dormancy [8, 13, 18], we continued to investigate the role of PEAR1 and the chemokine CCL2 in endothelium-mediated induction of dormancy of various other tumor cell types. Human PC3 prostate cancer cells, when co-cultured with HUVECs, in which PEAR1 or CCL2 was knocked down showed increased proliferation and decreased dormancy compared to PC3 cells cultured together with control HUVECs (Suppl. Figure 4A-E). Mouse E0771 breast cancer cells and mouse B16F10 melanoma cells, when co-cultured with MLECs with suppressed expression of *Pear1*, re-entered the cell cycle and proliferated compared to cells co-cultured with control MLECs (Suppl. Figure 4A-E). However, both E0771 and B16F10 tumor cells did not show an increased percentage of Ki67^+^ cells in co-culture with MLECs with suppressed *Ccl2* expression, although p27 expression was reduced (Suppl. Figure 4B-E).

### Endothelium-specific loss of PEAR1, but not of CCL2, reduces tumor cell dormancy and accelerates metastasis *in vivo*

**Fig. 3:**
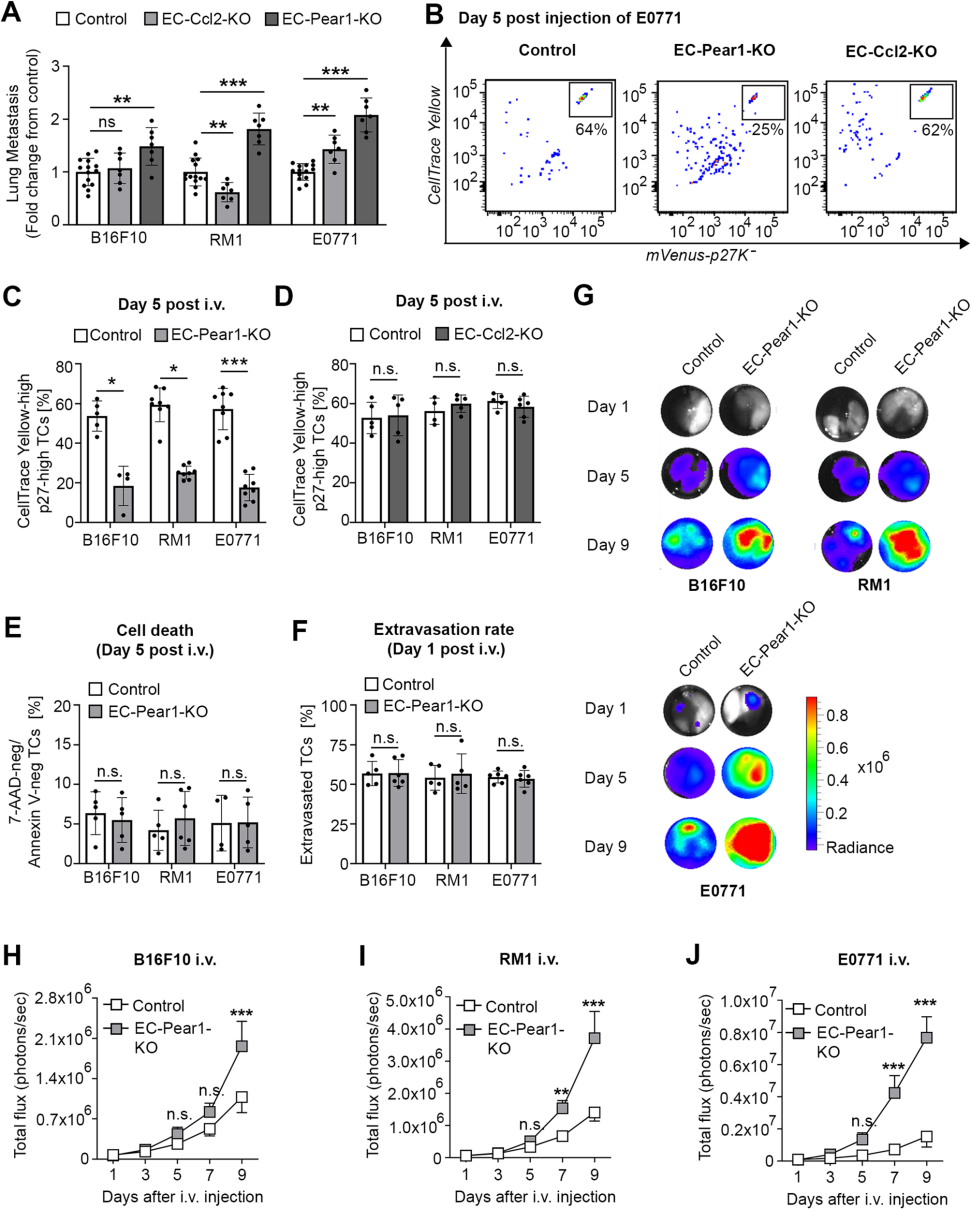
Endothelial loss of PEAR1 reduces tumor cell dormancy and promotes metastasis. **A**, B16F10, RM1 or E0771 mouse tumor cells were i.v. injected into wild-type, EC-Ccl2-KO or EC-Pear1-KO mice. Lung metastasis was analyzed by quantifying the number of visible nodules 12 days after i.v. injection. Shown is the fold change in the two knock-out lines compared to wild-type animals (n = 14 mice per group (Control); n = 7 mice per group (EC-Ccl2-KO and EC-Pear1-KO). **B**-**D**, E0771, RM1 and B16F10 mouse tumor cells co-expressing mCherry-luciferase and mVenus-p27K^−^ and loaded with the cell division tracking dye CellTrace Yellow were i.v. injected into control, EC-Pear1-KO or EC-Ccl2-KO animals. 5 days after the injection, animals were euthanized and tumor cells in lungs were analyzed. Shown in (B) are representative flow cytometric analysis plots of mCherry-positive tumor cells from digested lungs with mVenus-p27K^−^ intensity plotted against the CellTrace Yellow intensity. The bar diagrams in (C) and (D) show the statistical analysis of the percentage of CellTrace Yellow-high and p27-positive tumor cells in EC-Pear1-KO (C) and in EC-Ccl2-KO (D) compared to control animals (*n* = 4 mice per group (B16F10 for EC-Pear1-KO and RM1 control group in (D)); *n* = 5 mice per group (B16F10 control group in (C), and B16F10 for EC-Ccl2-KO and corresponding control, RM1 EC-Ccl2-KO, and E0771 control group in (D)); *n* = 6 mice (E0771 (EC-Ccl2-KO)); n = 8 mice per group (RM1 and E0771 (EC-Pear1-KO and corresponding control))). **E**, **F**, The indicated mouse tumor cells loaded with the cell division tracker dye CFSE (F) were i.v. injected into control or EC-Pear1-KO mice, and mouse lungs were analyzed by flow cytometry 5 days after i.v. injection (E) or were analyzed histologically 1 day after i.v. injection. Shown is the percentage of alive, 7-AAD-negative and annexin V-negative, tumor cells (E) or the percentage of extravasated tumor cells after staining of lung sections with DAPI and anti-CD31 antibodies (F) (*n* = 4 mice (E0771 in E), *n* = 5 mice (B16F10 in E, B16F10 control in F, RM1 control in E, RM1 in F and E0771 EC-Pear1-KO in E), *n* = 6 mice (RM1 EC-Pear1-KO in E, B16F10 EC-Pear1-KO in F and E0771 in F)). **G**-**J**, The indicated tumor cells co-expressing mCherry-luciferase and mVenusp-27K^−^ were i.v. injected into control or EC-Pear1-KO mice. 1, 5 or 9 days after the injection, mice were euthanized, and isolated lungs were analyzed by bioluminescence imaging. Shown are representative bioluminescent images of lungs (G) at the end of each experiment as well as the statistical analysis of photon flux of lungs at the indicated time points after i.v. injection of B16F10 (H), RM1 (I), or E0771 (J) mouse tumor cells (*n* = 4 mice per group). Shown are mean values ± S.E.M.; *, p ≤ 0.05; **, p ≤ 0.01; ***, p ≤ 0.001; n.s., non-significant (one-way ANOVA with Tukey’s multiple comparison test (A); Mann-Whitney U test (C: B16F10, E: E0771); two-tailed, unpaired t-test with Welch’s correction (C: RM1); two-tailed, unpaired t-test (C: E0771, D-F); 2-way ANOVA with Bonferroni’s post-hoc test (H-J))−−−

As knock-down of endothelial PEAR1 and, depending on the tumor cell line, also CCL2 inhibited the ability of endothelial cells to induce tumor cell dormancy *in vitro*, we generated conditional knock-out mice lacking either PEAR1 or CCL2 in endothelial cells by crossing *Pear1*^*flox*/flox^ or *Ccl2*^*flox*/flox^ mice with Cdh5-CreER^T2^ mice (Suppl. Figure 4F). After intravenous injection of E0771, RM1 or B16F10 tumor cells, Cdh5-CreER^T2^;*Pear1*^flox/flox^ mice (EC-Pear1-KO) showed a significantly higher number of lung metastasis compared to the controls in all tumor types (Fig. 3A and Suppl. Figure 5A). However, in Cdh5-CreER^T2^;*Ccl2*^flox/flox^ mice (EC-Ccl2-KO) only E0771 cells showed an increased number of lung metastases after i.v. injection, whereas numbers were unchanged after injection of B16F10 cells and were decreased when RM1 cells were tested (Fig. 3A and Suppl. Figure 5A). To further investigate whether the observed phenotype was due to loss of tumor cell dormancy, we injected tumor cells expressing mCherry-luciferase and mVenus-p27K^−^ which, in addition, were loaded with the cell division tracking dye CellTrace Yellow (CTY) to monitor possible dormancy during lung metastasis. In parallel to the observed increase in lung metastases, EC-Pear1-KO mice showed an increased loss of CTY^high^ p27^high^ dormant tumor cells in all tumor cell types on day 5 compared to the corresponding controls (Fig. 3B and C). In contrast, in EC-Ccl2-KO mice the degree of tumor cell dormancy was unchanged (Fig. 3B and D). We therefore decided to focus our investigation on the role of endothelial PEAR1 in the regulation of tumor cell dormancy. In EC-Pear1-KO mice, we found unchanged tumor cell death 5 days after i.v. injection and tumor cell extravasation 1 day after i.v. injection compared to the controls (Fig. 3E and F and Suppl. Figure 5B), whereas *in vivo* bioluminescence imaging of luciferase-expressing tumor cells showed increased lung metastatic burden starting from day 5 (Fig. 3G-J).

**Fig. 4:**
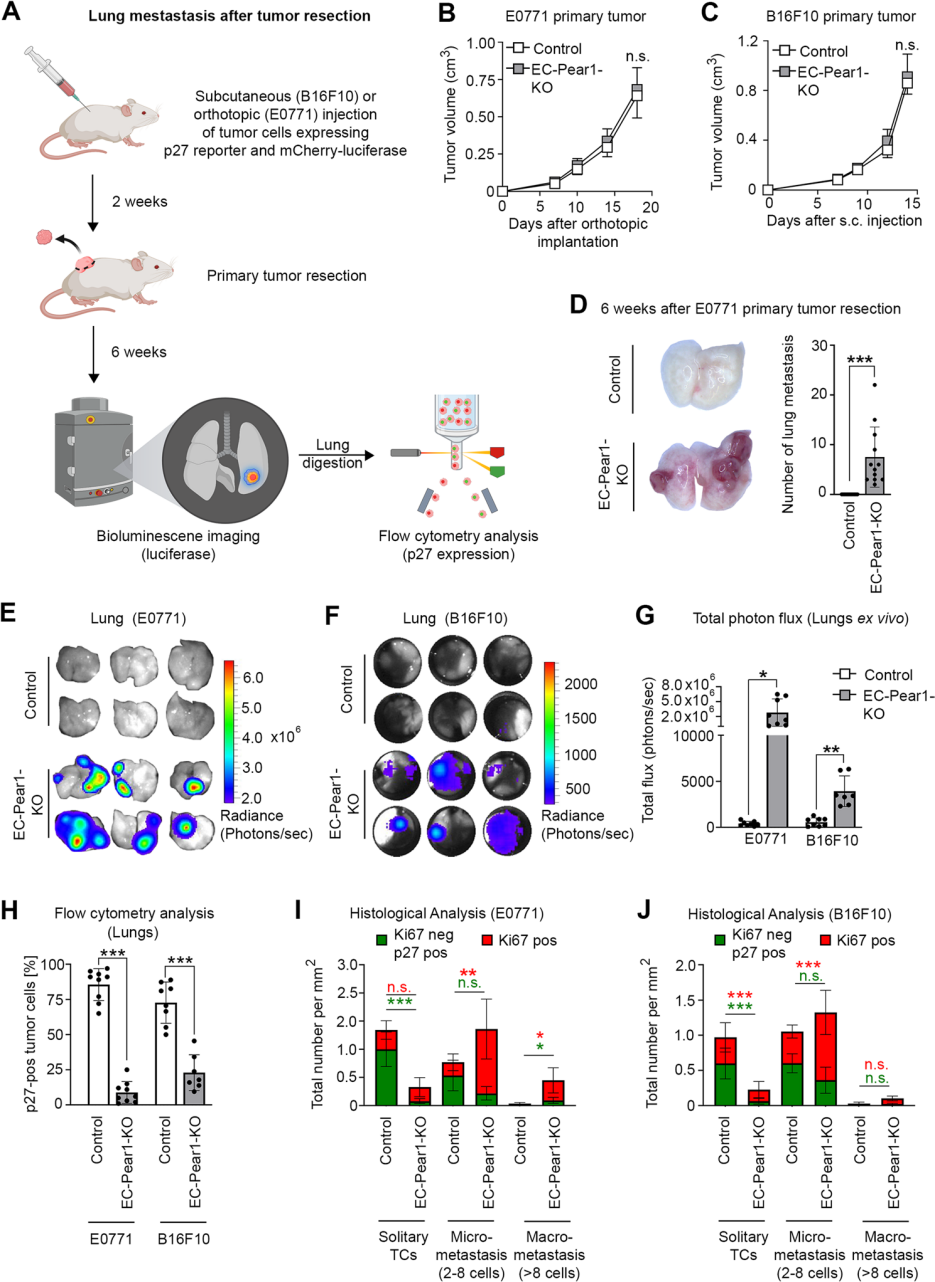
Loss of endothelial PEAR1 promoted lung metastasis from primary tumors. **A**, E0771 of B16F10 tumor cells co-expressing mCherry-luciferase and mVenus-p27K− and isolated from stand-alone cultures were injected orthotopically or subcutaneously, respectively, into EC-Pear1-KO or control animals to allow for primary tumor formation for 18 and 14 days, respectively. After resection of the primary tumor, animals were kept for another 6 weeks. Thereafter, mice were euthanized and analyzed. **B**, **C**, Shown are quantifications of the growth of E0771 (B) and B16F10 (C) primary tumors using a microcaliper (*n* = 9 mice per group in (B); *n* = 10 mice per group in (C)). **D**-**G**, Shown are representative images of lungs 6 weeks after E0771 primary tumor resection, the quantification of macrometastatic nodules (*n* = 12 mice per group) (D), representative bioluminescent images of lungs 6 weeks after resection of E0771 (E) and B16F10 primary tumors (F) and the statistical analysis of photon flux of lungs (G) (*n* = 8 mice per group (E0771 for all groups, B16F10 (control group); *n* = 7 mice (B16F10 (EC-Pear1-KO)). **H**, Flow cytometric quantification of the percentage of p27-positive tumor cells in lung digests from EC-Pear1-KO and control animals 6 weeks after primary tumor resection (n = 8 mice per group (E0771 for all groups, B16F10 (control group); *n* = 7 mice (B16F10 (EC-Pear1-KO)). **I**, **J**, Cryosections of whole lungs taken 6 weeks after tumor resection were stained with DAPI and antibodies against mVenus-p27K^−^, against mCherry to identify tumor cells, and against Ki67, and a comprehensive analysis of whole lung sections was conducted. Tumor cells (TC) were grouped into three categories: solitary (1 cell) and those present in micrometastasis (2–8 cells) and macrometastasis (more than 8 cells). The bar graphs illustrate the total counts of proliferating (Ki67-positive) and dormant (Ki67-negative and p27-positive) E0771 (I) and B16F10 (J) tumor cells per area that have metastasized to the lung 6 weeks after resection in both EC-Pear1-KO and control mice (*n* = 6 mice per group; 3 sections per mice). Shown are mean values ± S.E.M.; *, P ≤ 0.05; **, P ≤ 0.01; ***, P ≤ 0.001; n.s., non-significant (2-way ANOVA repeated measures with Bonferroni’s post-hoc test (B-C); Mann-Whitney U test, unpaired, one-tailed (D); Mann-Witney U test, unpaired, two-tailed (H: E0771) unpaired t-test; two-tailed with Welch’s correction (G); unpaired t-test, two-tailed (H: B16F10); 2-way ANOVA with Bonferroni’s post-hoc test (I: solitary TCs, J: solitary TCs and micrometastasis) and Kruskal-Wallis with Dunn’s multiple comparison test (I: microtetastasis and macrometatsis, J: macrometastasis))

Since loss of PEAR1 in endothelial cells led to an increased loss of dormant tumor cells after intravenous injection of parental E0771 cells, we tested whether this would also be the case after metastasis from a primary tumor (Fig. 4A). We therefore injected B16F10 and E0771 tumor cells expressing mCherry-luciferase and mVenus-p27K^−^ subcutaneously and orthotopically, respectively. After 14 or 18 days, respectively, primary tumors were resected, and spontaneous lung metastases and tumor dormancy were assessed 6 weeks post-resection. EC-Pear1-KO mice had unchanged primary tumor growth compared to the controls (Fig. 4B and C) but had a significant increase in lung metastatic nodules and overall metastatic burden (Fig. 4D-G), which were visible in the case of E0771 tumors both as micro- and macrometastases (Fig. 4D, E and G) and in the case of B16F10 tumors only as micrometastases (Fig. 4F, G and Suppl. Fig. 5C). We observed a corresponding decrease in p27-positive dormant tumor cells in both tumor cell types (Fig. 4H). Histologically, relatively high numbers of dormant (Ki67^neg^ p27^pos^) tumor cells were observed as solitary tumor cells or in micrometastases in the lungs of control mice, but these numbers were significantly decreased in EC-Pear1-KO mice 6 weeks post-resection (Fig. 4I and J and Suppl. Figure 5C).

**Fig. 5:**
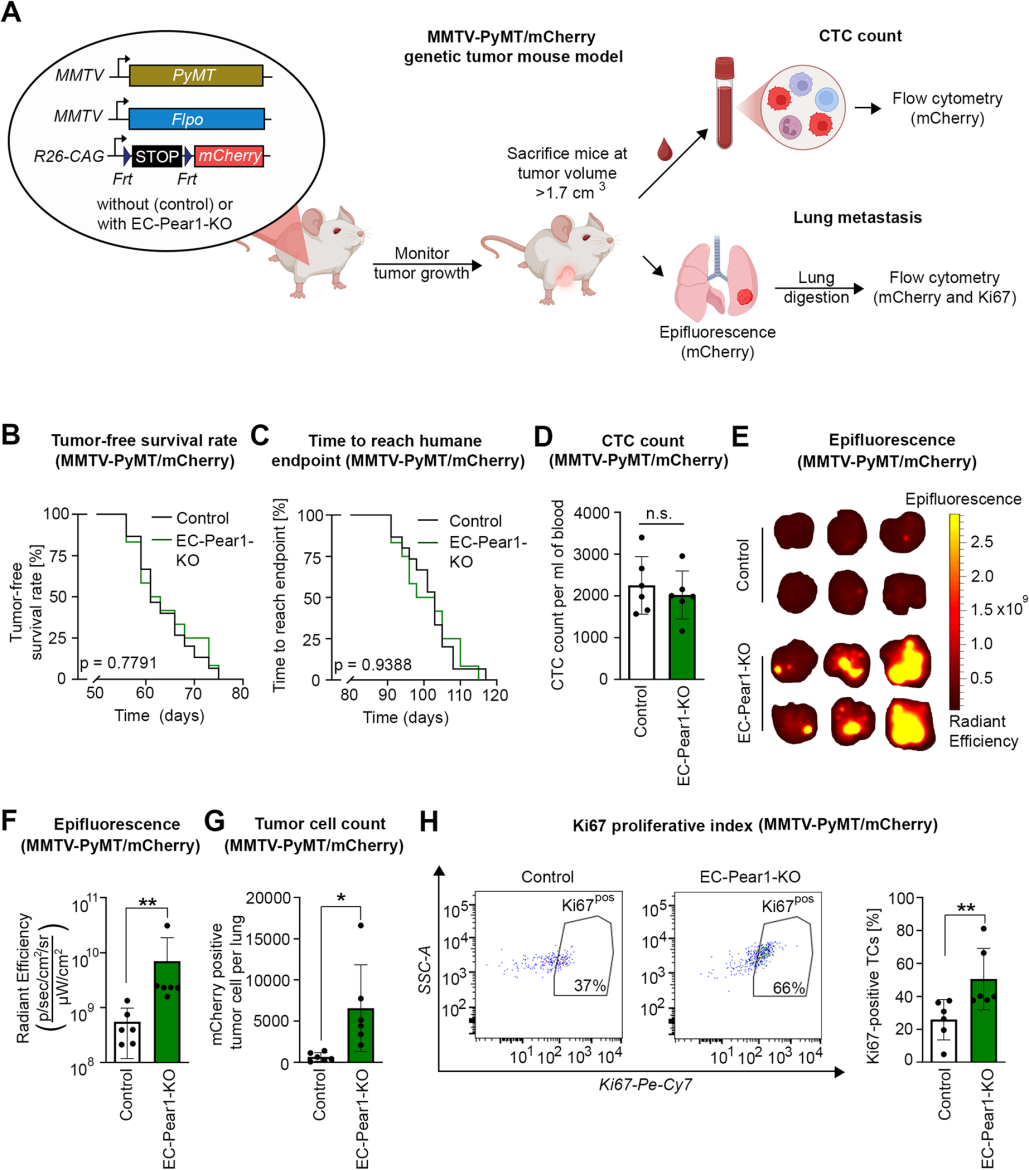
Endothelium-specific KO of Pear1 promoted lung metastasis in the spontaneous MMTV-PyMT mouse model. **A**, Schematic representation of the experimental strategy. MMTV-PyMT mice without (control) or with endothelial Pear1 deficiency (EC-Pear1-KO), which in addition express the Flp recombinase under the control of the MMTV promoter as well as the mCherry-expressing Flp reporter RC::FrePe, which allows to mark disseminated MMTV-PyMT breast cancer cells with mCherry fluorescence, were generated. Once MMTV-PyMT mice reached the humane endpoint, circulating tumor cells (CTC) as well as lung metastases were analyzed. **B**, **C**, Shown are the tumor-free survival rate (B) and the overall time after tumor occurrence until the humane endpoint was reached (C) (*n* = 15 mice (control group); *n* = 12 mice (EC-Pear1-KO)). **D**-**F**, Upon reaching the humane endpoint, mice were euthanized, blood samples were taken intracardially, and lungs were collected for analysis using epifluorescence imaging for mCherry. Shown in (D) is the number of mCherry-positive tumor cells per milliliter blood obtained from EC-Pear1-KO and control PyMT mice (*n* = 6 mice per group). Representative epifluorescent images of the lungs and the statistical analysis of mCherry epifluorescence at the endpoint are shown in (E) and (F) respectively. **G**, **H**, Lung tissues were digested and analyzed by flow cytometry. Shown are the results from flow cytometric analysis, which quantified the total number of mCherry-positive breast cancer cells present in the lung (G) and the percentage of Ki67-positive, mCherry-positive breast cancer cells (H) (*n* = 6 mice per group). Shown are mean values ± S.E.M.; ^**^, *P* ≤ 0.01; n.s., non-significant (two-sided log-rank test for Kaplan-Meier curves (B, C); two-tailed, unpaired t-test (D); Mann-Whitney U test (F, H); two-tailed, unpaired t-test with Welch’s correction (G))

We also checked the effect of endothelium-specific loss of PEAR1 in a genetic mouse tumor model, the mouse mammary tumor virus (MMTV)-polyomavirus middle T antigen (PyMT) breast cancer model. EC-Pear1-KO animals were crossed with MMTV-PyMT mice as well as with animals expressing flp recombinase under the control of the MMTV promoter, which in addition express the flp-recombinase responsive fluorescent reporter allele RC::FrePe (Fig. 5A and Suppl. Figure 6A). In this mouse line, breast cancer cells express mCherry fluorescence to visualize and detect both circulating and metastatic tumor cells via flow cytometry and with an *in vivo* imaging system (IVIS). Similar to the resection model, primary tumor growth was unchanged in EC-Pear1-KO mice as there was no difference in tumor-free survival and overall survival rates compared to controls (Fig. 5B and C). There was also no difference in circulating tumor cells in EC-Pear1-KO MMTV-PyMT mice compared to controls (Fig. 5D and Suppl. Figure 6B). However, consistent with all the previous mouse models, there was an increased lung metastatic burden in EC-Pear1-KO mice indicated by an increase in the mCherry epifluorescence IVIS signal (Fig. 5E and F), increased total tumor cell count in the lungs (Fig. 5G) and an increased Ki67 proliferative index of tumor cells in the lungs (Fig. 5H and Suppl. Figure 6C) compared to controls.

### PEAR1 sequesters CTSD and LOXL2 to induce tumor cell dormancy

**Fig. 6:**
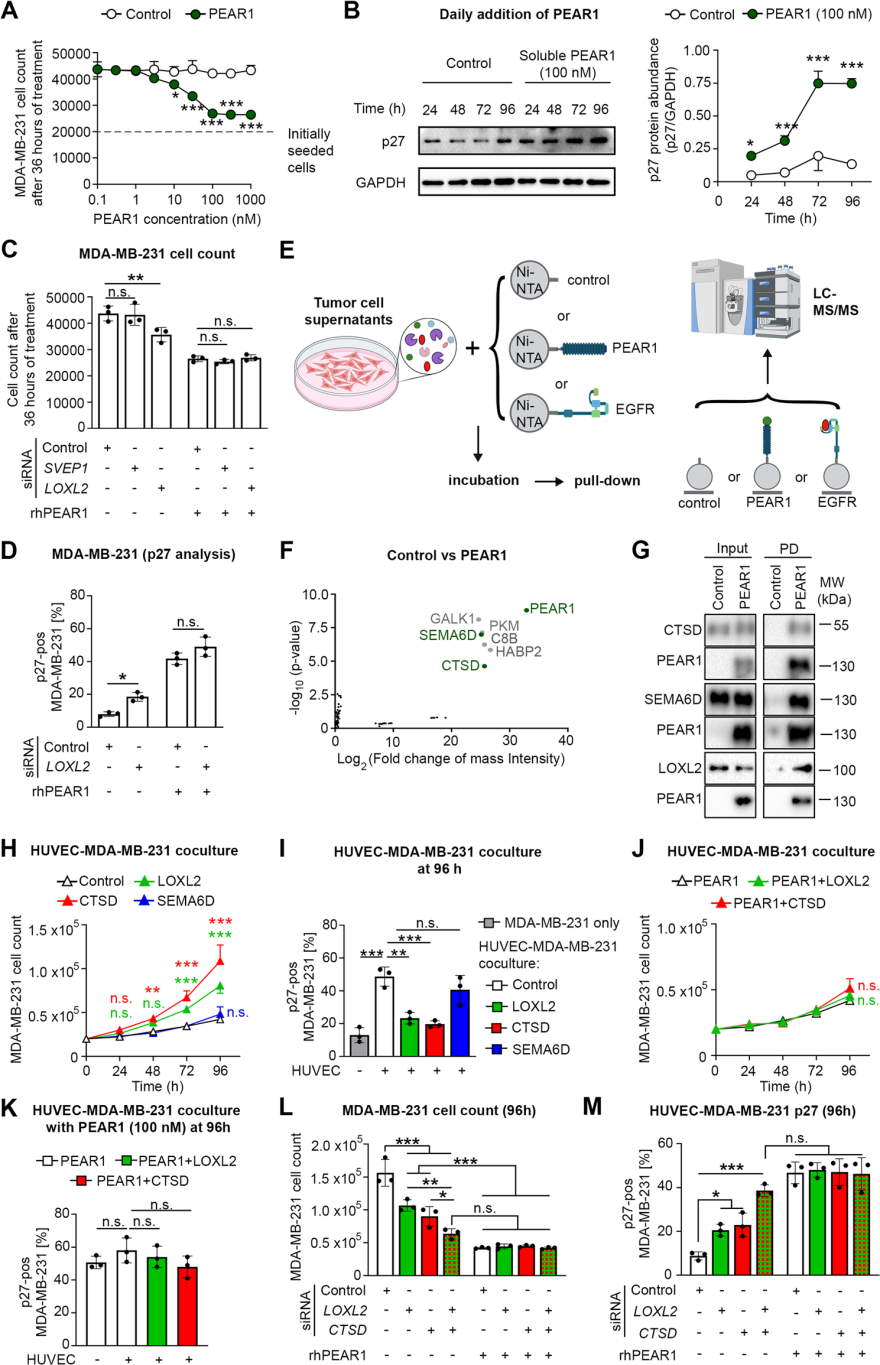
PEAR1 sequesters CTSD and LOXL2 to promote tumor cell dormancy. **A**, MDA-MB-231 cells were treated with increasing concentrations of the extracellular domain of recombinant human PEAR1 containing a His-tag and immobilized on Ni-NTA beads. Cells incubated with Ni-NTA beads only served as a control group. Cell count was determined by flow cytometry analysis (*n* = 3 independent experiments). **B**, MDA-MB-231 cells were treated daily for 4 days with PEAR1 immobilized on Ni-NTA beads, and cells were analyzed by immunoblotting using anti-GAPDH and anti-p27 antibodies. Shown is a representative immunoblot and the statistical analysis (*n* = 3 independently performed experiments). **C**, **D**, MDA-MB-231 cells expressing mVenus-p27K^−^ were transfected with control siRNA or siRNA directed against *SVEP1* or *LOXL2* and were incubated in the absence or presence of PEAR1 (100 nM). After 36 h, the cell count (C) and the percentage of p27-positive cells (D) was determined (*n* = 3 independent experiments). **E**, Procedure to search for interaction partners of PEAR1 within the conditioned medium of tumor cells using a pull-down assay with PEAR1 immobilized on Ni-NTA beads. Control Ni-NTA beads and the recombinant extracellular part of human epidermal growth factor receptor immobilized on Ni-NTA beads served as controls. **F**, The plot shows the proteins that were specifically co-precipitated with PEAR1 compared to control samples. Proteins only found with PEAR1 are indicated by green dots, and proteins found with both PEAR1 and EGFR are represented by grey dots. **G**, Recombinant CTSD, SEMA6D or LOXL2 were incubated alone or together with purified His-tagged PEAR1. PEAR1 was precipitated with Ni-NTA beads, and precipitates were analyzed by immunoblotting. Shown are representative of 3 independently performed experiments. **H**, **I**, MDA-MB-231 cells co-expressing mCherry-luciferase and mVenus-p27K^−^ were co-cultured with HUVECs for the indicated time periods, and cells were treated daily without or with CTSD (100 nM), LOXL2 (100 nM) or SEMA6D (1000 nM). The cell count (H) was assessed using flow cytometry. At the end of the experiment, the percentage of p27-positive cells (l) was determined by flow cytometry (*n* = 3 independent experiments). **J**, **K**, Co-cultures of HUVECs and MDA-MD-231 cells were incubated for the indicated time periods with 100 nM PEAR1 alone or in combination with 100 nM CTSD or LOXL2. Assessment of cell count (J) was conducted utilizing flow cytometry, and, after 4 days, the percentage of p27-positive cells (K) was analyzed by flow cytometry (*n* = 3 independent experiments). **L**, **M**, MDA-MB-231 cells co-expressing mCherry and mVenus-p27K^−^ were transfected with control siRNA or siRNA directed against CTSD, LOXL2 or both, and were cultured in the absence or presence of the recombinant extracellular part of human PEAR1 (rhPEAR1; 100 nM). After 96 h, cells were counted (L), and p27 expression was determined (M) (*n* = 3 independently performed experiments). Shown are mean values ± S.E.M.; ^*^, *p* ≤ 0.05; ^**^, *p* ≤ 0.01; ^***^, *p* ≤ 0.001; n.s., non-significant (2-way ANOVA and Bonferroni’s post-hoc test (A-D, H, J, L, M); multiple two-tailed t-test, Bayesian moderated, Benjamin, Krieger, and Yekutieli-corrected, false discovery rate = 1% (F); one-way ANOVA with Tukey’s multiple comparison test (I, K))

To determine how PEAR1 induces tumor cell dormancy, we tested whether the extracellular part of PEAR1 alone is able to promote tumor cell dormancy. Treatment of MDA-MB-231 cells with the His-tagged extracellular part of PEAR1 bound to agarose beads for 36 h led to a decrease in cell count with a maximal effect at 100 nM (Fig. 6A), to an increase in p27 protein levels (Fig. 6B) as well as to an increased ratio of phosphorylated p38 and ERK1/2 and an increased expression of *NR2F1* (Suppl. Figure 7A and B). PEAR1 treatment of MDA-MB-231 cells mimicked coculture of MDA-MB-231 and HUVECs and decreased tumor cell count and Ki67 index while resulting in an increased percentage of dormant Ki67-negative and p27-positive cells and unchanged cell viability (Suppl. Figure 8 A-D).

Previous reports showed that the membrane receptor PEAR1 binds to the transmembrane protein FCER1A and can also bind and sequester soluble proteins such as SVEP1 and LOXL2 [19–21]. In contrast to FCER1A, which is not expressed in tumor cells, we found expression of LOXL2 and low levels of SVEP1 (Suppl. Figure 8E). MDA-MB-231 cells with suppressed SVEP1 expression showed no difference in growth compared to control cells and still responded to PEAR1. However, knock-down of LOXL2 in MDA-MB-231 cells led to a reduced proliferation of tumor cells and an increase in the percentage of p27-positive MDA-MB-231 cells (Fig. 6C and D). This is consistent with the well-established role of LOXL2, which acts both extracellularly and intracellularly to promote tumor cell growth and metastasis and to inhibit tumor cell dormancy [22–24]. When compared to the effect of recombinant PEAR1 on tumor cell growth and expression of p27, the effect of a suppression of LOXL2 in tumor cells was much smaller (Fig. 6C and D), indicating that binding and sequestration of LOXL2 by PEAR1 can only partially explain the dormancy-inducing activity of PEAR1. We therefore searched for other interaction partners of PEAR1 and incubated supernatants of cultured tumor cells with the His-tagged extracellular part of PEAR1 bound to magnetic agarose beads. The bound proteins were then subjected to proteomic analysis using mass spectrometry (Fig. 6E). The extracellular domain of the EGF receptor (EGFR) bound to magnetic agarose served as a control. Several proteins such as C8B, GALK1, HABP2 and PKM were pulled down by both PEAR1 and EGFR (Fig. 6F and Suppl. Figure 8 F), suggesting that they interacted unspecifically. However, cathepsin D (CTSD) and semaphorin 6D (SEMA6D) were found after pull down with PEAR1-agarose beads (Fig. 6F) but not with EGFR agarose beads (Suppl. Figure 8 F). After PEAR1 was co-incubated with purified CTSD or SEMA6D, we were able to pull down PEAR1 with both proteins as well as with LOXL2 (Fig. 6G). When we tested the effect of CTSD, LOXL2 and SEMA6D on tumor cell growth and tumor cell dormancy, CTSD and LOXL2, but not SEMA6D, increased MDA-MB-231 tumor cell growth and decreased the percentage of p27-positive tumor cells in the presence of endothelial cells (Fig. 6H and I; Suppl. Figure 8G). Similarly, in E0771 and B16F10 mouse tumor cells cocultured with MLECs, CTSD and LOXL2, but not SEMA6D, concentration-dependently increased proliferation and decreased the percentage of p27-positive cells (Suppl. Figure 8 H-M). The effect of CTSD and LOXL2 on proliferation and p27 expression of MDA-MB-231 cells was not seen in the presence of equimolar concentrations of PEAR1 agarose (Fig. 6J and K). Finally, knock-down of CTSD in MDA-MB-231 cells led to a similar decrease in cell number and increase in the percentage of p27-positive MDA-MB-231 cells as well as knock-down of LOXL2 (Fig. 6L and M), and knock-down of both CTSD and LOXL2 had an additive effect, which was similar to the effect of recombinant PEAR1 (Fig. 6L and M). This indicates that CTSD and LOXL2 act independently of each other and that their sequestration by PEAR1 is responsible for the dormancy-promoting effect of PEAR1.

### CTSD inhibits tumor cell dormancy and promotes metastasis

Since LOXL2 is well known to inhibit tumor cell dormancy and to promote metastasis and tumor growth [21, 22, 25, 26], we focused our anlaysis on the function of CTSD. When incubating MDA-MB-231 tumor cells with CTSD, we found a concentration-dependent binding of CTSD to tumor cells (Suppl. Figure 9 A) as well as an uptake of CTSD by tumor cells (Suppl. Figure 9B-D). The cellular binding and uptake of CTSD was blocked by PEAR1 (Suppl. Figure 9B-D). Similar effects were seen with E0771 and B16F10 cells (Suppl. Figure 9E-H). This indicates that CTSD can act extracellularly as well as intracellularly as described previously [27, 28]. Since CTSD has previously been proposed to inhibit tumor cells quiescence by activation of mammalian target of rapamycin complex 1 (mTORC1) signaling [28], we tested the effect of extracellularly applied CTSD on signaling pathways in MDA-MB-231 cells and found that it induced phosphorylation of AKT and mTOR, an effect which was blocked by coapplication of PEAR1 (Suppl. Figure 9I). We also tested the dormancy-prone cell line D2.0R and its counterpart D2A1, which has no dormancy tendency [29]. The expression of p27 in D2.0R cells cocultured with mouse lung endothelial cells (MLECs) was dependent on the presence of endothelial PEAR1 expression, since knock-down of PEAR1 strongly reduced p27 protein levels in D2.0R cells (Suppl. Figure 10 A). When exposed to CTSD, p27 protein levels were reduced in the presence and absence of PEAR1. This is consistent with a model in which PEAR1 promotes tumor cell dormancy by binding CTSD. Decreased dormancy of D2.0R cells was always accompanied by increased phosphorylation of mTOR as well as phosphorylation of AKT and ERK1/2 (Suppl. Figure 10 A). The regulation of D2-0R tumor cell dormancy by PEAR1 and CTSD was also reflected by differences in cell count (Suppl. Figure 10B). When we did the same experiment with the control cell line D2A1 cocultured with MLECs, we found that the effects of PEAR1 and CTSD were reduced as expected for a tumor cell line not prone to undergo tumor cell dormancy (Suppl. Figure 10 C and D).

**Fig. 7:**
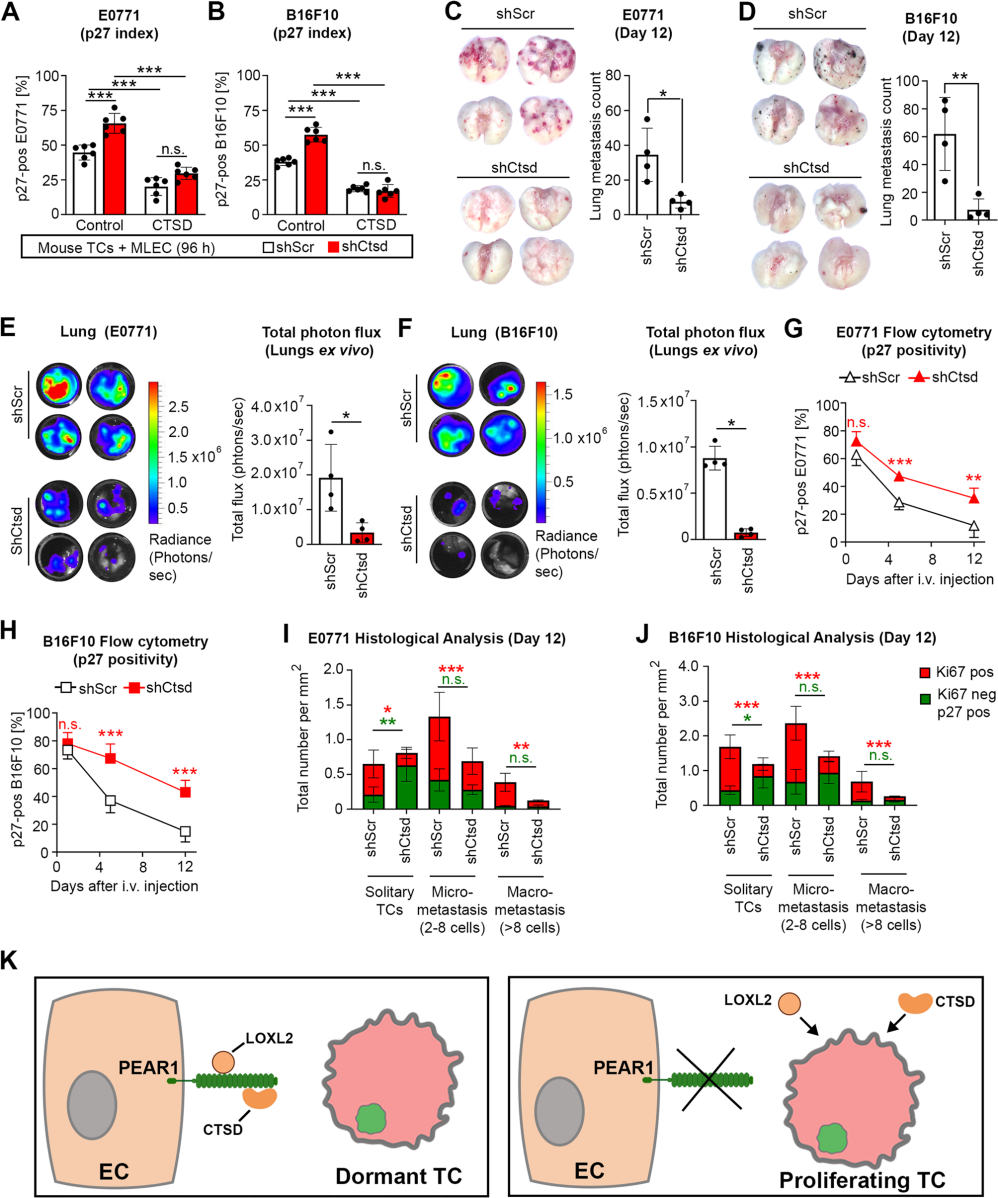
Suppression of CTSD in tumor cells increased tumor cell dormancy both *in vitro *and *in vivo*. **A**, **B**, The indicated tumor cells co-expressing mCherry-luciferase and mVenusp27K^−^ were subjected to stable transduction with either a scrambled control shRNA (shScr) or an shRNA targeting *Ctsd* (shCtsd). Subsequently, tumor cells were co-cultured with MLECs and treated daily with either a vehicle control or recombinant mouse CTSD (100 nM) over a period of 96 h, and the percentages of p27-positive E0771 (A) and B16F10 (B) cells were quantified (*n* = 3 independent experiments). **C**-**J**, The indicated tumor cells without or with stable knock-down of CTSD (shScr or shCtsd, respectively) co-expressing mCherry-luciferase and mVenus-p27K^−^ were i.v. injected into wild-type animals. Lung metastasis was assessed by determining the number of visible metastatic nodules 12 days after injection of E0771 (C) or B16F10 (D) as well as by analyzing isolated lungs by bioluminescence imaging (E and F) (*n* = 4 mice per group). The proportion of p27-positive tumor cells present in lung digests collected 1, 5, and 12 days after i.v. injection of E0771 (G) or B16F10 (H) was determined by flow cytrometry (*n* = 4 mice per group). Cryosections of whole lungs were stained with Hoechst 33342, and antibodies against mVenus, mCherry and Ki67. Thereafter, entire lung sections were imaged, enabling the categorization of tumor cells into three distinct groups: solitary tumor cells (TC) (1 cell) and those present in micrometastasis (2–8 cells) and macrometastasis (> 8 cells). The bar graphs show the total numbers of proliferating (Ki67-positive) and dormant (Ki67-negative and p27-positive) tumor cells per area for E0771 (I) and B16F10 cells (J) without (shScr) or with CTSD knock-down (shCtsd) (*n* = 4 mice per group; 3 sections per mice). **K**, Schematic representation of the proposed role of endothelial PEAR1 in promoting tumor cell dormancy. EC, endothelial cell; TC, tumor cell. Shown are mean values ± S.E.M.; ^*^, *P* ≤ 0.05; ^**^, *P* ≤ 0.01; ^***^, *P* ≤ 0.001; n.s., non-significant (2-way ANOVA and Bonferroni’s post-hoc test (A, B, G, H, I (solitary TCs and micrometastasis and J); two-tailed, unpaired t-test with Welch’s correction (C); two-tailed, unpaired t-test (D, E); Mann-Whitney U test, unpaired, two-tailed (F); Kruskal-Wallis test with Dunn’s multiple comparison test (I (macrometastasis))

To further test the role of CTSD in the regulation of tumor cell dormancy, mouse E0771 and B16F10 tumor cells with a stable shRNA-mediated knock-down of CTSD were generated and then tested *in vitro* and *in vivo *(Suppl. Figure 11 A and B). Tumor cell lines with CTSD knock-down exhibited increased p27 expression compared to controls when co-cultured with MLECs (Fig. 7A, B). Addition of CTSD reverted the effects of CTSD knock-down in all tumor cells tested (Fig. 7A, B). Immunocompetent mice intravenously injected with either E0771 or B16F10 with a stable knock-down of CTSD developed fewer metastasis (Fig. 7C-F) while having increased p27 expression compared to controls (Fig. 7G and H). Histologically, the numbers of dormant (Ki67^neg^;p27^pos^) tumor cells increased in the fraction of solitary tumor cells after knock-down of CTSD, and the number of proliferating tumor cells (Ki67^pos^) decreased both among the solitary tumor cells as well as in the micrometastases (Fig. 7I and J and Suppl. Figure 11 C).

## Discussion

The perivascular space is a site where dormant tumor cells reside, and endothelial cells contribute to the pro-dormant milieu of this niche by releasing factors which promote cellular tumor dormancy [15, 16]. These factors include thrombospondin-1 (TSP1), stem cell factor or extracellular vesicles [13, 30–32]. A more recent investigation assessing the interaction between tumor cells and endothelial cells revealed that Wnt ligands released from endothelial cells induce tumor latency in mouse models of lung metastasis [33]. These studies focused on secreted molecules but did not study potential contributions from endothelial surface receptors and possible direct interaction of tumor cells and endothelial cells that could influence tumor cell dormancy. In an attempt to identify additional endothelium-derived mediators of tumor cell dormancy, we established an *in vitro* system that allowed to screen for endothelial proteins that regulate tumor dormancy. We thereby identified PEAR1 as an endothelial transmembrane protein which promotes tumor cell dormancy *in vitro *and *in vivo* by binding and thereby inactivating the pro-proliferative proteins LOXL2 and CTSD (Fig. 7K).

A number of approaches have explored the dormancy-inducing niche or aimed at reconstruction of tumor dormancy *in vitro*, but mainly relied on secretome analyses, particularly regarding extracellular matrix (ECM) components. These studies led to the identification of COL3A1, COL17A1, THBS1, TGFB2, BMPs, and Laminin-211 as dormancy-inducing mediators [13, 34–38]. Among these known mediators, THBS1 and BMP6 were also found in our *in vitro* screen for tumor cell dormancy-inducing endothelial factors, reinforcing our confidence in the validity of our screening methodology. Our study eventually identified PEAR1 as a factor promoting tumor cell dormancy. In contrast to most other proteins implicated in the regulation of cellular tumor dormancy, PEAR1 is a transmembrane protein that is expressed by various cells including endothelial cells and platelets. PEAR1 is believed to play a role in platelet activation [39, 40] and to mediate the effect of heparin and heparin proteoglycan mimetics on platelets [41]. PEAR1 has also been described in tumor cells. In triple-negative cancer cells, PEAR1 has been shown to promote metastasis [21], and in acute myeloid leukemia cells its expression is correlated with poor progrnosis [42]. However, its exact physiological function remains poorly understood although it has been shown to interact with SVEP1, LOXL2 and FCER1A [19–21]. We were able to rule out that FCER1A or SVEP1 is involved in the observed tumor dormancy-inducing activity of PEAR1. Interaction of PEAR1 with LOXL2 appeared likely to be involved in the effects of PEAR1, since LOXL2 has previously been shown to promote tumor cell proliferation and metastasis and to inhibit tumor cell dormancy [22–24], effects which would be blocked by PEAR1-mediated sequestration of LOXL2. However, loss of LOXL2 only partially recapitulated the effect of PEAR1 on tumor cells, suggesting that the effect of PEAR1 involved additional mechanisms. We therefore searched for other proteins interacting with the extracellular part of PEAR1, which may mediate the effect of PEAR1 on tumor cell dormancy, and identified CTSD.

Our data show that the induction of tumor cell dormancy by endothelial PEAR1 also involves the sequestration of CTSD. Loss of CTSD in tumor cells mimicked the effect of endothelial PEAR1 expression *in vitro* and *in vivo*. This is consistent with previous studies showing that CTSD increased tumor cell proliferation and prevented tumor cells from entering a quiescent state [27, 28, 43–46]. How CTSD promotes tumor growth and inhibits tumor cell dormancy is still poorly understood. In breast cancer cells derived from murine MMTV-PyMT breast cancers, CTSD deficiency has been shown to block tumor cell proliferation and to enhance cellular quiescence by impairing mTORC1 signaling [28]. This effect, which is consistent with our observations, is believed to involve intracellular CTSD. Since CTSD cannot only be secreted by cells but is also efficiently taken up by cells through endocytosis [47–49], binding of extracellular CTSD by PEAR1 may also reduce intracellular levels of CTSD and thereby promote tumor cell quiescence. Consistent with this, we found that CTSD is rapidly taken up by tumor cells, a process that can be blocked by extracellular coapplication of PEAR1, and that CTSD induced activation of AKT and of mTORC1 signaling in tumor cells, resulting in promotion of tumor cell proliferation and inhibition of tumor cell dormancy. This led us to propose a model in which PEAR1 promotes tumor cell dormancy by extracellular binding of CTSD and LOXL2, thereby preventing CTSD and LOXL2 to exert pro-proliferative activity. This suggests that PEAR1 exerts its pro-dormancy effect primarily by binding and thereby blocking factors which inhibit tumor cell dormancy and promote tumor cell proliferation.

Elevated levels of CTSD and LOXL2 were found in various tumors and patient plasma samples, and CTSD as well as LOXL2 have been consistently associated with unfavorable prognosis across various cancers [24, 50–55]. Based on this as well as on multiple evidence indicating that CTSD and LOXL2 have pro-proliferative functions, inhibition of CTSD and LOXL2 have been suggested as a therapeutic approach for various cancers [55–60]. Our data suggest that inhibition of CTSD and LOXL2 would not only have an anti-proliferative effect but may also be an effective therapeutic approach for eradicating residual disseminated tumor cells or maintaining them in a dormant state to avert cancer recurrence. Our identification of PEAR1 as a protein which efficiently binds CTSD and LOXL2 and thereby inhibits their pro-proliferative activity in the context of tumor cell dormancy also suggests PEAR1 as a tool to lower levels of free CTSD and LOXL2. Alternatively, inhibition of PEAR1 could be used as a strategy to reactivate dormant tumor cells to make them susceptible for chemotherapy.

## Materials and methods

### Reagents

Tamoxifen (catalog #T5468) was purchased from Sigma. Human recombinant PEAR1 extracellular part (catalog #4527-PR-050), human Sema6D extracellular part (catalog #2095-S6), human cathepsin D (catalog #1014-AS), the extracellular part of the human EGF receptor (EGFR) (catalog #AVI10493), mouse recombinant Sema6D extracellular part (catalog #7067-S6), human recombinant LOXL2 (catalog #2639-AO-010), and mouse recombinant LOXL2 (catalog #9259-AO) were purchased from R&D systems. DAPI (catalog #D3571), Hoechst 33342 (catalog #H3570), fixable viability dye EFLUOR™ 450 (catalog #65–0863−14), Live/Dead fixable green dye (catalog #L-23101), EZ-Link™ Sulfo-NHS-Biotinylation Kit (catalog #21425), Alexafluor 647-conjugated WGA membrane stain (catalog #W32466), Pierce™ streptavidin magnetic beads (catalog #88817), and Pierce™ Cell Surface Protein Isolation Kit (catalog# 89881) were purchased from ThermoFisher Scientific. Lipofectamine RNAiMAX (catalog #13778150) and lipofectamine 3000 (catalog #L3000015) were purchased from Invitrogen. Human recombinant EGF (catalog #Z03368-1) was purchased from Genscript. 6x-His tagged mouse recombinant CTSD protein (catalog #50127-M08H) was purchased from Sinobiological. 6x-His tagged human recombinant CTSD (catalog #CAD-H52-H3) was purchased from ACROBiosystems. Ni-NTA magnetic agarose beads (catalog #36113) were purchased from Qiagen. Lenti-X Concentrator (catalog #631231) was purchased from Takara Bio. Polybrene (catalog #TR-1003-50UL) was purchased from Merck. IVISbrite D-luciferin potassium salt bioluminescent substrate (catalog #122799) was purchased from Revvity.

### Plasmids

Lentiviral plasmids encoding a scrambled shRNA control (pLV[shRNA]-Puro-U6 > Scramble[shRNA#1]), and an shRNA against *CTSD* (pLV[shRNA]-Puro-U6 > mCtsd[shRNA#1]) were purchased from VectorBuilder. Lentiviral expression plasmids encoding mCherry and firefly luciferase (pLV[Exp]-Hygro- EF1A > mCherry(ns): E2A: Luciferase), mVenus fused with human p27K^−^ (pLV[Exp]-Bsd-EF1A > mVenus: hCDKN1B[NM_004064.5]* and mVenus fused with mouse p27K^−^ (pLV[Exp]-Bsd-EF1A > mVenus: mCdkn1b[NM_009875.4]*) were purchased from VectorBuilder.

### Cell culture

HUVECs (Lonza; catalog #C2519A) were cultured in EGM-2 medium on plates coated with collagen (Corning^®^ Collagen I, rat tail; catalog #356236) at a final concentration of 40 µg/ml. MLECs (Cell biologics; catalog #C57-6011) were cultured in complete mouse endothelial cell medium (Cell biologics; catalog #PB-M1168) on plates coated with 0.1% gelatin (Carl Roth; catalog #0646.1). HUVECs and MLECs were used until passage 7. B16F10 (ATCC; catalog #CRL-6475), MEWO (ATCC; catalog #HTB-65), MDA-MB-231 (ATCC; catalog #CRM-HTB-26), PC3 (ATCC; catalog #CRL-1435), RM1 (ATCC; catalog #CRL-3310), E0771 (CH3 BioSystems; catalog #940001-A), D2.0R and D2A1 cells (kindly provided by Dr. Kent W. Hunter, NIH, Bethesda, USA) and Lenti-X HEK293T cells (Takara-Bio; catalog #632180) were cultured in DMEM (Thermo Fisher Scientific; catalog #11965092) supplemented with 10% fetal bovine serum (FBS) (Thermo Fisher Scientific; catalog #A5670701) and L-glutamine (Thermo Fisher Scientific; catalog #25030081). Prior to experimentation, all cells were tested and confirmed to be free of mycoplasma contamination.

For co-culture of tumor cells and endothelial cells, tumor cells were seeded onto a confluent layer of endothelial cells and cultured at 37 °C at 5% CO_2_. A corresponding culture of tumor cells only was included in each experimental set-up as a control.

### siRNA-mediated knockdown and siRNA screen

Primary endothelial cells or tumor cell lines at 60–70% confluence were transfected with siRNA using Opti-MEM (Thermo Fisher Scientific) and Lipofectamine RNAiMAX (Invitrogen). Lipofectamine RNAiMAX, diluted to a concentration of 6% (v/v) in Opti-MEM, was mixed with siRNA, also diluted in Opti-MEM. Following a 5-min incubation period, the mixture was added to cells at a final siRNA concentration of 28.5 nM. SiRNAs are listed in Suppl. Table 2 (Sigma-Aldrich). Knock-down efficiency was determined by Western blot analysis or by qPCR (QuantStudio 1 Real-Time PCR System; Applied Biosystem). For the siRNA screen, siRNAs directed against RNAs encoding 532 cell surface and secreted proteins which show high expression (RPKM ≥ 100) in HUVECs were used. For each of the 532 selected genes, we used 3 siRNAs which were pooled. 4 independent experiments were performed, and the effect on cell count of MDA-MB-231 cells cocultured with HUVECs was determined (Suppl. Table 1).

### Generation of stable cell lines

To generate tumor cell lines with stable knockdown of CTSD and expression of mCherry-Luciferase: mVenus-p27K^−^, lentiviral vectors transducing shScr, shCTSD, mVenus-p27K^−^, and mCherry-Luciferase were used. At 80% confluence, Lenti-X HEK293T cells were transfected with psPax2 (Addgene) and pCAG-Eco (Addgene) for the generation of mouse-specific lentivirus, while psPax2 (Addgene) and pMD2.g (Addgene) were utilized for producing human lentivirus, in combination with the designated knockdown and overexpression vectors, following the manufacturer’s protocols using Opti-MEM (Thermo Fisher Scientific) and lipofectamine 3000 (Invitrogen). Following a 72-hour incubation, the supernatant was collected and centrifuged at 1500 x g for 3 min at room temperature. Subsequently, the supernatant was filtered through a low protein-binding filter (0.45 μm; Sartorius), and one-third of the volume of Lenti-X-concentrator (Takara) was added and incubated at 4 °C for 24 h. The resulting mixture was centrifugated at 1500 x g for 45 min at 4 °C, and the pellet was resuspended in 1.5 ml of warm media (DMEM + 10% FBS, L-glutamine; Thermo Fisher Scientific) containing 3 µg/ml polybrene (Merck). Following 30 min of incubation at room temperature, 400 µl of the virus solution was combined with 200 µl of tumor cells (1.8 × 10^5^ cells/well), and the cells were cultured in 12-well plates at 37 °C at 5% CO_2_. Once cells reached confluency, they were transferred to 10 cm dishes with DMEM media supplemented with puromycin (10 µg/ml; Sigma). Antibiotic selection was complete when all non-transduced cells had died. Tumor cells transduced with mCherry-Luciferase and mVenus-p27K^−^ were sorted (FACSMelody Cell sorter, BD Biosciences). The efficiency of viral transduction was assessed using quantitative reverse transcription polymerase chain reaction (q-RT-PCR) or Western blot analysis.

### Protein lysate preparation

Cells were washed once with ice-cold PBS, and ice-cold RIPA buffer (25 mM Tris-HCl, pH 7.6, 150 mM NaCl, 1% NP-40, 1% sodium deoxycholate, 0.1% SDS) containing protease and phosphatase inhibitors (Thermo Scientific™; catalog #1861281) was added. Cell lysates were carefully transferred into a pre-cooled Eppendorf tube. After rotating the mixture continuously for 15 min at 4 °C, lysates were centrifuged at 16,000 × g for 20 min at 4 °C, and supernatants were used for further analysis.

### Western blotting

Protein concentrations were determined using the bicinchoninic acid (BCA) assay (Pierce, catalog #23225) using BSA as a standard. Equal amounts of protein were denatured by boiling in Laemmli sample buffer supplemented with 2% β-mercaptoethanol and subsequently separated on 4–12% Tris-glycine SDS-PAGE gels (Novex, catalog #NP0321BOX). Proteins were then transferred onto methanol-activated PVDF membranes (Hybond P 0.2 μm PVDF; Amersham, catalog #GE10600023). To minimize non-specific antibody binding, membranes were blocked with 5% BSA in Tris-buffered saline containing 0.05% Tween-20 (TBST). Primary antibodies (Suppl. Table 3) were added, and membranes were incubated overnight at 4 °C. Next, membranes were washed 5 times with TBST and were then incubated with anti-rabbit IgG HRP-linked secondary antibodies (Cell Signalling, catalog #7074s) or anti-sheep IgG HRP-linked secondary antibodies (R&D Systems, catalog #HAF016). Thereafter, membranes were washed again 5 times with TBST, and bound antibodies were visualized by enhanced chemiluminescence detection using ECL substrate (Pierce, catalog #32106). The densitometric analysis of Western blots was performed using Image J.

### RNA isolation and quantitative RT-PCR

Total RNA was extracted from tumor cells utilizing the Quick-RNA Micro Prep kit (Zymo Research) following the guidelines provided by the manufacturer. The synthesis of complementary DNA (cDNA) was conducted using the ProtoScript II Reverse Transcription kit (New England BioLabs). For quantitative real-time PCR, a Light-Cycler 480 Probe Master System (Roche) was used. Each sample was assessed in triplicates, with GAPDH serving as a control. The relative expression levels were determined employing the ΔΔCt method. Sequences of primers used are listed in Suppl. Table 4.

### Flow cytometry analysis of tumor cell dormancy and survival *in vitro*

Co-cultures of endothelial cells and tumor cells co-expressing mCherry-luciferase and mVenus-p27K^−^ were detached with 0.25% Trypsin-EDTA (Thermo Fisher Scientific) prior to the addition of DMEM maintenance medium. Cell suspensions were centrifuged for 3 min at 500 x g, and the cell pellet was resuspended in 2 ml EGM medium and stained for 30 min at room temperature with anti-CD31 antibodies (Suppl. Table 3). Prior to acquisition of data (FACSMelody Cell sorter, BD Biosciences), cells were filtered through a FACS tube with a strainer cap (Corning). Additionally, DAPI (2 µg/ml) and 10 µl of AccuCount beads (Spherotec Inc.; catalog #ACFP-100-3) were added to the samples to detect dead cells and to account for total number of cells per sample, respectively.

For cell death analysis, both attached and detached cells were collected in tubes and centrifuged for 3 min at 500 x g at room temperature. The supernatant was subsequently discarded, and the pellet was stained with Annexin-V Pacific Blue (Invitrogen; catalog #A35122) following the manufacturer’s guidelines. The reaction mixture (250 µl) was vortexed and incubated for 20 min at room temperature. Then, 250 µl of DMEM maintenance media and 1 µl of the SYTOX AADvanced Dead Cell Stain Kit (Invitrogen; catalog #S10274) were added to each tube prior to flow cytometry analysis using BD FACSMelody Cell Sorter (BD Biosciences).

### Immunostaining of *in vitro* co-culture samples

Cells were washed with phosphate-buffered saline (PBS) and were subsequently fixed with 1% paraformaldehyde (Merck) for 10 min at room temperature. After washing with PBS, the cells were permeabilized with 0.1% triton X-100 (Sigma-Aldrich) for 10 min and were then incubated with 5% BSA (Biomol) for another 10 min. After washing with PBS, cells were incubated overnight at 4 °C with primary antibodies. After washing with PBS, the cells were incubated with Alexafluor 488, 594 and 647-conjugated secondary antibodies (Suppl. Table 3). Cell nuclei were detected with Hoechst 33342 (10 µg/ml; Thermo Fisher Scientific) for 15 min. The cells were then washed in PBS and mounted with Aqua-polymount (Polyscience). Images were taken using Zeiss Airyscan 880 and were analyzed using the ImageJ software.

### Pulldown assay

Recombinant human PEAR1 was immobilized on Ni-NTA magnetic beads (Qiagen) at a concentration of 100 nM, following manufacturer’s guidelines. These beads, along with control Ni-NTA magnetic beads, were separately incubated with 100 nM of either recombinant human CTSD, SEMA6D or LOXL2 in protein binding buffer (50 mM NaH_2_PO_4_, 300 mM NaCl, 20 mM imidazole, pH 8.0). Following an incubation period of one hour at room temperature, beads were placed on a magnetic stand for 2 min, the supernatant was removed, and the magnetic beads were resuspended in 1 ml of wash buffer (50 mM NaH_2_PO_4_, 300 mM NaCl, 20 mM imidazole, 0.005% Tween 20, pH 8.0). The washing procedure was repeated 3 times. The proteins bound to the beads were subsequently eluted using 4x Laemmli buffer at 95 °C for 5 min. Input samples and pulldown eluates were then loaded onto an SDS-PAGE gel for immunoblotting analysis.

### Mass spectrometry

Tumor cells were cultured in DMEM maintenance media (Thermo Fisher Scientific) for 24 h, after which the conditioned media was collected and centrifuged for 5 min at 1000 x g at room temperature. The resulting supernatant was filtered using a low protein binding filter (Sartorius 0.45 µM) and a cocktail of phosphatase and protease inhibitors (Thermo Scientific™) was added. Recombinant human PEAR1 extracellular part (R&D systems) and recombinant human EGFR extracellular part (R&D Systems), both at a concentration of 1000 nM, were immobilized onto Ni-NTA magnetic beads (Qiagen) following the manufacturer’s instructions. In parallel, control NI-NTA beads were prepared, and all beads, including those with immobilized recombinant human PEAR1 and EGFR, were incubated individually with the 24-hour conditioned media from tumor cells for 1 h at RT. The pull-down assay was conducted in accordance with the manufacturer’s guidelines (Qiagen).

Washed beads were mixed with 30 µl digestion buffer (6 M Urea/2 M thiourea; Sigma-Aldrich), reduced with 10 mM DTT for 30 min at room temperature and alkylated with 55 mM IAA for 30 min at room temperature. Each sample was diluted with 60 µl of 0.1 M triethyl ammonium bicarbonate (TEAB; Sigma-Aldrich) and digested with 0.5 µg of trypsin overnight. Digested proteins were desalted using a C18 stage tip. Desalted peptides were measured by LC-MS/MS. Quantitative analyses were performed on an Orbitrap Q-exactive HF mass spectrometry system (Thermo Scientific) coupled to an EASY-nLC capillary nano-chromatography system (Thermo Scientific). Desalted peptides were separated on an in-house-made capillary column (150 mm x 1.7 μm x 75 μm) packed with ReproSil-Pur 120 C18-AQ resin (Dr. Maisch). The mobile phases were (A) 2% acetonitrile, 0.1% fomic acid (B) 90% acetonitrile, 0.1% formic acid. Peptides were separated using a 150 min acetonitrile gradient at room temperature. The mass spectrometer was operated in positive electrospray ionization (ESI) mode, and MS/MS data were collected in data dependent analysis mode with a resolution of 60,000 for precursor mass spectra and 15,000 for tandem mass spectra. Normalized collision energy was set to 28 and exclusion time was set to 30s. Collected data were processed using Maxquant software (2.4.10.0).

### Animal models

All mice were on a C57BL/6J background or were backcrossed at least eight times. Males and females aged between 8 and 12 weeks were used unless otherwise specified. The mice were maintained under a 12-hour light and 12-hour dark cycle with unrestricted access to food and water, and were kept in specific pathogen-free environments. Inducible endothelium-specific *Pear1*- and *Ccl2*-deficient mice (Cdh5-CreER^T2^;*Pear1*^fl/fl^ or Cdh5-CreER^T2^;*Ccl2*^fl/fl^) were generated by crossing Cdh5-CreER^T2^ mice [61] with mice carrying a floxed *Pear1* allele (PEAR1^tm1a(KOMP)Wtsi^ (MRC) originated from ES cell clone EPD0299_2_C05 (KOMP-CSD programme)) or a floxed *Ccl2* allele [62] (The Jackson Laboratory, strain #016849). In some cases, these lines were mated with MMTV-Flp [63], MMTV-PyMT [64], and the dual recombinase responsive fluorescent reporter mice RC::FrePe [65]. Littermates which did not carry the Cre-transgene served as controls for the experiments. Cre-mediated recombination was induced through intraperitoneal injection of tamoxifen (MilliporeSigma, catalog #T5648), which was dissolved in Miglyol 812 at a dosage of 1 mg per mouse per day, administered over a period of 5 consecutive days. Throughout the experiments, mice were housed in cages where their identities were obscured by cage cards, ensuring that the procedures were conducted in a blinded manner.

### Lung perfusion

Mice were euthanized using CO_2_ asphyxiation. Thereafter, the abdominal aorta was opened, and lungs were perfused by injecting a total of 10 ml of PBS into the right ventricle using a 21G needle until the lungs turned white. Subsequently, the perfused lungs were removed from the body and were either immersed in IVISbrite D-Luciferin potassium salt bioluminescent substrate (15 mg/ml; Revvity) for IVIS imaging, transferred directly to DMEM (Thermo Fisher Scientific) for digestion, or were placed into 4% PFA (Sigma) for cryosectioning.

### Lung digestion

The harvested perfused lung tissue was cut and then placed into a gentleMACS C tube (Miltenyi Biotec) containing 5 ml of digestion medium consisting of DMEM with 2 mg/ml of Collagenase type II (Worthington) and 50 mg/ml of DNAse I (ApplyChem). The mixture was processed using the gentleMACS octo dissociator program (Miltenyi Biotec) for lung digestion at 37 °C for 30 min. Before being filtered through a 70 μm sieve into a tube with 10 ml of complete media, the digestion medium was carefully passed 3 times through a 20G needle. After centrifugation at 500 x g for 10 min, the supernatant was removed, and the resultant cell pellet was resuspended in 2 ml of 0.5% BSA in PBS, pH 8.0.

### Endothelial cell sorting

The digested lung tissue was resuspended in 2 ml of 0.5% BSA and then loaded into the AutoMACS NEO Separator from Miltenyi Biotec, using anti-CD31 mouse MicroBeads according to the manufacturer’s specifications. The CD31-positive fraction was subsequently stained with anti-CD31 Pe-Cy7 mouse antibody and anti-CD45 FITC mouse antibody (BD Biosciences) for 20 min at room temperature. Prior to flow cytometry analysis using the BD FACS Melody Cell Sorter (BD Biosciences), DAPI (2 µg/ml; Invitrogen) was added, and the cells were filtered. Mouse lung endothelial cells (MLECs) were stained by an anti-mouse CD31 antibody conjugated with Pe-Cy7 for visualization (Suppl. Table 3). Tumor cells expressed mCherry while immune cells were identified by an anti-mouse CD45 antibody conjugated with FITC to differentiate them from MLECs. The sorted MLECs were then collected for further analysis using Western Blot and qPCR techniques.

### Intravenous injection of tumor cells

Mice were subjected to deep anesthesia using 2% isoflurane (Merck). To facilitate vein dilation, the tail of each mouse was warmed using an infrared red lamp. A total of 100 µl containing 2.5 × 10^5^ B16F10/E0771/RM1 tumor cells was injected into the tail vein for the evaluation of extravasation and tumor cell dormancy. Lung metastases were assessed using a stereo-microscope (Zeiss) to identify and quantify metastatic lesions and nodules. Bioluminescent imaging of the lungs was conducted using IVIS (PerkinElmer) to evaluate the total metastatic load. Flow cytometry was used for tumor dormancy and cell death analysis. Additionally, immunohistochemistry was carried out to examine the extravasation and dormancy of tumor cells, both individually and in clusters, including micrometastases and macrometastases. Tumor cells co-expressing mCherry, luciferase, and mVenusp27K^−^ were used for assessing the effect of suppressed CTSD expression on tumor cell dormancy. For assessing tumor cell dormancy in EC-Pear1-KO, EC-Ccl2-KO, and wildtype controls, tumor cells were additionally loaded with CellTrace Yellow tracker dye prior to injection. For the extravasation and cell death analysis experiment *in vivo*, tumor cells co-expressing GFP and luciferase were loaded with carboxyfluorescein succinimidyl ester (CFSE) prior to injection.

### Primary tumor resection model

Mice were subjected to deep anesthesia with 2% isoflurane (Merck) prior to subcutaneous injection of 2.5 × 10^5^ B16F10 cells or orthotopic implantation of E0771 tumor cell mixture (50 µl Matrigel (Corning) + 50 µl of 5.0 × 10^6^ E0771 cells/ml) in the mammary fat pad. Tumor growth was assessed one week post-injection and was monitored for a duration of at least 2–3 weeks before the primary tumor was resected. Six weeks post-resection, lungs were harvested and examined using IVIS (Bio-Rad). Tumor dormancy was evaluated through flow cytometric analysis of the mVenus-p27K^−^ reporter, as well as through immunohistochemistry to detect the presence of solitary cells, micrometastases (2–8 cells), and macrometastases (> 8 cells).

### PyMT genetic mouse model

After 6 weeks of age, mice were subjected to deep anesthesia using 2% isoflurane (Merck) to assess the total tumor volume using a caliper. Upon reaching a tumor volume of 1.7 cm³, PyMT mice were euthanized, and blood samples were obtained for the analysis of circulating tumor cells (CTCs). Epifluorescent imaging of the lungs was performed, followed by lung tissue processing for digestion to assess tumor cell dormancy. This was achieved through flow cytometry analysis of cell suspensions that were stained with Ki67.

### IVIS imaging

For live imaging procedures, mice were kept under deep anesthesia using 2% isoflurane (Merck). To monitor the progression of metastatic disease of luciferase-expressing tumor cells, mice received intraperitoneal injections of 150 mg/kg of D-luciferin in 100 µl PBS and were subsequently imaged with the *in vivo *imaging system (IVIS) (PerkinElmer, catalog #128201) using luminescence detection. For ex vivo imaging, lungs were incubated in an Eppendorf tube containing 500 µl of D-luciferin (15 mg/ml) for 10 min at room temperature. Lungs were washed with PBS before being positioned in an imaging plate for the visualization of lung metastases via luminescence. For PyMT animals, the perfused lungs were immediately analyzed ex vivo using epifluorescence to visualize the mCherry signal.

### Flow cytometry analysis of tumor cell dormancy and cell death *in vivo*

Digested lung tissue was resuspended in 200 µl of mouse microbead cocktail solution (50 µl anti-TER119, 20 µl anti-CD31, 20 µl anti-CD45 mouse microbeads in 0.5% BSA in PBS) and incubated for 30 min at 4 °C. Following incubation, 2 ml of 0.5% BSA in PBS was added and the resulting mixture was filtered through a 35 μm filter before being loaded into the AutoMACS NEO Separator from Miltenyi Biotec using the DEPLETE program. These MicroBeads can capture endothelial cells (CD31), immune cells (CD45), and erythrocytes (TER119) in cell suspensions, allowing them to be eluted. Conversely, cells that do not attach to the MicroBeads, such as tumor cells, were subsequently used for flow cytometric analysis in various metastasis mouse models.

For dormancy analysis using the resection and the intravenous injection model of lung metastasis, tumor cells coexpressing mCherry-luciferase and mVnenus-p27K^−^ were used. Prior to analysis using the BD FACSMelody Cell Sorter (BD Biosciences), DAPI (2 µg/ml) was added.

For the Ki67 analysis of mCherry-positive breast cancer cells derived from MMTV-PyMT/mCherry mice, the pre-enriched tumor fraction underwent centrifugation at 500 x g for 10 min at 4 °C. The resulting cell pellet was resuspended, and cells were incubated with Hoechst 33342 (5 µg/mL) and Live/Dead™ fixable green dye (1:1000, ThermoFisher Scientific, catalog #L-23101) for 45 min at 37 °C. After another round of centrifugation at 500 x g for 10 min at 4 °C, cells were fixed with 1% paraformaldehyde (PFA) for 10 min at 4 °C. Subsequently, the samples were centrifuged at 1000 x g for 5 min at 4 °C, washed with PBS, and then incubated in a 1x permeabilization buffer (Invitrogen; catalog #00–8333−56) for 10 min at room temperature. Following another centrifugation at 1000 x g for 5 min at 4 °C, cells were washed with PBS again and incubated in PBS containing 5% BSA for 30 min at room temperature to reduce unspecific binding of antibodies. Cells were then incubated overnight at 4 °C in a staining solution that included anti-mCherry and Pe-Cy7-conjugated Rat IgG2a anti-mouse Ki67 antibodies, along with a separate isotype control stained with the mCherry antibody but paired with Pe-Cy7-conjugated Rat IgG2a (Suppl. Table 3). After washing with PBS, the pellet was resuspended, and cells were stained with an Alexafluor 594 anti-chicken secondary antibody (Suppl. Table 3) for 1 h at room temperature. Following two additional washes, cells were resuspended in PBS prior to analysis using the BD FACSMelody Cell Sorter (BD Biosciences).

For cell death analysis in the intravenous injection model of lung metastasis, tumor cells expressing GFP were used. The enriched tumor fraction was centrifuged at 500 x g for 10 min at 4 °C. The cells were resuspended and were stained with Pe-Cy7 anti-mouse CD45 and CD31 antibodies (Suppl. Table 3) along with Annexin-V Pacific Blue (Invitrogen; catalog #A35122) following the manufacturer’s guidelines. The reaction mixture (250 µl) was vortexed and incubated for 20 min at room temperature. Then, 250 µl of DMEM media and 1 µl of the SYTOX AADvanced Dead Cell Stain Kit (Invitrogen; catalog #S10274) were added to each tube prior to flow cytometry analysis using a BD FACSMelody Cell Sorter (BD Biosciences).

### Immunohistochemistry of mouse lungs for metastasis and extravasation

Freshly isolated lungs were fixed in 4% PFA overnight at 4 °C and were then placed into 15 ml tubes containing 10%, 20%, 30% sucrose for 24 h each. The tissue was cryopreserved in Tissuetek (Sakura Finetek, Staufen, Germany) and was sectioned at a thickness of 20 μm. Sections were mounted on Superfrost™ Plus adhesion microscope slides (catalaog #J1800AMNZ; Epredia), and slides were incubated in PBS containing 5% BSA and 0.2% Triton X-100 for 30 min, followed by an overnight incubation at 4 °C with the relevant staining solutions designed for evaluating either tumor cell dormancy or tumor extravasation.

For assessing tumor cell dormancy, the sections on slides were stained with antibodies against Ki67, mCherry, and Venus (Suppl. Table 3). For assessing tumor cell extravasation, the slides were stained with antibodies against GFP and CD31 (Suppl. Table 3). After overnight incubation with the primary antibody, slides were washed with PBS and incubated with the corresponding secondary antibodies. For assessing tumor cell dormancy, the slides were incubated with Alexafluor 488, 594 and 647-conjugated secondary antibodies (Suppl. Table 3) for 2 h at room temperature. For assessing tumor cell extravasation, the slides were incubated with Alexafluor 488 and 647-conjugated secondary antibodies (Suppl. Table 3) for 2 h at room temperature. Following incubation, the slides were then washed with PBS and nuclei were stained with Hoechst 33342 (5 µg/ml) for 15 min at room temperature. The slides were then washed in PBS and mounted with Aqua-polymount (Polyscience). Images were taken using Zeiss Airyscan 880 and were analyzed using the ImageJ software.

### Detection of circulating tumor cells in PyMT mice

PyMT mice in which tumor cells express mCherry were euthanized through CO_2_ asphyxiation and intracardiacally obtained blood samples were collected in EDTA-coated tubes and placed on ice. To separate the plasma, the blood was centrifuged for 10 min at 1000 x g at 4 °C. The plasma was removed, and the cell pellet was resuspended in 1 ml of PBS. Red blood cells were depleted using an Acrodisc WBC syringe filter (Pall Corporation; catalog #AP-4952) according to the manufacturer’s instructions. The remaining cell suspension was then centrifuged for 5 min at 500 x g at 4 °C. After discarding the supernatant, the cells were resuspended and stained with anti-mouse CD45 FITC antibody for 20 min at room temperature in the dark (Suppl. Table 3). Prior to flow cytometry analysis (FACSMelody cell sorter, BD Biosciences), DAPI (2 µg/ml) and 10 µl of AccuCount beads (Spherotec Inc., catalog #ACFP-100-3) were added per 1 ml of whole blood to detect dead cells and to account for total number of cells per sample.

### Analysis of CTSD binding to cells

MDA-MB-231, E0771, and B16F10 cancer cell lines were cultured until reaching 60% confluence. Following an overnight period of starvation in serum-free medium, the cells were detached using CellStripper (Corning; catalog #25–056-CI). Cell suspensions were prepared at a concentration of 1 × 10^6^ cells/ml and centrifuged for 3 min at 500 x g at 4 °C. The resulting cell pellet was then resuspended in HBSS binding buffer containing varying concentrations of 6x-His tagged CTSD along with 1 µl of fixable viability dye eFluor™ 450 (ThermoFisher Scientific) per ml of cell suspension and incubated for 1 h at 4 °C. After washing the cells twice with HBSS, cells were fixed in 1% PFA for 10 min at 4 °C. Following centrifugation at 1000 x g at 4 °C for 3 min, cells were washed once with HBSS and then incubated with a PE-conjugated antibody specific to 6x-His (Suppl. Table 3) for 30 min at RT. After another centrifugation at 1000 x g at 4 °C for 3 min, cells were washed twice with HBSS before data acquisition was performed using a FACSMelody Cell Sorter (BD Biosciences).

Immunocytochemical staining was performed on cells treated with CTSD (100 nM), either alone or together with the recombinant extracellular part of PEAR1 conjugated to streptavidin beads. Since both PEAR1 and CTSD are labeled with 6x His, we biotinylated PEAR1 using the EZ-Link™ Sulfo-NHS-Biotinylation Kit (ThermoFisher Scientific). This was followed by the conjugation of the biotinylated PEAR1 to Pierce™ streptavidin magnetic beads (ThermoFisher Scientific) in accordance with the manufacturer’s guidelines. MDA-MB-231, E0771, and B16F10 cancer cell lines were cultured on µ-slides (Ibidi; catalog #81816) until they reached 60% confluence. After an overnight starvation period in serum-free media, cells were treated with either recombinant human or mouse CTSD for 1 h at 37 °C. The slides were subsequently placed on ice and washed twice with HBSS before being fixed with 1% PFA for 10 min at 4 °C. Following washing with HBSS, the cells were incubated with an Alexafluor 647-conjugated WGA membrane stain (ThermoFisher Scientific) for 30 min at room temperature. The cells were washed with HBSS before being incubated with 0.1% Triton-X (Sigma; catalog #T8787) for 10 min at room temperature. After washing with PBS, the cells were incubated overnight at 4 °C with PE-conjugated antibody directed against 6x-His. After washing with PBS, the cells were incubated with an Alexafluor 594-conjugated secondary antibody (Suppl. Table 3) for 90 min at room temperature. Cell nuclei were stained with Hoechst 33342 (10 µg/ml) for 15 min. The cells were then washed with HBSS (Thermo Fisher Scientific) and mounted using Aqua-polymount (Polyscience). Imaging was performed using a Leica SP8 confocal microscope, and the images were analyzed with ImageJ software.

### Analysis of CTSD endocytosis and CTSD-induced cellular effects

To determine whether CTSD added to cells remains on the surface of the plasma membrane or can be taken up by cells, MDA-MB-231, E0771, and B16F10 cancer cell lines were cultured until reaching 60% confluence. After an overnight period of starvation in serum-free medium, the cells were incubated without or with 300 nM recombinant 6x-His tagged CTSD for one hour at 37 °C. The cells were then subjected to a 10-minute incubation at 4 °C followed by two washes with ice-cold PBS. Biotinylation of cell surface proteins was subsequently conducted using the Pierce™ Cell Surface Protein Isolation Kit. Briefly, the cells were treated with EZ-Link-NHS-SS-Biotin (0.25 mg/ml) for 30 min at 4 °C, and a quenching solution was added before washing the cells five times with Tris-buffered saline (TBS; 136.8 mM NaCl, 2.682 mM KCl, 100 mM Tris, pH 7.4) to remove excess biotin. Following this, the cells were lysed, and biotinylated surface proteins were isolated via Pierce™ streptavidin magnetic beads (Thermo Fisher Scientific). The non-biotinylated fraction, containing intracellular proteins, was retained for further analysis. Protein concentrations for both fractions were assessed as described above, and 6 µg of both biotinylated (plasma membrane) and non-biotinylated (intracellular) fractions were subjected to SDS-PAGE and analyzed by immunoblotting using antibodies specific to 6x-His, Notch2, Rab5, and GAPDH (Suppl. Table 3).

For assessing the functional consequence of CTSD endocytosis and its sequestration by PEAR1, immunoblotting was performed on samples collected 24 h after treatment to assess the phosphorylation levels of mTOR, AKT, and ERK1/2 (Suppl. Table 3). Additionally, cell counting and p27 analysis were conducted following four days of daily treatment, adhering to the previously outlined methodology.

### Statistical analysis

Analysis was carried out utilizing GraphPad Prism software version 9 (GraphPad Software Inc.). The data are expressed as the mean ± S.E.M. or S.D. as indicated in the figure legends, and “n” indicates the number of independent experiments. Data were tested for normality using the Shapiro-Wilk test. For comparing two groups of Gaussian distributed data, either an unpaired two-tailed Student’s t-test (parametric), unpaired t-test with Welch’s correction (unequal variance) or the Mann-Whitney U test (non-parametric) were applied. When analyzing differences among multiple groups, a 1-way ANOVA with Tukey’s post hoc test was employed. For assessments involving multiple groups across different time points, a 2-way ANOVA followed by Bonferroni’s post hoc test was conducted. For non-parametric multiple group comparisons, the Kruskal-Wallis test was performed, followed by Dunn’s multiple comparison. For the survival curve analyses, two-sided log-rank test was performed. A p-value of less than 0.05 was considered to indicate statistical significance.

## Supplementary Information

Supplementary Material 1.

Supplementary Material 2.

Supplementary Material 3.

## Data Availability

The reported mass spectrometry data are available via ProteomeXchange (PXD061825).

## References

[1] Ghajar CM. Metastasis prevention by targeting the dormant niche. Nat Rev Cancer. 2015;15:238–47. 10.1038/nrc3910. 25801619 10.1038/nrc3910 PMC4842412

[2] Giancotti FG. Mechanisms governing metastatic dormancy and reactivation. Cell. 2013;155:750–64. 10.1016/j.cell.2013.10.029. 24209616 10.1016/j.cell.2013.10.029 PMC4354734

[3] Aguirre-Ghiso JA. Models, mechanisms and clinical evidence for cancer dormancy. Nat Rev Cancer. 2007;7:834–46. 10.1038/nrc2256. 17957189 10.1038/nrc2256 PMC2519109

[4] Klein CA. Cancer progression and the invisible phase of metastatic colonization. Nat Rev Cancer. 2020;20:681–94. 10.1038/s41568-020-00300-6. 33024261 10.1038/s41568-020-00300-6

[5] Heidrich I, Deitert B, Werner S, Pantel K. Liquid biopsy for monitoring of tumor dormancy and early detection of disease recurrence in solid tumors. Cancer Metastasis Rev. 2023;42:161–82. 10.1007/s10555-022-10075-x. 36607507 10.1007/s10555-022-10075-x PMC10014694

[6] Sosa MS, Bragado P, Aguirre-Ghiso JA. Mechanisms of disseminated cancer cell dormancy: an awakening field. Nat Rev Cancer. 2014;14:611–22. 10.1038/nrc3793. 25118602 10.1038/nrc3793 PMC4230700

[7] Weston WA, Barr AR. A cell cycle centric view of tumour dormancy. Br J Cancer. 2023;129:1535–45. 10.1038/s41416-023-02401-z. 37608096 10.1038/s41416-023-02401-z PMC10645753

[8] Risson E, Nobre AR, Maguer-Satta V, Aguirre-Ghiso JA. The current paradigm and challenges ahead for the dormancy of disseminated tumor cells. Nat Cancer. 2020;1:672–80. 10.1038/s43018-020-0088-5. 33681821 10.1038/s43018-020-0088-5 PMC7929485

[9] Phan TG, Croucher PI. The dormant cancer cell life cycle. Nat Rev Cancer. 2020;20:398–411. 10.1038/s41568-020-0263-0. 32488200 10.1038/s41568-020-0263-0

[10] Bushnell GG, Deshmukh AP, den Hollander P, Luo M, Soundararajan R, Jia D, et al. Breast cancer dormancy: need for clinically relevant models to address current gaps in knowledge. NPJ Breast Cancer. 2021;7:66. 10.1038/s41523-021-00269-x. 34050189 10.1038/s41523-021-00269-x PMC8163741

[11] Damen MPF, van Rheenen J, Scheele C. Targeting dormant tumor cells to prevent cancer recurrence. FEBS J. 2021;288:6286–303. 10.1111/febs.15626. 33190412 10.1111/febs.15626

[12] Goddard ET, Bozic I, Riddell SR, Ghajar CM. Dormant tumour cells, their niches and the influence of immunity. Nat Cell Biol. 2018;20:1240–9. 10.1038/s41556-018-0214-0. 30361702 10.1038/s41556-018-0214-0

[13] Ghajar CM, Peinado H, Mori H, Matei IR, Evason KJ, Brazier H, et al. The perivascular niche regulates breast tumour dormancy. Nat Cell Biol. 2013;15:807–17. 10.1038/ncb2767. 23728425 10.1038/ncb2767 PMC3826912

[14] Kienast Y, von Baumgarten L, Fuhrmann M, Klinkert WE, Goldbrunner R, Herms J, Winkler F. Real-time imaging reveals the single steps of brain metastasis formation. Nat Med. 2010;16:116–22. 10.1038/nm.2072. 20023634 10.1038/nm.2072

[15] Nowosad A, Marine JC, Karras P. Perivascular niches: critical hubs in cancer evolution. Trends Cancer. 2023;9:897–910. 10.1016/j.trecan.2023.06.010. 37453870 10.1016/j.trecan.2023.06.010

[16] Liu R, Zhao Y, Su S, Kwabil A, Njoku PC, Yu H, et al. Unveiling cancer dormancy: intrinsic mechanisms and extrinsic forces. Cancer Lett. 2024;591:216899. 10.1016/j.canlet.2024.216899. 38649107 10.1016/j.canlet.2024.216899 PMC13001072

[17] Oki T, Nishimura K, Kitaura J, Togami K, Maehara A, Izawa K, et al. A novel cell-cycle-indicator, mVenus-p27K-, identifies quiescent cells and visualizes G0-G1 transition. Sci Rep. 2014;4(1):4012. 10.1038/srep04012. 24500246 10.1038/srep04012 PMC3915272

[18] Singh A, Veeriah V, Xi P, Labella R, Chen J, Romeo SG, et al. Angiocrine signals regulate quiescence and therapy resistance in bone metastasis. JCI Insight. 2019;4:e125679. 10.1172/jci.insight.125679. 31292293 10.1172/jci.insight.125679 PMC6629249

[19] Elenbaas JS, Pudupakkam U, Ashworth KJ, Kang CJ, Patel V, Santana K, et al. SVEP1 is an endogenous ligand for the orphan receptor PEAR1. Nat Commun. 2023;14:850. 10.1038/s41467-023-36486-0. 36792666 10.1038/s41467-023-36486-0 PMC9932102

[20] Sun Y, Vandenbriele C, Kauskot A, Verhamme P, Hoylaerts MF, Wright GJ. A human platelet receptor protein microarray identifies the high affinity Immunoglobulin E receptor subunit alpha (FcepsilonR1alpha) as an activating platelet endothelium aggregation receptor 1 (PEAR1) ligand. Mol Cell Proteom. 2015;14:1265–74. 10.1074/mcp.M114.046946. 10.1074/mcp.M114.046946 PMC4424398 25713122

[21] Shen Y, Yan J, Li L, Sun H, Zhang L, Li G, et al. LOXL2-induced PEAR1 Ser891 phosphorylation suppresses CD44 degradation and promotes triple-negative breast cancer metastasis. J Clin Invest. 2024;134:e177357. 10.1172/JCI177357. 39145451 10.1172/JCI177357 PMC11324313

[22] Weidenfeld K, Schif-Zuck S, Abu-Tayeh H, Kang K, Kessler O, Weissmann M, et al. Dormant tumor cells expressing LOXL2 acquire a stem-like phenotype mediating their transition to proliferative growth. Oncotarget. 2016;7:71362–77. 10.18632/oncotarget.12109. 27655685 10.18632/oncotarget.12109 PMC5342084

[23] Liburkin-Dan T, Toledano S, Neufeld G. Lysyl oxidase family enzymes and their role in tumor progression. Int J Mol Sci. 2022;23:6249. 10.3390/ijms23116249. 35682926 10.3390/ijms23116249 PMC9181702

[24] Cano A, Eraso P, Mazon MJ, Portillo F. LOXL2 in cancer: a two-decade perspective. Int J Mol Sci. 2023. 10.3390/ijms241814405. 37762708 10.3390/ijms241814405 PMC10532419

[25] Kim BR, Dong SM, Seo SH, Lee JH, Lee JM, Lee SH, et al. Lysyl oxidase-like 2 (LOXL2) controls tumor-associated cell proliferation through the interaction with MARCKSL1. Cell Signal. 2014;26:1765–73. 10.1016/j.cellsig.2014.05.010. 24863880 10.1016/j.cellsig.2014.05.010

[26] Fan Z, Liu Y, Liu X, Nian W, Huang X, Yang Q, et al. Phosphorylation of AKT by Lysyl oxidase-like 2 activates the PI3K/AKT signaling pathway to promote proliferation, invasion and metastasis in esophageal squamous carcinoma. Clin Transl Oncol. 2023;25:2487–98. 10.1007/s12094-023-03133-5. 36995521 10.1007/s12094-023-03133-5 PMC10293450

[27] Benes P, Vetvicka V, Fusek M. Cathepsin d–many functions of one aspartic protease. Crit Rev Oncol Hematol. 2008;68:12–28. 10.1016/j.critrevonc.2008.02.008. 18396408 10.1016/j.critrevonc.2008.02.008 PMC2635020

[28] Ketterer S, Mitschke J, Ketscher A, Schlimpert M, Reichardt W, Baeuerle N, et al. Cathepsin D deficiency in mammary epithelium transiently stalls breast cancer by interference with mTORC1 signaling. Nat Commun. 2020;11:5133. 10.1038/s41467-020-18935-2. 33046706 10.1038/s41467-020-18935-2 PMC7552405

[29] Morris VL, Tuck AB, Wilson SM, Percy D, Chambers AF. Tumor progression and metastasis in murine D2 hyperplastic alveolar nodule mammary tumor cell lines. Clin Exp Metastasis. 1993;11:103–12. 10.1007/BF00880071. 8422701 10.1007/BF00880071

[30] Kusumbe AP. Vascular niches for disseminated tumour cells in bone. J Bone Oncol. 2016;5:112–6. 10.1016/j.jbo.2016.04.003. 27761369 10.1016/j.jbo.2016.04.003 PMC5063228

[31] D’Antonio C, Liguori GL. Dormancy and awakening of cancer cells: the extracellular vesicle-mediated cross-talk between Dr. Jekill and Mr. Hyde. Front Immunol. 2024;15:1441914. 10.3389/fimmu.2024.1441914. 39301024 10.3389/fimmu.2024.1441914 PMC11410588

[32] Hernandez-Barranco A, Nogues L, Peinado H. Could extracellular vesicles contribute to generation or awakening of sleepy. Metastatic niches? Front Cell Dev Biol. 2021;9:625221. 10.3389/fcell.2021.625221. 33738282 10.3389/fcell.2021.625221 PMC7960773

[33] Jakab M, Lee KH, Uvarovskii A, Ovchinnikova S, Kulkarni SR, Jakab S, et al. Lung endothelium exploits susceptible tumor cell states to instruct metastatic latency. Nat Cancer. 2024;5:716–30. 10.1038/s43018-023-00716-7. 38308117 10.1038/s43018-023-00716-7 PMC11136671

[34] Yumoto K, Eber MR, Wang J, Cackowski FC, Decker AM, Lee E, et al. Axl is required for TGF-β2-induced dormancy of prostate cancer cells in the bone marrow. Sci Rep. 2016;6:36520. 10.1038/srep36520. 27819283 10.1038/srep36520 PMC5098246

[35] Kobayashi A, Okuda H, Xing F, Pandey PR, Watabe M, Hirota S, et al. Bone morphogenetic protein 7 in dormancy and metastasis of prostate cancer stem-like cells in bone. J Exp Med. 2011;208:2641–55. 10.1084/jem.20110840. 22124112 10.1084/jem.20110840 PMC3244043

[36] Di Martino JS, Nobre AR, Mondal C, Taha I, Farias EF, Fertig EJ, et al. A tumor-derived type III collagen-rich ECM niche regulates tumor cell dormancy. Nat Cancer. 2022;3:90–107. 10.1038/s43018-021-00291-9. 35121989 10.1038/s43018-021-00291-9 PMC8818089

[37] Dai J, Cimino PJ, Gouin KH 3rd, Grzelak CA, Barrett A, Lim AR, Long A, Weaver S, Saldin LT, Uzamere A, et al. Astrocytic laminin-211 drives disseminated breast tumor cell dormancy in brain. Nat Cancer. 2022;3:25–42. 10.1038/s43018-021-00297-3. 35121993 10.1038/s43018-021-00297-3 PMC9469899

[38] Mukherjee A, Bravo-Cordero JJ. Regulation of dormancy during tumor dissemination: the role of the ECM. Cancer Metastasis Rev. 2023;42:99–112. 10.1007/s10555-023-10094-2. 36802311 10.1007/s10555-023-10094-2 PMC10027413

[39] Nanda N, Bao M, Lin H, Clauser K, Komuves L, Quertermous T, et al. Platelet endothelial aggregation receptor 1 (PEAR1), a novel epidermal growth factor repeat-containing transmembrane receptor, participates in platelet contact-induced activation. J Biol Chem. 2005;280:24680–9. 10.1074/jbc.M413411200. 15851471 10.1074/jbc.M413411200

[40] Kauskot A, Di Michele M, Loyen S, Freson K, Verhamme P, Hoylaerts MF. A novel mechanism of sustained platelet alphaIIbbeta3 activation via PEAR1. Blood. 2012;119:4056–65. 10.1182/blood-2011-11-392787. 22371881 10.1182/blood-2011-11-392787

[41] Kardeby C, Evans A, Campos J, Al-Wahaibi AM, Smith CW, Slater A, et al. Heparin and heparin proteoglycan-mimetics activate platelets via PEAR1 and PI3Kβ. J Thromb Haemost. 2023;21(1):101–16. 10.1016/j.jtha.2022.10.008. 36695374 10.1016/j.jtha.2022.10.008

[42] Bottomly D, Long N, Schultz AR, Kurtz SE, Tognon CE, Johnson K, Abel M, Agarwal A, Avaylon S, Benton E, et al. Integrative analysis of drug response and clinical outcome in acute myeloid leukemia. Cancer Cell. 2022;40:850–e864859. 10.1016/j.ccell.2022.07.002. 35868306 10.1016/j.ccell.2022.07.002 PMC9378589

[43] Westley B, Rochefort H. A secreted glycoprotein induced by Estrogen in human breast cancer cell lines. Cell. 1980;20:353–62. 10.1016/0092-8674(80)90621-2. 7388945 10.1016/0092-8674(80)90621-2

[44] Olson OC, Joyce JA. Cysteine cathepsin proteases: regulators of cancer progression and therapeutic response. Nat Rev Cancer. 2015;15:712–29. 10.1038/nrc4027. 26597527 10.1038/nrc4027

[45] Masson O, Bach AS, Derocq D, Prebois C, Laurent-Matha V, Pattingre S, et al. Pathophysiological functions of cathepsin D: targeting its catalytic activity versus its protein binding activity? Biochimie. 2010;92:1635–43. 10.1016/j.biochi.2010.05.009. 20493920 10.1016/j.biochi.2010.05.009

[46] Capony F, Rougeot C, Montcourrier P, Cavailles V, Salazar G, Rochefort H. Increased secretion, altered processing, and glycosylation of pro-cathepsin d in human mammary cancer cells. Cancer Res. 1989;49:3904–9. 2736531

[47] Derocq D, Prebois C, Beaujouin M, Laurent-Matha V, Pattingre S, Smith GK, et al. Cathepsin D is partly endocytosed by the LRP1 receptor and inhibits LRP1-regulated intramembrane proteolysis. Oncogene. 2012;31:3202–12. 10.1038/onc.2011.501. 22081071 10.1038/onc.2011.501 PMC3579766

[48] Laurent-Matha V, Farnoud MR, Lucas A, Rougeot C, Garcia M, Rochefort H. Endocytosis of pro-cathepsin D into breast cancer cells is mostly independent of mannose-6-phosphate receptors. J Cell Sci 111. 1998; (Pt. 17):2539–49. 10.1242/jcs.111.17.2539. 10.1242/jcs.111.17.2539 9701553

[49] Prieto Huarcaya S, Drobny A, Marques ARA, Di Spiezio A, Dobert JP, Balta D, Werner C, Rizo T, Gallwitz L, Bub S, et al. Recombinant pro-CTSD (cathepsin D) enhances SNCA/alpha-Synuclein degradation in alpha-Synucleinopathy models. Autophagy. 2022;18:1127–51. 10.1080/15548627.2022.2045534. 35287553 10.1080/15548627.2022.2045534 PMC9196656

[50] Tan G, Liu Q, Tang X, Kang T, Li Y, Lu J, et al. Diagnostic values of serum cathepsin B and D in patients with nasopharyngeal carcinoma. BMC Cancer. 2016;16:241. 10.1186/s12885-016-2283-4. 26995190 10.1186/s12885-016-2283-4 PMC4799840

[51] Losch A, Tempfer C, Kohlberger P, Joura EA, Denk M, Zajic B, et al. Prognostic value of cathepsin D expression and association with histomorphological subtypes in breast cancer. Br J Cancer. 1998;78:205–9. 10.1038/bjc.1998.465. 9683294 10.1038/bjc.1998.465 PMC2062888

[52] Kang J, Yu Y, Jeong S, Lee H, Heo HJ, Park JJ, et al. Prognostic role of high cathepsin D expression in breast cancer: a systematic review and meta-analysis. Ther Adv Med Oncol. 2020;12:1758835920927838. 10.1177/1758835920927838. 32550865 10.1177/1758835920927838 PMC7281710

[53] Foekens JA, Look MP, Bolt-de Vries J, Meijer-van Gelder ME, van Putten WL, Klijn JG. Cathepsin-D in primary breast cancer: prognostic evaluation involving 2810 patients. Br J Cancer. 1999;79:300–7. 10.1038/sj.bjc.6690048. 9888472 10.1038/sj.bjc.6990048 PMC2362199

[54] Abbott DE, Margaryan NV, Jeruss JS, Khan S, Kaklamani V, Winchester DJ, et al. Reevaluating cathepsin D as a biomarker for breast cancer: serum activity levels versus histopathology. Cancer Biol Ther. 2010;9:23–30. 10.4161/cbt.9.1.10378. 19923884 10.4161/cbt.9.1.10378 PMC3187721

[55] Wen B, Xu LY, Li EM. LOXL2 in cancer: regulation, downstream effectors and novel roles. Biochimica et Biophysica Acta (BBA). 2020;1874:188435. 10.1016/j.bbcan.2020.188435. 10.1016/j.bbcan.2020.188435 32976981

[56] Dubey V, Luqman S. Cathepsin D as a promising target for the discovery of novel anticancer agents. Curr Cancer Drug Targets. 2017;17:404–22. 10.2174/1568009616666161229145115. 28215160 10.2174/1568009616666161229145115

[57] Zheng W, Chen Q, Wang C, Yao D, Zhu L, Pan Y, et al. Inhibition of cathepsin D (CTSD) enhances radiosensitivity of glioblastoma cells by attenuating autophagy. Mol Carcinog. 2020;59:651–60. 10.1002/mc.23194. 32253787 10.1002/mc.23194

[58] Seo SU, Woo SM, Im SS, Jang Y, Han E, Kim SH, et al. Cathepsin d as a potential therapeutic target to enhance anticancer drug-induced apoptosis via RNF183-mediated destabilization of Bcl-xL in cancer cells. Cell Death Dis. 2022;13:115. 10.1038/s41419-022-04581-7. 35121737 10.1038/s41419-022-04581-7 PMC8816936

[59] Peng T, Lin S, Meng Y, Gao P, Wu P, Zhi W, et al. LOXL2 small molecule inhibitor restrains malignant transformation of cervical cancer cells by repressing LOXL2-induced epithelial-mesenchymal transition (EMT). Cell Cycle. 2022;21:1827–41. 10.1080/15384101.2022.2073047. 35509127 10.1080/15384101.2022.2073047 PMC9359382

[60] Chitty JL, Yam M, Perryman L, Parker AL, Skhinas JN, Setargew YFI, et al. A first-in-class pan-lysyl oxidase inhibitor impairs stromal remodeling and enhances gemcitabine response and survival in pancreatic cancer. Nat Cancer. 2023;4:1326–44. 10.1038/s43018-023-00614-y. 37640930 10.1038/s43018-023-00614-y PMC10518255

[61] Sorensen I, Adams RH, Gossler A. DLL1-mediated Notch activation regulates endothelial identity in mouse fetal arteries. Blood. 2009;113:5680–8. 10.1182/blood-2008-08-174508. 19144989 10.1182/blood-2008-08-174508

[62] Shi C, Jia T, Mendez-Ferrer S, Hohl TM, Serbina NV, Lipuma L, et al. Bone marrow mesenchymal stem and progenitor cells induce monocyte emigration in response to circulating toll-like receptor ligands. Immunity. 2011;34:590–601. 10.1016/j.immuni.2011.02.016. 21458307 10.1016/j.immuni.2011.02.016 PMC3081416

[63] Radler PD, Vistisen K, Triplett AA, Dennaoui R, Li Y, Shrestha H, et al. Dual recombinase action in the normal and neoplastic mammary gland epithelium. Sci Rep. 2021;11:20775. 10.1038/s41598-021-00231-8. 34675248 10.1038/s41598-021-00231-8 PMC8531329

[64] Guy CT, Cardiff RD, Muller WJ. Induction of mammary tumors by expression of polyomavirus middle T oncogene: a transgenic mouse model for metastatic disease. Mol Cell Biol. 1992;12:954–61. 10.1128/mcb.12.3.954-961.1992. 1312220 10.1128/mcb.12.3.954-961.1992 PMC369527

[65] Bang SJ, Jensen P, Dymecki SM, Commons KG. Projections and interconnections of genetically defined serotonin neurons in mice. Eur J Neurosci. 2012;35:85–96. 10.1111/j.1460-9568.2011.07936.x. 22151329 10.1111/j.1460-9568.2011.07936.x PMC3268345

