# Supplementary material for: Lung endothelial PEAR1 induces tumor cell dormancy
Source: Mol Cancer. 2025 Nov 3;24:278. doi: 10.1186/s12943-025-02488-3 (PMC12581312; doi:10.1186/s12943-025-02488-3)
Supplement: Supplementary file 1 — Supplementary Material 1. [file 12943_2025_2488_MOESM1_ESM.pdf]

Supplementary Tables

Supplementary Table 1. Results of siRNA screen for endothelial transmembrane or secreted proteins mediating tumor cell dormancy.

Gene	Axis number	Log fold change in cell count normalized to scrambled control		
		Mean	SD	N
ITGA6	1	-1,836	0,265	4
PDGFB	2	-1,676	0,206	4
C12orf49	3	-1,529	0,029	4
ADAM9	4	-1,466	0,117	4
GPR124	5	-1,383	0,197	4
STAB1	6	-1,312	0,213	4
ADAMTS9	7	-1,280	0,347	4
BACE2	8	-1,123	0,077	4
MMRN2	9	-1,117	0,051	4
STC2	10	-1,104	0,189	4
ADM	11	-1,032	0,043	4
CECR1	12	-0,968	0,052	4
COL12A1	13	-0,945	0,134	4
STC1	14	-0,915	0,167	4
TMEM50A	15	-0,906	0,200	4
COL6A1	16	-0,877	0,359	4
KIAA1324L	17	-0,842	0,042	4
NF2	18	-0,828	0,353	4
ORAI2	19	-0,824	0,220	4
LAMB3	20	-0,812	0,364	4
MANSC1	21	-0,792	0,082	4
IL8	22	-0,782	0,296	4
MTUS1	23	-0,764	0,220	4
ARL10	24	-0,751	0,161	4
FUCA2	25	-0,749	0,175	4
SLC25A6	26	-0,732	0,377	4
C11orf41	27	-0,726	0,055	4
TFPI	28	-0,708	0,292	4
SYNJ2	29	-0,700	0,183	4
CYB5D2	30	-0,661	0,611	4
SERPINB2	31	-0,655	0,433	4
GPC1	32	-0,650	0,053	4
SPOCK1	33	-0,621	0,225	4
RHBDF1	34	-0,615	0,257	4
HHIP	35	-0,605	0,347	4

Gene	Axis number	Log fold change in cell count normalized to scrambled control		
		Mean	SD	N
CYR61	36	-0,595	0,223	4
TM4SF1	37	-0,591	0,163	4
CDH5	38	-0,590	0,334	4
ATP1A1	39	-0,589	0,011	4
GPR126	40	-0,583	0,247	4
KLF2	41	-0,578	0,460	4
C1RL	42	-0,570	0,094	4
PLAU	43	-0,568	0,270	4
TNFRSF1B	44	-0,564	0,371	4
BMP2	45	-0,558	0,297	4
ITPR3	46	-0,540	0,192	4
TSPAN15	47	-0,532	0,227	4
IL13RA1	48	-0,531	0,087	4
THSD1, THSD1P1	49	-0,531	0,369	4
SRGN	50	-0,513	0,181	4
CPQ	51	-0,508	0,409	4
COPS6	52	-0,507	0,236	4
COL4A6	53	-0,506	0,103	4
OXA1L	54	-0,505	0,218	4
BMPR2	55	-0,497	0,711	4
PAPLN	56	-0,497	0,359	4
IL32	57	-0,486	0,663	4
FSTL1	58	-0,481	0,258	4
SEMA6B	59	-0,473	0,207	4
GLB1L	60	-0,465	0,313	4
F2R	61	-0,465	0,199	4
BSG	62	-0,462	0,130	4
FLRT2	63	-0,455	0,273	4
PTPRB	64	-0,450	0,163	4
CST3	65	-0,442	0,238	4
APLP2	66	-0,438	0,317	4
ASPH	67	-0,438	0,307	4
DST	68	-0,438	0,013	4
IGFBP3	69	-0,436	0,140	4
TIMP2	73	-0,422	0,135	4

Gene	Axis number	Log fold change in cell count normalized to scrambled control		
		Mean	SD	N
NOTCH4	74	-0,420	0,107	4
CD63	75	-0,404	0,677	4
CNOT1	76	-0,399	0,232	4
CANX	77	-0,398	0,092	4
DCBLD1	78	-0,389	0,125	4
IGFBP7	79	-0,388	0,611	4
DYSF	80	-0,380	0,204	4
ABI3BP	81	-0,379	0,262	4
TGFB1	82	-0,378	0,047	4
TGFBR2	83	-0,369	0,182	4
PTDSS1	84	-0,365	0,105	4
MEGF6	85	-0,364	0,107	4
SMPDL3A	86	-0,364	0,166	4
LAMA4	87	-0,362	0,319	4
PGF	88	-0,361	0,346	4
MCL1	89	-0,358	0,224	4
SEC61A1	90	-0,357	0,130	4
IL17D	91	-0,354	0,341	4
C19orf10	92	-0,353	0,112	4
GLG1	93	-0,351	0,152	4
TNFSF15	94	-0,347	0,199	4
VAMP8	95	-0,346	0,142	4
C10orf58	70	-0,425	0,173	4
B2M	71	-0,425	0,229	4
C2orf69	72	-0,423	0,520	4
TIMP2	73	-0,422	0,135	4
NOTCH4	74	-0,420	0,107	4
CD63	75	-0,404	0,677	4
CNOT1	76	-0,399	0,232	4
CANX	77	-0,398	0,092	4
DCBLD1	78	-0,389	0,125	4
IGFBP7	79	-0,388	0,611	4
DYSF	80	-0,380	0,204	4
ABI3BP	81	-0,379	0,262	4
TGFB1	82	-0,378	0,047	4
TGFBR2	83	-0,369	0,182	4
PTDSS1	84	-0,365	0,105	4
MEGF6	85	-0,364	0,107	4
SMPDL3A	86	-0,364	0,166	4
LAMA4	87	-0,362	0,319	4
PGF	88	-0,361	0,346	4
MCL1	89	-0,358	0,224	4
SEC61A1	90	-0,357	0,130	4
IL17D	91	-0,354	0,341	4
C19orf10	92	-0,353	0,112	4
GLG1	93	-0,351	0,152	4
TNFSF15	94	-0,347	0,199	4
VAMP8	95	-0,346	0,142	4
F11R	96	-0,345	0,141	4
MFGE8	97	-0,344	0,383	4
TMED10	98	-0,339	0,201	4
DHRSX	99	-0,338	0,386	4
MBTPS1	100	-0,333	0,445	4
SLC38A1	101	-0,333	0,187	4
HERC4	102	-0,329	0,087	4
BCL2L1	103	-0,324	0,240	4
SPNS2	104	-0,324	0,197	4
IGFBP1	105	-0,324	0,636	4
GSN	106	-0,320	0,544	4
LAMC1	107	-0,316	0,018	4
PDGFRL	108	-0,309	0,092	4
SYT11	109	-0,303	0,147	4
CXCL1	110	-0,300	0,631	4
DNASE1	111	-0,299	0,046	4
CLEC1A	112	-0,297	0,244	4
ACVR1	113	-0,294	0,186	4
TMCO3	114	-0,293	0,255	4
MYCT1	115	-0,284	0,372	4
EPHB4	116	-0,282	0,424	4
SEMA3F	117	-0,281	0,187	4
FBN2	118	-0,278	0,400	4
SEL1L3	119	-0,277	0,231	4
PDGFC	120	-0,275	0,593	4
KTN1	121	-0,273	0,108	4
GPR56	122	-0,267	0,128	4
COPB2	123	-0,266	0,428	4
TCN2	124	-0,261	0,510	4
GNAS	125	-0,256	0,408	4
ARSK	126	-0,252	0,498	4
HSD17B11	127	-0,250	0,494	4
PSAP	128	-0,250	0,116	4
ERMP1	129	-0,249	0,478	4
ATL3	130	-0,245	0,473	4
STT3A	131	-0,240	0,188	4

Gene	Axis number	Log fold change in cell count normalized to scrambled control		
		Mean	SD	N
VAMP3	132	-0,232	0,254	4
BCAP29	133	-0,231	0,026	4
PLXND1	134	-0,230	0,341	4
SLC35F2	135	-0,230	0,223	4
VWCE	136	-0,228	0,348	4
CNPY4	137	-0,227	0,152	4
FAM20C	138	-0,226	0,412	4
MERTK	139	-0,226	0,220	4
AXL	140	-0,224	0,171	4
ATP2B4	141	-0,224	0,227	4
VOPP1	142	-0,222	0,312	4
POSTN	143	-0,221	0,327	4
CHID1	144	-0,211	0,753	4
MMP16	145	-0,206	0,002	4
S1PR1	146	-0,205	0,082	4
PDGFA	147	-0,203	0,458	4
HEG1	148	-0,201	0,223	4
LAMP1	149	-0,201	0,160	4
IFITM3	150	-0,201	0,251	4
ANGPTL2	151	-0,195	0,373	4
TNFRSF10D	152	-0,191	0,206	4
CHSY1	153	-0,191	0,248	4
NT5E	154	-0,190	0,284	4
LRRC32	155	-0,189	0,232	4
UBE2J1	156	-0,189	0,074	4
ITGBL1	157	-0,183	0,222	4
CRELD2	158	-0,176	0,209	4
PPT1	159	-0,174	0,329	4
MTDH	160	-0,173	0,044	4
MBOAT7	161	-0,171	0,242	4
LAMC2	162	-0,165	0,086	4
LY96	163	-0,164	0,083	4
C14orf93	164	-0,156	0,131	4
CTHRC1	165	-0,140	0,389	4
SLC25A3	166	-0,139	0,153	4
VASH1	167	-0,137	0,127	4
EPHB2	168	-0,131	0,097	4
ATP11A	169	-0,130	0,163	4
ATP8B1	170	-0,127	0,243	4
SLC29A1	171	-0,124	0,139	4
ITGA2	172	-0,122	0,519	4
TMX3	173	-0,118	0,318	4
CD81	174	-0,116	0,285	4
ACVRL1	175	-0,116	0,116	4
BCHE	176	-0,113	0,377	4
CPT1C	177	-0,113	0,148	4
NPR3	178	-0,113	0,349	4
TLR4	179	-0,109	0,114	4
COL1A2	180	-0,109	0,311	4
VEGFC	181	-0,106	0,025	4
HMOX2	182	-0,100	0,349	4
LPHN2	183	-0,097	0,109	4
LRIG3	184	-0,097	0,076	4
EMCN	185	-0,097	0,302	4
CD164	186	-0,093	0,188	4
FSTL5	187	-0,090	0,074	4
COL8A1	188	-0,089	0,372	4
LIPG	189	-0,083	0,111	4
TCTN1	190	-0,082	0,158	4
MANF	191	-0,081	0,270	4
PVRL2	192	-0,080	0,232	4
PARP12	193	-0,079	0,068	4
NID1	194	-0,078	0,196	4
MDK	195	-0,078	0,503	4
CLEC11A	196	-0,076	0,147	4
TNFRSF10B	197	-0,075	0,489	4
TNFSF12	198	-0,072	0,058	4
SSR3	199	-0,068	0,071	4
ABCA3	200	-0,056	0,138	4
METRNL	201	-0,056	0,093	4
PROCR	202	-0,056	0,095	4
ATP2A2	203	-0,055	0,074	4
RTN4	204	-0,051	0,068	4
COL4A1	205	-0,049	0,223	4
DHRS4L2	206	-0,047	0,369	4
STX7	207	-0,046	0,110	4
ADAM10	208	-0,039	0,155	4
TSKU	209	-0,038	0,300	4
ELOVL1	210	-0,037	0,029	4
ABHD15	211	-0,035	0,090	4
SCARF1	212	-0,035	0,335	4
CTSB	213	-0,031	0,323	4
GBP2	214	-0,030	0,200	4
C4orf48	215	-0,028	0,217	4

Gene	Axis number	Log fold change in cell count normalized to scrambled control			Gene	Axis number	Log fold change in cell count normalized to scrambled control		
		Mean	SD	N			Mean	SD	N
LRRC17	216	-0,026	0,239	4	PLSCR1	258	0,033	0,268	4
FN1	217	-0,023	0,360	4	PXDN	259	0,037	0,184	4
CMKLR1	218	-0,020	0,297	4	OSTC	260	0,038	0,058	4
THSD7A	219	-0,019	0,083	4	FJX1	261	0,040	0,361	4
NXPE3	220	-0,018	0,068	4	MYOF	262	0,041	0,340	4
F2RL1	221	-0,018	0,191	4	CPD	263	0,042	0,304	4
CREG1	222	-0,017	0,215	4	HSP90AB1	264	0,042	0,075	4
SPTLC2	223	-0,017	0,220	4	C3orf58	265	0,044	0,147	4
CXorf36	224	-0,016	0,470	4	IL18BP	266	0,053	0,187	4
STOM	225	-0,012	0,121	4	PTX3	267	0,057	0,161	4
LTBP1	226	-0,009	0,117	4	ROBO4	268	0,060	0,215	4
EFEMP1	227	-0,009	0,200	4	PLAT	269	0,060	0,076	4
COL4A5	228	-0,009	0,157	4	LTBP2	270	0,061	0,318	4
CTSD	229	-0,003	0,150	4	CKAP4	271	0,065	0,475	4
FNDC3B	230	-0,001	0,164	4	EI24	272	0,068	0,132	4
SDCBP	231	0,000	0,164	4	ADAM23	273	0,073	0,140	4
APOA1BP	232	0,000	0,245	4	SCD5	274	0,075	0,148	4
CALCRL	233	0,001	0,369	4	LAMB1	275	0,078	0,364	4
LTBP3	234	0,002	0,223	4	FGFR1	276	0,079	0,506	4
SEMA3A	235	0,002	0,128	4	LRIG1	277	0,081	0,071	4
SCCPDH	236	0,002	0,247	4	TM9SF3	278	0,081	0,202	4
TMEM173	237	0,002	0,082	4	IL4R	279	0,086	0,168	4
S100A10	238	0,003	0,095	4	MPZL2	280	0,088	0,195	4
RPN2	239	0,005	0,334	4	DPY19L1	281	0,090	0,203	4
EMILIN2	240	0,005	0,240	4	CTGF	282	0,090	0,167	4
RSPRY1	241	0,006	0,171	4	CD99	283	0,090	0,340	4
PVR	242	0,007	0,180	4	PMP22	284	0,092	0,244	4
CALD1	243	0,008	0,065	4	PTPRF	285	0,093	0,227	4
TNC	244	0,008	0,238	4	PRSS3	286	0,094	0,319	4
BMP1	245	0,010	0,036	4	ATP1B3	287	0,096	0,100	4
TMEM44	246	0,012	0,235	4	HTRA1	288	0,107	0,019	4
CD46	247	0,012	0,189	4	BDP1	289	0,108	0,120	4
BGN	248	0,013	0,234	4	APLN	290	0,108	0,185	4
TOR2A	249	0,013	0,268	4	QPCT	291	0,109	0,254	4
TM9SF2	250	0,014	0,040	4	NENF	292	0,109	0,194	4
TINAGL1	251	0,017	0,083	4	COLEC12	293	0,109	0,114	4
SDF2	252	0,018	0,264	4	GIMAP1	294	0,110	0,403	4
TIMM22	253	0,021	0,582	4	COL27A1	295	0,110	0,487	4
ADCY4	254	0,025	0,325	4	ANGPTL4	296	0,112	0,173	4
SPARC	255	0,029	0,270	4	RPN1	297	0,117	0,301	4
ESAM	256	0,030	0,256	4	BTD	298	0,124	0,192	4
GOLM1	257	0,032	0,176	4	CCDC80	299	0,130	0,383	4

Gene	Axis number	Log fold change in cell count normalized to scrambled control		
		Mean	SD	N
ESM1	300	0,131	0,611	4
ARL6IP1	301	0,132	0,042	4
STX3	302	0,134	0,287	4
SCD	303	0,136	0,203	4
EXT1	304	0,136	0,178	4
GJA1	305	0,147	0,100	4
NHLRC3	306	0,149	0,144	4
COL18A1	307	0,149	0,342	4
ENTPD4	308	0,149	0,168	4
TIMP3	309	0,150	0,140	4
ICAM2	310	0,152	0,219	4
IL6ST	311	0,153	0,287	4
INHBA	312	0,154	0,108	4
GPR176	313	0,157	0,061	4
KIAA1797	314	0,157	0,329	4
FAM174B	315	0,160	0,400	4
PLXNA2	316	0,160	0,110	4
TSPAN14	317	0,164	0,124	4
VANGL1	318	0,165	0,094	4
QSOX1	319	0,170	0,125	4
NRP1	320	0,177	0,213	4
DCHS1	321	0,181	0,126	4
KLF4	322	0,184	0,145	4
PCDH12	323	0,189	0,217	4
TNFSF10	324	0,189	0,142	4
CLDN5	325	0,191	0,176	4
PRNP	326	0,192	0,057	4
ADAM15	327	0,198	0,192	4
GAS6	328	0,200	0,076	4
KDR	329	0,201	0,188	4
LRRC8C	330	0,206	0,146	4
PLA2G12A	331	0,208	0,023	4
MMP1	332	0,208	0,301	4
CLSTN1	333	0,209	0,153	4
EFNB2	334	0,209	0,094	4
HTRA3	335	0,210	0,189	4
LAPTM5	336	0,211	0,075	4
TSPAN18	337	0,213	0,199	4
SSR1	338	0,219	0,388	4
NGRN	339	0,220	0,361	4
ECM1	340	0,224	0,098	4
ITGA5	341	0,227	0,283	4

Gene	Axis number	Log fold change in cell count normalized to scrambled control		
		Mean	SD	N
SPATA20	342	0,228	0,253	4
ALDOA	343	0,229	0,177	4
CMTM3	344	0,229	0,175	4
MMP11	345	0,230	0,646	4
GDF11	346	0,231	0,513	4
MTCH1	347	0,235	0,121	4
GRN	348	0,236	0,182	4
ENTPD1	349	0,239	0,238	4
MARVELD1	350	0,244	0,112	4
DKK1	351	0,245	0,216	4
FADS2	352	0,248	0,090	4
TNFRSF10C	353	0,250	0,227	4
LPCAT2	354	0,250	0,074	4
CLDN11	355	0,253	0,310	4
IGFBP4	356	0,255	0,382	4
DRAXIN	357	0,255	0,126	4
EFEMP2	358	0,256	0,220	4
NID2	359	0,260	0,083	4
ADAMTS7	360	0,261	0,234	4
RNF144B	361	0,261	0,137	4
FLT1	362	0,263	0,313	4
SRPX2	363	0,263	0,124	4
FAT4	364	0,268	0,221	4
SPCS3	365	0,272	0,230	4
COL5A2	366	0,273	0,474	4
ENTPD5	367	0,277	0,128	4
SFRP1	368	0,284	0,186	4
LAMB2	369	0,289	0,160	4
CHST15	370	0,291	0,293	4
ADPGK	371	0,291	0,600	4
GALNT7	372	0,291	0,184	4
TSPAN6	373	0,296	0,080	4
ARMCX2	374	0,296	0,026	4
KIAA0100	375	0,298	0,273	4
IL1RL1	376	0,302	0,095	4
COX4I1	377	0,309	0,128	4
ARMCX3	378	0,309	0,285	4
LAPTM4A	379	0,310	0,149	4
VEGFA	380	0,310	0,265	4
VAMP5	381	0,311	0,307	4
ATP6V0E1	382	0,313	0,434	4
P2RX4	383	0,314	0,078	4

Gene	Axis number	Log fold change in cell count normalized to scrambled control		
		Mean	SD	N
SLC7A7	384	0,316	0,157	4
CPT1A	385	0,321	0,174	4
BMP4	386	0,324	0,046	4
FAM131A	387	0,324	0,039	4
FAM172A	388	0,327	0,107	4
NTN4	389	0,328	0,097	4
IFITM2	390	0,331	0,315	4
ENG	391	0,337	0,239	4
PCYOX1L	392	0,344	0,063	4
LAMA3	393	0,352	0,125	4
TGOLN2	394	0,353	0,250	4
FZD4	395	0,353	0,065	4
IGFBP2	396	0,357	0,181	4
APP	397	0,364	0,088	4
MST1	398	0,366	0,261	4
PRADC1	399	0,372	0,226	4
MATN2	400	0,374	0,124	4
DKK3	401	0,374	0,163	4
PTPRM	402	0,376	0,105	4
PON2	403	0,378	0,322	4
TTYH3	404	0,380	0,136	4
VEGFB	405	0,382	0,144	4
VWF	406	0,387	0,262	4
GPX3	407	0,390	0,044	4
TM4SF18	408	0,395	0,054	4
COL4A2	409	0,401	0,194	4
C6orf120	410	0,401	0,087	4
NRCAM	411	0,410	0,211	4
STT3B	412	0,410	0,065	4
ITGB1	413	0,411	0,039	4
GOLGB1	414	0,411	0,053	4
RNPEP	415	0,414	0,023	4
CAV2	416	0,416	0,123	4
GIMAP2	417	0,418	0,246	4
TMEM184B	418	0,420	0,424	4
TMEM109	419	0,422	0,031	4
IGFBP6	420	0,423	0,263	4
STX4	421	0,429	0,077	4
CSF2RB	422	0,436	0,180	4
MFAP2	423	0,446	0,077	4
CFH	424	0,457	0,083	4
IFI27	425	0,458	0,187	4
HSPG2	426	0,463	0,033	4
SLC38A2	427	0,467	0,342	4
PODXL	428	0,470	0,108	4
ANO10	429	0,475	0,226	4
LOXL2	430	0,476	0,037	4
NDST1	431	0,476	0,121	4
TM2D2	432	0,481	0,140	4
FAM171A1	433	0,488	0,067	4
DDOST	434	0,492	0,303	4
GDF15	435	0,509	0,086	4
NPC2	436	0,509	0,179	4
GPX7	437	0,510	0,249	4
DPP7	438	0,515	0,276	4
ARHGEF10	439	0,518	0,165	4
IDE	440	0,521	0,016	4
GDF3	441	0,530	0,323	4
PLTP	442	0,531	0,104	4
TSPAN3	443	0,533	0,117	4
TRAM2	444	0,533	0,136	4
YIPF5	445	0,539	0,178	4
ECE1	446	0,541	0,126	4
EMP1	447	0,544	0,312	4
ITGAV	448	0,548	0,084	4
SEC22C	449	0,549	0,084	4
IGF2R	450	0,551	0,237	4
CFI	451	0,551	0,178	4
TNFRSF11A	452	0,555	0,236	4
CD93	453	0,556	0,110	4
TEK	454	0,558	0,165	4
CD9	455	0,567	0,169	4
ITM2B	456	0,569	0,030	4
SLC7A11	457	0,570	0,233	4
EDN1	458	0,578	0,247	4
TMEM123	459	0,580	0,238	4
CXCL2	460	0,582	0,121	4
MATN3	461	0,582	0,422	4
STX12	462	0,587	0,200	4
PDGFD	463	0,592	0,138	4
ANGPT2	464	0,593	0,184	4
CRTAP	465	0,594	0,086	4
GALNT1	466	0,594	0,068	4
TMED2	467	0,595	0,217	4

Gene	Axis number	Log fold change in cell count normalized to scrambled control		
		Mean	SD	N
LCAT	484	0,653	0,031	4
SERINC3	485	0,656	0,181	4
CALR	486	0,657	0,271	4
ELTD1	487	0,661	0,149	4
C1orf54	488	0,662	0,040	4
GGH	489	0,664	0,117	4
NUCB1	490	0,677	0,104	4
LAMA5	491	0,690	0,071	4
CRIM1	492	0,691	0,069	4
MMRN1	493	0,697	0,065	4
PTPRE	494	0,700	0,069	4
NOTCH1	495	0,718	0,165	4
SERPINE1	496	0,725	0,048	4
ANPEP	497	0,728	0,099	4
A2M	498	0,730	0,151	4
BST2	499	0,732	0,067	4
CLU	500	0,736	0,087	4
PLSCR4	501	0,752	0,110	4
CALU	502	0,753	0,086	4
COL5A1	503	0,757	0,112	4
TSPAN5	504	0,773	0,067	4
JAM3	505	0,781	0,114	4
GANAB	506	0,788	0,242	4
CYTL1	507	0,789	0,171	4
EPB41L3	508	0,797	0,060	4

Gene	Axis number	Log fold change in cell count normalized to scrambled control		
		Mean	SD	N
GPX8	509	0,798	0,201	4
C18orf54	510	0,802	0,196	4
EGFL7	511	0,804	0,253	4
CYYR1	512	0,817	0,265	4
FBN1	513	0,821	0,123	4
TGFBI	514	0,832	0,323	4
TMCC3	515	0,844	0,099	4
DNAJC10	516	0,851	0,229	4
LTBP4	517	0,866	0,104	4
WLS	518	0,873	0,091	4
PTTG1IP	519	0,910	0,132	4
DNAJC13	520	0,911	0,315	4
CCBE1	521	0,943	0,091	4
HPSE	522	0,947	0,156	4
TIMP1	523	0,955	0,292	4
CXCL11	524	0,973	0,159	4
NBL1	525	0,988	0,379	4
TMEM30A	526	0,995	0,184	4
HUWE1	527	1,000	0,144	4
**MCAM**	**528**	**1,004**	**0,103**	**4**
**CCL2**	**529**	**1,052**	**0,078**	**4**
**THBS1**	**530**	**1,101**	**0,083**	**4**
**BMP6**	**531**	**1,309**	**0,106**	**4**
**PEAR1**	**532**	**1,340**	**0,054**	**4**
**TC only**	**533**	**1,138**	**0,325**	**4**

Listed are the 532 gene names, numbers in the X-axis of Fig. 2A corresponding to siRNA pools as well as the mean and standard deviation of the fold change in cell count normalized to scrambled control, and the number of replicates.

Supplementary Table 2. Sequences of siRNAs used in the study.

Gene name	Sense	Antisense
A2M_1	GAGUUGAUUCCACUAGUAU[dT][dT]	AUACUAGUGGAAUCAACUC[dT][dT]
A2M_2	GUUAUGGGUACCUCUCUUA[dT][dT]	UAAGAGAGGUACCCAUAAC[dT][dT]
A2M_3	GCAAGAUGUCCCAGUAAGA[dT][dT]	UCUUACUGGGACAUCUUGC[dT][dT]
ABCA3_1	GACUUUGAGGACUACAUUA[dT][dT]	UAAUGUAGUCCUCAAAGUC[dT][dT]
ABCA3_2	GAAAUACGGUGCCGGCUAU[dT][dT]	AUAGCCGGCACCGUAUUUC[dT][dT]
ABCA3_3	CAAGUUUAUCUCAUCCUUU[dT][dT]	AAAGGAUGAGAUAAACUUG[dT][dT]
ABHD15_1	GGAUGAGACGUUUGUGUAU[dT][dT]	AUACACAAACGUCUCAUCC[dT][dT]
ABHD15_2	CGCUGUUCGCGGUGAGCGA[dT][dT]	UCGCUCACCGCGAACAGCG[dT][dT]
ABHD15_3	CUCUGACAACUGAACUCUU[dT][dT]	AAGAGUUCAGUUGUCAGAG[dT][dT]
ABI3BP_1	CAAGAACCUUUCACAACUA[dT][dT]	UAGUUGUGAAAGGUUCUUG[dT][dT]
ABI3BP_2	GAUACUAGUCCACCUUAUU[dT][dT]	AAUAAGGUGGACUAGUAUC[dT][dT]
ABI3BP_3	CUCAAGACACAAUUAGCUA[dT][dT]	UAGCUAAUUGUGUCUUGAG[dT][dT]
ACVR1_1	CACAGAAUUUCCACUUGGA[dT][dT]	UCCAAGUGGAAAUUCUGUG[dT][dT]
ACVR1_2	CAGCUUACUACUCUGGAUA[dT][dT]	UAUCCAGAGUAGUAAGCUG[dT][dT]
ACVR1_3	GUUCUCAGACCCGACAUUA[dT][dT]	UAAUGUCGGGUCUGAGAAC[dT][dT]
ACVRL1_1	CUGAGCAGGGCGACACGAU[dT][dT]	AUCGUGUCGCCCUGCUCAG[dT][dT]
ACVRL1_2	CAGCCAUUGCCCACCGCGA[dT][dT]	UCGCGGUGGGCAAUGGCUG[dT][dT]
ACVRL1_3	CCCAUCAGUUUCUCUCUGU[dT][dT]	ACAGAGAGAAACUGAUGGG[dT][dT]
ADAM10_1	CAGUCAUGUUAAAGCGAUU[dT][dT]	AAUCGCUUUAACAUGACUG[dT][dT]
ADAM10_2	CUAUAGUGACCAGUGUAAA[dT][dT]	UUUACACUGGUCACUAUAG[dT][dT]
ADAM10_3	CUCACAAGUAACAGUUGAA[dT][dT]	UUCAACUGUUACUUGUGAG[dT][dT]
ADAM15_1	CCAUGAUUUGCCUGGGAAU[dT][dT]	AUUCCCAGGCAAAUCAUGG[dT][dT]
ADAM15_2	CAUUAUUUCGCGAAUCCAA[dT][dT]	AUGGCAAUUAAUUAGUGAC[dT][dT]
ADAM15_3	GCUUUCUGCGGAAAUAUGU[dT][dT]	AGGGUCCACACAGAAGUCG
ADAM23_1	CCUUCAAGACUCAUAUAUU[dT][dT]	AAUAUAUGAGUCUUGAAGG[dT][dT]
ADAM23_2	GAAUGAGUAUGGUCUUCCA[dT][dT]	UGGAAGACCAUACUCAUUC[dT][dT]
ADAM23_3	GUUCGAUCCUACUCAGCAA[dT][dT]	UUGCUGAGUAGGAUCGAAC[dT][dT]
ADAM9_1	CAUAUUAUUCACUUGGAAA[dT][dT]	UUUCCAAGUGAAUAAUAUG[dT][dT]
ADAM9_2	GAAUCACGAUGAUGGGAGA[dT][dT]	UCUCCCAUCAUCGUGAUUC[dT][dT]
ADAM9_3	GUGAGAAUGUACAAGAGAU[dT][dT]	AUCUCUUGUACAUUCUCAC[dT][dT]
ADAMTS7_1	GAGAUUGACUCCGGUGCUA[dT][dT]	UAGCACCGGAGUCAAUCUC[dT][dT]
ADAMTS7_2	GCAUCAACGGCAUCUGUAA[dT][dT]	UUACAGAUGCCGUUGAUGC[dT][dT]
ADAMTS7_3	GUAAGAACGUGGGCUGUGA[dT][dT]	UCACAGCCCACGUUCUUAC[dT][dT]

ADAMTS9_1	CCACUUAUGCCACACUGAU[dT][dT]	AUCAGUGUGGCAUAAGUGG[dT][dT]
ADAMTS9_2	CCGUAAUGUCUAUAACUGU[dT][dT]	ACAGUUAUAGACAUUACGG[dT][dT]
ADAMTS9_3	CCCUAUAGAAGCUGUUCUA[dT][dT]	UAGAACAGCUUCUAUAGGG[dT][dT]
ADCY4_1	CUAAGGAGCUGGUGCUCAU[dT][dT]	AUGAGCACCAGCUCCUUAG[dT][dT]
ADCY4_2	CCAUUACCAGCCUGUUCUU[dT][dT]	AAGAACAGGCUGGUAAUGG[dT][dT]
ADCY4_3	GUUGAACCAUGGACCCGUA[dT][dT]	UACGGGUCCAUGGUUCAAC[dT][dT]
ADM_1	CGCUGACACCGCUCGGUUG[dT][dT]	CAACCGAGCGGUGUCAGCG[dT][dT]
ADM_2	CGUCGGAGUUUCGAAAGAA[dT][dT]	UUCUUUCGAAACUCCGACG[dT][dT]
ADM_3	GGUUGGAUGUCGCGUCGGA[dT][dT]	UCCGACGCGACAUCCAACC[dT][dT]
ADPGK_1	GGAUUGUAUUAAACCCAAA[dT][dT]	UUUGGGUUUAAUACAAUCC[dT][dT]
ADPGK_2	GAACUAGCCAACUUAAGAA[dT][dT]	UUCUUAAGUUGGCUAGUUC[dT][dT]
ADPGK_3	GAUUAAACUGUAUCAGGUA[dT][dT]	UACCUGAUACAGUUUAAUC[dT][dT]
ALDOA_1	CUGUCACUGGGAUCACCUU[dT][dT]	AAGGUGAUCCCAGUGACAG[dT][dT]
ALDOA_2	GUGUCAUCCUCUUCCAUGA[dT][dT]	UUGGAUUCGCGAAAUAAUG
ALDOA_3	CGUCUCUAACCACGCCUAU[dT][dT]	AUAUAGCAGGUUCUAAGUC
ANGPT2_1	CCUAUAACAACUUUCGGAA[dT][dT]	UUCCGAAAGUUGUUAUAGG[dT][dT]
ANGPT2_2	CUUCCAACUUGAACGGAAU[dT][dT]	AUUCCGUUCAAGUUGGAAG[dT][dT]
ANGPT2_3	CAAAUAAGUUCAACGGCAU[dT][dT]	AUGCCGUUGAACUUAUUUG[dT][dT]
ANGPTL2_1	GGCAUUGUGAGCGAGGUGA[dT][dT]	UCACCUCGCUCACAAUGCC[dT][dT]
ANGPTL2_2	GCCAUUACCGGAGCCGCUA[dT][dT]	UAGCGGCUCCGGUAAUGGC[dT][dT]
ANGPTL2_3	GUUUCCGCCUGGAACCUGA[dT][dT]	UCAGGUUCCAGGCGGAAAC[dT][dT]
ANGPTL4_1	GCAUGGAGCUUCACUCCUU[dT][dT]	AAGGAGUGAAGCUCCAUGC[dT][dT]
ANGPTL4_2	GGGACAAGAACUGCGCCAA[dT][dT]	UUGGCGCAGUUCUUGUCCC[dT][dT]
ANGPTL4_3	GGAAUCUUCUGGAAGACCU[dT][dT]	AGGUCUUCCAGAAGAUUCC[dT][dT]
ANO10_1	GUAUCAGCCCAUAGACAGU[dT][dT]	ACUGUCUAUGGGCUGAUAC[dT][dT]
ANO10_2	CCUAUCAGAACCAUCUAAU[dT][dT]	AUUAGAUGGUUCUGAUAGG[dT][dT]
ANO10_3	CACUCAAGCAGCAGCAAAU[dT][dT]	AUUUGCUGCUGCUUGAGUG[dT][dT]
ANPEP_1	CCAUGAAGGCCGAGUUCAA[dT][dT]	UUGAACUCGGCCUUCAUGG[dT][dT]
ANPEP_1	CAGAUGACCUGGCGGGCUU[dT][dT]	AAGCCCGCCAGGUCAUCUG[dT][dT]
ANPEP_2	GCUAUUACCGGGUGAACUA[dT][dT]	UCAUGGAAGAGGAUGACAC
ANPEP_2	GGAAUGACCUGUGGCUGAA[dT][dT]	UUCAGCCACAGGUCAUUCC[dT][dT]
ANPEP_3	GCGAUUAUGCCCUGAACGU[dT][dT]	ACAUAUUUCCGCAGAAAGC
ANPEP_3	CCAACAACGUCAUUGGGCA[dT][dT]	UGCCCAAUGACGUUGUUGG[dT][dT]
APLN_1	CGGUUAUGUCUCCUCCAUA[dT][dT]	UAUGGAGGAGACAUAACCG[dT][dT]
APLN_2	GAUGAUUCCCGUUCUCUCA[dT][dT]	UGAGAGAACGGGAAUCAUC[dT][dT]

APLN_3	GGCUGUAGUUUGGAUGAUU[dT][dT]	AAUCAUCCAAACUACAGCC[dT][dT]
APLNR_1	CAGUCUGAGUGUGAGUACA[dT][dT]	UGUACUCACACUCAGACUG[dT][dT]
APLNR_2	GCAUCGAGGGCCUGCGGAA[dT][dT]	UUCCGCAGGCCCUCGAUGC[dT][dT]
APLNR_3	GAGAACAGAUGCACGAGAA[dT][dT]	UUCUCGUGCAUCUGUUCUC[dT][dT]
APLP2_1	CAAAUGUGAUGGAGGCAAA[dT][dT]	UUUGCCUCCAUCACAUUUG[dT][dT]
APLP2_2	GCUUACAUACCAUCCGUCA[dT][dT]	UGACGGAUGGUAUGUAAGC[dT][dT]
APLP2_3	CUGAGUAGCAGUGCUCUCA[dT][dT]	UGAGAGCACUGCUACUCAG[dT][dT]
APOA1BP_1	CAGACUUGCUCAUAUCCCU[dT][dT]	AGGGAUAUGAGCAAGUCUG[dT][dT]
APOA1BP_2	CCCAUUGCCAGCAUCGACA[dT][dT]	UGUCGAUGCUGGCAAUGGG[dT][dT]
APOA1BP_3	CCCAGUUUACCGGUCGCUA[dT][dT]	UAGCGACCGGUAAACUGGG[dT][dT]
APP_1	CUUAGUUGGUGAGUUUGUA[dT][dT]	UACAAACUCACCAACUAAG[dT][dT]
APP_2	CCAUCUUUGACCGAAACGA[dT][dT]	UAGUUCACCCGGUAAUAGC
APP_3	GUGUGAUUUAUGAGCGCAU[dT][dT]	AUAGGCGUGGUUAGAGACG
ARHGEF10_1	CUAAGCUGGUGCUCCCGAU[dT][dT]	AUCGGGAGCACCAGCUUAG[dT][dT]
ARHGEF10_2	CUCAUGACAGCCGUGUGAU[dT][dT]	AUCACACGGCUGUCAUGAG[dT][dT]
ARHGEF10_3	CUACACUGGUUGGGAAUAA[dT][dT]	UUAUUCCCAACCAGUGUAG[dT][dT]
ARL10_1	GCACGUUCCUGCGCGUGUU[dT][dT]	AACACGCGCAGGAACGUGC[dT][dT]
ARL10_2	CUUGCCACCUGCCUGUCAA[dT][dT]	UUGACAGGCAGGUGGCAAG[dT][dT]
ARL10_3	GAGGAUAGUGUCUGGCUCA[dT][dT]	UGAGCCAGACACUAUCCUC[dT][dT]
ARL6IP1_1	GUACUAUCUGGAUACUAAA[dT][dT]	UUUAGUAUCCAGAUAGUAC[dT][dT]
ARL6IP1_2	GAUAGUGACUUCCUUACUA[dT][dT]	UCGUUUCGGUCAAAGAUGG
ARL6IP1_3	CCUAAGAUGUACUUCAUGA[dT][dT]	ACGUUCAGGGCAUAAUCGC
ARL6IP1_4	CUAUCUAGAUCCAUCUGUU[dT][dT]	AACAGAUGGAUCUAGAUAG
ARMCX2_1	CAAAGUUGAAGUAGACGAA[dT][dT]	UUCGUCUACUUCAACUUUG[dT][dT]
ARMCX2_2	CAAUUCCAUUGCAAACUUU[dT][dT]	AAAGUUUGCAAUGGAAUUG[dT][dT]
ARMCX2_3	GAACAAAUUCUGAUUGGUU[dT][dT]	AACCAAUCAGAAUUUGUUC[dT][dT]
ARMCX3_1	GGAUCCCAUAGUUAAGGAA[dT][dT]	UUCCUUAACUAUGGGAUCC[dT][dT]
ARMCX3_2	GACUGUUACUAAUGAGUAU[dT][dT]	AUACUCAUUAGUAACAGUC[dT][dT]
ARMCX3_3	GAGUUGGUCUUUAGGUUUA[dT][dT]	UAAACCUAAAGACCAACUC[dT][dT]
ARSK_1	GAAUUAAAGCCGGCCUACA[dT][dT]	UGUAGGCCGGCUUUAAUUC[dT][dT]
ARSK_2	GGACUAAAGUCAGAGUGAU[dT][dT]	AUCACUCUGACUUUAGUCC[dT][dT]
ARSK_3	GAUACUCUUUGUUGCCGUU[dT][dT]	AACGGCAACAAAGAGUAUC[dT][dT]
ASPH_1	CCAUGAUACAGAUGAUGUA[dT][dT]	AACCUUUGGCCAAGAGUUC
ASPH_2	CAAAUACCCUCAGAGUCCA[dT][dT]	AUGGAUGCCAUCACUGGUC
ASPH_3	GACUGAUGUAGAUGAUAGA[dT][dT]	UCUGGAAGGCAUAAGUUGG

ATL3_1	GAAAGAUCAACAUUCCUUU[dT][dT]	AAAGGAAUGUUGAUCUUUC[dT][dT]
ATL3_2	GAAAUUCAGAAUGUUCGAA[dT][dT]	UUCGAACAUUCUGAAUUUC[dT][dT]
ATL3_3	GUAUUCUGGUCAAUAUCGU[dT][dT]	ACGAUAUUGACCAGAAUAC[dT][dT]
ATP11A_1	GGGUUAUGAAGACUGGCUU[dT][dT]	AAGCCAGUCUUCAUAACCC[dT][dT]
ATP11A_2	GGAUCAAAGUCUGGGUUCU[dT][dT]	AGAACCCAGACUUUGAUCC[dT][dT]
ATP11A_3	CUAAAGCUUGCAUUGGACA[dT][dT]	UGUCCAAUGCAAGCUUUAG[dT][dT]
ATP1A1_1	GAGAGAAAGAUACGCCAAA[dT][dT]	UACAUCAUCUGUAUCAUGG[dT][dT]
ATP1A1_2	CGUAUAUGACGAAGUCAGA[dT][dT]	UAGUAAGGAAGUCACUAUC
ATP1A1_3	CCUUUCAGAACGCCUAUUU[dT][dT]	AUGCGCUCAUAAAUCACAC
ATP1A1_4	CAAGUACCAGUUGUCUAUU[dT][dT]	AAUAGACAACUGGUACUUG
ATP1B3_1	CAUUCACGAUGUGGGUUAU[dT][dT]	AUAACCCACAUCGUGAAUG[dT][dT]
ATP1B3_2	GUGGUAUGAAUGAUCCUGA[dT][dT]	UCAGGAUCAUUCAUACCAC[dT][dT]
ATP1B3_3	GAUAUCUCCACAGAGUAAA[dT][dT]	UUUACUCUGUGGAGAUAUC[dT][dT]
ATP2A2_1	CCUAUGACCUCUGGAGUCA[dT][dT]	UUUGGCGUAUCUUUCUCUC[dT][dT]
ATP2A2_2	CACCUAUUGGAGAAGUGCA[dT][dT]	UGGACUCUGAGGGUAUUUG
ATP2A2_3	CCCUUAUUUGCAUUGCAGU[dT][dT]	UCAUGAAGUACAUCUUAGG
ATP2B4_1	CCAUGAACCGCAUGACUGU[dT][dT]	UGACUCCAGAGGUCAUAGG[dT][dT]
ATP2B4_2	CAGUGAUCAUCGUGGUGUU[dT][dT]	UCUGACUUCGUCAUAUACG
ATP2B4_3	CCUUUAACUCAGUGCGCAA[dT][dT]	UCUAUCAUCUACAUCAGUC
ATP6V0E1_1	CAGUUUGCUGCUAUCUCUU[dT][dT]	AAGAGAUAGCAGCAAACUG[dT][dT]
ATP6V0E1_2	CUCCAAACCAGACCACUUU[dT][dT]	AAAGUGGUCUGGUUUGGAG[dT][dT]
ATP6V0E1_3	GUACUCUUCUGAGAUAGAA[dT][dT]	UUCUAUCUCAGAAGAGUAC[dT][dT]
ATP8B1_1	GAGAGUAAAUAUGCGAAUA[dT][dT]	UAUUCGCAUAUUUACUCUC[dT][dT]
ATP8B1_2	GCCUUAAUCAUCACUGGUU[dT][dT]	AACCAGUGAUGAUUAAGGC[dT][dT]
ATP8B1_3	GGAUACUUCUUAUUGGACU[dT][dT]	AGUCCAAUAAGAAGUAUCC[dT][dT]
AXL_1	GGUACAUUGGCUUCGGGAU[dT][dT]	AUCCCGAAGCCAAUGUACC[dT][dT]
AXL_2	GUCAUCUUACCUUUCAUGA[dT][dT]	UCAUGAAAGGUAAGAUGAC[dT][dT]
AXL_3	GACGAAAUCCUCUAUGUCA[dT][dT]	UGACAUAGAGGAUUUCGUC[dT][dT]
B2M_1	CAUUCAGACUUGUCUUUCA[dT][dT]	UGAAAGACAAGUCUGAAUG[dT][dT]
B2M_2	GUUUGAAGAUGCCGCAUUU[dT][dT]	AAAUGCGGCAUCUUCAAAC[dT][dT]
B2M_3	CGACAUUGAAGUUGACUUA[dT][dT]	UAAGUCAACUUCAAUGUCG[dT][dT]
BACE2_1	CGCAGAAGCUACAGAUUCU[dT][dT]	AGAAUCUGUAGCUUCUGCG[dT][dT]
BACE2_2	GACCAACGGAGGUAGUCUU[dT][dT]	AAGACUACCUCCGUUGGUC[dT][dT]
BACE2_3	GGGUCAAAGUGGUACAGGA[dT][dT]	UCCUGUACCACUUUGACCC[dT][dT]
BCAP29_1	CUUACCAUUAUCAUCCUAU[dT][dT]	AUAGGAUGAUAAUGGUAAG[dT][dT]

BCAP29_2	AUUUCUAGAUGCUGUGAGA[dT][dT]	UCUCACAGCAUCUAGAAAU[dT][dT]
BCAP29_3	UUCCUCAGUUCAUACCAUU[dT][dT]	AAUGGUAUGAACUGAGGAA[dT][dT]
BCHE_1	GGAACAAUUACAUGAUGGA[dT][dT]	UCCAUCAUGUAAUUGUUCC[dT][dT]
BCHE_2	GUAACAGCCUUUCUUGGAA[dT][dT]	UUCCAAGAAAGGCUGUUAC[dT][dT]
BCHE_3	GUUAUUGUAGUGUCAAUGA[dT][dT]	UCAUUGACACUACAAUAAC[dT][dT]
BCL2L1_1	CCUACAAGCUUUCCCAGAA[dT][dT]	UUCUGGGAAAGCUUGUAGG[dT][dT]
BCL2L1_2	CAGCAUAUCAGAGCUUUGA[dT][dT]	UCAAAGCUCUGAUAUGCUG[dT][dT]
BCL2L1_3	GACUAGAUUGCCUUUGUUU[dT][dT]	AAACAAAGGCAAUCUAGUC[dT][dT]
BDP1_1	GCAUUAGAAGUAGACCUAA[dT][dT]	UUAGGUCUACUUCUAAUGC[dT][dT]
BDP1_2	GUGCUAUGCCUUCACUAGA[dT][dT]	UCUAGUGAAGGCAUAGCAC[dT][dT]
BDP1_3	CUUAUACCUGGAUUAAGAA[dT][dT]	UUCUUAAUCCAGGUAUAAG[dT][dT]
BGN_1	GGCCAUUCAUGAUGAACGA[dT][dT]	UCGUUCAUCAUGAAUGGCC[dT][dT]
BGN_2	GUCCAAGGCUCCAGUCCUA[dT][dT]	UAGGACUGGAGCCUUGGAC[dT][dT]
BGN_3	GCCUUCAGCCCACUGCGGA[dT][dT]	UCCGCAGUGGGCUGAAGGC[dT][dT]
BMP1_1	GUUUCCAUCGUUCGUGAGA[dT][dT]	UCUCACGAACGAUGGAAAC[dT][dT]
BMP1_2	GGACUAUGGCCACAUUCAA[dT][dT]	UUGAAUGUGGCCAUAGUCC[dT][dT]
BMP1_3	CACAUUCAAUCGCCCAACU[dT][dT]	AGUUGGGCGAUUGAAUGUG[dT][dT]
BMP2_1	GGCCAUUGCUAGUAACUUU[dT][dT]	AAAGUUACUAGCAAUGGCC[dT][dT]
BMP2_2	CCUGCAACAGCCAACUCGA[dT][dT]	UCGAGUUGGCUGUUGCAGG[dT][dT]
BMP2_3	GUGUCUCCAAGAGACAUGU[dT][dT]	ACAUGUCUCUUGGAGACAC[dT][dT]
BMP4_1	CGAGACUGGUCCACCACAA[dT][dT]	UUGUGGUGGACCAGUCUCG[dT][dT]
BMP4_2	GAGACUGGUCCACCACAAU[dT][dT]	AUUGUGGUGGACCAGUCUC[dT][dT]
BMP4_3	GGACUUCGAGGCGACACUU[dT][dT]	AAGUGUCGCCUCGAAGUCC[dT][dT]
BMP6_1	CAGCAUUUAUCAAGUCUUA[dT][dT]	UAAGACUUGAUAAAUGCUG[dT][dT]
BMP6_2	CUUACGACAAGCAGCCCUU[dT][dT]	AAGGGCUGCUUGUCGUAAG[dT][dT]
BMP6_3	CAGAUUACAACAGCAGUGA[dT][dT]	UCACUGCUGUUGUAAUCUG[dT][dT]
BMPR2_1	GACAAUAUUAUGCUCGAAA[dT][dT]	UUUCGAGCAUAAUAUUGUC[dT][dT]
BMPR2_2	CUGCAAUUUCCCAUCGAGA[dT][dT]	UCUCGAUGGGAAAUUGCAG[dT][dT]
BMPR2_3	GCAAAUCAGGAUCAGGUGA[dT][dT]	UCACCUGAUCCUGAUUUGC[dT][dT]
BSG_1	CUCAUGAACGGCUCCGAGA[dT][dT]	ACAGUCAUGCGGUUCAUGG[dT][dT]
BSG_2	GGUCCUUGCCCUUUGUGGA[dT][dT]	UGCACUUCUCCAAUAGGUG
BSG_3	GGAUUCUGUUCCUUAGGUU[dT][dT]	AAAUAGGCGUUCUGAAAGG
BST2_1	CCCUUGAUCUCAUCAGUUC[dT][dT]	UCUCGGAGCCGUUCAUGAG[dT][dT]
BST2_2	GAAUCGCGGACAAGAAGUA[dT][dT]	AACACCACGAUGAUCACUG
BST2_3	CACACUGUGAUGGCCCUAA[dT][dT]	ACUGCAAUGCAAAUAAGGG

BTD_1	CCACUAUUUCCUGAGGAAA[dT][dT]	UUUCCUCAGGAAAUAGUGG[dT][dT]
BTD_2	CUUUGACCCUGCCAUCAGA[dT][dT]	UCUGAUGGCAGGGUCAAAG[dT][dT]
BTD_3	GACAAAGGAGCCUUGUCAU[dT][dT]	AUGACAAGGCUCCUUUGUC[dT][dT]
C10orf58_1	GGAGUACCUGGAGGAUAUA[dT][dT]	UAUAUCCUCCAGGUACUCC[dT][dT]
C10orf58_2	GGGAUUUAUCCGUCUGGGA[dT][dT]	UCCCAGACGGAUAAAUCCC[dT][dT]
C10orf58_3	CACAGACUUUGGCCUCAGA[dT][dT]	UCUGAGGCCAAAGUCUGUG[dT][dT]
C11orf41_1	CGACUUUCCCUCCAUACUU[dT][dT]	AAGUAUGGAGGGAAAGUCG[dT][dT]
C11orf41_2	GUGAAGAACCCGCCCAAUA[dT][dT]	UAUUGGGCGGGUUCUUCAC[dT][dT]
C11orf41_3	CAUAGCAAUGGGUGAGUCU[dT][dT]	AGACUCACCCAUUGCUAUG[dT][dT]
C12orf49_1	GGAAGCACCUCAUCACGGA[dT][dT]	UCCGUGAUGAGGUGCUUCC[dT][dT]
C12orf49_2	CUUACCAGAUGGAACUCUU[dT][dT]	AAGAGUUCCAUCUGGUAAG[dT][dT]
C12orf49_3	CCAUUCUAGUUGAAUAUGU[dT][dT]	ACAUAUUCAACUAGAAUGG[dT][dT]
C14orf93_1	GUCGAUAAGGCAGUGGGUU[dT][dT]	AACCCACUGCCUUAUCGAC[dT][dT]
C14orf93_2	CCAACAAUGAGUUGUUAAA[dT][dT]	UUUAACAACUCAUUGUUGG[dT][dT]
C14orf93_3	CUGUAAGAGUGAGACUAAU[dT][dT]	AUUAGUCUCACUCUUACAG[dT][dT]
C18orf54_1	CCAUUUAUCUCGCCUGAGA[dT][dT]	UCUCAGGCGAGAUAAAUGG[dT][dT]
C18orf54_2	CAGAAGAUGAUUUCUCUAA[dT][dT]	UUAGAGAAAUCAUCUUCUG[dT][dT]
C18orf54_3	CUCGUUUCACUAUAAAGAU[dT][dT]	AUCUUUAUAGUGAAACGAG[dT][dT]
C19orf10_1	CUCUCAAGGAGGGACCAAU[dT][dT]	AUUGGUCCCUCCUUGAGAG[dT][dT]
C19orf10_2	CUCUAAAGCCGCAUUUGAA[dT][dT]	UUCAAAUGCGGCUUUAGAG[dT][dT]
C19orf10_3	GUCCUAUCUGUACUUCACA[dT][dT]	UGUGAAGUACAGAUAGGAC[dT][dT]
C1orf54_1	GCAAUAGAGACUACCAUUA[dT][dT]	UAAUGGUAGUCUCUAUUGC[dT][dT]
C1orf54_2	AGACUACCAUUAGUCUUGA[dT][dT]	UCAAGACUAAUGGUAGUCU[dT][dT]
C1orf54_3	GAGACUACCAUUAGUCUUG[dT][dT]	CAAGACUAAUGGUAGUCUC[dT][dT]
C1RL_1	CUGGUCAGAGGGAGUUUGU[dT][dT]	ACAAACUCCCUCUGACCAG[dT][dT]
C1RL_2	GAAUGAGUCCCAUAACUUU[dT][dT]	AAAGUUAUGGGACUCAUUC[dT][dT]
C1RL_3	CCGAUAAUGAGACCCUCUA[dT][dT]	UAGAGGGUCUCAUUAUCGG[dT][dT]
C2orf69_1	CAUUCUGGAGGAAGCAAUA[dT][dT]	UAUUGCUUCCUCCAGAAUG[dT][dT]
C2orf69_2	CUGAGAAUUAUCAAUGGGA[dT][dT]	UCCCAUUGAUAAUUCUCAG[dT][dT]
C2orf69_3	GCAUCUAACUGUAGAUCCA[dT][dT]	UGGAUCUACAGUUAGAUGC[dT][dT]
C3orf58_1	GAGUUGACCUCGCUUGGCA[dT][dT]	UGCCAAGCGAGGUCAACUC[dT][dT]
C3orf58_2	CCACAAUUACUAUGCUGUU[dT][dT]	AACAGCAUAGUAAUUGUGG[dT][dT]
C3orf58_3	GCAAGUUUGAUGACUGUGA[dT][dT]	UCACAGUCAUCAAACUUGC[dT][dT]
C4orf48_1	CCACGCUGCUCCGUGUGAA[dT][dT]	UUCACACGGAGCAGCGUGG[dT][dT]
C4orf48_2	CACGCUGCUCCGUGUGAAU[dT][dT]	AUUCACACGGAGCAGCGUG[dT][dT]

C4orf48_3	CCGUGUGAAUAAAUGCCCA[dT][dT]	UGGGCAUUUAUUCACACGG[dT][dT]
C6orf120_1	CAGAUAGGCGCCGGGAACU[dT][dT]	AGUUCCCGGCGCCUAUCUG[dT][dT]
C6orf120_2	GAAUCUGUUCUCUGGACGA[dT][dT]	UCGUCCAGAGAACAGAUUC[dT][dT]
C6orf120_3	GCCAGAUAGGCGCCGGGAA[dT][dT]	UUCCCGGCGCCUAUCUGGC[dT][dT]
CALCRL_1	CAAAGUGUCCCAGUUCAUU[dT][dT]	GAACUGAUGAGAUCAAGGG[dT][dT]
CALCRL_1	CAUGACAGCUCAAUAUGAA[dT][dT]	UUCAUAUUGAGCUGUCAUG[dT][dT]
CALCRL_2	GUCAUGACUGUCCUAGUGA[dT][dT]	UCCACAAAGGGCAAGGACC
CALCRL_2	CAAAGUGUCCCAGUUCAUU[dT][dT]	AAUGAACUGGGACACUUUG[dT][dT]
CALCRL_3	GAAAGUGAAGACUGCACUA[dT][dT]	UUGCGCACUGAGUUAAAGG
CALCRL_3	GUCAUGACUGUCCUAGUGA[dT][dT]	UCACUAGGACAGUCAUGAC[dT][dT]
CALD1_1	GCAGUAUACCAGUGCAAUU[dT][dT]	AAUUGCACUGGUAUACUGC[dT][dT]
CALD1_2	CAAGUCACCUGCUCCCAAA[dT][dT]	UUUGGGAGCAGGUGACUUG[dT][dT]
CALD1_3	CAUGUAUAUUAUCACUAUA[dT][dT]	UAUAGUGAUAAUAUACAUG[dT][dT]
CALR_1	CAGUAUCUAUGCCUAUGAU[dT][dT]	AUCAUAGGCAUAGAUACUG[dT][dT]
CALR_2	GAGUAUUCUCCCGAUCCCA[dT][dT]	UGGGAUCGGGAGAAUACUC[dT][dT]
CALR_3	GUAUUCUCCCGAUCCCAGU[dT][dT]	ACUGGGAUCGGGAGAAUAC[dT][dT]
CALU_1	CUGGAUUUACGAGGAUGUA[dT][dT]	UACAUCCUCGUAAAUCCAG[dT][dT]
CALU_2	CUAUGAUCAUGCAGAGGCA[dT][dT]	UGCCUCUGCAUGAUCAUAG[dT][dT]
CALU_3	CCUUAGUACGGCAUGAUGA[dT][dT]	UCAUCAUGCCGUACUAAGG[dT][dT]
CANX_1	CACUAAUCUUGAAUCCAGA[dT][dT]	AAUGAACUGGGACACUUUG[dT][dT]
CANX_2	GUAAAUCCUUCACGUGAAA[dT][dT]	UACUUCUUGUCCGCGAUUC
CANX_3	CUGGUUAGAUGAUGAGCCU[dT][dT]	AACCUAAGGAACAGAAUCC
CAV1_1	CCCUAAACACCUCAACGAU[dT][dT]	AUCGUUGAGGUGUUUAGGG[dT][dT]
CAV1_2	GCAAUGUCCGCAUCAACUU[dT][dT]	AAGUUGAUGCGGACAUUGC[dT][dT]
CAV1_3	GAUAUGAACAUAUUGUUGA[dT][dT]	UCAACAAUAUGUUCAUAUC[dT][dT]
CAV2_1	CCUUCAUUGCGGGAAUUCU[dT][dT]	AGAAUUCCCGCAAUGAAGG[dT][dT]
CAV2_2	CAUUUCCUAGCUGUUCUAA[dT][dT]	UUAGAACAGCUAGGAAAUG[dT][dT]
CAV2_3	CUGGAAAUUGCAGUAACAA[dT][dT]	UUGUUACUGCAAUUUCCAG[dT][dT]
CCBE1_1	CAUAACUCCUCUAGUAUCA[dT][dT]	UGAUACUAGAGGAGUUAUG[dT][dT]
CCBE1_2	GACCAUAACUCCUCUAGUA[dT][dT]	UACUAGAGGAGUUAUGGUC[dT][dT]
CCBE1_3	CCGAUAUGACCGGGAGAGA[dT][dT]	UCUCUCCCGGUCAUAUCGG[dT][dT]
CCDC80_1	GUGUUAGAACUGUUCCCAA[dT][dT]	UUGGGAACAGUUCUAACAC[dT][dT]
CCDC80_2	GAAUGACCUACAAUGACUU[dT][dT]	AAGUCAUUGUAGGUCAUUC[dT][dT]
CCDC80_3	GAUCUCUGCUCCUAACGAU[dT][dT]	AUCGUUAGGAGCAGAGAUC[dT][dT]
CCL2_1	CCCAAACUCCGAAGACUUG[dT][dT]	CAAGUCUUCGGAGUUUGGG[dT][dT]

CCL2_2	GACUUGAACACUCACUCCA[dT][dT]	UGGAGUGAGUGUUCAAGUC[dT][dT]
CCL2_3	CUCCACAACCCAAGAAUCU[dT][dT]	AGAUUCUUGGGUUGUGGAG[dT][dT]
CD164_1	CUAUUGUUCACAUAACUCA[dT][dT]	UCUGGAUUCAAGAUUAGUG[dT][dT]
CD164_2	CUGUGACUCCAACCUCACA[dT][dT]	UCACUAGGACAGUCAUGAC
CD164_3	CGAAACAGCUGCGUUUCCU[dT][dT]	UUAGGGCCAUCACAGUGUG
CD164_4	CAGCUGCGUUUCCUGUUUU[dT][dT]	AAAACAGGAAACGCAGCUG
CD46_1	GUUAUCAGAUGCACUUUAU[dT][dT]	UGAGUUAUGUGAACAAUAG[dT][dT]
CD46_2	CAUACUAUUUGUGAUCGGA[dT][dT]	UUUCACGUGAAGGAUUUAC
CD46_3	CUUUGAAACUUGUAUGAAU[dT][dT]	UAGUGCAGUCUUCACUUUC
CD63_1	CUCUCUGUUGCCAGUGGUC[dT][dT]	AUAAAGUGCAUCUGAUAAC[dT][dT]
CD63_2	GAACUAUUGUCUUAUGAUC[dT][dT]	UGUGAGGUUGGAGUCACAG
CD63_3	CUCUUAUCAUGUUGGUGGA[dT][dT]	AGGCUCAUCAUCUAACCAG
CD63_4	CAGAGUUUAAUAACAACUU[dT][dT]	AAGUUGUUAUUAAACUCUG
CD81_1	CUGUGAUCAUGAUCUUCGA[dT][dT]	GACCACUGGCAACAGAGAG[dT][dT]
CD81_2	GUAUAUAACGUUUCCGGUA[dT][dT]	UCCGAUCACAAAUAGUAUG
CD81_3	GGAUGUGAAGCAGUUCUAU[dT][dT]	AGGAAACGCAGCUGUUUCG
CD9_1	CCAAGAGCAUCUUCGAGCA[dT][dT]	UGCUCGAAGAUGCUCUUGG[dT][dT]
CD9_2	GGUGAUAUUCGCCAUUGAA[dT][dT]	UUCAAUGGCGAAUAUCACC[dT][dT]
CD9_3	CUCGAAACCUUCACCGUGA[dT][dT]	UCACGGUGAAGGUUUCGAG[dT][dT]
CD93_1	GGAUUGGGCUCCAGCGAGA[dT][dT]	UCUCGCUGGAGCCCAAUCC[dT][dT]
CD93_2	GAUUCCGGCUGCUGGAUGA[dT][dT]	UCAUCCAGCAGCCGGAAUC[dT][dT]
CD93_3	GUUCGGACUGGAAACAUUU[dT][dT]	AAAUGUUUCCAGUCCGAAC[dT][dT]
CD99_1	CGAUUUAUCCGAUGCCCUU[dT][dT]	AAGGGCAUCGGAUAAAUCG[dT][dT]
CD99_2	GGAGCCAUCUCUAGCUUCA[dT][dT]	UGAAGCUAGAGAUGGCUCC[dT][dT]
CD99_3	GAGAUGGAGGCCUUCUGUU[dT][dT]	AACAGAAGGCCUCCAUCUC[dT][dT]
CDH5_1	GAUUGGAUUUGGAACCAGA[dT][dT]	UCUGGUUCCAAAUCCAAUC[dT][dT]
CDH5_2	CAUUGUGCAAGUCCACAUU[dT][dT]	AAUGUGGACUUGCACAAUG[dT][dT]
CDH5_3	GUGGAUUACGACUUCCUUA[dT][dT]	UAAGGAAGUCGUAAUCCAC[dT][dT]
CECR1_1	CCAGCUAUGUUUGGUGCCA[dT][dT]	UGGCACCAAACAUAGCUGG[dT][dT]
CECR1_2	CAGAAUCGGCCAUGGAUUU[dT][dT]	AAAUCCAUGGCCGAUUCUG[dT][dT]
CECR1_3	CCUAUGAUUUCUAUGAGGU[dT][dT]	ACCUCAUAGAAAUCAUAGG[dT][dT]
CFH_1	GCUCUUAAUCCAUUAAGGA[dT][dT]	UCCUUAAUGGAUUAAGAGC[dT][dT]
CFH_2	GAGAAUGGGUUGCUCUUAA[dT][dT]	UUAAGAGCAACCCAUUCUC[dT][dT]
CFH_3	GAGAUGAAAUCACGUACCA[dT][dT]	UGGUACGUGAUUUCAUCUC[dT][dT]
CFI_1	GCAGAAAGAAGACGGAUAA[dT][dT]	UUAUCCGUCUUCUUUCUGC[dT][dT]

CFI_2	GCGAUAAAGUCUUCUGCCA[dT][dT]	UGGCAGAAGACUUUAUCGC[dT][dT]
CFI_3	CUUACCUAUUCCAACCUAA[dT][dT]	UUAGGUUGGAAUAGGUAAG[dT][dT]
CHID1_1	GCUACGAUGUCACCAAGGU[dT][dT]	ACCUUGGUGACAUCGUAGC[dT][dT]
CHID1_2	GAGCACUUCUUCGAGUACA[dT][dT]	UGUACUCGAAGAAGUGCUC[dT][dT]
CHID1_3	GAUGUACUGGGCUAUGUCA[dT][dT]	UGACAUAGCCCAGUACAUC[dT][dT]
CHST15_1	GCUGUUACCCGACGGCGCA[dT][dT]	UGCGCCGUCGGGUAACAGC[dT][dT]
CHST15_2	GUCAAGUUCUCCGCCAUCA[dT][dT]	UGAUGGCGGAGAACUUGAC[dT][dT]
CHST15_3	GGGAUUUCUACAGGCCCUU[dT][dT]	AAGGGCCUGUAGAAAUCCC[dT][dT]
CHSY1_1	CAGAUGAUGACGUGUACAU[dT][dT]	AUGUACACGUCAUCAUCUG[dT][dT]
CHSY1_2	CUGAUGAGCAAAUACAGCA[dT][dT]	UGCUGUAUUUGCUCAUCAG[dT][dT]
CHSY1_3	GCUACAUGCUGAGCCGCAA[dT][dT]	UUGCGGCUCAGCAUGUAGC[dT][dT]
CKAP4_1	CAGUCUUUGCAAGCCACAU[dT][dT]	AUGUGGCUUGCAAAGACUG[dT][dT]
CKAP4_2	GAAGUACCCUUCAGACUAU[dT][dT]	AUAGUCUGAAGGGUACUUC[dT][dT]
CKAP4_3	GUCCAAUUUCUCAUGACCA[dT][dT]	UGGUCAUGAGAAAUUGGAC[dT][dT]
CLDN11_1	GUGGUGACCUGCGGCUACA[dT][dT]	UGUAGCCGCAGGUCACCAC[dT][dT]
CLDN11_2	CUCUAAAGCCAAAGGUCUA[dT][dT]	UAGACCUUUGGCUUUAGAG[dT][dT]
CLDN11_3	CAAAUACUGAUGGUGCAUU[dT][dT]	AAUGCACCAUCAGUAUUUG[dT][dT]
CLDN5_1	CGUUCGUUGCGCUCUUCGU[dT][dT]	ACGAAGAGCGCAACGAACG[dT][dT]
CLDN5_2	CGUCUGUGCCCGUGUCGCA[dT][dT]	UGCGACACGGGCACAGACG[dT][dT]
CLDN5_3	GUGUCAGACUGAGGAUUUC[dT][dT]	GAAAUCCUCAGUCUGACAC[dT][dT]
CLEC11A_1	CGGCGUCUCUACUACGUCU[dT][dT]	AGACGUAGUAGAGACGCCG[dT][dT]
CLEC11A_2	GCUUCCUGCUCUCGCGCGA[dT][dT]	UCGCGCGAGAGCAGGAAGC[dT][dT]
CLEC11A_3	CGCAUCCGCUCAGCCCGGA[dT][dT]	UCCGGGCUGAGCGGAUGCG[dT][dT]
CLEC14A_1	CUGAACAAGACAAUUCAGU[dT][dT]	ACUGAAUUGUCUUGUUCAG[dT][dT]
CLEC14A_2	GACUGUGAUCUGCGGGACA[dT][dT]	UGUCCCGCAGAUCACAGUC[dT][dT]
CLEC14A_3	CCACUGAGGAGCUAAAUCA[dT][dT]	UGAUUUAGCUCCUCAGUGG[dT][dT]
CLEC1A_1	GUCAAGACACCAUUUCUCA[dT][dT]	UGAGAAAUGGUGUCUUGAC[dT][dT]
CLEC1A_2	GAAUUGAAGCGUUGUGUCU[dT][dT]	AGACACAACGCUUCAAUUC[dT][dT]
CLEC1A_3	CAUAAUCAGGAAAGACUAU[dT][dT]	AUAGUCUUUCCUGAUUAUG[dT][dT]
CLSTN1_1	GACAAAGAUGGUUAUAUAA[dT][dT]	UCGAAGAUCAUGAUCACAG[dT][dT]
CLSTN1_2	GUGUUUGAGUUCAACGGCA[dT][dT]	GAUCAUAAGACAAUAGUUC
CLSTN1_3	CUGGAUACCUGUGAGGUCA[dT][dT]	AUUCAUACAAGUUUCAAAG
CLU_1	GAAAGAGGAUGCCCUAAAU[dT][dT]	AUUUAGGGCAUCCUCUUUC[dT][dT]
CLU_2	GAAAUACAACGAGCUGCUA[dT][dT]	UAGCAGCUCGUUGUAUUUC[dT][dT]
CLU_3	CCCUAAAUGAGACCAGGGA[dT][dT]	UCCCUGGUCUCAUUUAGGG[dT][dT]

CMKLR1_1	CAGUGAACAUGGUCUGGUU[dT][dT]	AACCAGACCAUGUUCACUG[dT][dT]
CMKLR1_2	CCCUUCAAGAUUAUUGUGA[dT][dT]	UCACAAUAAUCUUGAAGGG[dT][dT]
CMKLR1_3	GGUCAAUGCUCUAAGUGAA[dT][dT]	UUCACUUAGAGCAUUGACC[dT][dT]
CMTM3_1	CAUCGUGUUUGCAACUGAU[dT][dT]	AUCAGUUGCAAACACGAUG[dT][dT]
CMTM3_2	CUGUAUCUGGGCAGCAGGU[dT][dT]	ACCUGCUGCCCAGAUACAG[dT][dT]
CMTM3_3	CUUCCAAGAAUCUUUGGUU[dT][dT]	AACCAAAGAUUCUUGGAAG[dT][dT]
CNOT1_1	GAUUGACCCUCUUGCUGGA[dT][dT]	UUAUAUAACCAUCUUUGUC[dT][dT]
CNOT1_2	CUUACACACUGACUUGGAU[dT][dT]	UACCGGAAACGUUAUAUAC
CNOT1_3	GCAAGAACCUUGCAUUAGA[dT][dT]	UCCACCAACAUGAUAAGAG
CNPY4_1	GGUGUAUGUGCUGACAGUA[dT][dT]	UACUGUCAGCACAUACACC[dT][dT]
CNPY4_2	GGAAGAGACACGUGCCUUA[dT][dT]	UAAGGCACGUGUCUCUUCC[dT][dT]
CNPY4_3	CACUGAAAGGCCUAGUGCA[dT][dT]	UGCACUAGGCCUUUCAGUG[dT][dT]
COL12A1_1	CCUCUAAACACUGUAAGGA[dT][dT]	UCCUUACAGUGUUUAGAGG[dT][dT]
COL12A1_2	GUCUUUGGUGAAACAACCA[dT][dT]	UGGUUGUUUCACCAAAGAC[dT][dT]
COL12A1_3	GGAUUCAGAAGAAUGCGUU[dT][dT]	AACGCAUUCUUCUGAAUCC[dT][dT]
COL18A1_1	GUGUGCUCGCCCUGCGGUA[dT][dT]	UACCGCAGGGCGAGCACAC[dT][dT]
COL18A1_2	CAUACUUUCCUGUAUAGUU[dT][dT]	AACUAUACAGGAAAGUAUG[dT][dT]
COL18A1_3	CUGUGCAACCUCUUGGCCU[dT][dT]	AGGCCAAGAGGUUGCACAG[dT][dT]
COL1A2_1	CUGGUAAUCCUGGAGCAAA[dT][dT]	UUUGCUCCAGGAUUACCAG[dT][dT]
COL1A2_2	GGUGUAAGCGGUGGUGGUU[dT][dT]	AACCACCACCGCUUACACC[dT][dT]
COL1A2_3	GUAACCUUAUGCCUAGCAA[dT][dT]	UUGCUAGGCAUAAGGUUAC[dT][dT]
COL27A1_1	CAGAAGAUGGUGGAUGGUA[dT][dT]	UACCAUCCACCAUCUUCUG[dT][dT]
COL27A1_2	CUGCUAAGCUCCGAGGUGA[dT][dT]	UCACCUCGGAGCUUAGCAG[dT][dT]
COL27A1_3	GACAUUGGCUUCAAAGGCA[dT][dT]	UGCCUUUGAAGCCAAUGUC[dT][dT]
COL4A1_1	GUUACAGGAUUGGUGGGUA[dT][dT]	UACCCACCAAUCCUGUAAC[dT][dT]
COL4A1_2	GACAAAUGGGCUUAAGUUU[dT][dT]	AAACUUAAGCCCAUUUGUC[dT][dT]
COL4A1_3	GUUUCCAGGUUCCAAGGGA[dT][dT]	UCCCUUGGAACCUGGAAAC[dT][dT]
COL4A2_1	CUUAUGCACUGCCUAAAGA[dT][dT]	UCUUUAGGCAGUGCAUAAG[dT][dT]
COL4A2_2	CCUGUAUGGGCCUCCAGGA[dT][dT]	UCCUGGAGGCCCAUACAGG[dT][dT]
COL4A2_3	GGAGUUUGUGGAUCGGAUA[dT][dT]	UAUCCGAUCCACAAACUCC[dT][dT]
COL4A5_1	GGUAUAACUAUAGGAGAAA[dT][dT]	UUUCUCCUAUAGUUAUACC[dT][dT]
COL4A5_2	CCACAAUGCCCACAGGGAA[dT][dT]	UUCCCUGUGGGCAUUGUGG[dT][dT]
COL4A5_3	GACCAAUUGAUGUAGAGUU[dT][dT]	AACUCUACAUCAAUUGGUC[dT][dT]
COL4A6_1	CCUAGUAGGACUGAAAGGA[dT][dT]	UCCUUUCAGUCCUACUAGG[dT][dT]
COL4A6_2	CGGAUUAAUAGGACCCAAA[dT][dT]	UUUGGGUCCUAUUAAUCCG[dT][dT]

COL4A6_3	GAACCAAUUCUCAGUACAA[dT][dT]	UUGUACUGAGAAUUGGUUC[dT][dT]
COL5A1_1	GACAUCAUGUUCAAUGACU[dT][dT]	AGUCAUUGAACAUGAUGUC[dT][dT]
COL5A1_2	GAAUUGCUCUCAGCGUCCA[dT][dT]	UGGACGCUGAGAGCAAUUC[dT][dT]
COL5A1_3	CGUAUGAUGACCUCACCUA[dT][dT]	UAGGUGAGGUCAUCAUACG[dT][dT]
COL5A2_1	GGAAUUCGGCGUUGAAAUU[dT][dT]	AAUUUCAACGCCGAAUUCC[dT][dT]
COL5A2_2	CGUUGAAAUUGGGCCAGUU[dT][dT]	AACUGGCCCAAUUUCAACG[dT][dT]
COL5A2_3	CCAGUAAAUCUCCUGACAA[dT][dT]	UUGUCAGGAGAUUUACUGG[dT][dT]
COL6A1_1	GUACUACCGCUGUGACCGA[dT][dT]	UCGGUCACAGCGGUAGUAC[dT][dT]
COL6A1_2	CAGACCAGCUCAAUGUCAU[dT][dT]	AUGACAUUGAGCUGGUCUG[dT][dT]
COL6A1_3	CCGAGUACGACGUGGCCUA[dT][dT]	UAGGCCACGUCGUACUCGG[dT][dT]
COL8A1_1	CUGUAUAACGGCAGACAGA[dT][dT]	UCUGUCUGCCGUUAUACAG[dT][dT]
COL8A1_2	GCAUUUACCGCCGAGCUAA[dT][dT]	UUAGCUCGGCGGUAAAUGC[dT][dT]
COL8A1_3	GUGUCUACUACUUUGCAUA[dT][dT]	UAUGCAAAGUAGUAGACAC[dT][dT]
COLEC12_1	CAUGGAAACAUCUCGCCAA[dT][dT]	UUGGCGAGAUGUUUCCAUG[dT][dT]
COLEC12_2	GAAUCAAGAACGACUUUCA[dT][dT]	UGAAAGUCGUUCUUGAUUC[dT][dT]
COLEC12_3	CUUAUCAGUGAUUAUGGAA[dT][dT]	UUCCAUAAUCACUGAUAAG[dT][dT]
COPB2_1	CAUGGAUGUUGGCAAGUCU[dT][dT]	AGACUUGCCAACAUCCAUG[dT][dT]
COPB2_2	GAAUUGAACAUCAUUAGCU[dT][dT]	AGCUAAUGAUGUUCAAUUC[dT][dT]
COPB2_3	GACUGUAAUGCUUUCCAUU[dT][dT]	AAUGGAAAGCAUUACAGUC[dT][dT]
COPS6_1	CCAAGGAGGAGCAGUUUAA[dT][dT]	UUAAACUGCUCCUCCUUGG[dT][dT]
COPS6_2	CGUGCAACACCAUGAACCA[dT][dT]	UGGUUCAUGGUGUUGCACG[dT][dT]
COPS6_3	CACACUGGUUGGUCAACUU[dT][dT]	AAGUUGACCAACCAGUGUG[dT][dT]
COX4I1_1	GUGUGUGUACGAGCUCAUG[dT][dT]	UCCAGCAAGAGGGUCAAUC[dT][dT]
COX4I1_2	CCAUGGAUGAGAAAGUCGA[dT][dT]	UGCCGUUGAACUCAAACAC
COX4I1_3	CGAGCAAUUUCCACCUCUG[dT][dT]	AUAGAACUGCUUCACAUCC
CPD_1	CUUACAACCUUACAGUAGU[dT][dT]	ACUACUGUAAGGUUGUAAG[dT][dT]
CPD_2	CCUACAAAGACUUAUCAGA[dT][dT]	UCUGAUAAGUCUUUGUAGG[dT][dT]
CPD_3	CAAUCAAGUCUAAUAGACA[dT][dT]	UGUCUAUUAGACUUGAUUG[dT][dT]
CPQ_1	GCAAAGACCUACCCAGAUA[dT][dT]	UAUCUGGGUAGGUCUUUGC[dT][dT]
CPQ_2	GAUACUGAUUCCUUCAACA[dT][dT]	UGUUGAAGGAAUCAGUAUC[dT][dT]
CPQ_3	GAGAUCACUGGGAGCAAAU[dT][dT]	AUUUGCUCCCAGUGAUCUC[dT][dT]
CPT1A_1	CUCGUUAGGAAUAAUUGCA[dT][dT]	UGCAAUUAUUCCUAACGAG[dT][dT]
CPT1A_2	GAAGAUGGGCCUCAACGCU[dT][dT]	AGCGUUGAGGCCCAUCUUC[dT][dT]
CPT1A_3	CCUUUACGUGGUGUCUAAA[dT][dT]	UUUAGACACCACGUAAAGG[dT][dT]
CPT1C_1	CACGUUUCUGGAAUGACUU[dT][dT]	AAGUCAUUCCAGAAACGUG[dT][dT]

CPT1C_2	CAUGAUCGCUGGUUUGACA[dT][dT]	UGUCAAACCAGCGAUCAUG[dT][dT]
CPT1C_3	GGUUAUGGUGUUUCUUAUA[dT][dT]	UAUAAGAAACACCAUAACC[dT][dT]
CREG1_1	CAUUAUGGUGCUUCUGAAU[dT][dT]	AUUCAGAAGCACCAUAAUG[dT][dT]
CREG1_2	GCAAAGCAUUCGUUAUUCA[dT][dT]	UGAAUAACGAAUGCUUUGC[dT][dT]
CREG1_3	CUUCCAGCCAUAAUUGGUU[dT][dT]	AACCAAUUAUGGCUGGAAG[dT][dT]
CRELD2_1	CUGACUUAUUCGAGUGGUU[dT][dT]	AACCACUCGAAUAAGUCAG[dT][dT]
CRELD2_2	CAGAGACUGCGGCGAGUGU[dT][dT]	ACACUCGCCGCAGUCUCUG[dT][dT]
CRELD2_3	CCUGUAAUGUGCCGGACUU[dT][dT]	AAGUCCGGCACAUUACAGG[dT][dT]
CRIM1_1	GAUAUGUGCCUUUCAGCUU[dT][dT]	AAGCUGAAAGGCACAUAUC[dT][dT]
CRIM1_2	CUCAGUACUCCCUCCAUUU[dT][dT]	AAAUGGAGGGAGUACUGAG[dT][dT]
CRIM1_3	CCAUCUACAGGCAGACAAU[dT][dT]	AUUGUCUGCCUGUAGAUGG[dT][dT]
CRTAP_1	GAAGCAUCCUGAUGACGAA[dT][dT]	UUCGUCAUCAGGAUGCUUC[dT][dT]
CRTAP_2	GCUAUCUGCUCUUUGAUCA[dT][dT]	UGAUCAAAGAGCAGAUAGC[dT][dT]
CRTAP_3	CAUAGCAGAUCAUUAUGUA[dT][dT]	UACAUAAUGAUCUGCUAUG[dT][dT]
CSF2RB_1	CCCUCAUUCGCCGGGUGAA[dT][dT]	UUCACCCGGCGAAUGAGGG[dT][dT]
CSF2RB_2	CCCUCAACGUGACCAAGGA[dT][dT]	UCCUUGGUCACGUUGAGGG[dT][dT]
CSF2RB_3	GUGGCUAUACCCAUGAGCU[dT][dT]	AGCUCAUGGGUAUAGCCAC[dT][dT]
CST3_1	CCAGCAACGACAUGUACCA[dT][dT]	UGGUACAUGUCGUUGCUGG[dT][dT]
CST3_2	CCUAUCACCUCUUAUGCAC[dT][dT]	GUGCAUAAGAGGUGAUAGG[dT][dT]
CST3_3	GGCACAAUGACCUUGUCGA[dT][dT]	UCGACAAGGUCAUUGUGCC[dT][dT]
CTGF_1	CCUAUCAAGUUUGAGCUUU[dT][dT]	AAAGCUCAAACUUGAUAGG[dT][dT]
CTGF_2	GACAUUAACUCAUUAGACU[dT][dT]	AGUCUAAUGAGUUAAUGUC[dT][dT]
CTGF_3	CAAGUUAUUUAAAUCUGUU[dT][dT]	AACAGAUUUAAAUAACUUG[dT][dT]
CTHRC1_1	GAAGGACUUUGUGAAGGAA[dT][dT]	UUCCUUCACAAAGUCCUUC[dT][dT]
CTHRC1_2	CGUUCAAAUAGUGCUCUAA[dT][dT]	UUAGAGCACUAUUUGAACG[dT][dT]
CTHRC1_3	CCUCUUCCCAUUGAAGCUA[dT][dT]	UAGCUUCAAUGGGAAGAGG[dT][dT]
CTSB_1	GCAAGUAGCUUUCCACAUU[dT][dT]	AAUGUGGAAAGCUACUUGC[dT][dT]
CTSB_2	GCAUCUAUCGAGUUUGCAA[dT][dT]	UUGCAAACUCGAUAGAUGC[dT][dT]
CTSB_3	CAUGUAGGGUGCAGACCGU[dT][dT]	ACGGUCUGCACCCUACAUG[dT][dT]
CTSD_1	CUUGCUGGAUCCACCACAA[dT][dT]	UUGUGGUGGAUCCAGCAAG[dT][dT]
CTSD_2	CUGUGUUUGACCGUGACAA[dT][dT]	UUGUCACGGUCAAACACAG[dT][dT]
CTSD_3	CAGUCAGCGUCGUCAGCCU[dT][dT]	AGGCUGACGACGCUGACUG[dT][dT]
CXCL1_1	GCGGAAAGCUUGCCUCAAU[dT][dT]	AUUGAGGCAAGCUUUCCGC[dT][dT]
CXCL1_2	CGGAAAGCUUGCCUCAAUC[dT][dT]	GAUUGAGGCAAGCUUUCCG[dT][dT]
CXCL1_3	CUCACUGGUGGCUGUUCCU[dT][dT]	AGGAACAGCCACCAGUGAG[dT][dT]

CXCL11_1	GAAAUACAGUCUUCCUGAA[dT][dT]	UUCAGGAAGACUGUAUUUC[dT][dT]
CXCL11_2	CAAGCAAGGCUUAUAAUCA[dT][dT]	UGAUUAUAAGCCUUGCUUG[dT][dT]
CXCL11_3	GUUACCAUCGGAGUUUACA[dT][dT]	UGUAAACUCCGAUGGUAAC[dT][dT]
CXCL2_1	CAAUGUGACGGCAGGGAAA[dT][dT]	UUUCCCUGCCGUCACAUUG[dT][dT]
CXCL2_2	CUGUGAUAGAGGCUGAGGA[dT][dT]	UCCUCAGCCUCUAUCACAG[dT][dT]
CXCL2_3	GUUCAAUCCUGAUUCAUAU[dT][dT]	AUAUGAAUCAGGAUUGAAC[dT][dT]
CXorf36_1	CCAGCAACAGGCUACCUUU[dT][dT]	AAAGGUAGCCUGUUGCUGG[dT][dT]
CXorf36_2	GGACUUUCCUCGGUCUUGA[dT][dT]	UCAAGACCGAGGAAAGUCC[dT][dT]
CXorf36_3	CCUGCAUCGGGACAUCUAU[dT][dT]	AUAGAUGUCCCGAUGCAGG[dT][dT]
CYB5D2_1	CACUUGAAUUUACAACCAA[dT][dT]	UUGGUUGUAAAUUCAAGUG[dT][dT]
CYB5D2_2	CACUAUUCCCUUUGACCUA[dT][dT]	UAGGUCAAAGGGAAUAGUG[dT][dT]
CYB5D2_3	CAAUUGGCUUUCAUUCUAU[dT][dT]	AUAGAAUGAAAGCCAAUUG[dT][dT]
CYR61_1	GAAUUGAUUGCAGUUGGAA[dT][dT]	UUCCAACUGCAAUCAAUUC[dT][dT]
CYR61_2	CCAAGAACGUCAUGAUGAU[dT][dT]	AUCAUCAUGACGUUCUUGG[dT][dT]
CYR61_3	CCUUUACAAGGCCAGAAAU[dT][dT]	AUUUCUGGCCUUGUAAAGG[dT][dT]
CYTL1_1	CUCGUUCUGCAGGAGAGAU[dT][dT]	AUCUCUCCUGCAGAACGAG[dT][dT]
CYTL1_2	GUCAGCUACCCAGACUUAA[dT][dT]	UUAAGUCUGGGUAGCUGAC[dT][dT]
CYTL1_3	GGACUAACAAGCUUCACUU[dT][dT]	AAGUGAAGCUUGUUAGUCC[dT][dT]
CYYR1_1	CGAAGUUGGUCCUGCUCUU[dT][dT]	AAGAGCAGGACCAACUUCG[dT][dT]
CYYR1_2	CUUAUAUUGGGAAUAUCCU[dT][dT]	AGGAUAUUCCCAAUAUAAG[dT][dT]
CYYR1_3	CAGACUUGCCUCCUCCAUA[dT][dT]	UAUGGAGGAGGCAAGUCUG[dT][dT]
DCBLD1_1	CAGAUCAAUAUGGUCCAUA[dT][dT]	UAUGGACCAUAUUGAUCUG[dT][dT]
DCBLD1_2	GCUCAUUGGUUGCCAGAUU[dT][dT]	AAUCUGGCAACCAAUGAGC[dT][dT]
DCBLD1_3	CAAGUGAUAUGGCAGGUUA[dT][dT]	UAACCUGCCAUAUCACUUG[dT][dT]
DCHS1_1	GGUAUAUGUGAAGGUGUUU[dT][dT]	AAACACCUUCACAUAUACC[dT][dT]
DCHS1_2	CACUAUGUGCUGACACUGA[dT][dT]	UCAGUGUCAGCACAUAGUG[dT][dT]
DCHS1_3	GACAUCAGCUUGUAGUACA[dT][dT]	UGUACUACAAGCUGAUGUC[dT][dT]
DDOST_1	CUAUGACAAUCUCAUCAUU[dT][dT]	CAUGAGCUCGUACACACAC[dT][dT]
DDOST_2	GGAACAAUGCCCGCGUCAU[dT][dT]	AUCCAAGUCAGUGUGUAAG
DDOST_3	GGAUUGAGUUUGACGAGGA[dT][dT]	UGACCUCACAGGUAUCCAG
DHRS4L2_1	GUGGUGAUCGUGUCUUCCA[dT][dT]	UGGAAGACACGAUCACCAC[dT][dT]
DHRS4L2_2	CCUUUCUUUGGAAGCCUAA[dT][dT]	UUAGGCUUCCAAAGAAAGG[dT][dT]
DHRS4L2_3	CUUUGGAAGCCUAAUGGAU[dT][dT]	AUCCAUUAGGCUUCCAAAG[dT][dT]
DHRSX_1	GAUAUCCUGUCUCAGGAUA[dT][dT]	UAUCCUGAGACAGGAUAUC[dT][dT]
DHRSX_2	CCCAUUACGUCGCUGAGCU[dT][dT]	AGCUCAGCGACGUAAUGGG[dT][dT]

DHRSX_3	CCAAACAAGUUGUAAGCAA[dT][dT]	UUGCUUACAACUUGUUUGG[dT][dT]
DKK1_1	CACUAAACCAGCUAUCCAA[dT][dT]	UUGGAUAGCUGGUUUAGUG[dT][dT]
DKK1_2	CAAUCCUAAGGAUAUACAA[dT][dT]	UUGUAUAUCCUUAGGAUUG[dT][dT]
DKK1_3	CUGUAAACCUGUCCUGAAA[dT][dT]	UUUCAGGACAGGUUUACAG[dT][dT]
DKK3_1	CAGACAGCCGUUUGUUCUA[dT][dT]	UAGAACAAACGGCUGUCUG[dT][dT]
DKK3_2	CAUCCAUGUGCACCGAGAA[dT][dT]	UUCUCGGUGCACAUGGAUG[dT][dT]
DKK3_3	CCUACAUCUUCUUCCCAGU[dT][dT]	ACUGGGAAGAAGAUGUAGG[dT][dT]
DNAJC10_1	CCAAAUGCACAUGGCGAUU[dT][dT]	AAUCGCCAUGUGCAUUUGG[dT][dT]
DNAJC10_2	GAUAGAAUGCUUUGCCGAA[dT][dT]	UUCGGCAAAGCAUUCUAUC[dT][dT]
DNAJC10_3	GAUCAACGUGGGCAGUAUA[dT][dT]	UAUACUGCCCACGUUGAUC[dT][dT]
DNAJC13_1	GAAAUCCUGUGCCGUUCUU[dT][dT]	AAUGAUGAGAUUGUCAUAG[dT][dT]
DNAJC13_2	CUAUUACCUGAGAUUACUA[dT][dT]	UCGACUUUCUCAUCCAUGG
DNAJC13_3	CAACUAUCCACAACUCGAA[dT][dT]	UCUAAUGCAAGGUUCUUGC
DNASE1_1	CAGCCAUUGUCAGGUUCUU[dT][dT]	AAGAACCUGACAAUGGCUG[dT][dT]
DNASE1_2	CGAGAUCGACGCUCUCUAU[dT][dT]	AUAGAGAGCGUCGAUCUCG[dT][dT]
DNASE1_3	GCCAUUGUCAGGUUCUUCU[dT][dT]	AGAAGAACCUGACAAUGGC[dT][dT]
DPP7_1	GCAACAAUGUGACCGAUAU[dT][dT]	AUAUCGGUCACAUUGUUGC[dT][dT]
DPP7_2	CAGCAACAAUGUGACCGAU[dT][dT]	AUCGGUCACAUUGUUGCUG[dT][dT]
DPP7_3	GUGAUCGGCUGCUGAGUGA[dT][dT]	UCACUCAGCAGCCGAUCAC[dT][dT]
DPY19L1_1	CCAUAUAACACACCUCUUU[dT][dT]	AAAGAGGUGUGUUAUAUGG[dT][dT]
DPY19L1_2	CAGUGUUUGCGGGUGCCAU[dT][dT]	AUGGCACCCGCAAACACUG[dT][dT]
DPY19L1_3	CUAAGUCAUGUGUUGUUCA[dT][dT]	UGAACAACACAUGACUUAG[dT][dT]
DRAXIN_1	GCUGCUAUGCCAAAUUCCA[dT][dT]	UGGAAUUUGGCAUAGCAGC[dT][dT]
DRAXIN_2	GCGGUAAACUCUCCAGUGA[dT][dT]	UCACUGGAGAGUUUACCGC[dT][dT]
DRAXIN_3	GCCUCAACAACAAAUGCUU[dT][dT]	AAGCAUUUGUUGUUGAGGC[dT][dT]
DST_1	CAGAUUAGAGAGUCAGUAU[dT][dT]	AAGAACGGCACAGGAUUUC[dT][dT]
DST_2	GUGCUAAAUGGAAGGUCAU[dT][dT]	AUGACGCGGGCAUUGUUCC
DST_3	CUAACCUCUUCUAGUAUGA[dT][dT]	CAGAGGUGGAAAUUGCUCG
DYSF_1	GAAACUACCUUCACGUCCU[dT][dT]	AGGACGUGAAGGUAGUUUC[dT][dT]
DYSF_2	CUUAUGGUGCAGACGAGUU[dT][dT]	AACUCGUCUGCACCAUAAG[dT][dT]
DYSF_3	CGAACUAUGCUGCCAUGAA[dT][dT]	UUCAUGGCAGCAUAGUUCG[dT][dT]
ECE1_1	CUCUAAUGGAGUUGAUUGA[dT][dT]	UCAAUCAACUCCAUUAGAG[dT][dT]
ECE1_2	GACAGAUGCCUGCUCAACA[dT][dT]	UGUUGAGCAGGCAUCUGUC[dT][dT]
ECE1_3	CCAUGAACCCGCCUCACAA[dT][dT]	UUGUGAGGCGGGUUCAUGG[dT][dT]
ECM1_1	GAAGGAACGCCAGCUCCAU[dT][dT]	AUGGAGCUGGCGUUCCUUC[dT][dT]

ECM1_2	GACGUAACAUCUGGCGAGA[dT][dT]	UCUCGCCAGAUGUUACGUC[dT][dT]
ECM1_3	CUCACCAAGCAUAAACAUA[dT][dT]	UAUGUUUAUGCUUGGUGAG[dT][dT]
EDN1_1	GUCUUAGGCGCUGAGCUCA[dT][dT]	UGAGCUCAGCGCCUAAGAC[dT][dT]
EDN1_2	CAAACCAGGUCGGAGACCA[dT][dT]	UGGUCUCCGACCUGGUUUG[dT][dT]
EDN1_3	CUUGGAAGCCCUAGGUCCA[dT][dT]	UGGACCUAGGGCUUCCAAG[dT][dT]
EFEMP1_1	CUAUUUAUGCCAACACCAU[dT][dT]	AUGGUGUUGGCAUAAAUAG[dT][dT]
EFEMP1_2	GAUACAGGCCACAACUAUU[dT][dT]	AAUAGUUGUGGCCUGUAUC[dT][dT]
EFEMP1_3	GAGAGUUCUACCUACGACA[dT][dT]	UGUCGUAGGUAGAACUCUC[dT][dT]
EFEMP2_1	CAGCUACACGGAAUGCACA[dT][dT]	UGUGCAUUCCGUGUAGCUG[dT][dT]
EFEMP2_2	CCUACAUCCAGGUCUCUGA[dT][dT]	UCAGAGACCUGGAUGUAGG[dT][dT]
EFEMP2_3	CGCAAGAUCGGGCCCGAGU[dT][dT]	ACUCGGGCCCGAUCUUGCG[dT][dT]
EFNB2_1	GACAGAUGCACUAUUAAGA[dT][dT]	UCUUAAUAGUGCAUCUGUC[dT][dT]
EFNB2_2	CAGCUUACCUAGUCUUGUA[dT][dT]	UACAAGACUAGGUAAGCUG[dT][dT]
EFNB2_3	CUCUAGAGUUUAGUCCUUA[dT][dT]	UAAGGACUAAACUCUAGAG[dT][dT]
EGFL7_1	GCACCUACCGAACCAUCUA[dT][dT]	UAGAUGGUUCGGUAGGUGC[dT][dT]
EGFL7_2	GGUACACUCUGUGUGCCCA[dT][dT]	UGGGCACACAGAGUGUACC[dT][dT]
EGFL7_3	GUGACUGAGCGGAAGGCCA[dT][dT]	UGGCCUUCCGCUCAGUCAC[dT][dT]
EI24_1	CGAAUUAUCGGUGACCCAU[dT][dT]	AUACUGACUCUCUAAUCUG[dT][dT]
EI24_2	CUUGUUUGUGCUUAGCAAA[dT][dT]	UAGUAAUCUCAGGUAAUAG
EI24_3	CUGAGUUGCCUGCCAUCCA[dT][dT]	UCCUCGUCAAACUCAAUCC
ELOVL1_1	CCAUUCUCCUGACCUACGU[dT][dT]	ACGUAGGUCAGGAGAAUGG[dT][dT]
ELOVL1_2	CCAUUCAGCUGAUCCAGUU[dT][dT]	AACUGGAUCAGCUGAAUGG[dT][dT]
ELOVL1_3	CUUAUGUGGUCAGGACUGA[dT][dT]	UCAGUCCUGACCACAUAAG[dT][dT]
ELTD1_1	GGAAUUGAAGCCUGCUAUU[dT][dT]	AAUAGCAGGCUUCAAUUCC[dT][dT]
ELTD1_2	CUCAAACCCACCCACAUUA[dT][dT]	UAAUGUGGGUGGGUUUGAG[dT][dT]
ELTD1_3	GAAUAUUACAGAUUGUUCA[dT][dT]	UGAACAAUCUGUAAUAUUC[dT][dT]
EMCN_1	GGAUUGAAAGCCACAACCA[dT][dT]	UGGUUGUGGCUUUCAAUCC[dT][dT]
EMCN_2	GUUAUUGCUUUGAUUGUAA[dT][dT]	UUACAAUCAAAGCAAUAAC[dT][dT]
EMCN_3	GCUUAAUCUCCAGCUUCUA[dT][dT]	UAGAAGCUGGAGAUUAAGC[dT][dT]
EMILIN2_1	GGACAAAGUUCAAGUUGUU[dT][dT]	AACAACUUGAACUUUGUCC[dT][dT]
EMILIN2_2	CUCUAUCUCAGGAAAUCUU[dT][dT]	AAGAUUUCCUGAGAUAGAG[dT][dT]
EMILIN2_3	GAACUUCAGACCUAGAUAU[dT][dT]	AUAUCUAGGUCUGAAGUUC[dT][dT]
EMP1_1	GUAUCUUUGUGGUCCACAU[dT][dT]	AUGUGGACCACAAAGAUAC[dT][dT]
EMP1_2	GUCAUUAUGCGAAUCGUGA[dT][dT]	UCACGAUUCGCAUAAUGAC[dT][dT]
EMP1_3	CAUCACAUUCCAGUCUGAA[dT][dT]	UUCAGACUGGAAUGUGAUG[dT][dT]

ENG_1	CACAAGUCUUGCAGAAACA[dT][dT]	UGUUUCUGCAAGACUUGUG[dT][dT]
ENG_2	GAAAGAGCUUGUUGCGCAU[dT][dT]	AUGCGCAACAAGCUCUUUC[dT][dT]
ENG_3	CUUCUACACAGUACCCAUA[dT][dT]	UAUGGGUACUGUGUAGAAG[dT][dT]
ENTPD1_1	GUAGAAGAAUGCAGGGUUA[dT][dT]	UAACCCUGCAUUCUUCUAC[dT][dT]
ENTPD1_2	GGGUAUUGGAAACUAUCAA[dT][dT]	UUGAUAGUUUCCAAUACCC[dT][dT]
ENTPD1_3	CUAUGUCUUCCUCAUGGUU[dT][dT]	AACCAUGAGGAAGACAUAG[dT][dT]
ENTPD4_1	CGAAUUCUGAAUACCAAUU[dT][dT]	AAUUGGUAUUCAGAAUUCG[dT][dT]
ENTPD4_2	GUAUGCUUGGAUUGGCAUU[dT][dT]	AAUGCCAAUCCAAGCAUAC[dT][dT]
ENTPD4_3	CUUAAAGACUGCCUUGCAA[dT][dT]	UUGCAAGGCAGUCUUUAAG[dT][dT]
ENTPD5_1	CAGACUUGGUUUGAGGGUA[dT][dT]	UACCCUCAAACCAAGUCUG[dT][dT]
ENTPD5_2	CACUUAUAAGCUCUAUACA[dT][dT]	UGUAUAGAGCUUAUAAGUG[dT][dT]
ENTPD5_3	GCUCUUAGAGGUGGCCAAA[dT][dT]	UUUGGCCACCUCUAAGAGC[dT][dT]
EPB41L3_1	CUACUUUGGGCUUACGUAU[dT][dT]	AUACGUAAGCCCAAAGUAG[dT][dT]
EPB41L3_2	GUGUUGAGCAUCAUACAUU[dT][dT]	AAUGUAUGAUGCUCAACAC[dT][dT]
EPB41L3_3	GGAUUGACCAGAGGAAUAA[dT][dT]	UUAUUCCUCUGGUCAAUCC[dT][dT]
EPHB2_1	CCUCUAUUACUAUGAGGCU[dT][dT]	AGCCUCAUAGUAAUAGAGG[dT][dT]
EPHB2_2	GAGAGAUCUUUGUGGCCAU[dT][dT]	AUGGCCACAAAGAUCUCUC[dT][dT]
EPHB2_3	CUCAGAUGAUGAUGGAGGA[dT][dT]	UCCUCCAUCAUCAUCUGAG[dT][dT]
EPHB4_1	GAUCUGAAGUGGGUGACAU[dT][dT]	AUGUCACCCACUUCAGAUC[dT][dT]
EPHB4_2	CCCAUUUGAGCCUGUCAAU[dT][dT]	AUUGACAGGCUCAAAUGGG[dT][dT]
EPHB4_3	GGAGUUACGGGAUUGUGAU[dT][dT]	AUCACAAUCCCGUAACUCC[dT][dT]
ERMP1_1	CUUGCAUCAUGCUGUCAUA[dT][dT]	UAUGACAGCAUGAUGCAAG[dT][dT]
ERMP1_2	GACUUUGCUCGGCGUUUAU[dT][dT]	AUAAACGCCGAGCAAAGUC[dT][dT]
ERMP1_3	GCCAUAUGUCCUUCUAUGU[dT][dT]	ACAUAGAAGGACAUAUGGC[dT][dT]
ESAM_1	GUAUCCUUGGUCUACUCCA[dT][dT]	UGGAGUAGACCAAGGAUAC[dT][dT]
ESAM_2	CUCAUUGGCUAAAGGAUUU[dT][dT]	AAAUCCUUUAGCCAAUGAG[dT][dT]
ESAM_3	GAAAUUAGCUACUCACCAA[dT][dT]	UUGGUGAGUAGCUAAUUUC[dT][dT]
ESM1_1	CUUCUUCCAAUAUUCAGUA[dT][dT]	UACUGAAUAUUGGAAGAAG[dT][dT]
ESM1_2	GCAAUAUUGUGAGAGAAGA[dT][dT]	UCUUCUCUCACAAUAUUGC[dT][dT]
ESM1_3	GUAACACUGAAGAACUAAA[dT][dT]	UUUAGUUCUUCAGUGUUAC[dT][dT]
EXT1_1	GUAGGAAUGGCUUGCACCA[dT][dT]	UGGUGCAAGCCAUUCCUAC[dT][dT]
EXT1_2	GACAGAAUAUUCAAGCACA[dT][dT]	UGUGCUUGAAUAUUCUGUC[dT][dT]
EXT1_3	CCAAUUGUGAGGACAUUCU[dT][dT]	AGAAUGUCCUCACAAUUGG[dT][dT]
F11R_1	CAUCCAAGCCUACAGUUAA[dT][dT]	UUAACUGUAGGCUUGGAUG[dT][dT]
F11R_2	CGAGUAAGAAGGUGAUUUA[dT][dT]	UAAAUCACCUUCUUACUCG[dT][dT]

F11R_3	CUUCUAAGUAGACAGCAAA[dT][dT]	UUUGCUGUCUACUUAGAAG[dT][dT]
F2R_1	GAUUAGUCUCCAUCAAUAA[dT][dT]	UUAUUGAUGGAGACUAAUC[dT][dT]
F2R_2	GCUACUAUGCCUACUACUU[dT][dT]	AAGUAGUAGGCAUAGUAGC[dT][dT]
F2R_3	CUGAAUAACAGCAUAUACA[dT][dT]	UGUAUAUGCUGUUAUUCAG[dT][dT]
F2RL1_1	GAAGAAGCCUUAUUGGUAA[dT][dT]	UUACCAAUAAGGCUUCUUC[dT][dT]
F2RL1_2	GAGACAUGUUCAAUUACUU[dT][dT]	AAGUAAUUGAACAUGUCUC[dT][dT]
F2RL1_3	GUCUGUUAUUUCCUAAUCA[dT][dT]	UGAUUAGGAAAUAACAGAC[dT][dT]
FADS2_1	CCAUGAUCGUCCAUAAGAA[dT][dT]	AUGGGUCACCGAUAAUUCG[dT][dT]
FADS2_2	GACAUGAACCUGUUCAAGA[dT][dT]	AUGACCUUCCAUUUAGCAC
FADS2_3	CACGAAUACUUCUUCCUGA[dT][dT]	UUCGAGUUGUGGAUAGUUG
FAM131A_1	CUGAUGACUCCUAUGAUGA[dT][dT]	UCAUCAUAGGAGUCAUCAG[dT][dT]
FAM131A_2	CGAACAAGAGGCUCGCUUU[dT][dT]	AAAGCGAGCCUCUUGUUCG[dT][dT]
FAM131A_3	GUGACCAAGCCUACCGCCA[dT][dT]	UGGCGGUAGGCUUGGUCAC[dT][dT]
FAM171A1_1	GACUGAUGGCGUCGCCUUU[dT][dT]	AAAGGCGACGCCAUCAGUC[dT][dT]
FAM171A1_2	GCGUUUACAGGAAAGUACU[dT][dT]	AGUACUUUCCUGUAAACGC[dT][dT]
FAM171A1_3	CAUUAAAUGAGCUAUCGCA[dT][dT]	UGCGAUAGCUCAUUUAAUG[dT][dT]
FAM172A_1	GUACAGUCAUCAUCUGAUA[dT][dT]	UAUCAGAUGAUGACUGUAC[dT][dT]
FAM172A_2	GAUAUGGAGUAAUAGUACU[dT][dT]	AGUACUAUUACUCCAUAUC[dT][dT]
FAM172A_3	GUAAUAGUACUAAAUCCCA[dT][dT]	UGGGAUUUAGUACUAUUAC[dT][dT]
FAM174B_1	GUAUUCGACAUCAAAUACA[dT][dT]	UGUAUUUGAUGUCGAAUAC[dT][dT]
FAM174B_2	CCCUUUGGGCAUCCCUUUA[dT][dT]	UAAAGGGAUGCCCAAAGGG[dT][dT]
FAM174B_3	CAAUUGUUGACAAUAGUAA[dT][dT]	UUACUAUUGUCAACAAUUG[dT][dT]
FAM20C_1	GAGCCUUUAUUUACUAUUU[dT][dT]	AAAUAGUAAAUAAAGGCUC[dT][dT]
FAM20C_2	AGGAGGACGUCCUGUUCAA[dT][dT]	UUGAACAGGACGUCCUCCU[dT][dT]
FAM20C_3	CAGGUCUCAUAGGACACAU[dT][dT]	AUGUGUCCUAUGAGACCUG[dT][dT]
FAR1_1	CAGUUAAAUGUGAUUGCAA[dT][dT]	UUGCAAUCACAUUUAACUG[dT][dT]
FAR1_2	CUUUCAAGAGGAAUCCUCU[dT][dT]	AGAGGAUUCCUCUUGAAAG[dT][dT]
FAR1_3	CCAUCUUAGAUCGGAGUGU[dT][dT]	ACACUCCGAUCUAAGAUGG[dT][dT]
FAT4_1	GUGAUAGCCUUGGGCAGUU[dT][dT]	AACUGCCCAAGGCUAUCAC[dT][dT]
FAT4_2	CUACCUACCAUUUAACUGU[dT][dT]	ACAGUUAAAUGGUAGGUAG[dT][dT]
FAT4_3	CCUAUAAGGGAUGGUAAUA[dT][dT]	UAUUACCAUCCCUUAUAGG[dT][dT]
FBN1_1	CCAGAUAUGUCCUUAUGGA[dT][dT]	UCCAUAAGGACAUAUCUGG[dT][dT]
FBN1_2	GUCCAAUGAUGACAUGUGA[dT][dT]	UCACAUGUCAUCAUUGGAC[dT][dT]
FBN1_3	GCAUUAAUGGAGUCUGCAA[dT][dT]	UUGCAGACUCCAUUAAUGC[dT][dT]
FBN2_1	GCUACUACCUCAGCGAGGA[dT][dT]	UCCUCGCUGAGGUAGUAGC[dT][dT]

FBN2_2	CAAAUGGUGUGUGCAUUAA[dT][dT]	UUAAUGCACACACCAUUUG[dT][dT]
FBN2_3	CAACGAAUGUGGGUCUCAA[dT][dT]	UUGAGACCCACAUUCGUUG[dT][dT]
FGFR1_1	ACACCUGCAUUGUGGAGAA[dT][dT]	UUCUCCACAAUGCAGGUGU[dT][dT]
FGFR1_2	CACCUGCAUUGUGGAGAAU[dT][dT]	AUUCUCCACAAUGCAGGUG[dT][dT]
FGFR1_3	CCUUGUAUGUCAUCGUGGA[dT][dT]	UCCACGAUGACAUACAAGG[dT][dT]
FJX1_1	CAGGCAUGUGGGACAAGUA[dT][dT]	UACUUGUCCCACAUGCCUG[dT][dT]
FJX1_2	GGCAUGUGGGACAAGUAUA[dT][dT]	UAUACUUGUCCCACAUGCC[dT][dT]
FJX1_3	GCAUCAACCCGGAGCAGAU[dT][dT]	AUCUGCUCCGGGUUGAUGC[dT][dT]
FLRT2_1	CACAACAACCAAAUUAAUA[dT][dT]	UAUUAAUUUGGUUGUUGUG[dT][dT]
FLRT2_2	GUAUUAAAUGGGUCACAGA[dT][dT]	UCUGUGACCCAUUUAAUAC[dT][dT]
FLRT2_3	GCGUUAUCAAGGCGGACAA[dT][dT]	UUGUCCGCCUUGAUAACGC[dT][dT]
FLT1_1	CCUAACAUCACUGUUACUU[dT][dT]	AAGUAACAGUGAUGUUAGG[dT][dT]
FLT1_2	CAAGCAUCAGCAUUUGGCA[dT][dT]	UGCCAAAUGCUGAUGCUUG[dT][dT]
FLT1_3	GAUGAUGUCAGAUACGUAA[dT][dT]	UUACGUAUCUGACAUCAUC[dT][dT]
FN1_1	CAAUUACACUGAUUGCACU[dT][dT]	AGUGCAAUCAGUGUAAUUG[dT][dT]
FN1_2	CACUUAUGAGCGUCCUAAA[dT][dT]	UUUAGGACGCUCAUAAGUG[dT][dT]
FN1_3	CUGAAGAGACUUGCUUUGA[dT][dT]	UCAAAGCAAGUCUCUUCAG[dT][dT]
FNDC3B_1	GAGAUAUGCCGCCUCAGUU[dT][dT]	AACUGAGGCGGCAUAUCUC[dT][dT]
FNDC3B_2	CUCAAGUACUUGCUAGAGA[dT][dT]	UCUCUAGCAAGUACUUGAG[dT][dT]
FNDC3B_3	GACUACAGGUUCCGCGUAU[dT][dT]	AUACGCGGAACCUGUAGUC[dT][dT]
FSTL1_1	GUGAAAUCCUAGACAAGUA[dT][dT]	UACUUGUCUAGGAUUUCAC[dT][dT]
FSTL1_2	GAUCUAAUGAGGAGGCACA[dT][dT]	UGUGCCUCCUCAUUAGAUC[dT][dT]
FSTL1_3	CAGUGAAUUCCUGAAGUUU[dT][dT]	AAACUUCAGGAAUUCACUG[dT][dT]
FSTL5_1	GAUAUUACACCAAAGCUUU[dT][dT]	AAAGCUUUGGUGUAAUAUC[dT][dT]
FSTL5_2	CAAUAUAACAUCUACGGUA[dT][dT]	UACCGUAGAUGUUAUAUUG[dT][dT]
FSTL5_3	GAAUGAAGCAGGAGUGGAU[dT][dT]	AUCCACUCCUGCUUCAUUC[dT][dT]
FUCA2_1	CUAUCAGGUGUCUAUAAUU[dT][dT]	AAUUAUAGACACCUGAUAG[dT][dT]
FUCA2_2	CAAGUUAUGUCUAAGGCUA[dT][dT]	UAGCCUUAGACAUAACUUG[dT][dT]
FUCA2_3	CUGACUAAUGUGAUCUAAA[dT][dT]	UUUAGAUCACAUUAGUCAG[dT][dT]
FZD4_1	CACAGUUCCUGCAACGUGU[dT][dT]	UUCUUAUGGACGAUCAUGG[dT][dT]
FZD4_2	GUGAAUCUCAGUUUGAACA[dT][dT]	UUUGCUAAGCACAAACAAG
FZD4_3	CAGGUUUGGUGGCCUUGUU[dT][dT]	UCAUACUAGAAGAGGUUAG
GALNT1_1	CGUCAUUCCUAAAGAGGAU[dT][dT]	AUCCUCUUUAGGAAUGACG[dT][dT]
GALNT1_2	GGUUCAACUGGAAGCUCAA[dT][dT]	UUGAGCUUCCAGUUGAACC[dT][dT]
GALNT1_3	CGAAUCAGUGUCUAGAUAA[dT][dT]	UUAUCUAGACACUGAUUCG[dT][dT]

GALNT7_1	GGACUUAGAGUCUAUUAGA[dT][dT]	UCUAAUAGACUCUAAGUCC[dT][dT]
GALNT7_2	CAUUUGCACUGUGCCGCUU[dT][dT]	AAGCGGCACAGUGCAAAUG[dT][dT]
GALNT7_3	CAAAUGGAGGCUUUGUUGA[dT][dT]	UCAACAAAGCCUCCAUUUG[dT][dT]
GANAB_1	CAUUAGUGCUGAGAGAUAU[dT][dT]	ACACGUUGCAGGAACUGUG[dT][dT]
GANAB_2	GGUAUGACAUUCAAAGCUA[dT][dT]	UCUUGAACAGGUUCAUGUC
GANAB_3	CAUAUCGCCUCUACAAUUU[dT][dT]	UGGAUGGCAGGCAACUCAG
GAS6_1	CUCUGGACGUCGGGACUGA[dT][dT]	UCAGUCCCGACGUCCAGAG[dT][dT]
GAS6_2	CUAUCACUCCACGAAGAAA[dT][dT]	UUUCUUCGUGGAGUGAUAG[dT][dT]
GAS6_3	GGUAUUCCCUUCCAUGAGA[dT][dT]	UCUCAUGGAAGGGAAUACC[dT][dT]
GBP2_1	CCAACUUCACUAUGUGACA[dT][dT]	UGUCACAUAGUGAAGUUGG[dT][dT]
GBP2_2	GAAGCAAGGCGAGAUGACU[dT][dT]	AGUCAUCUCGCCUUGCUUC[dT][dT]
GBP2_3	GCAACUUCAGAGUGUCAAA[dT][dT]	UUUGACACUCUGAAGUUGC[dT][dT]
GDF11_1	CUGCAGAUCUUGCGACUAA[dT][dT]	UUAGUCGCAAGAUCUGCAG[dT][dT]
GDF11_2	GCAUCGAGAUCAACGCCUU[dT][dT]	AAGGCGUUGAUCUCGAUGC[dT][dT]
GDF11_3	GCCAUUGGCAGAGCAUCGA[dT][dT]	UCGAUGCUCUGCCAAUGGC[dT][dT]
GDF15_1	GACCAACUGCUGGCAGAAU[dT][dT]	AUUCUGCCAGCAGUUGGUC[dT][dT]
GDF15_2	CAGAGCUGGGAAGAUUCGA[dT][dT]	UCGAAUCUUCCCAGCUCUG[dT][dT]
GDF15_3	GCUACGAGGACCUGCUAAC[dT][dT]	GUUAGCAGGUCCUCGUAGC[dT][dT]
GDF3_1	CGAGACUUAUGCUACGUAA[dT][dT]	UUACGUAGCAUAAGUCUCG[dT][dT]
GDF3_2	GAGACUUAUGCUACGUAAA[dT][dT]	UUUACGUAGCAUAAGUCUC[dT][dT]
GDF3_3	CCAAUUAUGCUUUCAUGCA[dT][dT]	UGCAUGAAAGCAUAAUUGG[dT][dT]
GGH_1	GAAAGACUAUGAAAUACUU[dT][dT]	AAGUAUUUCAUAGUCUUUC[dT][dT]
GGH_2	CUAUAUUGCUGCGUCCUAU[dT][dT]	AUAGGACGCAGCAAUAUAG[dT][dT]
GGH_3	GGAAGAAUUUGGAUGGCAU[dT][dT]	AUGCCAUCCAAAUUCUUCC[dT][dT]
GIMAP1_1	CAGAGACGGUUCUUCUCCA[dT][dT]	UGGAGAAGAACCGUCUCUG[dT][dT]
GIMAP1_2	GCACGAUUACGUGAGCAAC[dT][dT]	GUUGCUCACGUAAUCGUGC[dT][dT]
GIMAP1_3	CCAUUACUCCAACGAGGUG[dT][dT]	CACCUCGUUGGAGUAAUGG[dT][dT]
GIMAP2_1	CGAAUCUGUGCCUUUAAUA[dT][dT]	UAUUAAAGGCACAGAUUCG[dT][dT]
GIMAP2_2	GAGAACAGUUAUUAGACUA[dT][dT]	UAGUCUAAUAACUGUUCUC[dT][dT]
GIMAP2_3	CACAGUAAUUUCCCUGUAA[dT][dT]	UUACAGGGAAAUUACUGUG[dT][dT]
GJA1_1	GAGAUGAGCAGUCUGCCUU[dT][dT]	AAGGCAGACUGCUCAUCUC[dT][dT]
GJA1_2	CUAUGUCUCCUCCUGGGUA[dT][dT]	UACCCAGGAGGAGACAUAG[dT][dT]
GJA1_3	GAAAGUGCACCAGGUGUUA[dT][dT]	UAACACCUGGUGCACUUUC[dT][dT]
GLB1L_1	GGUUUAACUUGGGCCGGUA[dT][dT]	UACCGGCCCAAGUUAAACC[dT][dT]
GLB1L_2	CAAGAUAUAUCCAUGGCUU[dT][dT]	AAGCCAUGGAUAUAUCUUG[dT][dT]

GLB1L_3	CAGAAUCACUCCACACGGU[dT][dT]	ACCGUGUGGAGUGAUUCUG[dT][dT]
GLG1_1	GCAAAUCUACUAUAACAGA[dT][dT]	UCUGUUAUAGUAGAUUUGC[dT][dT]
GLG1_2	GUUAGAAUCAGAGGAUAUU[dT][dT]	AAUAUCCUCUGAUUCUAAC[dT][dT]
GLG1_3	GUUACAGCCCGAGUGCAAA[dT][dT]	UUUGCACUCGGGCUGUAAC[dT][dT]
GNAS_1	CUGAUUGACUGUGCCCAGU[dT][dT]	ACUGGGCACAGUCAAUCAG[dT][dT]
GNAS_2	CUUUGACUUCCCUCCCGAA[dT][dT]	UUCGGGAGGGAAGUCAAAG[dT][dT]
GNAS_3	CUGACUAUGUGCCGAGCGA[dT][dT]	UCGCUCGGCACAUAGUCAG[dT][dT]
GOLGB1_1	GUCUAUGAGUUCCUUGCAA[dT][dT]	AUAUCUCUCAGCACUAAUG[dT][dT]
GOLGB1_2	CAGCUAAAGAGUGUAUGGA[dT][dT]	UGUUCAAACUGAGAUUCAC
GOLGB1_3	GAGAAGACCUCUCAUAUUU[dT][dT]	UCAGGAAGAAGUAUUCGUG
GOLM1_1	CAGUUAAAGACCCUGCAGA[dT][dT]	UCUGCAGGGUCUUUAACUG[dT][dT]
GOLM1_2	GAGAAGAUGACUACAACAU[dT][dT]	AUGUUGUAGUCAUCUUCUC[dT][dT]
GOLM1_3	GGAACAUAGUUCUCCUUCA[dT][dT]	UGAAGGAGAACUAUGUUCC[dT][dT]
GPC1_1	CUGACUAUUGCCGAAAUGU[dT][dT]	ACAUUUCGGCAAUAGUCAG[dT][dT]
GPC1_2	CCUCUUCCUGGCCCUUACA[dT][dT]	UGUAAGGGCCAGGAAGAGG[dT][dT]
GPC1_3	GCCUACAGAGGAGGCCUCA[dT][dT]	UGAGGCCUCCUCUGUAGGC[dT][dT]
GPR116_1	CACCAUUGCAGUUGCCUUA[dT][dT]	UAAGGCAACUGCAAUGGUG[dT][dT]
GPR116_2	GAAGCUAUGCCGGUUCUCA[dT][dT]	UGAGAACCGGCAUAGCUUC[dT][dT]
GPR116_3	GAAUACUCCUGGAUAUUAU[dT][dT]	AUAAUAUCCAGGAGUAUUC[dT][dT]
GPR124_1	CGACUAAACAUAUCUGGAA[dT][dT]	UUCCAGAUAUGUUUAGUCG[dT][dT]
GPR124_2	CAUUGGAGGCCUACCUCAU[dT][dT]	AUGAGGUAGGCCUCCAAUG[dT][dT]
GPR124_3	CUCAUCACCUGGAUCUAUU[dT][dT]	AAUAGAUCCAGGUGAUGAG[dT][dT]
GPR126_1	CAGAUAACAUUUAACGACU[dT][dT]	AGUCGUUAAAUGUUAUCUG[dT][dT]
GPR126_2	GAACUUAACCUCAGCCAAU[dT][dT]	AUUGGCUGAGGUUAAGUUC[dT][dT]
GPR126_3	GUGAUGAAUUCUGUUGGAU[dT][dT]	AUCCAACAGAAUUCAUCAC[dT][dT]
GPR176_1	GACAUAACGGGAGCUGGAU[dT][dT]	AUCCAGCUCCCGUUAUGUC[dT][dT]
GPR176_2	CUGUGUUUGCAGUAACCAA[dT][dT]	UUGGUUACUGCAAACACAG[dT][dT]
GPR176_3	CUUCCAUAUUCCCACCAGA[dT][dT]	UCUGGUGGGAAUAUGGAAG[dT][dT]
GPR56_1	CUCCGAAGAGGCCCUCACA[dT][dT]	UGUGAGGGCCUCUUCGGAG[dT][dT]
GPR56_2	GAAUGUGACUCUGCAAUGU[dT][dT]	ACAUUGCAGAGUCACAUUC[dT][dT]
GPR56_3	CCUUGGAUCUUGAGGGUCU[dT][dT]	AGACCCUCAAGAUCCAAGG[dT][dT]
GPX3_1	GACUUUGUUCAGGAAGAAA[dT][dT]	UUUCUUCCUGAACAAAGUC[dT][dT]
GPX3_2	GGACUUUGUUCAGGAAGAA[dT][dT]	UUCUUCCUGAACAAAGUCC[dT][dT]
GPX3_3	CAGAGAUCCUUCCUACCCU[dT][dT]	AGGGUAGGAAGGAUCUCUG[dT][dT]
GPX7_1	GCAACAAGGAGAUUGAGAG[dT][dT]	CUCUCAAUCUCCUUGUUGC[dT][dT]

GPX7_2	GGAAGCUCAUCCUACUGAA[dT][dT]	UUCAGUAGGAUGAGCUUCC[dT][dT]
GPX7_3	GAACUUCUGGAAGUACCUA[dT][dT]	UAGGUACUUCCAGAAGUUC[dT][dT]
GPX8_1	GGCUGAAGGAACUGCACAA[dT][dT]	UUGUGCAGUUCCUUCAGCC[dT][dT]
GPX8_2	CCAAGCAAGGAAGUAGAAU[dT][dT]	AUUCUACUUCCUUGCUUGG[dT][dT]
GPX8_3	GCAUUUAGAUUUCUUGUUG[dT][dT]	CAACAAGAAAUCUAAAUGC[dT][dT]
GRN_1	GUAAGUGCCUCUCCAAGGA[dT][dT]	UCCUUGGAGAGGCACUUAC[dT][dT]
GRN_2	GUGACCUGAUCCAGAGUAA[dT][dT]	UUACUCUGGAUCAGGUCAC[dT][dT]
GRN_3	GCUACCACGGACCUCCUCA[dT][dT]	UGAGGAGGUCCGUGGUAGC[dT][dT]
GSN_1	CUGUUGAGGUAUUGCCUAA[dT][dT]	UUAGGCAAUACCUCAACAG[dT][dT]
GSN_2	GCUACAUCAUUCUGUACAA[dT][dT]	UUGUACAGAAUGAUGUAGC[dT][dT]
GSN_3	CACAUAUGGACAGUUCUAU[dT][dT]	AUAGAACUGUCCAUAUGUG[dT][dT]
HEG1_1	CAAUGCAUGUUGCCACCGU[dT][dT]	ACGGUGGCAACAUGCAUUG[dT][dT]
HEG1_2	GUCAACUCCUGCAAGUCCU[dT][dT]	AGGACUUGCAGGAGUUGAC[dT][dT]
HEG1_3	GACAGUAUAACCCGUCUUU[dT][dT]	AAAGACGGGUUAUACUGUC[dT][dT]
HERC4_1	GUACACAUGUGGAUGUAAU[dT][dT]	AUUACAUCCACAUGUGUAC[dT][dT]
HERC4_2	GGAGAUAGCCAAUGAGAUA[dT][dT]	UAUCUCAUUGGCUAUCUCC[dT][dT]
HERC4_3	CACGAAUUACCAUUGGAAA[dT][dT]	UUUCCAAUGGUAAUUCGUG[dT][dT]
HHIP_1	CUGUUACCAACAACACAGA[dT][dT]	UCUGUGUUGUUGGUAACAG[dT][dT]
HHIP_2	CAUCUAACUACUUGGACCA[dT][dT]	UGGUCCAAGUAGUUAGAUG[dT][dT]
HHIP_3	CAGAUCAUCAGCCAGAAUU[dT][dT]	AAUUCUGGCUGAUGAUCUG[dT][dT]
HMOX2_1	GCACUUUACUUCACAUACU[dT][dT]	AGUAUGUGAAGUAAAGUGC[dT][dT]
HMOX2_2	GAGUAUAACAUGCAGAUAU[dT][dT]	AUAUCUGCAUGUUAUACUC[dT][dT]
HMOX2_3	CUGGGUUUAAGAAAGGCAA[dT][dT]	UUGCCUUUCUUAAACCCAG[dT][dT]
HPSE_1	CUAAGAAAGUCCACCUUCA[dT][dT]	UGAAGGUGGACUUUCUUAG[dT][dT]
HPSE_2	GCAAUGAACCUAACAGUUU[dT][dT]	AAACUGUUAGGUUCAUUGC[dT][dT]
HPSE_3	GAAGCUUCGAGUAUACCUU[dT][dT]	AAGGUAUACUCGAAGCUUC[dT][dT]
HSD17B11_1	CGAAGAAUAACCAUGGCCA[dT][dT]	UGGCCAUGGUUAUUCUUCG[dT][dT]
HSD17B11_2	GAAUAACCAUGGCCAUAUU[dT][dT]	AAUAUGGCCAUGGUUAUUC[dT][dT]
HSD17B11_3	GCACAUUUCUGGACUACAA[dT][dT]	UUGUAGUCCAGAAAUGUGC[dT][dT]
HSP90AB1_1	CUCAUGUCCCUCAUCAUCA[dT][dT]	UGAUGAUGAGGGACAUGAG[dT][dT]
HSP90AB1_2	CAUUCUCAGUUCAUAGGCU[dT][dT]	AGCCUAUGAACUGAGAAUG[dT][dT]
HSP90AB1_3	GCAUCUAUCGCAUGAUCAA[dT][dT]	UUGAUCAUGCGAUAGAUGC[dT][dT]
HSPG2_1	GACAUGUCUGAUGAGCUCA[dT][dT]	UGAGCUCAUCAGACAUGUC[dT][dT]
HSPG2_2	GUAACAACAUCAUCCUAGA[dT][dT]	UCUAGGAUGAUGUUGUUAC[dT][dT]
HSPG2_3	GUUACAACGUGCGCUACGA[dT][dT]	UCGUAGCGCACGUUGUAAC[dT][dT]

HTRA1_1	CCAAUGAUGUCAGCGACGU[dT][dT]	ACGUCGCUGACAUCAUUGG[dT][dT]
HTRA1_2	GAUGUCAGCGACGUCAUUA[dT][dT]	UAAUGACGUCGCUGACAUC[dT][dT]
HTRA1_3	GAAGUAUAUUGGUAUCCGA[dT][dT]	UCGGAUACCAAUAUACUUC[dT][dT]
HTRA3_1	GUGACAACGGGCAUCGUCA[dT][dT]	UGACGAUGCCCGUUGUCAC[dT][dT]
HTRA3_2	CAAGUUCUCCGGAUAUUCA[dT][dT]	UGAAUAUCCGGAGAACUUG[dT][dT]
HTRA3_3	GGUCAACGGGCGUCCUCUA[dT][dT]	UAGAGGACGCCCGUUGACC[dT][dT]
HUWE1_1	CUAGUAACACACUACAUUA[dT][dT]	UUGCAAGGAACUCAUAGAC[dT][dT]
HUWE1_2	GAGUUAGAGCUGCUUAUAU[dT][dT]	UAGCUUUGAAUGUCAUACC
HUWE1_3	CUACAAUGGUGCAGGUUAA[dT][dT]	AACAAGGCCACCAAACCUG
ICAM2_1	GACCUCUCUAAAUAAGAUU[dT][dT]	AAUCUUAUUUAGAGAGGUC[dT][dT]
ICAM2_2	CUGACACUGCAACCCACUU[dT][dT]	AAGUGGGUUGCAGUGUCAG[dT][dT]
ICAM2_3	CAGAUGGUCAUCAUAGUCA[dT][dT]	UGACUAUGAUGACCAUCUG[dT][dT]
IDE_1	CAGAUUAUCUCGGAACCUU[dT][dT]	AAGGUUCCGAGAUAAUCUG[dT][dT]
IDE_2	GCUAUCUGCUGAGUGUGCU[dT][dT]	AGCACACUCAGCAGAUAGC[dT][dT]
IDE_3	GGCAUUAGCAAUUCGUCGA[dT][dT]	UCGACGAAUUGCUAAUGCC[dT][dT]
IFI27_1	CCAGGAUUGCUACAGUUGU[dT][dT]	ACAACUGUAGCAAUCCUGG[dT][dT]
IFI27_2	CAGUCACUGGGAGCAACUG[dT][dT]	CAGUUGCUCCCAGUGACUG[dT][dT]
IFI27_3	CUCCAUUGGGUCUGCCAUU[dT][dT]	AAUGGCAGACCCAAUGGAG[dT][dT]
IFITM2_1	CACAUUGUGCAAACCUUCU[dT][dT]	AGAAGGUUUGCACAAUGUG[dT][dT]
IFITM2_2	CCACGUACUCUAUCUUCCA[dT][dT]	UGGAAGAUAGAGUACGUGG[dT][dT]
IFITM2_3	CCACUUACUCCACCUUCCA[dT][dT]	UGGAAGGUGGAGUAAGUGG[dT][dT]
IFITM3_1	CACACUGUCCAAACCUUCU[dT][dT]	AGAAGGUUUGGACAGUGUG[dT][dT]
IFITM3_2	CCACGUACUCCAACUUCCA[dT][dT]	UGGAAGUUGGAGUACGUGG[dT][dT]
IGF2R_1	CAGGAAACCUUGGACGGCU[dT][dT]	UAAUGUAGUGUGUUACUAG[dT][dT]
IGF2R_2	CCUACAACUUCCGGUGGUA[dT][dT]	UCCAUACACUCUUUAGCUG
IGF2R_3	CGGAAUACGCCUGUGUUAA[dT][dT]	AAAUUGUAGAGGCGAUAUG
IGFBP1_1	GAAGGAGCCCUGCCGAAUA[dT][dT]	UAUUCGGCAGGGCUCCUUC[dT][dT]
IGFBP1_2	GGCUGCGGCUGUUGCCCGA[dT][dT]	UCGGGCAACAGCCGCAGCC[dT][dT]
IGFBP1_3	GUCGUAGAGAGUUUAGCCA[dT][dT]	UGGCUAAACUCUCUACGAC[dT][dT]
IGFBP2_1	GAGUGUCAUCUCUUCUACA[dT][dT]	UGUAGAAGAGAUGACACUC[dT][dT]
IGFBP2_2	CGAGUGUCAUCUCUUCUAC[dT][dT]	GUAGAAGAGAUGACACUCG[dT][dT]
IGFBP2_3	CAAUGGCGAUGACCACUCA[dT][dT]	UGAGUGGUCAUCGCCAUUG[dT][dT]
IGFBP3_1	GCUUUAUCGAGAAUAGGAA[dT][dT]	UUCCUAUUCUCGAUAAAGC[dT][dT]
IGFBP3_2	GGCUUUAUCGAGAAUAGGA[dT][dT]	UCCUAUUCUCGAUAAAGCC[dT][dT]
IGFBP3_3	CUUUAUCGAGAAUAGGAAA[dT][dT]	UUUCCUAUUCUCGAUAAAG[dT][dT]

IGFBP4_1	GCAGAAGCACUUCGCCAAA[dT][dT]	UUUGGCGAAGUGCUUCUGC[dT][dT]
IGFBP4_2	CUCCUAUAAGGAGAGUUCA[dT][dT]	UGAACUCUCCUUAUAGGAG[dT][dT]
IGFBP4_3	GAGACUGGCACUUAGCCCA[dT][dT]	UGGGCUAAGUGCCAGUCUC[dT][dT]
IGFBP6_1	GUCUCCAGAUGGCAAUGGA[dT][dT]	UCCAUUGCCAUCUGGAGAC[dT][dT]
IGFBP6_2	GAGAUGGGCCCAUGCCGUA[dT][dT]	UACGGCAUGGGCCCAUCUC[dT][dT]
IGFBP6_3	CAUCGAGGCUUCUACCGGA[dT][dT]	UCCGGUAGAAGCCUCGAUG[dT][dT]
IGFBP7_1	GUGAAGGUGCCGAGCUAUA[dT][dT]	UAUAGCUCGGCACCUUCAC[dT][dT]
IGFBP7_2	GAGGUCAUCGGAAUCCCGA[dT][dT]	UCGGGAUUCCGAUGACCUC[dT][dT]
IGFBP7_3	GAAUAUUAUUAGUCUGCAU[dT][dT]	AUGCAGACUAAUAAUAUUC[dT][dT]
IL13RA1_1	CAGUGUAGCACCAAUGAGA[dT][dT]	UCUCAUUGGUGCUACACUG[dT][dT]
IL13RA1_2	CAAGAAAUGAGUAUAGGUA[dT][dT]	UACCUAUACUCAUUUCUUG[dT][dT]
IL13RA1_3	GAAACUACUGCACCAUUUA[dT][dT]	UAAAUGGUGCAGUAGUUUC[dT][dT]
IL17D_1	GCGACUAUAUACCUACUUU[dT][dT]	AAAGUAGGUAUAUAGUCGC[dT][dT]
IL17D_2	GAGAGCUACUCUGUUACAU[dT][dT]	AUGUAACAGAGUAGCUCUC[dT][dT]
IL17D_3	CUCGUAAGCAGCUUCAUCU[dT][dT]	AGAUGAAGCUGCUUACGAG[dT][dT]
IL18BP_1	CUGCUCUACUGCUCAGAAA[dT][dT]	UUUCUGAGCAGUAGAGCAG[dT][dT]
IL18BP_2	GGCAAUGGUUCCUUCAUUG[dT][dT]	CAAUGAAGGAACCAUUGCC[dT][dT]
IL18BP_3	CUGUUGAUGCCUUAGCCUU[dT][dT]	AAGGCUAAGGCAUCAACAG[dT][dT]
IL1RL1_1	GAAUGUCACCAUAUAUAAA[dT][dT]	AGCCGUCCAAGGUUUCCUG[dT][dT]
IL1RL1_2	CCUAACUUGCUCUGCUUGU[dT][dT]	AUAUAAGCAGCUCUAACUC
IL1RL1_3	GAUUGCAAUGUUCCAGAUU[dT][dT]	AAAUAUGAGAGGUCUUCUC
IL32_1	GAUUACGGUGCCGAGGCAA[dT][dT]	UUGCCUCGGCACCGUAAUC[dT][dT]
IL32_2	GGCCCUGGUCCAUGCAGUG[dT][dT]	CACUGCAUGGACCAGGGCC[dT][dT]
IL32_3	GGAUGUUGAGGAUCCCGCA[dT][dT]	UGCGGGAUCCUCAACAUCC[dT][dT]
IL4R_1	CCCACACGUGUAUCCCUGA[dT][dT]	UCAGGGAUACACGUGUGGG[dT][dT]
IL4R_2	CUGUGGAAGGGCUCCUUCA[dT][dT]	UGAAGGAGCCCUUCCACAG[dT][dT]
IL4R_3	CAAUUACCUGUAUAAUCAU[dT][dT]	AUGAUUAUACAGGUAAUUG[dT][dT]
IL6_1	CAUGUAACAAGAGUAACAU[dT][dT]	AUGUUACUCUUGUUACAUG[dT][dT]
IL6_2	CUCACCUCUUCAGAACGAA[dT][dT]	UUCGUUCUGAAGAGGUGAG[dT][dT]
IL6_3	CUUCCAAUCUGGAUUCAAU[dT][dT]	AUUGAAUCCAGAUUGGAAG[dT][dT]
IL6ST_1	CUUUACAGAUAUAGCUUCA[dT][dT]	UGAAGCUAUAUCUGUAAAG[dT][dT]
IL6ST_2	CUUGAGUGGUGUGUGUUAU[dT][dT]	AUAACACACACCACUCAAG[dT][dT]
IL6ST_3	GAUCAUAUUUCACAAUCCU[dT][dT]	AGGAUUGUGAAAUAUGAUC[dT][dT]
IL8_1	CUGCGCCAACACAGAAAUU[dT][dT]	AAUUUCUGUGUUGGCGCAG[dT][dT]
IL8_2	UGCAUAAAGACAUACUCCA[dT][dT]	UGGAGUAUGUCUUUAUGCA[dT][dT]

IL8_3	GACAUACUCCAAACCUUUC[dT][dT]	GAAAGGUUUGGAGUAUGUC[dT][dT]
INHBA_1	GAAGUCUGGCUCUUCCUAA[dT][dT]	UUAGGAAGAGCCAGACUUC[dT][dT]
INHBA_2	GCUUCUGAACGCGAUCAGA[dT][dT]	UCUGAUCGCGUUCAGAAGC[dT][dT]
INHBA_3	CGAUGUCACCCAGCCGGUA[dT][dT]	UACCGGCUGGGUGACAUCG[dT][dT]
ITGA10_1	CAACAUACAUGGAUGUUGU[dT][dT]	ACAACAUCCAUGUAUGUUG[dT][dT]
ITGA10_2	GGAUUGUGGCCCUGACAAU[dT][dT]	AUUGUCAGGGCCACAAUCC[dT][dT]
ITGA10_3	CUUCAAGAGACUUGCAUAA[dT][dT]	UUAUGCAAGUCUCUUGAAG[dT][dT]
ITGA2_1	CUGGUUACUGGUUGGUUCA[dT][dT]	UGAACCAACCAGUAACCAG[dT][dT]
ITGA2_2	GUCACAAGUGGGAUUCAGU[dT][dT]	ACUGAAUCCCACUUGUGAC[dT][dT]
ITGA2_3	CAGUAAGCAUGGCAACUGU[dT][dT]	ACAGUUGCCAUGCUUACUG[dT][dT]
ITGA5_1	GAGUACAAGUCCUUGCAGU[dT][dT]	ACUGCAAGGACUUGUACUC[dT][dT]
ITGA5_2	CCAUGAUGAGUUUGGCCGA[dT][dT]	UCGGCCAAACUCAUCAUGG[dT][dT]
ITGA5_3	GUGGUUUCCUUUCGGCUCU[dT][dT]	AGAGCCGAAAGGAAACCAC[dT][dT]
ITGA6_1	GUGACAUGUGCUCACCGAU[dT][dT]	AUCGGUGAGCACAUGUCAC[dT][dT]
ITGA6_2	GUUUCGAAACCAAGGUUCU[dT][dT]	AGAACCUUGGUUUCGAAAC[dT][dT]
ITGA6_3	CUCUUAACUGUAGCGUGAA[dT][dT]	UUCACGCUACAGUUAAGAG[dT][dT]
ITGAV_1	CCAAUUGAAUUUGAUGCAA[dT][dT]	UUGCAUCAAAUUCAAUUGG[dT][dT]
ITGAV_2	GACAUUGAUUGUUAAGGCU[dT][dT]	AGCCUUAACAAUCAAUGUC[dT][dT]
ITGAV_3	CUUUACUGCUGAUAGUGCU[dT][dT]	AGCACUAUCAGCAGUAAAG[dT][dT]
ITGB1_1	CUUACUCAAUGAAAGACGA[dT][dT]	UCGUCUUUCAUUGAGUAAG[dT][dT]
ITGB1_2	GCUUUACGGAGGAAGUAGA[dT][dT]	UCUACUUCCUCCGUAAAGC[dT][dT]
ITGB1_3	CUGUUCUUUGGAUACUAGU[dT][dT]	ACUAGUAUCCAAAGAACAG[dT][dT]
ITGBL1_1	GUAACCAAAUGUGCAAGAA[dT][dT]	UUCUUGCACAUUUGGUUAC[dT][dT]
ITGBL1_2	GACUUGUGUAUGUGGUGAA[dT][dT]	UUCACCACAUACACAAGUC[dT][dT]
ITGBL1_3	CUAUGACCGAUAUUCUGAU[dT][dT]	AUCAGAAUAUCGGUCAUAG[dT][dT]
ITM2B_1	CAGAAACCUACUGGAGUUA[dT][dT]	UUUAUAUAUGGUGACAUUC[dT][dT]
ITM2B_2	GAAACUUACAGCCUAUUUA[dT][dT]	UACCACCGGAAGUUGUAGG
ITM2B_3	GGUUAUUACUGAUCGCAUU[dT][dT]	UUAACCUGCACCAUUGUAG
ITM2B_4	CCAACAUUGUUCAUGACUU[dT][dT]	AAGUCAUGAACAAUGUUGG
ITPR2_1	GUAACAAUGAGUCCCGCAA[dT][dT]	UAACUCCAGUAGGUUUCUG[dT][dT]
ITPR2_1	GCUUAAUCCUGAUUAUCGA[dT][dT]	UCGAUAAUCAGGAUUAAGC[dT][dT]
ITPR2_2	GUAACAAUGAGUCCCGCAA[dT][dT]	UUGCGGGACUCAUUGUUAC[dT][dT]
ITPR2_2	GAAACUAUGUUUGCGGGAA[dT][dT]	ACAAGCAGAGCAAGUUAGG
ITPR2_3	CAUCGAAAGAUGAGCCCUU[dT][dT]	AAGGGCUCAUCUUUCGAUG[dT][dT]
ITPR2_3	GCUUAAUCCUGAUUAUCGA[dT][dT]	UUAACACAGGCGUAUUCCG

ITPR3_1	GCAAUUACGAGCUCAGCGA[dT][dT]	UCGCUGAGCUCGUAAUUGC[dT][dT]
ITPR3_2	CCAAGAACGAGAAGAUCUU[dT][dT]	AAGAUCUUCUCGUUCUUGG[dT][dT]
ITPR3_3	GCUAUAAGGCCAUGGUCAU[dT][dT]	AUGACCAUGGCCUUAUAGC[dT][dT]
JAM3_1	GAACUGUCUUGCAUCAUUA[dT][dT]	UAAUGAUGCAAGACAGUUC[dT][dT]
JAM3_2	CUGAACAUUGGCGGAAUUA[dT][dT]	UAAUUCCGCCAAUGUUCAG[dT][dT]
JAM3_3	CAUCGUUUGUGAUCUGAGA[dT][dT]	UCUCAGAUCACAAACGAUG[dT][dT]
KDR_1	CUGACUUGGCCUCGGUCAU[dT][dT]	AUGACCGAGGCCAAGUCAG[dT][dT]
KDR_2	GACAUUGCGUGGUCAGGCA[dT][dT]	UGCCUGACCACGCAAUGUC[dT][dT]
KDR_3	GGAUAUCACUCCGAUGACA[dT][dT]	UGUCAUCGGAGUGAUAUCC[dT][dT]
KIAA0100_1	CAAUGUCUGUCUCGUAUCU[dT][dT]	AGAUACGAGACAGACAUUG[dT][dT]
KIAA0100_2	CACUUAGUAGAUUUACGGA[dT][dT]	UCCGUAAAUCUACUAAGUG[dT][dT]
KIAA0100_3	GGUUAUAUGAUGGGUACAA[dT][dT]	UUGUACCCAUCAUAUAACC[dT][dT]
KIAA1324L_1	CAGAUAAUGUACAAGUGGA[dT][dT]	UCCACUUGUACAUUAUCUG[dT][dT]
KIAA1324L_2	CAAUCAGGACCAUUCGGUU[dT][dT]	AACCGAAUGGUCCUGAUUG[dT][dT]
KIAA1324L_3	GAGAUUGAGGGAGCCUGCA[dT][dT]	UGCAGGCUCCCUCAAUCUC[dT][dT]
KIAA1797_1	CAGCUAUUUCUCAAGUGUU[dT][dT]	AACACUUGAGAAAUAGCUG[dT][dT]
KIAA1797_2	GUGAUUGGUCUCCAGUCAA[dT][dT]	UUGACUGGAGACCAAUCAC[dT][dT]
KIAA1797_3	GACAUGAUGCAGAGAGUUA[dT][dT]	UAACUCUCUGCAUCAUGUC[dT][dT]
KLF2_1	CUUUCGGUGGCCCUGGUUU[dT][dT]	AAACCAGGGCCACCGAAAG[dT][dT]
KLF2_2	CAGAGAACCGGGCCGGGCA[dT][dT]	UGCCCGGCCCGGUUCUCUG[dT][dT]
KLF2_3	GAGAGACAGGUGGGCAUUU[dT][dT]	AAAUGCCCACCUGUCUCUC[dT][dT]
KLF4_1	CGAAUAACCGCUGGCGGGA[dT][dT]	UCCCGCCAGCGGUUAUUCG[dT][dT]
KLF4_2	CAAAGAGUUCCCAUCUCAA[dT][dT]	UUGAGAUGGGAACUCUUUG[dT][dT]
KLF4_3	CAGAAUUCCCUUGAAUUGU[dT][dT]	ACAAUUCAAGGGAAUUCUG[dT][dT]
KTN1_1	GCUUAAACCUGACCAAGUA[dT][dT]	UUGCGGGACUCAUUGUUAC[dT][dT]
KTN1_2	CAAAUGCAGGUUCAGGAUA[dT][dT]	UAAAUAGGCUGUAAGUUUC
KTN1_3	CAGAGUAAAUUUGUGGCCA[dT][dT]	AAUCUGGAACAUUGCAAUC
KTN1_4	GAAGUACAGAGUCUGCAUA[dT][dT]	UAUGCAGACUCUGUACUUC
LAMA3_1	GGAUAAAUGUGGACGUUCA[dT][dT]	UGAACGUCCACAUUUAUCC[dT][dT]
LAMA3_2	CAAUGUCACAGCUCACAUA[dT][dT]	UAUGUGAGCUGUGACAUUG[dT][dT]
LAMA3_3	CAACUACCGUUCUGCCAUU[dT][dT]	AAUGGCAGAACGGUAGUUG[dT][dT]
LAMA4_1	GAUGUGAUCAGCUUGUACA[dT][dT]	UGUACAAGCUGAUCACAUC[dT][dT]
LAMA4_2	GUCAGUUCGGAAACUUGAA[dT][dT]	UUCAAGUUUCCGAACUGAC[dT][dT]
LAMA4_3	CUGUGAAGCGGCCGGAACU[dT][dT]	AGUUCCGGCCGCUUCACAG[dT][dT]
LAMA5_1	CUGAUCACCUGCACGGGCA[dT][dT]	UGCCCGUGCAGGUGAUCAG[dT][dT]

LAMA5_2	GUUAUGAACUGCACUCAGA[dT][dT]	UCUGAGUGCAGUUCAUAAC[dT][dT]
LAMA5_3	GCGUAUUUGAUCUGCAGCA[dT][dT]	UGCUGCAGAUCAAAUACGC[dT][dT]
LAMB1_1	CUACCAAGAUCCUGUUACU[dT][dT]	AGUAACAGGAUCUUGGUAG[dT][dT]
LAMB1_2	CAUAGUGGAGCGGCAAUAU[dT][dT]	AUAUUGCCGCUCCACUAUG[dT][dT]
LAMB1_3	GACUGAAUGUGGCGGGCCA[dT][dT]	UGGCCCGCCACAUUCAGUC[dT][dT]
LAMB2_1	CUGUUACUGCAAACGUCUA[dT][dT]	UAGACGUUUGCAGUAACAG[dT][dT]
LAMB2_2	GGAUUCAGAACCUGUUGAA[dT][dT]	UUCAACAGGUUCUGAAUCC[dT][dT]
LAMB2_3	CUUGACUUGCUCAAACAUU[dT][dT]	AAUGUUUGAGCAAGUCAAG[dT][dT]
LAMB3_1	GACUUUCAUGCAGCCUAAA[dT][dT]	UUUAGGCUGCAUGAAAGUC[dT][dT]
LAMB3_2	CCCAAAUUGUGAGCGCUGU[dT][dT]	ACAGCGCUCACAAUUUGGG[dT][dT]
LAMB3_3	CCAACUUAACCUUAUGGAU[dT][dT]	AUCCAUAAGGUUAAGUUGG[dT][dT]
LAMC1_1	GUACUACAGUGGUUCCUGU[dT][dT]	ACAGGAACCACUGUAGUAC[dT][dT]
LAMC1_2	GCUACUUUCCUCGGUACUU[dT][dT]	AAGUACCGAGGAAAGUAGC[dT][dT]
LAMC1_3	CAGAUCAAGCCUUCGAGGA[dT][dT]	UCCUCGAAGGCUUGAUCUG[dT][dT]
LAMC2_1	GGAAUCAACAGGUGAGCUA[dT][dT]	UAGCUCACCUGUUGAUUCC[dT][dT]
LAMC2_2	CAGAUAGGUCUUAUCAGCA[dT][dT]	UGCUGAUAAGACCUAUCUG[dT][dT]
LAMC2_3	CAGAAUACAGUGUCCAUAA[dT][dT]	UUAUGGACACUGUAUUCUG[dT][dT]
LAMP1_1	GGCUUAGGGUCCUGUCGAA[dT][dT]	UACUUGGUCAGGUUUAAGC[dT][dT]
LAMP1_2	GUUACAGCGUCCAGCUCAU[dT][dT]	UUCCCGCAAACAUAGUUUC
LAMP1_3	CCUAUGAGAGGAAGGACAA[dT][dT]	AAUGCGAUCAGUAAUAACC
LAPTM4A_1	CCUAUGAAAUGGCCGUGAA[dT][dT]	UUCGACAGGACCCUAAGCC[dT][dT]
LAPTM4A_2	CUAUUUGCCAAGAAUCAAA[dT][dT]	UAUCCUGAACCUGCAUUUG
LAPTM4A_3	CUAAUUAACUGUGUUUGGA[dT][dT]	UCGAUAAUCAGGAUUAAGC
LAPTM5_1	GUCCUUAUCUUCAAGGUCU[dT][dT]	AGACCUUGAAGAUAAGGAC[dT][dT]
LAPTM5_2	GACUUGAUCAGUUCAGCCA[dT][dT]	UGGCUGAACUGAUCAAGUC[dT][dT]
LAPTM5_3	CAAGUAAUCAUUAAUUAGU[dT][dT]	ACUAAUUAAUGAUUACUUG[dT][dT]
LCAT_1	GUAGACUGCUGGAUCGAUA[dT][dT]	UAUCGAUCCAGCAGUCUAC[dT][dT]
LCAT_2	GCUGUCUACACUUGCUCUA[dT][dT]	UAGAGCAAGUGUAGACAGC[dT][dT]
LCAT_3	GUGCAGAACCUGGUCAACA[dT][dT]	UGUUGACCAGGUUCUGCAC[dT][dT]
LIPG_1	GUUACAAGAACAUGGGAGA[dT][dT]	UCUCCCAUGUUCUUGUAAC[dT][dT]
LIPG_2	GAAAGACGCCAAUGUAGUU[dT][dT]	AACUACAUUGGCGUCUUUC[dT][dT]
LIPG_3	CAAACCAUCUGUGAGGUUU[dT][dT]	AAACCUCACAGAUGGUUUG[dT][dT]
LOX_1	GACUAACUUCAGUAGGAUU[dT][dT]	AAUCCUACUGAAGUUAGUC[dT][dT]
LOX_2	GCGAUUUGCAUGUACUGCA[dT][dT]	UGCAGUACAUGCAAAUCGC[dT][dT]
LOX_3	GAAUCUGACUAUACCAACA[dT][dT]	UGUUGGUAUAGUCAGAUUC[dT][dT]

LOXL2_1	CAACUUCGAGGUUGCAGAA[dT][dT]	UUCUGCAACCUCGAAGUUG[dT][dT]
LOXL2_2	CAAUACCAAAGUGUACAAA[dT][dT]	UUUGUACACUUUGGUAUUG[dT][dT]
LOXL2_3	GAGACUACCUGUUCCAGGU[dT][dT]	ACCUGGAACAGGUAGUCUC[dT][dT]
LPCAT2_1	GUGUCUUAUUGGUUGCGUU[dT][dT]	AACGCAACCAAUAAGACAC[dT][dT]
LPCAT2_2	CAAUAAAGUCCGGAAUUUA[dT][dT]	UAAAUUCCGGACUUUAUUG[dT][dT]
LPCAT2_3	CUUAAAGCAUCCAGAAUAU[dT][dT]	AUAUUCUGGAUGCUUUAAG[dT][dT]
LPHN2_1	GCAAUUGUGGACUCACCAU[dT][dT]	AUGGUGAGUCCACAAUUGC[dT][dT]
LPHN2_2	CAAUCCAACUGUCCGCAAA[dT][dT]	UUUGCGGACAGUUGGAUUG[dT][dT]
LPHN2_3	CCACUUAUUCCUCAGCGGA[dT][dT]	UCCGCUGAGGAAUAAGUGG[dT][dT]
LRIG1_1	GAUCUGAGUUUGAACAACA[dT][dT]	UGUUGUUCAAACUCAGAUC[dT][dT]
LRIG1_2	GAAUCUUAAAGAGCUCCAU[dT][dT]	AUGGAGCUCUUUAAGAUUC[dT][dT]
LRIG1_3	CAGUACUUGCUUGUUUCCA[dT][dT]	UGGAAACAAGCAAGUACUG[dT][dT]
LRIG3_1	GACCUAACUUUCAAUCACU[dT][dT]	AGUGAUUGAAAGUUAGGUC[dT][dT]
LRIG3_2	GCAAUCAGCUUCUGAUUAU[dT][dT]	AUAAUCAGAAGCUGAUUGC[dT][dT]
LRIG3_3	GUCUUAUGACUUGGACACA[dT][dT]	UGUGUCCAAGUCAUAAGAC[dT][dT]
LRP10_1	CAGAUCUUACGCCAGGAUA[dT][dT]	UUCACGGCCAUUUCAUAGG[dT][dT]
LRP10_2	CUCAUAGUGGCACAACCUU[dT][dT]	AUGAGCUGGACGCUGUAAC
LRP10_3	CACAGUUGCUUGGAGCAAU[dT][dT]	UGGCCACAAAUUUACUCUG
LRRC17_1	GAAACAAGAUCCGCACAUU[dT][dT]	AAUGUGCGGAUCUUGUUUC[dT][dT]
LRRC17_2	GUGAGAAGCCGAGUGAAUC[dT][dT]	GAUUCACUCGGCUUCUCAC[dT][dT]
LRRC17_3	GUGUGAGAAGCCGAGUGAA[dT][dT]	UUCACUCGGCUUCUCACAC[dT][dT]
LRRC32_1	GACCUGAGCACCAAUGAGA[dT][dT]	UCUCAUUGGUGCUCAGGUC[dT][dT]
LRRC32_2	GAGAACAAACUGCUCCAUU[dT][dT]	AAUGGAGCAGUUUGUUCUC[dT][dT]
LRRC32_3	CCGAGAACCGCCUGAGCCA[dT][dT]	UGGCUCAGGCGGUUCUCGG[dT][dT]
LRRC8C_1	CAUCCAAUCUGGUCCAGAA[dT][dT]	UUCUGGACCAGAUUGGAUG[dT][dT]
LRRC8C_2	CUGGAAUUGCCUCUUAUCA[dT][dT]	UGAUAAGAGGCAAUUCCAG[dT][dT]
LRRC8C_3	CUACAUCCCAGAGCAUAUA[dT][dT]	UAUAUGCUCUGGGAUGUAG[dT][dT]
LTBP1_1	CUACAAGGUGUAUGCCCUA[dT][dT]	UAGGGCAUACACCUUGUAG[dT][dT]
LTBP1_2	CUGUUACCGACUUGUCAGU[dT][dT]	ACUGACAAGUCGGUAACAG[dT][dT]
LTBP1_3	GUCCUAUGAUUGUACUUGU[dT][dT]	ACAAGUACAAUCAUAGGAC[dT][dT]
LTBP2_1	GCUACAUCAUGGUCAGGAA[dT][dT]	UUCCUGACCAUGAUGUAGC[dT][dT]
LTBP2_2	GAGCUUAUGCUGGCGGUAU[dT][dT]	AUACCGCCAGCAUAAGCUC[dT][dT]
LTBP2_3	CCAAUGAUGUGUGCAGCGA[dT][dT]	UCGCUGCACACAUCAUUGG[dT][dT]
LTBP3_1	CCAUUCAGGGCGAGAGUGA[dT][dT]	UCACUCUCGCCCUGAAUGG[dT][dT]
LTBP3_2	CAGUCUACAGCUCAGCCGA[dT][dT]	UCGGCUGAGCUGUAGACUG[dT][dT]

LTBP3_3	CUGAUGAGUGCCGACUGAA[dT][dT]	UUCAGUCGGCACUCAUCAG[dT][dT]
LTBP4_1	GCUAUUGCAGCAACGGCUA[dT][dT]	UAGCCGUUGCUGCAAUAGC[dT][dT]
LTBP4_2	GAGUUAAUGGGUCCUGCGA[dT][dT]	UCGCAGGACCCAUUAACUC[dT][dT]
LTBP4_3	CUCCUUUGCCUGUACUUGU[dT][dT]	ACAAGUACAGGCAAAGGAG[dT][dT]
LY96_1	UACCCAAUUUCAAUUAAUG[dT][dT]	CAUUAAUUGAAAUUGGGUA[dT][dT]
LY96_2	CUUCCAAAGCGCAAAGAAG[dT][dT]	CUUCUUUGCGCUUUGGAAG[dT][dT]
LY96_3	UCCAAAGGAUUAUUGCACA[dT][dT]	UGUGCAAUAAUCCUUUGGA[dT][dT]
MANF_1	CGAUUUGUAGUCUGCUCAA[dT][dT]	UUGAGCAGACUACAAAUCG[dT][dT]
MANF_2	CAUGCAAAGGCUGUGCAGA[dT][dT]	UCUGCACAGCCUUUGCAUG[dT][dT]
MANF_3	CUGAGCACAGUGGACCUGA[dT][dT]	UCAGGUCCACUGUGCUCAG[dT][dT]
MANSC1_1	CUUGACUUACACUUUGGUA[dT][dT]	UACCAAAGUGUAAGUCAAG[dT][dT]
MANSC1_2	GAAACUAUUUAAGAUGGAU[dT][dT]	AUCCAUCUUAAAUAGUUUC[dT][dT]
MANSC1_3	CUUAUCGGGUCCCUGCUCU[dT][dT]	AGAGCAGGGACCCGAUAAG[dT][dT]
MARVELD1_1	GUUGUACCCUUACACUUGU[dT][dT]	ACAAGUGUAAGGGUACAAC[dT][dT]
MARVELD1_2	GGUAUGAGAAGAACACGUA[dT][dT]	UACGUGUUCUUCUCAUACC[dT][dT]
MARVELD1_3	CUCAUUGUUUAGUGGUGAU[dT][dT]	AUCACCACUAAACAAUGAG[dT][dT]
MATN2_1	CCAAUUUCAGCCAGAUUGA[dT][dT]	UCAAUCUGGCUGAAAUUGG[dT][dT]
MATN2_2	CAAAGCUCAGGCUAUUGUU[dT][dT]	AACAAUAGCCUGAGCUUUG[dT][dT]
MATN2_3	GUUAUAUACUCCGUGAAGA[dT][dT]	UCUUCACGGAGUAUAUAAC[dT][dT]
MATN3_1	CGAAUCACACCCUUGUCAA[dT][dT]	UUGACAAGGGUGUGAUUCG[dT][dT]
MATN3_2	CUUGAUAGGUGUGCUCUUA[dT][dT]	UAAGAGCACACCUAUCAAG[dT][dT]
MATN3_3	CUGUGAAGAUCGAGUUCCA[dT][dT]	UGGAACUCGAUCUUCACAG[dT][dT]
MBOAT7_1	CAUCGACUGCUACAGCACA[dT][dT]	UGUGCUGUAGCAGUCGAUG[dT][dT]
MBOAT7_2	CAGAAAGAGUCCUUACCUU[dT][dT]	AAGGUAAGGACUCUUUCUG[dT][dT]
MBOAT7_3	GUGUCUUUCGCUCCACUUU[dT][dT]	AAAGUGGAGCGAAAGACAC[dT][dT]
MBTPS1_1	CAAAGCUAGAAAUUCAUUU[dT][dT]	AAAUGAAUUUCUAGCUUUG[dT][dT]
MBTPS1_2	CUGAUCAAAUGGAUGUGAU[dT][dT]	AUCACAUCCAUUUGAUCAG[dT][dT]
MBTPS1_3	GUGUUACCCAACUUUCGAU[dT][dT]	AUCGAAAGUUGGGUAACAC[dT][dT]
MCAM_1	CUACUCUGGCCCUGACUCA[dT][dT]	UGAGUCAGGGCCAGAGUAG[dT][dT]
MCAM_2	CUAUGUGUCUGACGUCCGA[dT][dT]	UCGGACGUCAGACACAUAG[dT][dT]
MCAM_3	GAGAGAAAUACAUCGAUCU[dT][dT]	AGAUCGAUGUAUUUCUCUC[dT][dT]
MCL1_1	GUAAUAGAACUAUGACUGU[dT][dT]	UAUCCUGGCGUAAGAUCUG[dT][dT]
MCL1_2	GAAUUGAUUACCCGCCGAA[dT][dT]	UUUGAUUCUUGGCAAAUAG
MCL1_3	CCAGUAUACUUCUUAGAAA[dT][dT]	UUGUCCUUCCUCUCAUAGG
MCTP1_1	GGAUCUUAUUUCUCUGUGA[dT][dT]	UCACAGAGAAAUAAGAUCC[dT][dT]

MCTP1_2	GAUAUAGCCCAUUGAGGAU[dT][dT]	AUCCUCAAUGGGCUAUAUC[dT][dT]
MCTP1_3	GAACUACUUCUUGAUAAUA[dT][dT]	UAUUAUCAAGAAGUAGUUC[dT][dT]
MDK_1	GGACUAGACGCCAAGCCUG[dT][dT]	CAGGCUUGGCGUCUAGUCC[dT][dT]
MDK_2	GAGUUUGGAGCCGACUGCA[dT][dT]	UGCAGUCGGCUCCAAACUC[dT][dT]
MDK_3	GUCUGCUCGUUAGCUUUAA[dT][dT]	UUAAAGCUAACGAGCAGAC[dT][dT]
MEGF6_1	GGAUUGAGAUGGAAAUCGU[dT][dT]	ACGAUUUCCAUCUCAAUCC[dT][dT]
MEGF6_2	GCUACGAGCUGGACACAGA[dT][dT]	UCUGUGUCCAGCUCGUAGC[dT][dT]
MEGF6_3	GUCAGUAUGAUGUGGACGA[dT][dT]	UCGUCCACAUCAUACUGAC[dT][dT]
MERTK_1	CGCUAUUCCCGGGACCUUU[dT][dT]	AAAGGUCCCGGGAAUAGCG[dT][dT]
MERTK_2	GGAUAUCCCACGUGUGGCA[dT][dT]	UGCCACACGUGGGAUAUCC[dT][dT]
MERTK_3	CUGUUAAAUGGAUCGCCAU[dT][dT]	AUGGCGAUCCAUUUAACAG[dT][dT]
METRNL_1	CAGACUCUAUCGGCAGAAA[dT][dT]	UUUCUGCCGAUAGAGUCUG[dT][dT]
METRNL_2	GUCAGGACGCUGCUGGAGU[dT][dT]	ACUCCAGCAGCGUCCUGAC[dT][dT]
METRNL_3	CGCUUCAAGGACUUCCAGA[dT][dT]	UCUGGAAGUCCUUGAAGCG[dT][dT]
MFAP2_1	GUCUCAACGAGGUCUGCUU[dT][dT]	AAGCAGACCUCGUUGAGAC[dT][dT]
MFAP2_2	GAGCUGACCUCUGUCGGGA[dT][dT]	UCCCGACAGAGGUCAGCUC[dT][dT]
MFAP2_3	GGAGAUCUGUGUUCGUACA[dT][dT]	UGUACGAACACAGAUCUCC[dT][dT]
MFGE8_1	CGAUUUCAUCCAUGAUGUU[dT][dT]	AACAUCAUGGAUGAAAUCG[dT][dT]
MFGE8_2	CACUCUGCGCUUUGAGCUA[dT][dT]	UAGCUCAAAGCGCAGAGUG[dT][dT]
MFGE8_3	CGAAUUCGAUUUCAUCCAU[dT][dT]	AUGGAUGAAAUCGAAUUCG[dT][dT]
MMP1_1	CUUGAAGCUGCUUACGAAU[dT][dT]	AUUCGUAAGCAGCUUCAAG[dT][dT]
MMP1_2	CAAUACAAAUUUGAUCCUA[dT][dT]	UAGGAUCAAAUUUGUAUUG[dT][dT]
MMP1_3	CUAGAACUGUGAAGCAUAU[dT][dT]	AUAUGCUUCACAGUUCUAG[dT][dT]
MMP11_1	GCCUCUACUGGAAGUUUGA[dT][dT]	UCAAACUUCCAGUAGAGGC[dT][dT]
MMP11_2	GAUGUGACGCCACUCACCU[dT][dT]	AGGUGAGUGGCGUCACAUC[dT][dT]
MMP11_3	GUAUCCCUGUUGUGAGGUU[dT][dT]	AACCUCACAACAGGGAUAC[dT][dT]
MMP14_1	CAAAUGGCAACAUAAUGAA[dT][dT]	ACAGUCAUAGUUCUAUUAC[dT][dT]
MMP14_2	CAAGCAUUGGGUGUUUGAU[dT][dT]	AAGGUUGUGCCACUAUGAG
MMP14_3	GUACUACCGUUUCAACGAA[dT][dT]	UCCAAACACAGUUAAUUAG
MMP16_1	GACAAGAGGUAGCUCCAAA[dT][dT]	UUUGGAGCUACCUCUUGUC[dT][dT]
MMP16_2	CUCAGAUGAGCCAUGGACA[dT][dT]	UGUCCAUGGCUCAUCUGAG[dT][dT]
MMP16_3	CUCCUAUCCCGGAGCCAAA[dT][dT]	UUUGGCUCCGGGAUAGGAG[dT][dT]
MMRN1_1	CUUCAACUGCAAGUAUUAA[dT][dT]	UUAAUACUUGCAGUUGAAG[dT][dT]
MMRN1_2	CUCGAAAGCACCAGGCAAA[dT][dT]	UUUGCCUGGUGCUUUCGAG[dT][dT]
MMRN1_3	CUGAUAAGAGACAUAGUAA[dT][dT]	UUACUAUGUCUCUUAUCAG[dT][dT]

MMRN2_1	CUUUCGAUCAGAUUAGCAA[dT][dT]	UUGCUAAUCUGAUCGAAAG[dT][dT]
MMRN2_2	CGAGUAUGGUUUGAGUUAA[dT][dT]	UUAACUCAAACCAUACUCG[dT][dT]
MMRN2_3	GAGACUUUCGAUCAGAUUA[dT][dT]	UAAUCUGAUCGAAAGUCUC[dT][dT]
MPZL2_1	GGCCUAUAGCAGCUGUGGA[dT][dT]	UCCACAGCUGCUAUAGGCC[dT][dT]
MPZL2_2	CCAUGAGUGGGCGGUUUAA[dT][dT]	UUAAACCGCCCACUCAUGG[dT][dT]
MPZL2_3	CACUGUACGCUUCUCUGAG[dT][dT]	CUCAGAGAAGCGUACAGUG[dT][dT]
MST1_1	GACGUGUGCGGGAGAGUGA[dT][dT]	UCACUCUCCCGCACACGUC[dT][dT]
MST1_2	GAAUCGGCAGGGCCAGCAU[dT][dT]	AUGCUGGCCCUGCCGAUUC[dT][dT]
MST1_3	GCAAAGACCUUCGGGAGAA[dT][dT]	UUCUCCCGAAGGUCUUUGC[dT][dT]
MTCH1_1	CCAAGUACAUCGUGCAAGU[dT][dT]	UUCAUUAUGUUGCCAUUUG[dT][dT]
MTCH1_2	CUGGGAUUCUUCGUUGGAU[dT][dT]	UUCGGCGGGUAAUCAAUUC
MTCH1_3	CCUACGAGAUGAUGAUGCA[dT][dT]	AUUGCUCCAAGCAACUGUG
MTDH_1	CAGUUCACGCCAUGAUGGA[dT][dT]	ACUUGCACGAUGUACUUGG[dT][dT]
MTDH_2	GAAACGACAGCAGCGUAAA[dT][dT]	AUCAAACACCCAAUGCUUG
MTDH_3	GUUACCACCGAGCAACUUA[dT][dT]	UUUCUAAGAAGUAUACUGG
MTUS1_1	CACAUCCUCCGCCAUCCCU[dT][dT]	UCCAUCAUGGCGUGAACUG[dT][dT]
MTUS1_1	GUAAGCAGGUGUUAGAUAU[dT][dT]	AUAUCUAACACCUGCUUAC[dT][dT]
MTUS1_2	GGAUGUCACAGAGUGUCAU[dT][dT]	AUCCAACGAAGAAUCCCAG
MTUS1_2	GAGAUUAUGAAUGAGACUU[dT][dT]	AAGUCUCAUUCAUAAUCUC[dT][dT]
MTUS1_3	GGAAUGAGUUACAAACAGU[dT][dT]	UUCGUUGAAACGGUAGUAC
MTUS1_3	CUGGAUAUGUACUAAUCAA[dT][dT]	UUGAUUAGUACAUAUCCAG[dT][dT]
MYCT1_1	GCCUCAACAGAACUGGAUU[dT][dT]	AAUCCAGUUCUGUUGAGGC[dT][dT]
MYCT1_2	GUGACUCUCCCUUCUUCCA[dT][dT]	UGGAAGAAGGGAGAGUCAC[dT][dT]
MYCT1_3	GCCUAUGAGUCCAUCAUCA[dT][dT]	UGAUGAUGGACUCAUAGGC[dT][dT]
MYOF_1	GUGAUGAAGACAGGUUGGA[dT][dT]	UCCAACCUGUCUUCAUCAC[dT][dT]
MYOF_2	GGAUUAGUAGGACGUCAUA[dT][dT]	UAUGACGUCCUACUAAUCC[dT][dT]
MYOF_3	GGAGUAUUGACCAAACGGA[dT][dT]	UCCGUUUGGUCAAUACUCC[dT][dT]
NBL1_1	GCGUCUAUGUGCAGGGCGA[dT][dT]	UCGCCCUGCACAUAGACGC[dT][dT]
NBL1_2	GCACUGUUCCCAGAUAAGA[dT][dT]	UCUUAUCUGGGAACAGUGC[dT][dT]
NBL1_3	CCAAGCUGCACAAUUUAAU[dT][dT]	AUUAAAUUGUGCAGCUUGG[dT][dT]
NDST1_1	CCAACUACUUUGAUUCAGA[dT][dT]	AGGGAUGGCGGAGGAUGUG[dT][dT]
NDST1_2	GUCUUUGUGGAGAGCCUCU[dT][dT]	UUUACGCUGCUGUCGUUUC
NDST1_3	CACCAUUGACUACCACAAA[dT][dT]	UGCAUCAUCAUCUCGUAGG
NENF_1	GCUACACUGCCCGGAGAAU[dT][dT]	AUUCUCCGGGCAGUGUAGC[dT][dT]
NENF_2	CCCGGAGAAUUCUCAAUGA[dT][dT]	UCAUUGAGAAUUCUCCGGG[dT][dT]

NENF_3	CGGCUACACUGCCCGGAGA[dT][dT]	UCUCCGGGCAGUGUAGCCG[dT][dT]
NF2_1	GGAAGCAACCCAAGACGUU[dT][dT]	AACGUCUUGGGUUGCUUCC[dT][dT]
NF2_2	GGAGUUUACUAUUAAACCA[dT][dT]	UGGUUUAAUAGUAAACUCC[dT][dT]
NF2_3	CUCUCUAUGUGGUGAUGGU[dT][dT]	ACCAUCACCACAUAGAGAG[dT][dT]
NGRN_1	GAACUUCCUGUACAGAAUU[dT][dT]	AAUUCUGUACAGGAAGUUC[dT][dT]
NGRN_2	GUGAAACACAGCUGGGCAA[dT][dT]	UUGCCCAGCUGUGUUUCAC[dT][dT]
NGRN_3	GGAACUUCCUGUACAGAAU[dT][dT]	AUUCUGUACAGGAAGUUCC[dT][dT]
NHLRC3_1	CACAUCUCCUAGAAGUCGA[dT][dT]	UCGACUUCUAGGAGAUGUG[dT][dT]
NHLRC3_2	CCUAAGCACCCAGAAUAUU[dT][dT]	AAUAUUCUGGGUGCUUAGG[dT][dT]
NHLRC3_3	GGGAAUAUUUCAAGUGGCA[dT][dT]	UGCCACUUGAAAUAUUCCC[dT][dT]
NID1_1	GUGAAACUGGCCUUCAUAA[dT][dT]	UUAUGAAGGCCAGUUUCAC[dT][dT]
NID1_2	GAGUUGACUGUAUCGAACA[dT][dT]	UGUUCGAUACAGUCAACUC[dT][dT]
NID1_3	GCAAUCUGGUCAUUAAGCA[dT][dT]	UGCUUAAUGACCAGAUUGC[dT][dT]
NID2_1	GUCUUACAAUGUCCAGCUU[dT][dT]	AAGCUGGACAUUGUAAGAC[dT][dT]
NID2_2	CCAUAAUCGUGGGAAUUGA[dT][dT]	UCAAUUCCCACGAUUAUGG[dT][dT]
NID2_3	CGUAUAAUGCUGCCAACAA[dT][dT]	UUGUUGGCAGCAUUAUACG[dT][dT]
NOTCH1_1	GCUACAACUGCGUGUGUGU[dT][dT]	ACACACACGCAGUUGUAGC[dT][dT]
NOTCH1_2	GCAACAAGGUCUGCAGCCU[dT][dT]	AGGCUGCAGACCUUGUUGC[dT][dT]
NOTCH1_3	GUUUGAAUGGUCAAUGCGA[dT][dT]	UCGCAUUGACCAUUCAAAC[dT][dT]
NOTCH4_1	GAACUCUGCUGGUAGCUUU[dT][dT]	AAAGCUACCAGCAGAGUUC[dT][dT]
NOTCH4_2	CAUGGAACCUGUACUCCCA[dT][dT]	UGGGAGUACAGGUUCCAUG[dT][dT]
NOTCH4_3	CCGAUAAAGAUGCCCAGGA[dT][dT]	UCCUGGGCAUCUUUAUCGG[dT][dT]
NPC2_1	CUCAUUGAGUUCGGUGCAU[dT][dT]	AUGCACCGAACUCAAUGAG[dT][dT]
NPC2_2	CAGUACAGAUCGUUUCUCA[dT][dT]	UGAGAAACGAUCUGUACUG[dT][dT]
NPC2_3	GGCAACUUCAGGAUGACAA[dT][dT]	UUGUCAUCCUGAAGUUGCC[dT][dT]
NPR3_1	CAUCACAUUUCUCUGAAGA[dT][dT]	UCUUCAGAGAAAUGUGAUG[dT][dT]
NPR3_2	CUCUUUGGGAGAUCAAAUA[dT][dT]	UAUUUGAUCUCCCAAAGAG[dT][dT]
NPR3_3	GAAUUGUAGAGCAUACAAA[dT][dT]	UUUGUAUGCUCUACAAUUC[dT][dT]
NRCAM_1	CCAAUAUUCCAUUUCUCUA[dT][dT]	UAGAGAAAUGGAAUAUUGG[dT][dT]
NRCAM_2	CUGGAAUGCAAAGUGCAUA[dT][dT]	UAUGCACUUUGCAUUCCAG[dT][dT]
NRCAM_3	CUAUGUAUAGCUGUCGUUA[dT][dT]	UAACGACAGCUAUACAUAG[dT][dT]
NRP1_1	CUGAAUGUUCCCAGAACUA[dT][dT]	UCUGAAUCAAAGUAGUUGG[dT][dT]
NRP1_2	GAUGUUGUGGUUGCAGUAU[dT][dT]	AUGACACUCUGUGACAUCC
NRP1_3	CGAUAAAUGUGGCGAUACU[dT][dT]	UAAGUUGCUCGGUGGUAAC
NRP2_1	GCAAGUAUGACUUUAUCGA[dT][dT]	UCGAUAAAGUCAUACUUGC[dT][dT]

NRP2_2	CAAGUACUGUGGGACCAAA[dT][dT]	UUUGGUCCCACAGUACUUG[dT][dT]
NRP2_3	CCAACAACGAUGCAACUGA[dT][dT]	UCAGUUGCAUCGUUGUUGG[dT][dT]
NT5E_1	GCACUAUCUGGUUCACCGU[dT][dT]	ACGGUGAACCAGAUAGUGC[dT][dT]
NT5E_2	GAAAGUAAUUUAUCCAGCA[dT][dT]	UGCUGGAUAAAUUACUUUC[dT][dT]
NT5E_3	CAGUUGAAGGUCGGAUCAA[dT][dT]	UUGAUCCGACCUUCAACUG[dT][dT]
NTN4_1	CUUACAGUGCCAAAGUUCA[dT][dT]	UAGUUCUGGGAACAUUCAG[dT][dT]
NTN4_2	GUUAGACCUGGAAGCUGAA[dT][dT]	AGAGGCUCUCCACAAAGAC
NTN4_3	GUACUCAUGUUGAGGUCAA[dT][dT]	ACUGUUUGUAACUCAUUCC
NUCB1_1	CUCAGAACCAGCAUACAUU[dT][dT]	AAUGUAUGCUGGUUCUGAG[dT][dT]
NUCB1_2	CCUUCUUCAUACUGCAUGA[dT][dT]	UCAUGCAGUAUGAAGAAGG[dT][dT]
NUCB1_3	GGAGUUCCUCGCAUCCACU[dT][dT]	AGUGGAUGCGAGGAACUCC[dT][dT]
NXPE3_1	CCCAUUAUCUACCGCACAA[dT][dT]	UUGUGCGGUAGAUAAUGGG[dT][dT]
NXPE3_2	GGAUUACCAGAAUGGGUUU[dT][dT]	AAACCCAUUCUGGUAAUCC[dT][dT]
NXPE3_3	CAGAGUUCUUCAGCGCUUA[dT][dT]	UAAGCGCUGAAGAACUCUG[dT][dT]
ORAI2_1	CCUGGAACUGGUCACCUCU[dT][dT]	AGAGGUGACCAGUUCCAGG[dT][dT]
ORAI2_2	GCCACAACCGCGAGAUCGA[dT][dT]	UCGAUCUCGCGGUUGUGGC[dT][dT]
ORAI2_3	GACACGAAGGCUUCGGCGU[dT][dT]	ACGCCGAAGCCUUCGUGUC[dT][dT]
OSTC_1	CUUUGUACCGUGUCCCGUU[dT][dT]	AACGGGACACGGUACAAAG[dT][dT]
OSTC_2	GUAAAUGGACAAUAUAUUA[dT][dT]	UAAUAUAUUGUCCAUUUAC[dT][dT]
OSTC_3	CGUCUGUGUCCUAUUGAGU[dT][dT]	ACUCAAUAGGACACAGACG[dT][dT]
OXA1L_1	CUCAGUACCUCUGCUAUCU[dT][dT]	AGAUAGCAGAGGUACUGAG[dT][dT]
OXA1L_2	CCAUCUACAUAUUACCACU[dT][dT]	AGUGGUAAUAUGUAGAUGG[dT][dT]
OXA1L_3	CAAAUUACCUCCACGGGAA[dT][dT]	UUCCCGUGGAGGUAAUUUG[dT][dT]
P2RX4_1	GAUACCAGCUCAGGAGGAA[dT][dT]	UUCCUCCUGAGCUGGUAUC[dT][dT]
P2RX4_2	CUCAUCAAGGCCUAUGGCA[dT][dT]	UGCCAUAGGCCUUGAUGAG[dT][dT]
P2RX4_3	GUUCUUGGCUGGGUCAACU[dT][dT]	AGUUGACCCAGCCAAGAAC[dT][dT]
PAM_1	CACGUUUAAAGUUCUGUGU[dT][dT]	UGAACUUUGGCACUGUAAG[dT][dT]
PAM_2	CCAUUUAGGUAAGGUAGUA[dT][dT]	AUACUGCAACCACAACAUC
PAM_3	CACCAAAGAAUUUGUGAGA[dT][dT]	UUUGUGGUAGUCAAUGGUG
PAPLN_1	GCUUUGUGGCAAUGAGUAU[dT][dT]	AUACUCAUUGCCACAAAGC[dT][dT]
PAPLN_2	GUGAUACGGCCAGGCUAUU[dT][dT]	AAUAGCCUGGCCGUAUCAC[dT][dT]
PAPLN_3	GCUAUUGUGUGUGGUAGCA[dT][dT]	UGCUACCACACACAAUAGC[dT][dT]
PARP12_1	GCUAUAAUGAGCUAUGCCA[dT][dT]	UGGCAUAGCUCAUUAUAGC[dT][dT]
PARP12_2	GAGUUAGAUUUCAAAGCCU[dT][dT]	AGGCUUUGAAAUCUAACUC[dT][dT]
PARP12_3	GAACUGUUCUCUUAACAUU[dT][dT]	AAUGUUAAGAGAACAGUUC[dT][dT]

PCDH12_1	GGUGAAAUACCAAGUGUCA[dT][dT]	UGACACUUGGUAUUUCACC[dT][dT]
PCDH12_2	CAACCAUUGUGGCAAGAGA[dT][dT]	UCUCUUGCCACAAUGGUUG[dT][dT]
PCDH12_3	CCAACUACCGUGACAAUGU[dT][dT]	ACAUUGUCACGGUAGUUGG[dT][dT]
PCYOX1L_1	GUUCCGUUCCUAUUACUCA[dT][dT]	UGAGUAAUAGGAACGGAAC[dT][dT]
PCYOX1L_2	CCAACAUCCUUACCACAGA[dT][dT]	UCUGUGGUAAGGAUGUUGG[dT][dT]
PCYOX1L_3	CCCUGUUCCGUUCCUAUUA[dT][dT]	UAAUAGGAACGGAACAGGG[dT][dT]
PDGFA_1	CCCAUGUUCUGGCCGAGGA[dT][dT]	UCCUCGGCCAGAACAUGGG[dT][dT]
PDGFA_2	CGGAUACCUCGCCCAUGUU[dT][dT]	AACAUGGGCGAGGUAUCCG[dT][dT]
PDGFA_3	GAGAUAGACUCCGUAGGGA[dT][dT]	UCCCUACGGAGUCUAUCUC[dT][dT]
PDGFB_1	GCAAGCACCGGAAAUUCAA[dT][dT]	UUGAAUUUCCGGUGCUUGC[dT][dT]
PDGFB_2	GAAAUUCAAGCACACGCAU[dT][dT]	AUGCGUGUGCUUGAAUUUC[dT][dT]
PDGFB_3	CCCUUAACUCCAAGAACUU[dT][dT]	AAGUUCUUGGAGUUAAGGG[dT][dT]
PDGFC_1	GCUAUAACUGCCUUUAGUA[dT][dT]	UACUAAAGGCAGUUAUAGC[dT][dT]
PDGFC_2	GUCCAUAAGGGAAGAACUA[dT][dT]	UAGUUCUUCCCUUAUGGAC[dT][dT]
PDGFC_3	GAUAAGAUUUGUAUCUGAU[dT][dT]	AUCAGAUACAAAUCUUAUC[dT][dT]
PDGFD_1	CUGAAAGAACCUUUAGUUU[dT][dT]	AAACUAAAGGUUCUUUCAG[dT][dT]
PDGFD_2	CUCAAGACCACCUCGAUAA[dT][dT]	UUAUCGAGGUGGUCUUGAG[dT][dT]
PDGFD_3	GUUUAAGGAGGGUGAGAUA[dT][dT]	UAUCUCACCCUCCUUAAAC[dT][dT]
PDGFRL_1	CAAUGGAACGGACAUUGUU[dT][dT]	AACAAUGUCCGUUCCAUUG[dT][dT]
PDGFRL_2	GUAGCUACCACUGUUGAGU[dT][dT]	ACUCAACAGUGGUAGCUAC[dT][dT]
PDGFRL_3	GACAUUGUUUAUGACAUGA[dT][dT]	UCAUGUCAUAAACAAUGUC[dT][dT]
PEAR1_1	GCAACAACAGCUCGUGUGA[dT][dT]	UCACACGAGCUGUUGUUGC[dT][dT]
PEAR1_2	CCCUUUACUGUGAUGCCGA[dT][dT]	UCGGCAUCACAGUAAAGGG[dT][dT]
PEAR1_3	CGCUUAACAGAGCAAGUGA[dT][dT]	UCACUUGCUCUGUUAAGCG[dT][dT]
PGF_1	CCAUGCAGCUCCUAAAGAU[dT][dT]	AUCUUUAGGAGCUGCAUGG[dT][dT]
PGF_2	GUCAUGAGGCUGUUCCCUU[dT][dT]	AAGGGAACAGCCUCAUGAC[dT][dT]
PGF_3	CCUUCAACAACGUGAGAGA[dT][dT]	UCUCUCACGUUGUUGAAGG[dT][dT]
PLA2G12A_1	GAUGGAAGAACAUACCUUU[dT][dT]	AAAGGUAUGUUCUUCCAUC[dT][dT]
PLA2G12A_2	CAAAUGUCUUGACCAAUAU[dT][dT]	AUAUUGGUCAAGACAUUUG[dT][dT]
PLA2G12A_3	CCAAAUGUCUUGACCAAUA[dT][dT]	UAUUGGUCAAGACAUUUGG[dT][dT]
PLAT_1	GGAAUUCGAUGAUGACACU[dT][dT]	AGUGUCAUCAUCGAAUUCC[dT][dT]
PLAT_2	CUGCAAAGGCGCAGUGCUU[dT][dT]	AAGCACUGCGCCUUUGCAG[dT][dT]
PLAT_3	GGAAUUCCAUGAUCCUGAU[dT][dT]	AUCAGGAUCAUGGAAUUCC[dT][dT]
PLAU_1	CUGUCUAAAUGGAGGAACA[dT][dT]	UGUUCCUCCAUUUAGACAG[dT][dT]
PLAU_2	CCUAAAGCCGCUUGUCCAA[dT][dT]	UUGGACAAGCGGCUUUAGG[dT][dT]

PLAU_3	GAUGAAGUUUGAGGUGGAA[dT][dT]	UUCCACCUCAAACUUCAUC[dT][dT]
PLSCR1_1	CAGAUAGAUCAGAUACUGA[dT][dT]	UCAGUAUCUGAUCUAUCUG[dT][dT]
PLSCR1_2	GCAUUUACAGACGCUGAUA[dT][dT]	UAUCAGCGUCUGUAAAUGC[dT][dT]
PLSCR1_3	GAAUUAAGCUGUAAGGCCU[dT][dT]	AGGCCUUACAGCUUAAUUC[dT][dT]
PLSCR4_1	GCAAAUAUCCUAUGCCAAA[dT][dT]	UUUGGCAUAGGAUAUUUGC[dT][dT]
PLSCR4_2	CCAUCAACUAUGGUUAAUU[dT][dT]	AAUUAACCAUAGUUGAUGG[dT][dT]
PLSCR4_3	GACUCAUUAACCCAGUAAA[dT][dT]	UUUACUGGGUUAAUGAGUC[dT][dT]
PLTP_1	CACCUACUUUGGGAGCAUU[dT][dT]	AAUGCUCCCAAAGUAGGUG[dT][dT]
PLTP_2	GCAUCAACUUUGUGCAUGA[dT][dT]	UCAUGCACAAAGUUGAUGC[dT][dT]
PLTP_3	GAGGAAGAGCGGAUGGUGU[dT][dT]	ACACCAUCCGCUCUUCCUC[dT][dT]
PLXNA2_1	CUAUGAGCUACACAGCGAU[dT][dT]	ACACAGAACUUUAAACGUG[dT][dT]
PLXNA2_2	GAUGCAUUAUCAGGUGUCA[dT][dT]	UUCAGCUUCCAGGUCUAAC
PLXNA2_3	GGAUCAAUAUUUCAGAGGA[dT][dT]	AGUAUCGCCACAUUUAUCG
PLXNA2_4	CAGAACAGCUCGUACCAGU[dT][dT]	ACUGGUACGAGCUGUUCUG
PLXND1_1	CAGUUUGACCCAGCAGACU[dT][dT]	AGUCUGCUGGGUCAAACUG[dT][dT]
PLXND1_2	CCCUUUAUGAAGAGCGUUA[dT][dT]	UAACGCUCUUCAUAAAGGG[dT][dT]
PLXND1_3	GGAAAUACCAGAAUGAGUU[dT][dT]	AACUCAUUCUGGUAUUUCC[dT][dT]
PMP22_1	CAAUGGACACGCAACUGAU[dT][dT]	AUCAGUUGCGUGUCCAUUG[dT][dT]
PMP22_2	CAUCUCAACUCGGAUUACU[dT][dT]	AGUAAUCCGAGUUGAGAUG[dT][dT]
PMP22_3	GUAUAUAAUAUCUCCGGUU[dT][dT]	AACCGGAGAUAUUAUAUAC[dT][dT]
PODXL_1	GACACCACUACAGUUGCAA[dT][dT]	UUGCAACUGUAGUGGUGUC[dT][dT]
PODXL_2	CUUCGGAUGAGAAAUUGAU[dT][dT]	AUCAAUUUCUCAUCCGAAG[dT][dT]
PODXL_3	GCUGGAUCGUCCCUCUGGA[dT][dT]	UCCAGAGGGACGAUCCAGC[dT][dT]
PON2_1	CAAUAUUUCACCUGAUGAU[dT][dT]	AUCAUCAGGUGAAAUAUUG[dT][dT]
PON2_2	CUAAUAUGAAUUUAACUCA[dT][dT]	UGAGUUAAAUUCAUAUUAG[dT][dT]
PON2_3	GAUAACUUAACAAUUAAUU[dT][dT]	AAUUAAUUGUUAAGUUAUC[dT][dT]
POSTN_1	GACAACAAAUGGUGUAAUU[dT][dT]	AAUUACACCAUUUGUUGUC[dT][dT]
POSTN_2	GAUCCAUGGAGAGCCAAUU[dT][dT]	AAUUGGCUCUCCAUGGAUC[dT][dT]
POSTN_3	GGAUCUUGUGGCCCAAUUA[dT][dT]	UAAUUGGGCCACAAGAUCC[dT][dT]
PPT1_1	GGAAUUUACGUCUUAUCUU[dT][dT]	AAGAUAAGACGUAAAUUCC[dT][dT]
PPT1_2	CGUCUUAUCUUUAGAGAUU[dT][dT]	AAUCUCUAAAGAUAAGACG[dT][dT]
PPT1_3	CUUAAGCAUGGGUGCUAUU[dT][dT]	AAUAGCACCCAUGCUUAAG[dT][dT]
PRADC1_1	CCUUCUGGUAGAAGAGUUU[dT][dT]	AAACUCUUCUACCAGAAGG[dT][dT]
PRADC1_2	CGGUGAUCAUCUCUGACAA[dT][dT]	UUGUCAGAGAUGAUCACCG[dT][dT]
PRADC1_3	GGUAGAAGAGUUUGUCCCA[dT][dT]	UGGGACAAACUCUUCUACC[dT][dT]

PRNP_1	GUGACUAUGAGGACCGUUA[dT][dT]	AUCGCUGUGUAGCUCAUAG[dT][dT]
PRNP_2	CUCAGAACAGUCUGAAAUA[dT][dT]	UACUACCUUACCUAAAUGG
PRNP_3	CAUUGGUUAAUCUGGACUU[dT][dT]	UUGACCUCAACAUGAGUAC
PROCR_1	CCAACACCACGAUCAUUCA[dT][dT]	UGAAUGAUCGUGGUGUUGG[dT][dT]
PROCR_2	CUCGGUAUGAACUGCGGGA[dT][dT]	UCCCGCAGUUCAUACCGAG[dT][dT]
PROCR_3	GUCUGAUAAGUGAAUGUUU[dT][dT]	AAACAUUCACUUAUCAGAC[dT][dT]
PRSS3_1	GAGCUUUGGUGCUGACUAC[dT][dT]	GUAGUCAGCACCAAAGCUC[dT][dT]
PRSS3_2	CUUUGGUGCUGACUACCCA[dT][dT]	UGGGUAGUCAGCACCAAAG[dT][dT]
PRSS3_3	GAGAGCACAACAUCAAAGU[dT][dT]	ACUUUGAUGUUGUGCUCUC[dT][dT]
PSAP_1	GUGAUAUGCUGAAGGACAA[dT][dT]	UUGUCCUUCAGCAUAUCAC[dT][dT]
PSAP_2	CUUCCUUCGUGUGCUUGAA[dT][dT]	UUCAAGCACACGAAGGAAG[dT][dT]
PSAP_3	GAAACCACAGCAUUGGUUU[dT][dT]	AAACCAAUGCUGUGGUUUC[dT][dT]
PTDSS1_1	CUGAAUUUCGAGCAGGUUA[dT][dT]	UAACCUGCUCGAAAUUCAG[dT][dT]
PTDSS1_2	CUUUAUUGGUGGCAUCACA[dT][dT]	UGUGAUGCCACCAAUAAAG[dT][dT]
PTDSS1_3	CUGGUUAAUCAAAGAUGUU[dT][dT]	AACAUCUUUGAUUAACCAG[dT][dT]
PTPRB_1	CACAUACGGAUGUAACCUU[dT][dT]	AAGGUUACAUCCGUAUGUG[dT][dT]
PTPRB_2	CAGUUAUUCCUUAAUAGUA[dT][dT]	UACUAUUAAGGAAUAACUG[dT][dT]
PTPRB_3	CCUAUGAUGCCACGCGAGU[dT][dT]	ACUCGCGUGGCAUCAUAGG[dT][dT]
PTPRE_1	GAAUUCCCUGUUCAGACUA[dT][dT]	UAGUCUGAACAGGGAAUUC[dT][dT]
PTPRE_2	CGUAUGACUUCAACCGAGU[dT][dT]	ACUCGGUUGAAGUCAUACG[dT][dT]
PTPRE_3	GAUAAAGAAUGAUACCCUU[dT][dT]	AAGGGUAUCAUUCUUUAUC[dT][dT]
PTPRF_1	GCUUUACCCUUACUGGCCU[dT][dT]	UAACGGUCCUCAUAGUCAC[dT][dT]
PTPRF_2	CAAGAACCGCUAUGCGAAU[dT][dT]	UGACACCUGAUAAUGCAUC
PTPRF_3	CUAUGUGCCGCAGGCGGAA[dT][dT]	UCUCACAAAUUCUUUGGUG
PTPRM_1	CUAUGCAGAGUUGGUAGUU[dT][dT]	AACUACCAACUCUGCAUAG[dT][dT]
PTPRM_2	GGCAAUUAUAUCGAUGGUU[dT][dT]	AACCAUCGAUAUAAUUGCC[dT][dT]
PTPRM_3	CAAACUCAAGCCAGAUUAA[dT][dT]	UUAAUCUGGCUUGAGUUUG[dT][dT]
PTTG1IP_1	CAGUGAACCUGCCAGCCUU[dT][dT]	AAGGCUGGCAGGUUCACUG[dT][dT]
PTTG1IP_2	GUCAUUCUCCCAGGGCAGA[dT][dT]	UCUGCCCUGGGAGAAUGAC[dT][dT]
PTTG1IP_3	CCUGUUGACUCACACGGCU[dT][dT]	AGCCGUGUGAGUCAACAGG[dT][dT]
PTX3_1	GGAUAGUGUUCUUAGCAAU[dT][dT]	AUUGCUAAGAACACUAUCC[dT][dT]
PTX3_2	GUGUUCUUAGCAAUGAAGA[dT][dT]	UCUUCAUUGCUAAGAACAC[dT][dT]
PTX3_3	GUGCAUAAUAGGAACACUU[dT][dT]	AAGUGUUCCUAUUAUGCAC[dT][dT]
PVR_1	CCAACAUGGAGGUGACGCA[dT][dT]	UGCGUCACCUCCAUGUUGG[dT][dT]
PVR_2	GGCUAUAAUUGGAGCACGA[dT][dT]	UCGUGCUCCAAUUAUAGCC[dT][dT]

PVR_3	CUAUUCAGCUGUGAGCAGA[dT][dT]	UCUGCUCACAGCUGAAUAG[dT][dT]
PVRL2_1	GAGCACUGGGCAAGACACA[dT][dT]	UGUGUCUUGCCCAGUGCUC[dT][dT]
PVRL2_2	GCCAAAGAGACUCAGGUGU[dT][dT]	ACACCUGAGUCUCUUUGGC[dT][dT]
PVRL2_3	CUGGUACCUCGGCCGUACU[dT][dT]	AGUACGGCCGAGGUACCAG[dT][dT]
PXDN_1	GAGUAUGCGUGCUCUGCGA[dT][dT]	UCGCAGAGCACGCAUACUC[dT][dT]
PXDN_2	CAGCCCUAUUUAGUAUACU[dT][dT]	AGUAUACUAAAUAGGGCUG[dT][dT]
PXDN_3	GCACUUUAAUCAGAUAGAA[dT][dT]	UUCUAUCUGAUUAAAGUGC[dT][dT]
QPCT_1	CGGUAUUUCCAGAAUUACA[dT][dT]	UGUAAUUCUGGAAAUACCG[dT][dT]
QPCT_2	GUGAUUCAGGAUGACCAUA[dT][dT]	UAUGGUCAUCCUGAAUCAC[dT][dT]
QPCT_3	CUCUGAUUUAGUUUAGGAU[dT][dT]	AUCCUAAACUAAAUCAGAG[dT][dT]
QSOX1_1	GGAUUCUUUGCGAGAAAUA[dT][dT]	AGGCCAGUAAGGGUAAAGC[dT][dT]
QSOX1_2	CCCUGUAUCUCGCCGCCCU[dT][dT]	UAUUUCAGACUGUUCUGAG
QSOX1_3	CCAAGAUCUACAUGGCUGA[dT][dT]	UCCUCUGAAAUAUUGAUCC
RHBDF1_1	GUGUGAGUAUGCCAGCCGA[dT][dT]	UCGGCUGGCAUACUCACAC[dT][dT]
RHBDF1_2	GAGCUUGCGGGCCACAAGA[dT][dT]	UCUUGUGGCCCGCAAGCUC[dT][dT]
RHBDF1_3	GUGAGUUCCUCACCUGCAU[dT][dT]	AUGCAGGUGAGGAACUCAC[dT][dT]
RNF144B_1	CUGACAUGGUGUGCCUAAA[dT][dT]	UUUAGGCACACCAUGUCAG[dT][dT]
RNF144B_2	GACAAAGGGCCAUGCAGGA[dT][dT]	UCCUGCAUGGCCCUUUGUC[dT][dT]
RNF144B_3	GAUCUCACUGGACUGUUCA[dT][dT]	UGAACAGUCCAGUGAGAUC[dT][dT]
RNPEP_1	CAAAUAAGUUCUUCUUCCA[dT][dT]	UGGAAGAAGAACUUAUUUG[dT][dT]
RNPEP_2	GACAUCACUGGAGAGGAAA[dT][dT]	UUUCCUCUCCAGUGAUGUC[dT][dT]
RNPEP_3	CUCUUCAUGCCACCGUCCU[dT][dT]	AGGACGGUGGCAUGAAGAG[dT][dT]
ROBO4_1	GUCUACACAUGUGAGGCCA[dT][dT]	UGGCCUCACAUGUGUAGAC[dT][dT]
ROBO4_2	GACACUUGGCGUUCCACCU[dT][dT]	AGGUGGAACGCCAAGUGUC[dT][dT]
ROBO4_3	GAGUUCUCCCUCCACGAUU[dT][dT]	AAUCGUGGAGGGAGAACUC[dT][dT]
RPN1_1	GCUACAACCUCCCAAGCUA[dT][dT]	UAUUUCUCGCAAAGAAUCC[dT][dT]
RPN1_2	GGAUUAUGGGCCUUUCAGA[dT][dT]	AUUCGCAUAGCGGUUCUUG
RPN1_3	GACAUUGUGGUCCACUACA[dT][dT]	AAGUCCAGAUUAACCAAUG
RPN2_1	CUUUGAGUCCCUCUCCGAA[dT][dT]	UAGCUUGGGAGGUUGUAGC[dT][dT]
RPN2_2	CAGACAACAAGAACGUGUA[dT][dT]	AGGGCGGCGAGAUACAGGG
RPN2_3	GUUAAACUAGAACAUGCUA[dT][dT]	UUCCGCCUGCGGCACAUAG
RSPRY1_1	GUAGACAGCUGACCUAUGA[dT][dT]	UCAUAGGUCAGCUGUCUAC[dT][dT]
RSPRY1_2	GCAAAUUCCUCAAUCAUGA[dT][dT]	UCAUGAUUGAGGAAUUUGC[dT][dT]
RSPRY1_3	GAUGUUGUCCAGUCUUUAA[dT][dT]	UUAAAGACUGGACAACAUC[dT][dT]
RTN4_1	CAUGUGAACUGCACGAUAA[dT][dT]	UUCGGAGAGGGACUCAAAG[dT][dT]

RTN4_2	CAUUCAGGGCAUAUCUGGA[dT][dT]	UCUGAAAGGCCCAUAAUCC
RTN4_3	CAUGUGAACUGCACGAUAA[dT][dT]	UCAGCCAUGUAGAUCUUGG
S100A10_1	GUUCGCUCCUCCCUGAUAA[dT][dT]	UUAUCGUGCAGUUCACAUG[dT][dT]
S100A10_2	CCAUUGCAUGCAAUGACUA[dT][dT]	UACACGUUCUUGUUGUCUG
S100A10_3	CUGAGAGUACUCAUGGAAA[dT][dT]	UGUAGUGGACCACAAUGUC
S1PR1_1	CUGACUACGUCAACUAUGA[dT][dT]	UCAUAGUUGACGUAGUCAG[dT][dT]
S1PR1_1	CUGACUACGUCAACUAUGA[dT][dT]	UCAUAGUUGACGUAGUCAG[dT][dT]
S1PR1_2	GUACUUCCUGGUGUUAGCU[dT][dT]	AGCUAACACCAGGAAGUAC[dT][dT]
S1PR1_2	GUACUUCCUGGUGUUAGCU[dT][dT]	AGCUAACACCAGGAAGUAC[dT][dT]
S1PR1_3	GAGUUAGUUCCUGUGAACA[dT][dT]	UGUUCACAGGAACUAACUC[dT][dT]
S1PR1_3	GAGUUAGUUCCUGUGAACA[dT][dT]	UGUUCACAGGAACUAACUC[dT][dT]
SCARA3_1	CAUGGCUUCUCACGAGAUU[dT][dT]	UUAUCAGGGAGGAGCGAAC[dT][dT]
SCARA3_2	CCUUGACCCAGUCUAUUUA[dT][dT]	UCCAGAUAUGCCCUGAAUG
SCARA3_3	CUGGAGAGGCGGUCAAGAA[dT][dT]	UAGCAUGUUCUAGUUUAAC
SCARB2_1	GUUGAAACGGGAGACAUUA[dT][dT]	AAUCUCGUGAGAAGCCAUG[dT][dT]
SCARB2_2	GAAUGGUACUGAGGCAUUU[dT][dT]	UAGUCAUUGCAUGCAAUGG
SCARB2_3	GAAUGGUGCACCCAUCAUU[dT][dT]	UUAUCGUGCAGUUCACAUG
SCARF1_1	GCCAGAUACUGGCCACUGU[dT][dT]	ACAGUGGCCAGUAUCUGGC[dT][dT]
SCARF1_2	CUCUCCAGCCCGCUCCGAA[dT][dT]	UUCGGAGCGGGCUGGAGAG[dT][dT]
SCARF1_3	CCCUGAUGACCUUGAAUUU[dT][dT]	AAAUUCAAGGUCAUCAGGG[dT][dT]
SCCPDH_1	CUGUGAUAUUGCUAAUCCA[dT][dT]	UGGAUUAGCAAUAUCACAG[dT][dT]
SCCPDH_2	CAAUUUCUUAUUGUCGGGA[dT][dT]	UCCCGACAAUAAGAAAUUG[dT][dT]
SCCPDH_3	CAUAACGUGCGUGAAUUAA[dT][dT]	UUAAUUCACGCACGUUAUG[dT][dT]
SCD_1	GAUAUGCUGUGGUGCUUAA[dT][dT]	UAAUGUCUCCCGUUUCAAC[dT][dT]
SCD_2	GAUAUCGUCCUUAUGACAA[dT][dT]	UAAAUAGACUGGGUCAAGG
SCD_3	GACGAUAUCUCUAGCUCCU[dT][dT]	UUUCCAUGAGUACUCUCAG
SCD5_1	CCCUUUCUGCGACGCCAAG[dT][dT]	CUUGGCGUCGCAGAAAGGG[dT][dT]
SCD5_2	CUGAUCCUGUGGUCCGGAU[dT][dT]	AUCCGGACCACAGGAUCAG[dT][dT]
SCD5_3	CGAGUGAAUUUGGCUUAAA[dT][dT]	UUUAAGCCAAAUUCACUCG[dT][dT]
SDCBP_1	CCAAGUACUUCAGAUCAAU[dT][dT]	UUAAGCACCACAGCAUAUC[dT][dT]
SDCBP_1	CCAAGUACUUCAGAUCAAU[dT][dT]	AUUGAUCUGAAGUACUUGG[dT][dT]
SDCBP_2	GUCAUUGGAUUGAAGGACU[dT][dT]	AGUCCUUCAAUCCAAUGAC[dT][dT]
SDCBP_2	GUGAAACUUUCAAGAGAUU[dT][dT]	AAAUGCCUCAGUACCAUUC
SDCBP_3	CUUUCAAGAGAUUUACUGA[dT][dT]	UCAGUAAAUCUCUUGAAAG[dT][dT]
SDCBP_3	GGAUUGAAGGACUCUCAAA[dT][dT]	UUCUUGACCGCCUCUCCAG

SDCBP_4	CGAUUACCAUGCAUAAGGA[dT][dT]	UCCUUAUGCAUGGUAAUCG
SDF2_1	CCACUUCACUUCACCUCUU[dT][dT]	AAGAGGUGAAGUGAAGUGG[dT][dT]
SDF2_2	CUGUGAAUCUAGAGGCUCU[dT][dT]	AGAGCCUCUAGAUUCACAG[dT][dT]
SDF2_3	GAGAUGGUGAGGUGCGGUU[dT][dT]	AACCGCACCUCACCAUCUC[dT][dT]
SEC22C_1	GGCUCAAGAGUUUAGCCUU[dT][dT]	AAGGCUAAACUCUUGAGCC[dT][dT]
SEC22C_2	GGAAUUCACAGCUUCCUAU[dT][dT]	AUAGGAAGCUGUGAAUUCC[dT][dT]
SEC22C_3	GUGAUGAAUGGUCACACAC[dT][dT]	GUGUGUGACCAUUCAUCAC[dT][dT]
SEC61A1_1	GCCUUAUAAUGCAACUCUU[dT][dT]	AUUGAUCUGAAGUACUUGG[dT][dT]
SEC61A1_2	CUUUGUUGCUGGCUUAAUU[dT][dT]	UUGUCAUAAGGACGAUAUC
SEC61A1_3	CAGUUGUAUACAUAGUGUU[dT][dT]	AAUGAUGGGUGCACCAUUC
SEL1L3_1	GUUGUACCAUCUUUGGGUA[dT][dT]	UACCCAAAGAUGGUACAAC[dT][dT]
SEL1L3_2	GCUAUUCAAGGGUCAAGGA[dT][dT]	UCCUUGACCCUUGAAUAGC[dT][dT]
SEL1L3_3	CUCAACUCUCCAUUCUAAU[dT][dT]	AUUAGAAUGGAGAGUUGAG[dT][dT]
SEMA3A_1	CCCUUUACACCGGUGUGAU[dT][dT]	AUCACACCGGUGUAAAGGG[dT][dT]
SEMA3A_2	CAAGGUACUUAAGGCAUAU[dT][dT]	AUAUGCCUUAAGUACCUUG[dT][dT]
SEMA3A_3	CUCAAUGAUCCAAAGUUCA[dT][dT]	UGAACUUUGGAUCAUUGAG[dT][dT]
SEMA3F_1	GCAUGUACGUGGGCAGCAA[dT][dT]	UUGCUGCCCACGUACAUGC[dT][dT]
SEMA3F_2	GCAAUGAUGAUAAGCUUUA[dT][dT]	UAAAGCUUAUCAUCAUUGC[dT][dT]
SEMA3F_3	CUGUUAAGUGGCUGUUCCA[dT][dT]	UGGAACAGCCACUUAACAG[dT][dT]
SEMA6B_1	CUGACGACCUCAACAUCCA[dT][dT]	UGGAUGUUGAGGUCGUCAG[dT][dT]
SEMA6B_2	CUCUUUGUGUGCGGUUCCA[dT][dT]	UGGAACCGCACACAAAGAG[dT][dT]
SEMA6B_3	GAGAUUGCGAUGGAGUUUA[dT][dT]	UAAACUCCAUCGCAAUCUC[dT][dT]
SERINC3_1	CUGAUAUAAAUGCAGAUAA[dT][dT]	AAGAGUUGCAUUAUAAGGC[dT][dT]
SERINC3_2	CCACUAAUAGCCAAGUAGA[dT][dT]	AAUCUCUUGAAAGUUUCAC
SERINC3_3	CAAACCAGAUGGCUGCACA[dT][dT]	AGGAGCUAGAGAUAUCGUC
SERPINB2_1	GGUGUUAUGACAGGGAGAA[dT][dT]	UUCUCCCUGUCAUAACACC[dT][dT]
SERPINB2_2	GGUAGUUAUCCUGAUGCGA[dT][dT]	UCGCAUCAGGAUAACUACC[dT][dT]
SERPINB2_3	GUAGUUAUCCUGAUGCGAU[dT][dT]	AUCGCAUCAGGAUAACUAC[dT][dT]
SERPINE1_1	GAAACACCCUUUCAUCUCA[dT][dT]	UGAGAUGAAAGGGUGUUUC[dT][dT]
SERPINE1_2	CCAACAUUCUGAGUGCCCA[dT][dT]	UGGGCACUCAGAAUGUUGG[dT][dT]
SERPINE1_3	GAGAGAAGUUUGAAGCACA[dT][dT]	UGUGCUUCAAACUUCUCUC[dT][dT]
SFRP1_1	CAAACCAUUUCCAACAGCA[dT][dT]	UGCUGUUGGAAAUGGUUUG[dT][dT]
SFRP1_2	GAAACAGACUCAUACCACA[dT][dT]	UGUGGUAUGAGUCUGUUUC[dT][dT]
SFRP1_3	CAUUUAGAUUAGGAAGGUU[dT][dT]	AACCUUCCUAAUCUAAAUG[dT][dT]
SIAE_1	CUGAUACGUGGAUGGUGGU[dT][dT]	ACCACCAUCCACGUAUCAG[dT][dT]

SIAE_2	CUUUCGAAGUGAUGGCACA[dT][dT]	UGUGCCAUCACUUCGAAAG[dT][dT]
SIAE_3	GCUACAAGGGAGUUGUCUA[dT][dT]	UAGACAACUCCCUUGUAGC[dT][dT]
SLC25A3_1	CUGCUAAGGUUCGAAUUCA[dT][dT]	UUAUCUGCAUUUAUAUCAG[dT][dT]
SLC25A3_2	GUCUAUUCCUGAUAUAAUU[dT][dT]	AAUUAAGCCAGCAACAAAG
SLC25A3_3	GAUUGGUACCCUGACUGCA[dT][dT]	UUUGAGAGUCCUUCAAUCC
SLC25A6_1	GCAAGCAGAUCGCCGCCGA[dT][dT]	UCGGCGGCGAUCUGCUUGC[dT][dT]
SLC25A6_2	GAGACUGCCUGGUGAAGAU[dT][dT]	AUCUUCACCAGGCAGUCUC[dT][dT]
SLC25A6_3	CACAUCGUGGUGAGCUGGA[dT][dT]	UCCAGCUCACCACGAUGUG[dT][dT]
SLC29A1_1	GGCUUAUCUUCUUCAUGCU[dT][dT]	AGCAUGAAGAAGAUAAGCC[dT][dT]
SLC29A1_2	GAAAGUGCCUUCGGCUACU[dT][dT]	AGUAGCCGAAGGCACUUUC[dT][dT]
SLC29A1_3	GUUUCUUGACUUUCAAUAU[dT][dT]	AUAUUGAAAGUCAAGAAAC[dT][dT]
SLC35F2_1	CCAGUAUUUGGCAGAAAGA[dT][dT]	UCUUUCUGCCAAAUACUGG[dT][dT]
SLC35F2_2	GCUUCAUGCCAUUGGUGAU[dT][dT]	AUCACCAAUGGCAUGAAGC[dT][dT]
SLC35F2_3	CACUCUACAACCCUGGGUU[dT][dT]	AACCCAGGGUUGUAGAGUG[dT][dT]
SLC38A1_1	GAAGUGUGAUGAGUAUAUU[dT][dT]	AAUAUACUCAUCACACUUC[dT][dT]
SLC38A1_2	GAACAAGUCUUUGGCACCA[dT][dT]	UGGUGCCAAAGACUUGUUC[dT][dT]
SLC38A1_3	CAAUUUACAGUGAGCUUAA[dT][dT]	UUAAGCUCACUGUAAAUUG[dT][dT]
SLC38A2_1	CAAGCUACCUCUUCAUAGU[dT][dT]	UGAAUUCGAACCUUAGCAG[dT][dT]
SLC38A2_2	CUGUCUAUGCUGUGCCAAU[dT][dT]	UCUACUUGGCUAUUAGUGG
SLC38A2_3	CCUUCUAUAUCAAGUUGGU[dT][dT]	AACACUAUGUAUACAACUG
SLC38A2_4	CGAACAUGUUGAGUCAGAA[dT][dT]	UUCUGACUCAACAUGUUCG
SLC7A11_1	CUCCUAAGGGCGUGCUCCA[dT][dT]	UGGAGCACGCCCUUAGGAG[dT][dT]
SLC7A11_2	GGGAAAUUUCUCAUUAGCA[dT][dT]	UGCUAAUGAGAAAUUUCCC[dT][dT]
SLC7A11_3	GAACUAAUGGACUUGAGAU[dT][dT]	AUCUCAAGUCCAUUAGUUC[dT][dT]
SLC7A7_1	GUCUCUUAACCUUCAUUAA[dT][dT]	UUAAUGAAGGUUAAGAGAC[dT][dT]
SLC7A7_2	GAUCAGAUAUUUGGAAUAU[dT][dT]	AUAUUCCAAAUAUCUGAUC[dT][dT]
SLC7A7_3	GUUUCUGGUCUACUGGCUA[dT][dT]	UAGCCAGUAGACCAGAAAC[dT][dT]
SMPDL3A_1	CUCAUGUUCCUGUACCUGA[dT][dT]	UCAGGUACAGGAACAUGAG[dT][dT]
SMPDL3A_2	CAUUAUGGUUCUUUCAGAU[dT][dT]	AUCUGAAAGAACCAUAAUG[dT][dT]
SMPDL3A_3	GAAACCAUGGCUAGAUGAA[dT][dT]	UUCAUCUAGCCAUGGUUUC[dT][dT]
SPARC_1	CUACAUCGGGCCUUGCAAA[dT][dT]	UUUGCAAGGCCCGAUGUAG[dT][dT]
SPARC_2	GCAUCAAGCAGAAGGAUAU[dT][dT]	AUAUCCUUCUGCUUGAUGC[dT][dT]
SPARC_3	GAGCUAAUCCUGUCCAGGU[dT][dT]	ACCUGGACAGGAUUAGCUC[dT][dT]
SPATA20_1	CUGCAUAUGUGUGUGAGAA[dT][dT]	UUCUCACACACAUAUGCAG[dT][dT]
SPATA20_2	CAAAGAGGGCGCCUACUAU[dT][dT]	AUAGUAGGCGCCCUCUUUG[dT][dT]

SPATA20_3	CCUACAACAUGCCUACAAU[dT][dT]	AUUGUAGGCAUGUUGUAGG[dT][dT]
SPCS3_1	GGGAUUUAUCACAUUUGAU[dT][dT]	AUCAAAUGUGAUAAAUCCC[dT][dT]
SPCS3_2	GGUCUCAAGGGAAACAGGA[dT][dT]	UCCUGUUUCCCUUGAGACC[dT][dT]
SPCS3_3	GAUAUGUUUGAUUACUGGA[dT][dT]	UCCAGUAAUCAAACAUAUC[dT][dT]
SPNS2_1	GACCUCUUCACCAAGAACA[dT][dT]	UGUUCUUGGUGAAGAGGUC[dT][dT]
SPNS2_2	GCAUGUGGAUCCCGCUCUA[dT][dT]	UAGAGCGGGAUCCACAUGC[dT][dT]
SPNS2_3	GCCAUUGGGACAAUGAAGA[dT][dT]	UCUUCAUUGUCCCAAUGGC[dT][dT]
SPOCK1_1	GAAUUCAGAAGCUGAGUAA[dT][dT]	UUACUCAGCUUCUGAAUUC[dT][dT]
SPOCK1_2	GUUCCAUGCUUGUUCUACU[dT][dT]	AGUAGAACAAGCAUGGAAC[dT][dT]
SPOCK1_3	GACAAGUACUGGAACCGCU[dT][dT]	AGCGGUUCCAGUACUUGUC[dT][dT]
SPTLC2_1	GUACUCACCCUCUUUGGAU[dT][dT]	AUCCAAAGAGGGUGAGUAC[dT][dT]
SPTLC2_2	GCUAUUGAAAGAUGCCAUU[dT][dT]	AAUGGCAUCUUUCAAUAGC[dT][dT]
SPTLC2_3	CCUUUGACGAGACGACGUA[dT][dT]	UACGUCGUCUCGUCAAAGG[dT][dT]
SRGN_1	GGACCAAUGUUCGAACUAC[dT][dT]	GUAGUUCGAACAUUGGUCC[dT][dT]
SRGN_2	GGAACAGGAUUACCAACUA[dT][dT]	UAGUUGGUAAUCCUGUUCC[dT][dT]
SRGN_3	GGACUUGAAUCGUAUCUUC[dT][dT]	GAAGAUACGAUUCAAGUCC[dT][dT]
SRPX2_1	GUCUCUUGGAUCAAUUCUA[dT][dT]	UAGAAUUGAUCCAAGAGAC[dT][dT]
SRPX2_2	GUAUUGACCGAGACCGCUA[dT][dT]	UAGCGGUCUCGGUCAAUAC[dT][dT]
SRPX2_3	CAACUAUGGUGCCACCUGU[dT][dT]	ACAGGUGGCACCAUAGUUG[dT][dT]
SSR1_1	CACAAGAUCUUACAGAGGA[dT][dT]	ACUAUGAAGAGGUAGCUUG[dT][dT]
SSR1_2	CAGAUACAACUAUACUGUU[dT][dT]	AAUUAUAUCAGGAAUAGAC
SSR1_3	CGUAAGAGACCCAUACAGA[dT][dT]	UGUGCAGCCAUCUGGUUUG
SSR3_1	GAAUAUGGCAUAUGGAUCU[dT][dT]	AGAUCCAUAUGCCAUAUUC[dT][dT]
SSR3_2	GUCCAUAAGUGCUUCAUCA[dT][dT]	UGAUGAAGCACUUAUGGAC[dT][dT]
SSR3_3	GCAAUGUGCUGUAAUCACA[dT][dT]	UGUGAUUACAGCACAUUGC[dT][dT]
STAB1_1	GACACAAGGACCCAACAAA[dT][dT]	UUUGUUGGGUCCUUGUGUC[dT][dT]
STAB1_2	GGGAUAAGCAUGCCUCAUU[dT][dT]	AAUGAGGCAUGCUUAUCCC[dT][dT]
STAB1_3	CAGUUGUGGUUAGCCGUAU[dT][dT]	AUACGGCUAACCACAACUG[dT][dT]
STC1_1	CAGAUACUAUAACAGACUU[dT][dT]	AAGUCUGUUAUAGUAUCUG[dT][dT]
STC1_2	CUUAAAUGCUCAUAAAGAU[dT][dT]	AUCUUUAUGAGCAUUUAAG[dT][dT]
STC1_3	CUUGUACAGCGCUGCUAAA[dT][dT]	UUUAGCAGCGCUGUACAAG[dT][dT]
STC2_1	GCUUACAUGGGAUUUGCAU[dT][dT]	AUGCAAAUCCCAUGUAAGC[dT][dT]
STC2_2	CGCGUUAUCCUCGUACCUU[dT][dT]	AAGGUACGAGGAUAACGCG[dT][dT]
STC2_3	CGAACAGUCUGAGUAUUCU[dT][dT]	AGAAUACUCAGACUGUUCG[dT][dT]
STOM_1	GGUAAUCUGUUAGUCUUAA[dT][dT]	UCCUCUGUAAGAUCUUGUG[dT][dT]

STOM_2	GUCUAUUACCGCGUUCAGA[dT][dT]	AUUGGCACAGCAUAGACAG
STOM_3	CCGUUAUAACUUUCCCAAU[dT][dT]	UGCAGUCAGGGUACCAAUC
STT3A_1	CUUUGGACAGGUUUACACA[dT][dT]	UUAAGACUAACAGAUUACC[dT][dT]
STT3A_2	CAAGUUGAAUCCACAACAA[dT][dT]	AACAGUAUAGUUGUAUCUG
STT3A_3	GGAACAAUUUACCCAGGUU[dT][dT]	ACCAACUUGAUAUAGAAGG
STT3B_1	CUACAUAUCUCGGUCAGUA[dT][dT]	UGUGUAAACCUGUCCAAAG[dT][dT]
STT3B_2	CUACUUUGUUGAAUUGCCU[dT][dT]	UCUGAACGCGGUAAUAGAC
STT3B_3	CUGUUUACCCAGGGUUGAU[dT][dT]	UCUGUAUGGGUCUCUUACG
STT3B_4	GCUAUUGUACCAGGCUACA[dT][dT]	UGUAGCCUGGUACAAUAGC
STX12_1	CAAAUCAGCUCGCCAAGGA[dT][dT]	UCCUUGGCGAGCUGAUUUG[dT][dT]
STX12_2	CACUGAACAGUUACAGCGA[dT][dT]	UCGCUGUAACUGUUCAGUG[dT][dT]
STX12_3	GCCUAUUCUCUAUCUAUUU[dT][dT]	AAAUAGAUAGAGAAUAGGC[dT][dT]
STX3_1	GGAAACUCGGCUUAACAUU[dT][dT]	AAUGUUAAGCCGAGUUUCC[dT][dT]
STX3_2	GGAAGUUUGUGGAGGUGAU[dT][dT]	AUCACCUCCACAAACUUCC[dT][dT]
STX3_3	GAAUCAGGGUGAGAUGUUA[dT][dT]	UAACAUCUCACCCUGAUUC[dT][dT]
STX4_1	GCAAUUCAAUGCAGUCCGA[dT][dT]	UCGGACUGCAUUGAAUUGC[dT][dT]
STX4_2	GCUUGAACGCAGUAUUCGU[dT][dT]	ACGAAUACUGCGUUCAAGC[dT][dT]
STX4_3	GUGGUUGGAUAAUGUCGCA[dT][dT]	UGCGACAUUAUCCAACCAC[dT][dT]
STX7_1	CUGAAUUGAGGCAACAGUU[dT][dT]	AACUGUUGCCUCAAUUCAG[dT][dT]
STX7_2	GAGUUCACAACAUCACUGA[dT][dT]	UCAGUGAUGUUGUGAACUC[dT][dT]
STX7_3	CUUCUAUCAGGCAACUUGA[dT][dT]	UCAAGUUGCCUGAUAGAAG[dT][dT]
SYNJ2_1	CAAACGAUGGGUCUCGCUU[dT][dT]	AAGCGAGACCCAUCGUUUG[dT][dT]
SYNJ2_2	CUACAGAAAUCAAGUGGAA[dT][dT]	UUCCACUUGAUUUCUGUAG[dT][dT]
SYNJ2_3	GGUGUUUGACCCACUGGCA[dT][dT]	UGCCAGUGGGUCAAACACC[dT][dT]
SYT11_1	CAAUAUCCGACCUAGCUUU[dT][dT]	AAAGCUAGGUCGGAUAUUG[dT][dT]
SYT11_2	GGACUAUAACUUCCCGAAA[dT][dT]	UUUCGGGAAGUUAUAGUCC[dT][dT]
SYT11_3	GUGAAGAAGUGCACUUUGA[dT][dT]	UCAAAGUGCACUUCUUCAC[dT][dT]
TCN2_1	GAGUUAGGAGGAUUCACAU[dT][dT]	AUGUGAAUCCUCCUAACUC[dT][dT]
TCN2_2	GUCUGAAGCGCUCAAACUU[dT][dT]	AAGUUUGAGCGCUUCAGAC[dT][dT]
TCN2_3	GCAUUACAGUUCCUCAUGA[dT][dT]	UCAUGAGGAACUGUAAUGC[dT][dT]
TCTN1_1	GAUAUGGACAGCUUACUAU[dT][dT]	AUAGUAAGCUGUCCAUAUC[dT][dT]
TCTN1_2	CGUGGAUUUCAGUGUCUUU[dT][dT]	AAAGACACUGAAAUCCACG[dT][dT]
TCTN1_3	CUGCAUUAUCCUUUAUUAA[dT][dT]	UUAAUAAAGGAUAAUGCAG[dT][dT]
TEK_1	CUCUUUAGCCAGCUUAGUU[dT][dT]	UACUGACCGAGAUAUGUAG[dT][dT]
TEK_2	CUCUGUACUGCUUGUAUGA[dT][dT]	UUGUUGUGGAUUCAACUUG

TEK_3	CCUAAACAGGAAGGUCAAA[dT][dT]	AUUGGGAAAGUUAUAACGG
TFPI_1	CCACUGAAACUUAUGCAUU[dT][dT]	AAUGCAUAAGUUUCAGUGG[dT][dT]
TFPI_2	CUGUAUGCCUGCUGCUUAA[dT][dT]	UUAAGCAGCAGGCAUACAG[dT][dT]
TFPI_3	GCACUUUGGGCUUCUGUAU[dT][dT]	AUACAGAAGCCCAAAGUGC[dT][dT]
TGFB1_1	GCAACAAUUCCUGGCGAUA[dT][dT]	UAUCGCCAGGAAUUGUUGC[dT][dT]
TGFB1_2	GGUGGAAACCCACAACGAA[dT][dT]	UUCGUUGUGGGUUUCCACC[dT][dT]
TGFB1_3	CACUGCAAGUGGACAUCAA[dT][dT]	UUGAUGUCCACUUGCAGUG[dT][dT]
TGFBI_1	CAAGGAAUUUGCUUCGGAA[dT][dT]	UUCCGAAGCAAAUUCCUUG[dT][dT]
TGFBI_2	CUAAUGGGAUUGUAACUGU[dT][dT]	ACAGUUACAAUCCCAUUAG[dT][dT]
TGFBI_3	GAAGGUUAUUGGCACUAAU[dT][dT]	AUUAGUGCCAAUAACCUUC[dT][dT]
TGFBR2_1	GAAAUGACAUCUCGCUGUA[dT][dT]	AACUAAGCUGGCUAAAGAG[dT][dT]
TGFBR2_1	CCAUCAUCCUGGAAGAUGA[dT][dT]	UCAUCUUCCAGGAUGAUGG[dT][dT]
TGFBR2_2	GAAAUGACAUCUCGCUGUA[dT][dT]	UACAGCGAGAUGUCAUUUC[dT][dT]
TGFBR2_2	CCCUAAACACUACCAAAUA[dT][dT]	AGGCAAUUCAACAAAGUAG
TGFBR2_3	CCCUAAACACUACCAAAUA[dT][dT]	UAUUUGGUAGUGUUUAGGG[dT][dT]
TGFBR2_3	CCAAUAUCCUCGUGAAGAA[dT][dT]	AACCUGGGUAAAUUGUUCC
TGFBR2_4	GCAUGAAGGACAACGUGUU[dT][dT]	AACACGUUGUCCUUCAUGC
TGOLN2_1	GACCUUUAUCCGAACGGUU[dT][dT]	UACAGCGAGAUGUCAUUUC[dT][dT]
TGOLN2_2	CUCAUCACAACAAGCGGAA[dT][dT]	UCAUACAAGCAGUACAGAG
TGOLN2_3	CUAACAAGUCGGGUGCGGA[dT][dT]	AUCAACCCUGGGUAAACAG
THSD1_1	CACUAAAGUUUGAGUGCUU[dT][dT]	AAGCACUCAAACUUUAGUG[dT][dT]
THSD1_2	CUACCAAAGUGUAUCACGU[dT][dT]	ACGUGAUACACUUUGGUAG[dT][dT]
THSD1_3	GUCUCAAGGAAGCAGUCUU[dT][dT]	AAGACUGCUUCCUUGAGAC[dT][dT]
THSD7A_1	GGAAGAAUAAAGAACGGGA[dT][dT]	UCCCGUUCUUUAUUCUUCC[dT][dT]
THSD7A_2	CAGACAACCAGCUGAUGAA[dT][dT]	UUCAUCAGCUGGUUGUCUG[dT][dT]
THSD7A_3	GACUAAAGACCUGGGUUUA[dT][dT]	UAAACCCAGGUCUUUAGUC[dT][dT]
TIE1_1	GUUAAUGAACCUGAGGCCA[dT][dT]	UGGCCUCAGGUUCAUUAAC[dT][dT]
TIE1_2	GAUGAACGCAGCCAUCAAA[dT][dT]	UUUGAUGGCUGCGUUCAUC[dT][dT]
TIE1_3	CUUACAGCCUCUGACUUAA[dT][dT]	UUAAGUCAGAGGCUGUAAG[dT][dT]
TIMM22_1	CGAUGGAAAGCUGCGCUUU[dT][dT]	AAAGCGCAGCUUUCCAUCG[dT][dT]
TIMM22_2	CCAUUGUGGGAGCCAUGUU[dT][dT]	AACAUGGCUCCCACAAUGG[dT][dT]
TIMM22_3	CAGAGGACGUCCCGUGCCA[dT][dT]	UGGCACGGGACGUCCUCUG[dT][dT]
TIMP1_1	CCACAGACGGCCUUCUGCA[dT][dT]	UGCAGAAGGCCGUCUGUGG[dT][dT]
TIMP1_2	CACCUUAUACCAGCGUUAU[dT][dT]	AUAACGCUGGUAUAAGGUG[dT][dT]
TIMP1_3	CAUGGAGAGUGUCUGCGGA[dT][dT]	UCCGCAGACACUCUCCAUG[dT][dT]

TIMP2_1	GGUUUCGACUGGUCCAGCU[dT][dT]	AGCUGGACCAGUCGAAACC[dT][dT]
TIMP2_2	GUAUGAGAUCAAGCAGAUA[dT][dT]	UAUCUGCUUGAUCUCAUAC[dT][dT]
TIMP2_3	CUCUGGAUGGACUGGGUCA[dT][dT]	UGACCCAGUCCAUCCAGAG[dT][dT]
TIMP3_1	GGUUGUAACUGCAAGAUCA[dT][dT]	UGAUCUUGCAGUUACAACC[dT][dT]
TIMP3_2	CUCCAUUUGAGGAUUGUAA[dT][dT]	UUACAAUCCUCAAAUGGAG[dT][dT]
TIMP3_3	GCUGAAAGGCCAACCAUUU[dT][dT]	AAAUGGUUGGCCUUUCAGC[dT][dT]
TINAGL1_1	GAGAGGAGACGCUGCCAGA[dT][dT]	UCUGGCAGCGUCUCCUCUC[dT][dT]
TINAGL1_2	GCAUGGAGGACAUGGGUCA[dT][dT]	UGACCCAUGUCCUCCAUGC[dT][dT]
TINAGL1_3	GGACAUGGGUCAUCACUGA[dT][dT]	UCAGUGAUGACCCAUGUCC[dT][dT]
TLR4_1	CCUAAUAUUACUUAUCAAU[dT][dT]	AUUGAUAAGUAAUAUUAGG[dT][dT]
TLR4_2	GCAACAUUUAGAAUUAGUU[dT][dT]	AACUAAUUCUAAAUGUUGC[dT][dT]
TLR4_3	CUUCACUACAGAGACUUUA[dT][dT]	UAAAGUCUCUGUAGUGAAG[dT][dT]
TM2D2_1	CACUACUUCAUAACCACUU[dT][dT]	AAGUGGUUAUGAAGUAGUG[dT][dT]
TM2D2_2	GAAUCAAAUUUGUUCCUUU[dT][dT]	AAAGGAACAAAUUUGAUUC[dT][dT]
TM2D2_3	GGAUUGUAAUACACACUGA[dT][dT]	UCAGUGUGUAUUACAAUCC[dT][dT]
TM4SF1_1	CUCCUGUGCAUCGCGGCUA[dT][dT]	UAGCCGCGAUGCACAGGAG[dT][dT]
TM4SF1_2	CUUGUGUCUUAUUCAAGUA[dT][dT]	UACUUGAAUAAGACACAAG[dT][dT]
TM4SF1_3	GCCCUUUGAACUGCCUUGU[dT][dT]	ACAAGGCAGUUCAAAGGGC[dT][dT]
TM4SF18_1	GUGAACAUAUUAUUGUAUU[dT][dT]	AAUACAAUAAUAUGUUCAC[dT][dT]
TM4SF18_2	GGACGUUUCCUUACAGAUU[dT][dT]	AAUCUGUAAGGAAACGUCC[dT][dT]
TM4SF18_3	GCUAUUCAGUGAUCUUCCA[dT][dT]	UGGAAGAUCACUGAAUAGC[dT][dT]
TM9SF2_1	GUUAUACCAUGAUAAUGGU[dT][dT]	AACCGUUCGGAUAAAGGUC[dT][dT]
TM9SF2_2	GCUAUGUUGCUGCCAGAUU[dT][dT]	UAUUUGGUAGUGUUUAGGG
TM9SF2_3	GAAUACACAGCGUUUGAUU[dT][dT]	UUUGACCUUCCUGUUUAGG
TM9SF3_1	GAUAAUGUGUGGAGCGAUU[dT][dT]	ACCAUUAUCAUGGUAUAAC[dT][dT]
TM9SF3_2	GUUCAUGGAGCCUGCGGUU[dT][dT]	UUCCGCUUGUUGUGAUGAG
TM9SF3_3	GAAACUGGUUCCAAAUACU[dT][dT]	UUCUUCACGAGGAUAUUGG
TMCC3_1	CAAACAUCGCGCGAUGGGA[dT][dT]	UCCCAUCGCGCGAUGUUUG[dT][dT]
TMCC3_2	CACUUACUCCACCUGUGUU[dT][dT]	AACACAGGUGGAGUAAGUG[dT][dT]
TMCC3_3	GACCAUAUCCUGUGUGCCA[dT][dT]	UGGCACACAGGAUAUGGUC[dT][dT]
TMCO3_1	CUGUUUCAGGUGCACACGU[dT][dT]	ACGUGUGCACCUGAAACAG[dT][dT]
TMCO3_2	GGAAGAAGAGGCCAAUUCU[dT][dT]	AGAAUUGGCCUCUUCUUCC[dT][dT]
TMCO3_3	CACUGUUAAUGAUUGCAUU[dT][dT]	AAUGCAAUCAUUAACAGUG[dT][dT]
TMED10_1	GAAAUUGAUUGAGUAAUGA[dT][dT]	AAUCGCUCCACACAUUAUC[dT][dT]
TMED10_2	GAUACCAACGAGUCAACAA[dT][dT]	AAUCUGGCAGCAACAUAGC

TMED10_3	GAUUCACAAGGACCUGCUA[dT][dT]	UCCGCACCCGACUUGUUAG
TMED2_1	GAGAAUCCAGUGGGAAAUA[dT][dT]	UCAUUACUCAAUCAAUUUC[dT][dT]
TMED2_2	CUGAUGAUCCCAACUCAGA[dT][dT]	AACCGCAGGCUCCAUGAAC
TMED2_3	GUGAUGUUCACCAUUGAUA[dT][dT]	AAUCAAACGCUGUGUAUUC
TMEM109_1	CAGAGACCAUGCACCUGGU[dT][dT]	ACCAGGUGCAUGGUCUCUG[dT][dT]
TMEM109_2	CACACACCGCCAGUGUCAU[dT][dT]	AUGACACUGGCGGUGUGUG[dT][dT]
TMEM109_3	CUCCUGUCCUUCUGAAGUU[dT][dT]	AACUUCAGAAGGACAGGAG[dT][dT]
TMEM123_1	CAGAUUGAUGCUGCCCUAU[dT][dT]	UAUUUCCCACUGGAUUCUC[dT][dT]
TMEM123_2	CACACAACUCCAGUGCUAA[dT][dT]	UUGUUGACUCGUUGGUAUC
TMEM123_3	CAGAUAUCAACAUCCACAA[dT][dT]	AGUAUUUGGAACCAGUUUC
TMEM173_1	CUGCAUCCAUCCAUCCCGU[dT][dT]	ACGGGAUGGAUGGAUGCAG[dT][dT]
TMEM173_2	CAUUCGCUUCCUGGAUAAA[dT][dT]	UUUAUCCAGGAAGCGAAUG[dT][dT]
TMEM173_3	GCCUCAUUGCCUACCAGGA[dT][dT]	UCCUGGUAGGCAAUGAGGC[dT][dT]
TMEM184B_1	CCAUCUCUGGCUUCUUCGU[dT][dT]	ACGAAGAAGCCAGAGAUGG[dT][dT]
TMEM184B_2	CAGUGCUCACGCCGCCUUU[dT][dT]	AAAGGCGGCGUGAGCACUG[dT][dT]
TMEM184B_3	CCACCUUUGUCCAGUGCUA[dT][dT]	UAGCACUGGACAAAGGUGG[dT][dT]
TMEM30A_1	CAUUUGAGGGCAACGUGUU[dT][dT]	AACACGUUGCCCUCAAAUG[dT][dT]
TMEM30A_2	GUAUUGCUUGGUGGACAGA[dT][dT]	UCUGUCCACCAAGCAAUAC[dT][dT]
TMEM30A_3	CAUUACCAGCUGGCCGAUA[dT][dT]	UAUCGGCCAGCUGGUAAUG[dT][dT]
TMEM44_1	GCAGCUAUUGACUUAGUGA[dT][dT]	UCACUAAGUCAAUAGCUGC[dT][dT]
TMEM44_2	CACUGCAAGUCACUGAGGA[dT][dT]	UCCUCAGUGACUUGCAGUG[dT][dT]
TMEM44_3	GCAAAUGUGUCUGGUUAGA[dT][dT]	UCUAACCAGACACAUUUGC[dT][dT]
TMEM50A_1	GCAAUACUAUUGCUUCCAU[dT][dT]	AUGGAAGCAAUAGUAUUGC[dT][dT]
TMEM50A_2	GUAGUCUCAGCUUAAAGUU[dT][dT]	AACUUUAAGCUGAGACUAC[dT][dT]
TMEM50A_3	GAUUUGUCAAUGUAAGUAU[dT][dT]	AUACUUACAUUGACAAAUC[dT][dT]
TMX3_1	CUGUAAAGGAUUUGUAGAA[dT][dT]	UUCUACAAAUCCUUUACAG[dT][dT]
TMX3_2	CUAAUUCGGCCACUUCCAA[dT][dT]	UUGGAAGUGGCCGAAUUAG[dT][dT]
TMX3_3	GUUAUAUAGAAGAACGAUA[dT][dT]	UAUCGUUCUUCUAUAUAAC[dT][dT]
TNFRSF10B_1	CUCAGAAGACGGUAGAGAU[dT][dT]	AUCUCUACCGUCUUCUGAG[dT][dT]
TNFRSF10B_2	CAGACUUGGUGCCCUUUGA[dT][dT]	UCAAAGGGCACCAAGUCUG[dT][dT]
TNFRSF10B_3	GAUGGAACAUCCUGUAACU[dT][dT]	AGUUACAGGAUGUUCCAUC[dT][dT]
TNFRSF10C_1	CCCUAAAGUUCGUCGUCGU[dT][dT]	ACGACGACGAACUUUAGGG[dT][dT]
TNFRSF10C_2	GGAUCAUAGUUCUAAUUGU[dT][dT]	ACAAUUAGAACUAUGAUCC[dT][dT]
TNFRSF10C_3	GCUCUAUCUUCCUCCUUGU[dT][dT]	ACAAGGAGGAAGAUAGAGC[dT][dT]
TNFRSF10D_1	GCUUCCAACAAUUUGCCUU[dT][dT]	AAGGCAAAUUGUUGGAAGC[dT][dT]

TNFRSF10D_2	CUCCCUAUCACUACCUUAU[dT][dT]	AUAAGGUAGUGAUAGGGAG[dT][dT]
TNFRSF10D_3	CCAACUGGUGGGCUCCGAA[dT][dT]	UUCGGAGCCCACCAGUUGG[dT][dT]
TNFRSF11A_1	GAUAAAUGCUUGCUGCAUA[dT][dT]	UAUGCAGCAAGCAUUUAUC[dT][dT]
TNFRSF11A_2	CAGAGAAAUCCGAUGCGGU[dT][dT]	ACCGCAUCGGAUUUCUCUG[dT][dT]
TNFRSF11A_3	GUGUCUUACUGCUGACUCU[dT][dT]	AGAGUCAGCAGUAAGACAC[dT][dT]
TNFRSF1B_1	GCACAUGCCGGCUCAGAGA[dT][dT]	UCUCUGAGCCGGCAUGUGC[dT][dT]
TNFRSF1B_2	GGGUCUACUAAUAAUAGGA[dT][dT]	UCCUAUUAUUAGUAGACCC[dT][dT]
TNFRSF1B_3	CAGUGCUCCUCCCAAGCCA[dT][dT]	UGGCUUGGGAGGAGCACUG[dT][dT]
TNFSF10_1	GAACCUCUGAGGAAACCAU[dT][dT]	AUGGUUUCCUCAGAGGUUC[dT][dT]
TNFSF10_2	GCAACUUGCACUUGAGGAA[dT][dT]	UUCCUCAAGUGCAAGUUGC[dT][dT]
TNFSF10_3	GUCUAAAGAUGCAGAAUAU[dT][dT]	AUAUUCUGCAUCUUUAGAC[dT][dT]
TNFSF12_1	CGCCUUUCCUGAACCGACU[dT][dT]	AGUCGGUUCAGGAAAGGCG[dT][dT]
TNFSF12_2	GCCAGGAUCCUGCGCCUUU[dT][dT]	AAAGGCGCAGGAUCCUGGC[dT][dT]
TNFSF12_3	GCGCCUUUCCUGAACCGAC[dT][dT]	GUCGGUUCAGGAAAGGCGC[dT][dT]
TNFSF15_1	GAGGAUUGGCGAGUUUCUA[dT][dT]	UAGAAACUCGCCAAUCCUC[dT][dT]
TNFSF15_2	CCAUCUACCUCGGAGCCAU[dT][dT]	AUGGCUCCGAGGUAGAUGG[dT][dT]
TNFSF15_3	GGAGUUUGCACCUUCACAU[dT][dT]	AUGUGAAGGUGCAAACUCC[dT][dT]
TOR2A_1	CAAGUGAGAUGAACGGAGU[dT][dT]	ACUCCGUUCAUCUCACUUG[dT][dT]
TOR2A_2	GAGCCAUGUUCCUAUGUCA[dT][dT]	UGACAUAGGAACAUGGCUC[dT][dT]
TOR2A_3	CCCUCUUCCUCUUCGAUGA[dT][dT]	UCAUCGAAGAGGAAGAGGG[dT][dT]
TRAM2_1	GUCAAACACAGCAAGUUCA[dT][dT]	UGAACUUGCUGUGUUUGAC[dT][dT]
TRAM2_2	GAGAAACUGUUCAGUGCCU[dT][dT]	AGGCACUGAACAGUUUCUC[dT][dT]
TRAM2_3	CUUUCUGCUUGCUCUGUCU[dT][dT]	AGACAGAGCAAGCAGAAAG[dT][dT]
TSKU_1	GGCCUAUGUGGCAGCGUCA[dT][dT]	UGACGCUGCCACAUAGGCC[dT][dT]
TSKU_2	CCCUCUAGCUGUCAUUGGU[dT][dT]	ACCAAUGACAGCUAGAGGG[dT][dT]
TSKU_3	CGAGUAACUUAUGUUCAAU[dT][dT]	AUUGAACAUAAGUUACUCG[dT][dT]
TSPAN14_1	CCAUCACUUCUGAGGAGCA[dT][dT]	AUAGGGCAGCAUCAAUCUG[dT][dT]
TSPAN14_2	CAGUGUGGAUAUGAUGUCA[dT][dT]	UCUGAGUUGGGAUCAUCAG
TSPAN14_3	GAGUCUACCCAGAGACAGA[dT][dT]	UAGCAGGUCCUUGUGAAUC
TSPAN15_1	GUUGAGCGGCAGAAAUAUA[dT][dT]	UAUAUUUCUGCCGCUCAAC[dT][dT]
TSPAN15_2	CCGAGAUUGGAGCAAGAAU[dT][dT]	AUUCUUGCUCCAAUCUCGG[dT][dT]
TSPAN15_3	CUCUGCUCAGGGCCCAUUU[dT][dT]	AAAUGGGCCCUGAGCAGAG[dT][dT]
TSPAN18_1	CGGUGAUCCUCAACACCUU[dT][dT]	UGCUCCUCAGAAGUGAUGG[dT][dT]
TSPAN18_2	GAACAAGUGUCUGCUGCUA[dT][dT]	UUAGCACUGGAGUUGUGUG
TSPAN18_3	CCUCUAAUCUCCUCCUGCU[dT][dT]	UAUCAAUGGUGAACAUCAC

TSPAN3_1	CUAUUCAGCUGCUGGGCAU[dT][dT]	AAGGUGUUGAGGAUCACCG[dT][dT]
TSPAN3_2	CAUGUACACUGGUGUUGGA[dT][dT]	UGACAUCAUAUCCACACUG
TSPAN3_3	GAACUCUUGGCCAAAGGUU[dT][dT]	UUGUGGAUGUUGAUAUCUG
TSPAN5_1	GUCAGUUGUUGCAUCAAAU[dT][dT]	AUUUGAUGCAACAACUGAC[dT][dT]
TSPAN5_2	CUGAUGAUUGGAACCUAAA[dT][dT]	UUUAGGUUCCAAUCAUCAG[dT][dT]
TSPAN5_3	CCAGAAUUUGGUUAGCGAU[dT][dT]	AUCGCUAACCAAAUUCUGG[dT][dT]
TSPAN6_1	CAAUGUUUCUGACUCUCGU[dT][dT]	ACGAGAGUCAGAAACAUUG[dT][dT]
TSPAN6_2	CUGUAAACUUGAAGAUUGU[dT][dT]	ACAAUCUUCAAGUUUACAG[dT][dT]
TSPAN6_3	GAACUGACAACACUACUUA[dT][dT]	UAAGUAGUGUUGUCAGUUC[dT][dT]
TTYH3_1	GCCAGAACGCUAAUUUCCA[dT][dT]	AUGCCCAGCAGCUGAAUAG[dT][dT]
TTYH3_2	CGACUUCUGUGUGGACCCU[dT][dT]	UAGCAGCAGACACUUGUUC
TTYH3_3	GACCAGUGAUGGCAUCCAU[dT][dT]	UCUGUCUCUGGGUAGACUC
UBE2J1_1	CCUUUAGAGGAUAACCUUU[dT][dT]	AAAGGUUAUCCUCUAAAGG[dT][dT]
UBE2J1_2	CAGACUUAAACCACUCUUU[dT][dT]	AAAGAGUGGUUUAAGUCUG[dT][dT]
UBE2J1_3	CUUAUGAGCUGUGACUCAA[dT][dT]	UUGAGUCACAGCUCAUAAG[dT][dT]
VAMP3_1	GUCACUAAUUAAUUGCCAU[dT][dT]	UGGAAAUUAGCGUUCUGGC[dT][dT]
VAMP3_2	GACUUAGAACCUGCUAUAU[dT][dT]	UCCAACACCAGUGUACAUG
VAMP3_3	CCAACUUAUGCCUUCCAGA[dT][dT]	AGCAGGAGGAGAUUAGAGG
VAMP5_1	CGGAAAUUAUGCGUAACAA[dT][dT]	UUGUUACGCAUAAUUUCCG[dT][dT]
VAMP5_2	GAGAACAUCCGUUACCGGA[dT][dT]	UCCGGUAACGGAUGUUCUC[dT][dT]
VAMP5_3	CAGUGAGUGCCAAAGGGCA[dT][dT]	UGCCCUUUGGCACUCACUG[dT][dT]
VAMP8_1	CCCAGAAUGUGGAGCGGAU[dT][dT]	AUCCGCUCCACAUUCUGGG[dT][dT]
VAMP8_2	GUCCUUAUCUGCGUGAUUG[dT][dT]	CAAUCACGCAGAUAAGGAC[dT][dT]
VAMP8_3	CUCCAUAAAUGUGUGCCAA[dT][dT]	UUGGCACACAUUUAUGGAG[dT][dT]
VANGL1_1	GAAUCCACUUAUUCUGGAU[dT][dT]	AUCCAGAAUAAGUGGAUUC[dT][dT]
VANGL1_2	GCAAUAUGCAGUCUCCCUU[dT][dT]	AAGGGAGACUGCAUAUUGC[dT][dT]
VANGL1_3	CAUGAACGGCGAGUAAAGA[dT][dT]	UCUUUACUCGCCGUUCAUG[dT][dT]
VASH1_1	GCGACAUGCGGCUCAAGAU[dT][dT]	AUCUUGAGCCGCAUGUCGC[dT][dT]
VASH1_2	GCGAUGACUUCCGCAAGGA[dT][dT]	UCCUUGCGGAAGUCAUCGC[dT][dT]
VASH1_3	GGAACUACUUCCGCCACAU[dT][dT]	AUGUGGCGGAAGUAGUUCC[dT][dT]
VEGFA_1	CUCCGAAACCAUGAACUUU[dT][dT]	AAAGUUCAUGGUUUCGGAG[dT][dT]
VEGFA_2	GAGAUCGAGUACAUCUUCA[dT][dT]	UGAAGAUGUACUCGAUCUC[dT][dT]
VEGFA_3	GGCAGAAUCAUCACGAAGU[dT][dT]	ACUUCGUGAUGAUUCUGCC[dT][dT]
VEGFB_1	CUGAGGCCAUCAUCAAACA[dT][dT]	UGUUUGAUGAUGGCCUCAG[dT][dT]
VEGFB_2	GUGACUUGCCUCAGAGGCU[dT][dT]	AGCCUCUGAGGCAAGUCAC[dT][dT]

VEGFB_3	CAAAGAGGAGCCUGGUAAA[dT][dT]	UUUACCAGGCUCCUCUUUG[dT][dT]
VEGFC_1	CACCAAACAUGCAGCUGUU[dT][dT]	AACAGCUGCAUGUUUGGUG[dT][dT]
VEGFC_2	CCAAUUACAUGUGGAAUAA[dT][dT]	UUAUUCCACAUGUAAUUGG[dT][dT]
VEGFC_3	GACAGAAACUCAUGCCAGU[dT][dT]	ACUGGCAUGAGUUUCUGUC[dT][dT]
VOPP1_1	CUGAUGAUGGGCGUGCUUU[dT][dT]	AAAGCACGCCCAUCAUCAG[dT][dT]
VOPP1_2	CAGCUCUCUUUCAGGCUUU[dT][dT]	AAAGCCUGAAAGAGAGCUG[dT][dT]
VOPP1_3	CUCUCGUCAGCCUUCCUCU[dT][dT]	AGAGGAAGGCUGACGAGAG[dT][dT]
VWCE_1	GGAAAUGUGGCAUUCAGCA[dT][dT]	UGCUGAAUGCCACAUUUCC[dT][dT]
VWCE_2	GUGUACAGACAUUGACGAA[dT][dT]	UUCGUCAAUGUCUGUACAC[dT][dT]
VWCE_3	GCAACUACGAGGGAAGGAA[dT][dT]	UUCCUUCCCUCGUAGUUGC[dT][dT]
VWF_1	CAGAAACGCUCCUUCUCGA[dT][dT]	UCGAGAAGGAGCGUUUCUG[dT][dT]
VWF_2	CAAAGAGUCUCCAUGCCCU[dT][dT]	AGGGCAUGGAGACUCUUUG[dT][dT]
VWF_3	GAAAGAAAGUCACCUUGAA[dT][dT]	UUCAAGGUGACUUUCUUUC[dT][dT]
WLS_1	GCUAAACAACCAAAUCAGA[dT][dT]	UCUGAUUUGGUUGUUUAGC[dT][dT]
WLS_2	GCUGUUUGGUGACAUCCGA[dT][dT]	UCGGAUGUCACCAAACAGC[dT][dT]
WLS_3	GUUCAAGUUCCUCAUGCUU[dT][dT]	AAGCAUGAGGAACUUGAAC[dT][dT]
YIPF5_1	CAGUAUGCUGGCUAUGACU[dT][dT]	AGUCAUAGCCAGCAUACUG[dT][dT]
YIPF5_2	GAUAUUGUCUUCUGCCCAU[dT][dT]	AUGGGCAGAAGACAAUAUC[dT][dT]
YIPF5_3	CAUAUCCUUGCGCUUUGUU[dT][dT]	AACAAAGCGCAAGGAUAUG[dT][dT]

Supplementary Table 3. Antibodies used in the study.

**FLOW CYTOMETRY**					
Flow cytometry analysis of tumor cell dormancy and survival in vitro					
Target protein	Conjugated	Species Reactivity	Company	Catalog #	Dilution
CD31	Pe-Cy7	H, Cy, Rh	Biolegend	303117	1:400
CD31	Pe-Cy7	M	Biolegend	102418	1:400
Endothelial cell sorting					
EC-Pear1-KO and EC-Ccl2-KO experiments					
Target protein	Conjugated	Species Reactivity	Company	Catalog #	Dilution
CD31	Pe-Cy7	H, Cy, Rh	Biolegend	303117	1:400
CD45	FITC	M	BD Biosciences	553079	1:400
EC-Pear1-KO (MMTV-PyMT mCherry)					
Target protein	Conjugated	Species Reactivity	Company	Catalog #	Dilution
CD31	Pe-Cy7	M	Biolegend	102418	1:400
CD45	eFlour450	H, M	eBioscience	48-0451-82	1:400
Flow cytometry analysis of tumor cell dormancy and survival in vivo					
EC-Pear1-KO and shCTSD experiments(tumor dormancy analysis)					
Target protein	Conjugated	Species Reactivity	Company	Catalog #	Dilution
CD31	Pe-Cy7	M	Biolegend	102418	1:400
CD45	eFlour450	H, M	eBioscience	48-0451-82	1:400
EC-Pear1-KO cell death analysis)					
Target protein	Conjugated	Species Reactivity	Company	Catalog #	Dilution
CD31	Pe-Cy7	M	Biolegend	102418	1:400
CD45	Pe-Cy7	H, M	eBioscience	25-0451-82	1:400
EC-Pear1-KO (MMTV-PyMT/mCherry)					
Target protein	Conjugated	Species Reactivity	Company	Catalog #	Dilution
mCherry	None		Abcam	ab205402	1:200
IgY (H+L)	AF594	C	ThermoFisher	A-11042	1:500
Ki67	Pe-Cy7	M	Biolegend	652425	1:200
IgG2a	Pe-Cy7	Rt	Biolegend	400522	1:200
Detection of circulating tumor cells in PyMT mice					
Target protein	Conjugated	Species Reactivity	Company	Catalog #	Dilution
CD45	FITC	M	BD Biosciences	553079	1:400
CTSD treatment experiments					

Target	Conjugated	Species Reactivity	Company	Catalog #	Dilution
6x-His tag	PE		Abcam	EPR20547	1:100
**IMAGING**					
Immunostaining of in vitro co-culture samples and immunohistochemistry of mouse lungs					
Target	Conjugated	Species Reactivity	Company	Catalog #	Dilution
Venus	None		ThermoFisher	OSE00002W	1:200
Ki67	None	H, M, Mk, D, Rt	Invitrogen	14-5698-82	1:200
mCherry	None		Abcam	ab205402	1:200
IgG (H+L)	AF488	Rb	ThermoFisher	A-11008	1:500
IgG (H+L)	AF647	Rt	ThermoFisher	A-21247	1:500
IgY (H+L)	AF594	C	ThermoFisher	A-11042	1:500
Immunohistochemistry of mouse lungs (Extravasation)					
Target protein	Conjugated	Species Reactivity	Company	Catalog #	Dilution
GFP	None		Abcam	ab32146	1:500
CD31	None	M	Bio-Rad	MCA1370Z	1:50
IgG (H+L)	AF488	Rb	ThermoFisher	A-11042	1:500
IgG (H+L)	AF647	Hm	ThermoFisher	A-21451	1:500
CTSD treatment experiments					
Target	Conjugated	Species Reactivity	Company	Catalog #	Dilution
6x-His tag	PE		Abcam	EPR20547	1:100
IgG (H+L)	AF594	Rb	ThermoFisher	A-21207	1:500
**WESTERN BLOT**					
Primary antibodies					
Target	Conjugated	Species Reactivity	Company	Catalog #	Dilution
6x-His tag	HRP		Sigma	A7058	1:2000
CTSD	None	H, M, Rt	Cell signaling	69854	1:1000
GAPDH	None	H, M, R, Mk, B, Pg	Cell signaling	2118	1:2000
NOTCH2 FL	None	H, M, Rt	Cell signaling	5732s	1:1000
RAB5	None	H, M, Rt, Mk	Cell signaling	3547	1:1000
p-p38	None	H M R Mk Dm Pg Sc	Cell signaling	9215s	1:1000
total p38	None	H M R Hm Mk B Pg	Cell signaling	8690s	1:1000
p-mTOR	None	H	Cell signaling	2976s	1:1000
total mTOR	None	H, M, Rt, Mk	Cell signaling	2983s	1:1000
p-AKT	None	H, M, Rt, Hm, Mk, Dm, Z, B	Cell signaling	4060s	1:1000

total AKT	None	H, M, Rt, Hm, Mk, C, Dm, B, Dg, Pg, GP	Cell signaling	9272s	1:1000
p-ERK1/2	None	H, M, Rt, Hm, Mk, Mi, Dm, Z, B, Dg, Pg, Sc	Cell signaling	4370s	1:1000
total ERK1/2	None	H, M, Rt, Hm, Mk, Mi, Dm, Z, B, Dg, Pg, Ce	Cell signaling	4695s	1:1000
p27 KIP 1	None	H, M, Rt	Abcam	ab32034	1:1000
Sema6D	None	H	Abcam	ab198745	1:1000
EGFR	None	H, M, Rt	Abcam	ab52894	1:1000
EGF	None	H	Abcam	ab9695	1:1000
LOXL2	None	H, M	Abcam	ab96233	1:1000
PEAR1	None	H, M	R&D Systems	AF7607	1:1000
SVEP1	None	H	R&D Systems	MAB97741	1:1000
Secondary antibodies					
Target protein	Conjugated	Species Reactivity	Company	Catalog #	Dilution
IgG (H+L)	HRP	Rb	Cell signaling	7074s	1:2000
IgG (H+L)	HRP	Sh	R&D Systems	HAF016	1:2000

**H-Human, M-Mouse, Rt-Rat, Rb-Rabbit, C-Chicken, Hm-Hamster, Cy-Cynomolgus, Rh-Rhesus, Mk-Monkey, Mi-Mink, Dm-D. melanogaster, Z-Zebrafish, B-Bovine, Dg-Dog, Pg-Pig, Sc-S. cerevisiae, Ce-C. elegans*

Supplementary Table 4. Sequences of primers used in the study.

Gene Name	Forward primer (5' -> 3')	Reverse primer (5' -> 3')
**Human primers**		
A2M	TGTGAGAAATTCAGTGGACAGC	GCTGGAAGACCTTGGTTTTTAC
ABCA3	GTCCTGGTGACGGTCCTG	CCAGATGAGGATCCCAGAAA
ABHD15	CTGCTCCACCAGAAGATCG	TGCTGGTGTCCACAGTGTC
ABI3BP	CCAGAAATGCAGCCTACGAC	GGGCGTCGTTTAGGTGTAGA
ACVR1	TCGCCCTCATGAATAGCTG	TTTGGCAGTGTGACGCTTAC
ACVRL1	CACCGAGTTCGTCAACCAC	CACCAGGGACACGTTGTG
ADAM10	AAGGAGGAAGGAAACGAACG	ACCTCCCTCTCGCTCCAC
ADAM15	TCAGGAGGTGTGAACATGGA	AACTCATGGGCTATGGAGGA
ADAM23	GCAATGCAATGCAGAAAGAA	TAAGGGCTTTCCGAGTCTTG
ADAM9	CAGGGTTGGAAAATGATGGA	GCAGGTTCCTCTCAGCACTC
ADAMTS7	AGCCGCCAGTATATCACCAG	GAGGGGAAGTCGATAATGTCC
ADAMTS9	AACCCTGCGAGTATGTCTGG	TTCCACAGGTCACTGAGCAC
ADCY4	CAGACCCGAGCTTCCTGAC	CCATACCAAGCCGGACAG
ADM	GTAGCGCTTGACTCGGATG	GCCTGCCCAGACCCTTAT
ADPGK	ACATGATGGAGGGACAAAGC	GGGATGTCAGAAATGGAGGTT
ALDOA	TGCCAGTATGTGACCGAGAA	GCCTTCCAGGTAGATGTGGT
ANGPT2	TGCAAATGTTCACAAATGCTAA	AAGTTGGAAGGACCACATGC
ANGPTL2	GCCCACTATGCCCACTCTC	CCTGCAGGCAGTCTCTCC
ANGPTL4	GACAAGAACTGCGCCAAGA	GCCGTTGAGGTTGGAATG
ANO10	AGCCAGCATGCACTAGTTCTC	GGTGAGTCTCGCCAGTCC
ANPEP	TGCACAATCATCGCACTGT	GAGCTGTTGGCGTTCTTGT
APLN	ATAAGGGACCCATGCCTTTC	CCTCCAGAGAAGCAGACCAA
APLNR	CATCTTCGTCAACATGTACGC	GGTAGCGGTCGAAGCTGA
APLP2	CCACGGTCATCGTCATCA	TGGCTGATGGTGCCATACT
APOA1BP	TGGACATCCCTTTCCTTGG	TGAAGCTGAAGCCAAAGATG
APP	GCTGATAAGAAGGCAGTTATCCA	ACCAGCTGCTGTCTCTCGTT
ARHGEF10	CAGTGCCACTCGCTATTTCA	ATTTCCACGGAGTCCCACT
ARL10	GATGTGCTGGTGTTTGTGGT	AGCTTGTGCAGCTCCTGTC
ARL6IP1	CTAGTAAAAACTCGACGCAGAGC	GTCCCACCCAAGCAAC
ARMCX2	ATGGCCAAGCCCAAAAAC	TTGTGAATCCGGCTCTTAGC
ARMCX3	GCGCCTGCTATTGCATTTA	GAACAGTCCCCAGCATCATC
ARSK	TGTGGATATTTACCCTACCATGC	GCAACAAAGAGTATCCACTCAGG
ASPH	TGTGCCAACGAGACCAAG	ACCTCGTGCTCAAAGGAGTC
ATL3	TTGAGCTAGATGAGAAAGCCTTG	TGAAACCACCACCACATCAA
ATP11A	GAGCTGACCACCAAGAGGAT	TGCTCAGCTCGAACAGGAC
ATP1A1	GCCTCGGAGAACGAGTCC	CAAACTGGAACCCTTCAGGA
ATP1B3	TTGATCTTGCTCTTCTACCTAGTTTTT	TGAAGCATAACCCACATCGT
ATP2A2	AACGTCGGGGAAGTTGTCT	GAATCAAAGCCTCGGGAAAT
ATP2B4	CCTTGTCTTTGCGGGTGA	GGCTGGGTGGTGAATGTAGA
ATP6V0E1	CCTCACTGTGCCTCTCATTG	ATGAACCAAGGCACCAAGAA
ATP8B1	CGGCAGAAAAACTTTGTGG	CTTCTGCTTGGGGGTGAC
AXL	GGATTCCTGTAGCTGCCTCA	GCAGAGGACATAGCGTCCAG
B2M	TTCTGGCCTGGAGGCTATC	TCAGGAAATTTGACTTTCCATTC
BACE2	GCACATACCGCTCCAAGG	CTTTG GGGATGGTGAC GA
BCAP29	TTCCTCAGTTCATACCATTGAGAA	CTGTGTGTGTTCATAGGCATCA
BCHE	CTGAATGTCAGTGCAGTCCAA	TGTGACTTTGCTATGCATATTGATT
BCL2L1	CATCCCAG C TCCACATCAC	TCCCGGAAGAGTTCATTCAC
BDP1	TTCCTTCAGAATCTCATCCCTTA	TCTGTCTGAGCATGGCTGTT
BGN	CTCCCAGACCTCAAGCTCCT	TGGGACAGAAGTCGTTGACA
BMP1	ATGCATTACGCTCGGAACA	CCCGTTCACCTCATACTTGG

BMP2	CAGACCACCGGTTGGAGA	CCACTCGTTTCTGGTAGTTCTTC
BMP4	TCCACAGCACTGGTCTTGAG	GGGATGTTCTCCAGATGTTCTT
BMP6	ACATGGTCATGAGCTTTGTGA	ACTCTTTGTGGTGTCGTGA
BMPR2	GGAGATCCCCAAGAGTGTCA	ACAGCAGAAACGGTATGTTCC
BSG	GTCCGCCAGAGGAACTCTT	ATCTTGCAAGCACTGGGAGT
BST2	CCACCTGCAACCACACTG	CCTGAAGCTTATGGTTTAATGTAGTG
BTD	CATATTCAGGGCGGAAGG	TAACAGCCGCAGAGGAAAAG
C10ORF58	CACAGACGTGTTTCTGTCCAA	CCTTCTCCAGTGTTTTCAGGTC
C11ORF41	CATACAGCCATGCAAGGAAA	GGTTTCCTGGGGAGGTAAGT
C12ORF49	CAAGGGAAGCACCTCATCA	CGTGCTAGGGACGTTGACA
C14ORF93	GCAAGCGGGATCTTGTACTC	TGCTTTCAGACCCATTGAATC
C18ORF54	AGCCAGTGTCAATGTGAGAATC	TCTTGGCCTTCAAGATAAGCA
C19ORF10	GGAAGTCCTATCTGTACTTCACACAGT	TTTCAAATGCGGCTTTAGAGT
C1ORF54	TTGCTGTGCCACTTATCCTG	TCATCATAACTGGGGGTGACT
C1RL	GGAGAAGCCCTCACTCCAA	TGGAGGACTCCCCAGAGA
C2ORF69	GACCAGCGAAGGAGGAG	CAGGATGACGAGTCATAATTTCA
C3ORF58	TGCAGTTGGTCCTAGAGATGG	CCATACATCCCAATTTTCAGGT
C4ORF48	CAGGACCTGCGGAAGACA	GCCTGCAGCAGTAGGGTCT
C6ORF120	TCGGTCCGATGTATAAAG	CTGCGAGGCTAGGAGCAG
CALCRL	GGGTCAGAGTGATGTAACAGGAA	TTGTAAGTGTTGTCAGATTGTAAGTGA
CALD1	CCAGAGGAATGACGATGATG	GGTCACCTGTCCCAAGGA
CALR	CTATGATAACTTTGGCGTGCTG	ACTCCTCAGCGTATGCCTCA
CALU	AAGGATGGAGACCTCATTGC	CCATCAGCATTCTTATCTATATCTTCC
CANX	ATGGGGCCTGAAGAAAGC	ATCATCTGCCCCACAACG
CAV1	CGGAGCGGTTAGTTCGATT	AACAAGCGATGAGAAGTCAGC
CAV2	CGGTGACTACGCACTCCTTT	CGTATTTGCTGATTTCAAAGAGG
CCBE1	ACCGATATGACCGGGAGAG	CTGCTGGCACACTCATCAAT
CCDC80	TCCCTGGAGAACTTCCTATCC	AGCAGAGATCACCAGCAACC
CCL2	AGTCTCTGCCGCCCTTCT	GTGACTGGGGCATTGATTG
CD164	TCACAACCTGTGCGAAAGTC	TGTAGTTCCTTGTGTGGCATCT
CD46	CGGTAAGCCCCCAATATGT	ACTTCACTAAAGGTGTGTTTTCCA
CD63	GGAGAACTATTGTCTTATGATCACGTT	TCTGACATCACCTTATCTCTAAACACA
CD81	CAGCAACATCATCAGCAACC	CTTCCCGGAGAAGAGGTCAT
CD9	ATGATGCTGGTGGGCTTC	AGTCCCAGCATGCACTGG
CD93	GCGACAGCTTGTGCTTCA	GGTGCAAGAGACCCCATTT
CD99	TGGCAGCAGGGTTAGAACA	CCTCCATCTCTGCTCTCAGG
CDH5	GATGCTCCTCGCCACATC	GTTGGGCAGGGTTAGCAC
CECR1	GGGGCTCCGAATCAAGTT	CTTGTAGTCATGCAAGGAGTGG
CFH	GAATGGGTTGCTCTTAATCCA	CCTCCTGTAAGGGTAAAAGTACC
CFI	TTGGATTCTGACTGCTGCAC	TTGTCCATATTTGGTAACGATGA
CHID1	GTGGTCCAGGTGGCAAAG	CACGCGCTTCTGGCTTAG
CHST15	CTCATCACCTTTCCATTACGG	GTTTTCGCTGTCCATCAAGC
CHSY1	TGTACACCACCCATGAGGAC	CCCTTTTTGTTCTGCTCGTAA
CKAP4	ACAACATCGCCATCTTCACA	GCAACCTTTGCCTTCATGTC
CLDN11	GGCTTCGTCACGAGCTTC	GGTCACCACCCAGTCATTG
CLDN5	AGGCGTGCTCTACCTGTTTT	GACAATGTTGGCGAACCAG
CLEC11A	GGGGAGCAGAGAGGGAGT	GGCTTCCTGCAGATGCTT
CLEC14A	CTGGGACCGAGGTGAGTG	CCGCGATGCAAGTAACTGA
CLEC1A	ATGCAGGCCAAGTACAGCA	ATGCAGGCTCATGGTGGT
CLSTN1	CAGCACCATCACGAGAGAAG	CTCCTCGGACACCAGTGATT
CLU	GAGCAGAGCGCTATAAATACGG	CCAATTCTGGAGTCTTTGCAC
CMKLR1	GCAGTGGCAGATTTCCTGTT	CATGGCGGCATAGGTGATA
CMTM3	ATCTGCTATGTGGCGTCCTC	AGTACAAGGCCAGCAGGAAC
CNOT1	GCAATTTCCACATCATTTACAGG	TCCAGGGGTTGTGATAGAGC

CNPY4	TGAAGGAGGAGGACGATGAC	GCTCTGTGCTCAGCAGCTTAC
COL12A1	ACACCGAGCACCAGCTTC	GAGCTTACAGCGGCATGAA
COL18A1	GAAGTCGAGGAGCAGACCA	CCCACGTGGAGACAGAATC
COL1A2	GAGTCCGAGGACCTAATGGA	AGGGGAACCAGGAAGACCT
COL27A1	GGCCTTATGGAAATCCAGGT	GGGTCCCCTTTCTGTCCTT
COL4A1	GACCCCGGAGAATAG	CACTCCTGCAACACCATCTC
COL4A2	CCAGGACAGAAAGGAGACCA	GGTGTGATGCCTGGGAAC
COL4A5	AGAGCCCACGGTCAAGACT	CATGAAAGGCATGGTACTAAAGC
COL4A6	CTGGACTCCCAGGAGCATC	CAATGTCTGGCCGTTGTC
COL5A1	CGGTGGTCCGAGACAAAG	CCTGGATGAGGAGGTGTTTG
COL5A2	CAAAAGAGGTCCCAGAGGTG	GAGCCTGGAAAACCACGAT
COL6A1	GAAGAGAAGGCCCCGTTG	CGGTAGCCTTTAGGTCCGATA
COL8A1	CCAACTCACCCTTGAAGTCAT	GGCTGGTTTCTGTCTCTTCAG
COLEC12	TGGCTATGTCACGAATCTGC	CATTATGAGAATACATTTGGTTCTGC
COPB2	CGAGTTAAGAGTGTGGATCTGC	CACACACACTGCCATTGTAAAG
COPS6	CGGAGTGACTGGGAGTGTTT	GGATCCAGTGGTCTGAGATGTT
COX4I1	CCAGAAGGCATTGAAGGAGA	CTCCTTGAACTTAATGCGATACAA
CPD	GGTTTGGAAAGCCTCATGTT	GGTATCGATAAAGAGCCAGAGC
CPQ	TCATGGGAAGCACTCTCACTT	CCACCTTGTTCTTCTGCAGTC
CPT1A	CCCCTCCAGTTGGCTTATC	CTAACGAGGGGTCGATCTTG
CPT1C	CAGTTGGCTTTTCCTCTTCAGT	TCAGTCCTAAGGAAGGATCCAG
CREG1	AGCTCTCCGTGAGCAACC	TGTGCCAAAGTCATGGTCAG
CRELD2	TGTGTGTCCTGACGGCTTC	GTGTCGGGCTTTCTCCTTCT
CRIM1	CTCCTCCTGGGGAGTGCT	GCAGACTTTGCGCAGACA
CRTAP	GCCTCTCGGATGAGCACT	GCTCCTTCTGGAGTGTGGTC
CSF2RB	CACCGACCAGGACCACTT	CAGTGGCTCTGGGGACTC
CST3	CGAACCACGTGTACCAAGAC	GCACAGCGTAGATCTGGAAA
CTGF	CTCCTGCAGGCTAGAGAAGC	GATGCACTTTTTGCCCTTCTT
CTHRC1	CCAAGGGAAGCAAAAGG	CCCTTGTAAGCACATTCCATTA
CTSB	TGGAGGGAGCTTTCTCTGTG	TGACGTGTTGGTACACTCCTG
CTSD	CATCTTCTCCTTCTACCTGAGCA	GTCTGTGCCACCCAGCAT
CXCL1	TCCTGCATCCCCCATAGTTA	CTTCAGGAACAGCCACCAGT
CXCL11	AGTGTGAAGGGCATGGCTA	TCTTTTGAACATGGGGAAGC
CXCL2	CCCATGGTTAAGAAAATCATCG	CTTCAGGAACAGCCACCAAT
CXORF36	TGCATCGGGACATCTATTTG	AGGAATCGGGAGGCAGTC
CYB5D2	TGAGAAGAATTATGTGTGTGTTG	CAGCTTCTACCTGGGTCAGTG
CYR61	AAGAAACCCGGATTTGTGAG	GCTGCATTTCTTGCCCTTT
CYTL1	ATCACCCGCGACTTCAAC	CAGCTTGTCCAGCACACAGT
CYYR1	CCACGAGATGGAATACTGTGC	GTGGAGAACGCTGTGCTG
DCBLD1	GCGACAGTAGCAACAACCAC	CTTTTTCTCCCCCAAATCG
DCHS1	CCCCAGCTTTCGCTGTAG	GCAGGGGAGTTGTCATTCAC
DDOST	ATCCCATCCTCTTTCGAGGT	CCAGCACCAAAGGGTTATCA
DHRS4L2	TAGGGTGAACTGCCTGCAC	TGGCTCGCCTAACCTTCTTA
DHRSX	CGGCTGGTTGCTTTTCAA	TCTCTTTCTCGTTGTATAGGTAA
DKK1	CAGGCGTGCAAATCTGTCT	AATGATTTTGATCAGAAGACACA
DKK3	CACATCTGTGGGAGACGAAG	CCCACAGTCCTCGTCGAT
DNAJC10	AATTCTGGAGAACTGTGGTTTGT	GGAGCTAAATCATGGCAGTGT
DNAJC13	GAGTGGCATTCAGAAAAGTGAA	ACTCAGTTTGGCAAGGCTGT
DNASE1	CCCTCGCAGCTACATTGTG	TCTGGTGCATCCTGATTGAG
DPP7	CCCTACCCCACTGACTTCC	CCGAGGCGTTGTAGACCA
DPY19L1	CCTAGAAGTTGTAAAAGAATGACTGCT	GCAAAACATTAAAACCATTACAGATG
DRAXIN	AGCACCTGCCACAGAAGAGT	GTCAGACCTGGGCTGTGC
DST	CCAGCCCGGTTAACTATTGA	TGGCAGAGCTGTAAGATCCA
DYSF	GCCATTCCTGGATCAAAGC	CGGAGGAC GTGAAGGTAGTT

ECE1	GAGTGGCCAGAGGTGCTG	CTGCCGCCAGAAGTACCA
ECM1	CTTGACCTATTTGGCTGTTGC	TCTGTCCTGTAGCCGTGAAG
EDN1	TCTCTGCTGTTTGTGGCTTG	GAGCTCAGCGCCTAAGACTG
EFEMP1	GCATGTCAGTGCCCTCCT	AGGGATGGTACATTCATCTATGTCT
EFEMP2	TGCCACCAGGGCTATGAG	ATGCAGCGGTACTGACAGAG
EFNB2	TTATCATCCCGCTAAGGACTG	CCGCTGACCTTCTCGTAGTG
EGFL7	TGGACCTGCTGGAGGAGA	CGAGCTGCTGGAAGGAGT
EI24	AGTATAGAGCGGAAGCAAGAGAGT	CACTCCACCATTCCAAGCA
ELOVL1	CCCCCTTGCTAATGACCTC	GCCCAAGTGAGAGAACGAAG
ELTD1	TGCCGACCTGTAAAAGTGTG	GCAAGATTTGGCAGACTTCAT
EMCN	GCAGAATCAATCCCGACTTC	CATCACTTTACTGGGACAGACG
EMILIN2	TGGAACCAGATGCCCTGT	GCAATCTCCCCCTCTAAAGC
EMP1	GTGCAGGCCTTCATGATTCT	GGTGAAGAGCTGGAACACG
ENG	CAGCTGCCACCTGGACTT	ACAGCAGGCTCACACAGTTG
ENTPD1	CTATCAACAATGCCATCAAAGC	ATTGAAGGCACACTGGGAGT
ENTPD4	CGCCAAATTATGGTCATTAGTGT	CGCCCATACTTATTTCGGATT
ENTPD5	TTGGATTGAAAGCTGCAAGA	GAAAGTGTGCCCATCAGTCC
EPB41L3	TTGCAGTTGCGAGATGACA	CAGTGTAGGAGCCCAGCAA
EPHB2	ACTCGGCCACCAAGACCT	TGCAATGGTATCCACGTTCA
EPHB4	CGATTCCCGGAGACTGTG	ATCCACCACGCAGCTACC
ERMP1	CCCACAGGCTCTTTTAGCAT	GGGTTCCAGCTTTACCACAA
ESAM	TCACCAACCTTTCGTCTTCC	CGTCACATTACATTGGGCAGT
ESM1	GGTGCCGTAGGGACAGTCT	CATGGATGGCATGAAGTGTG
EXT1	GGCTTGGG TCCTTCAGATTC	CATCCATTGCTGAGCATCAC
F11R	CCTCCATCCAAGCCTACAGTT	GGGGAACCATCTTGTTCTGA
F2R	AGTCAGGAGAGAGGGTGAAGC	CCGCACAGACTGAAGCAG
F2RL1	CACATACATGGCAACAACTGG	GCCATAGAAAAAGCCAATAAGC
FADS2	TTAACTTCCAGATTGAGCACCA	GGGCGATCTTGTGTAAGTTGT
FAM131A	CTGGGGGAATGGACACAG	GTGAGCAGTCGCTCTCAGG
FAM171A1	TTTTC CTCTCTGAGCCTTGG	TTGGACGACATCTTCATATACCA
FAM172A	GAGTCCATGCTACCTGATTGC	TTCCAGGAGTTAGCTCGTGA
FAM174B	GAGGTTAAAGAAGACACGCAAGT	ATTTAGTGGCGCCATTTCC
FAM20C	CGCTGTTCAAACCCATGAA	CATTGTGCCTCTCGTAGTCAGA
FAR1	TGTAGTTGTCAACATGAGTCTTGC	CACCATGATGTTTCTTGGTCTATT
FAT4	GAAGGAGAAGTCATGGAAAACAA	GAGGGAGATCAGGGTCAGTG
FBN1	TGGGCAAATATCAGGATCTAGTG	CAGTGGGCCAGGAATGAC
FBN2	GCTGCGAGTGTGAGATGG	TTGGAAGGAGCATTCATCA
FGFR1	AGAGAGGCCGGCAGTGAT	GCCCCTGTGCAATAGATGAT
FJX1	AAGTCCCGGACCGAGTGT	TCCTCTCCTCTTCCACTTAAAGC
FLRT2	TCCTGAAGTCTTGGCTTATCATT	AGGCCAGGAGTTTGGACAC
FLT1	CGCATGGCAATAATAGAAGGA	GCTTGTAGGTGGCAACATGA
FN1	GGGAGAATAAGCTGTACCATCG	TCCATTACCAAGACACACACACT
FNDC3B	GATGATGACCGACCAAATCC	ATGGCTACCTCTCCGTTCAG
FSTL1	GCCATCAATATTACAACGTATCC	TCAATGAGAGCATCAACACAGA
FSTL5	TGCTAAGCTGGGGTACCTTG	GTGAGGCACATTCCCACTG
FUCA2	TCTTGGCCTCCTTATATAATGAAAG	AGAAGCCACCATGCTTACAGA
FZD4	TTCACACCGCTCATCCAGTA	TGCACATTGGCACATAAACA
GALNT1	AGAGCCAGTACAAAAGCCTCA	TAGGAATGACGACTGGTTTCC
GALNT7	ACACATATGAAATTATACCCCAAGG	TACTCCAATCCCATGCTCCT
GANAB	ATGGAACGCCCCTTTGTC	TGTTGTGGCCTGTGCACAC
GAPDH	GCATCCTGGGCTACACTGA	CCAGCGTCAAAGGTGGAG
GAS6	ACCTCATGGGCAACTTCTTC	GGCTGCATTCGTTGACATC
GBP2	GTGGGGATCTACCCTGCAT	GGCCCATCTGCTGTTCATAG
GDF11	ACCACCGAGACCGTCATTAG	AGGGCTGCCATCTGTCTG

GDF15	CCGGATACTCACGCCAGA	AGAGATACGCAGGTGCAGGT
GDF3	CGCTTTCTCCCAGACCAA	GGCAGACAGGTTAAAGTAGAGGAG
GGH	AGGCTGGATCTTACAGAGAAAGA	CTGAGCGTCTGAGGTCAACA
GIMAP1	CCAGTTGGGTCGGTTCAC	CGAACATGTCCCTCACCTG
GIMAP2	ACAGGAACTGGCAAAAGTGC	CGATTCAAATGCTTGCTTCC
GJA1	AGGTGTGGCTGTCAGTACTTTTC	CATCTCCCCAGGCTGACTC
GLB1L	TCATCTAAGAACCTCAGATCCAGA	GCAGCAAGACCTTGAACCA
GLG1	GTTTCTGAACTCTGCAAGAAAGC	GCCGGTCTAAGTGAAAGTCATC
GNAS	AAGGACAAGCAGGTCTACCG	GGTGCTTTTACCAGATTCTCCA
GOLGB1	TGACGTGGCTTGAATTGG	TGATAATCGGCTCAGCATTTC
GOLM1	GGAGCAGCTTGACAAAATCC	CCTGGTACAGCTTGTTGACG
GPC1	AGCACCTGCGGATCTGTC	GGCCAGGTTCTCCTCCAT
GPR116	GGTCCACTCAGAAAGCCATT	TGATGACCGAGATGGCAAG
GPR124	TCTTGAGCAATAACAAGATCACG	CTTCTCCAGCAGTGACAGTCC
GPR126	CGCTCAGATCGAATGTGGA	AGGAACACACATGATATATAAAGCAAA
GPR176	AGCCTGGAACCCAGCATAC	CTGCCCAATGTGGAACATCT
GPR56	GCCACAGGGAAGACTTTCG	GTAGTGGAGGCTGCTCCTGT
GPX3	CAGAGATCCTTCCTACCCTCAA	CCCTTTCTCAAAGAGCTGGA
GPX7	CCATCCTGCCTTCAAGTACC	TTCCATCTGGGGCTACTAGG
GPX8	CCTGAGGGTCAAGTTGTGAAG	CAGGCCTGATGACTTCAATG
GRN	CTGAGGTAGGCGGGTCATC	ACCAGGGTCCACATGGTCT
GSN	TCTGCCATCCTGACTGCTC	CTTGGACCACACGGCTCT
HEG1	CCGAATGTGACAAAGACACCT	AGTATCCCGACTTGCACTGG
HERC4	TTGCTTGGAATCCCTTTCAT	AGATAGCTCCAGAAAGGGTGAG
HHIP	GCTTCTACCCTCGGCTGTC	TATTCTCCAGGCGCCCTA
HMOX2	GAGGAAATGGAGCGCAAC	GCTCCATGGGGAAGTACAAA
HPSE	GAAAGACGGCTAAGATGCTGA	TGATGCCATGTAACTGAATCAA
HSD17B11	TGCTACACAAGATCCTCAGATTG	ATGGCCATGGTTATTCTTCG
HSP90AB1	CCACCCCATTGTGGAGAC	ACCACCAGGTCCTTAACTGC
HSPG2	TCTGGCTCAAGTGCTGTCC	GAGGAGGAGGGCTCGATG
HTRA1	CACTCATCAAAATTGACCACCA	ATGGCGACCACGAACTCT
HTRA3	GCAGCTACAGAATGGGGACT	CAACAACACAGGGAGCTTTTT
HUWE1	AGAGAGCGGCTGACAGAGG	CACTAACCCACTCAGGTCAGG
ICAM2	GTCCTCCAATGCCACTTCA	ACGCTGACGTTGGAATTCAT
IDE	TCTCGGAACCTTGCTTCAAC	TATGCCATTAGCTCGAGTG
IFI27	CCCTGCAGAGAAGAGAACCA	CCCCAGGTATATTTGGGATAGTT
IFITM2	GCCCTTGACCTGTATTCCAC	AAGGGCTGATGCAGGACTC
IFITM3	AGATGCTCAAGGAGGAGCAC	GATG TGGATCACGGTGGAC
IGF2R	TCTCCAGTGGACTGCCAAGT	GTGCTTAGGCCAGTCAGGTC
IGFBP1	AATGGATTTTATCACAGCAGACA	GGTAGACGCACCAGCAGAGT
IGFBP2	GGTGGCAAGCATCACCTT	TCCTGTTGGCAGGGAGTC
IGFBP3	AACGCTAGTGCCGTCAGC	CGGTCTTCCTCCGACTCAC
IGFBP4	CCTCTACATCATCCCCATCC	GGTCCACACACCAGCACTT
IGFBP6	TGACCATCGAGGCTTCTACC	CATCCGATCCACACACCA
IGFBP7	ACTGGCTGGGTGCTGGTA	TGGATGCATGGCACTCATA
IL13RA1	GCACAGTAATATGGACATGGAATC	GCCAAAATGACTAAAATACCATAGACT
IL17D	TGGACCAAAGGGATGCAC	AACCAAATCTGAGATCTTTGGAAC
IL18BP	GAGGTGGAAGTGCCACTGAG	TTAACCCTGCTGCTGTGGA
IL1RL1	TGGGAAGAGTAGCCTATATCTCAAC	ACTGCTGACTTGAATGTTACGG
IL32	TTTCAAGTAGAGGAGTGAGCTCT	GAAGGCCCGAATGGTGAT
IL4R	CGTGGTCAGTGCGGATAAC	TGAAGGAGCCCTTCCACA
IL6	GATGAGTACAAAAGTCCTGATCC	CTGCAGCCACTGGTTCTGT
IL6ST	CCATTTTACTATTCCTAAGGAGCAA	GAATGTTGCAAGTGAGCTGAATA
IL8	AGACAGCAGAGCACACAAGC	ATGGTTCCTTCCGGTGGT

INHBA	TTTTCCTTCTCCTCTTCAGCA	AGCTCAGACAGCTCTTACCACA
ITGA10	AGTCCTTGCTGACCCTCCA	CAAAGCCAAACCGAGCAT
ITGA2	TTGTTGTTTGGCCTACAATGTT	TCACTTGAAGGACCGGAAA
ITGA5	AGCTGTGCAATGGACCAAG	GCCAAACAGGATGGCTAGG
ITGA6	GATGAGATCACTTTTGTATCTGGTG	CAAAACCACGGCTCCACT
ITGAV	GCTTGAAGATTGTCTGCCAAGT	CATAAAAGTCTCAGTCCACAGTAATGA
ITGB1	AATAATTTCAAGAATCCAAACTACGG	AATAGGATTTTCACCGGCAAT
ITGBL1	CGATGGTGTGGTCTGTGG	TCTTCCGTCATGTTACACTTCC
ITM2B	GCATTTATGCTTGCAGGTGTTA	CCACAGTAGTACACGTCATCTGG
ITPR2	GAATGCCATGCGTGTGTC	TCAGTTTCCAGAACGGATGA
ITPR3	TACTTGGCCATCGACGAGAT	TGCCATGCACAGGAAAATC
JAM3	TGGTATCGCAATGATGTACCAC	AAAGAAGAATTGC GAAATCTGG
KDR	TCCCTGTGGATCTGAAACG	CTCCTCCACAAATCCAGAGC
KIAA0100	CTTTTGGATAGTGATGGGAAAAG	AAAGCTCCACTAGGCAGGTG
KIAA1324L	CAATGGCCTTCTCATTCCTG	GACAAGTGAGAATAGAAACATCAAGC
KIAA1797	TGGACTTGTTCTGTCCCTCA	AGCAATGCTGCTACGTGAAC
KLF2	CGTGCTGGACTTCATCCTG	GGGTTCGGGGTAATAGAACG
KLF4	CGTTCCAGTGCCAAAAATG	CATGTGTAAGGCGAGGTGGT
KTN1	CAGCTGCCTCGAAGAAGAA	TCCACCTTTTTATCGTGGTCA
LAMA3	CTTGAATACCCAGGGCATCTAT	TCCAGCTGTGTTGTGCTGA
LAMA4	GGATGCCGAAGACATGAAC	TTCCCTCACTCTTTCCTGTTGT
LAMA5	CCTCGTCCTCCAATGACAC	GCGCTGCAGTCACAATTC
LAMB1	AGTGCATGCCTGGGTTTG	CCTGGGGTCACAGTCACAG
LAMB2	GGTGGCAGTCAGAGAATGGTA	CAGCAGGGCGAAATGTCT
LAMB3	CCATTGCAGTCGCACCTT	GTGCACCCTTCTACAACAACC
LAMC1	GTGCTGTTGTTCCCAAGACA	GCCATCATCACAGAGCTCAC
LAMC2	CTACTTCGGGGACCATTG	GGTTACAGTTGCAAGCTCGAC
LAMP1	CGAAGGCGTTTTCAGTCAAT	CGTCCAGCAGACACTCCTC
LAPTM4A	TCACCTATTTGCCAAGAATCAA	AACAGGAGGCAGCTGGAGT
LAPTM5	GTCCCTGCAAATCATGGACT	CGGGCAGCTCAATGTAGG
LCAT	TGAGCTCAGTAACCACACACG	CAGCTTGGCTTCTAGCTGATTC
LIPG	GCTCTGGTTTCGCAAGTGTC	CCTCAGGGAAGCTCCACA
LOXL2	GGAGAGGACATACAATACCAAAGTGT	CCATGGAGAATGGCCAGTAG
LPCAT2	TACGCCCGGCTCAACTAT	GAAGAAGGACGCCTGACG
LPHN2	AGCTGGATCACAGGAAGGAA	TTGTTATAGGTGGAATTTTGGTTG
LRIG1	CAGCTCCTGGTGGTTCAGA	GGTGTTGGACATCTCACAGGT
LRIG3	CAAGTCCTCTTTAGATTTTAGTGCAA	GAAAGAATTACTCGAGGCAACC
LRP10	CCTCCTCCTCCTCCTTGG	AAGGTGCCCTGCACTTCTAA
LRRC17	TTTGCCACAATTATGTGTTTCC	GAGGGATGTTGTTTGGCACT
LRRC32	TGAGACCCCAGATCCTGCT	GGGCACTTTGTCTTGGTGTT
LRRC8C	ACCGCTCTTCATCCTGCTC	AGGATGTCACAGGTCTTCACC
LTBP1	TGTCTACGAGGGCTACAGGTT	GCAGAGGTGTTGGACCTGA
LTBP2	AGGGCTCCTTCAACTGTCTATG	CCATAGTCCTCACACTCGTCAA
LTBP3	CCAATGGCTCCTACAGATGTC	GGCTGCACTCATCTAGTCTTG
LTBP4	GGCTTTCTGCTCGACTCGT	AGAATGGGCAGCGAACAG
LY96	TGCCGAGGATCTGATGAC	ATTAGGTTGGTGTAGGATGAC
MANF	GGCAAAGAGAATCGGTTGTG	GGCCAGAGGCTTTGATACCT
MANSC1	ACCAATGCTTCAGTGACACCT	GGGAGGCTGAGAAGTGACAG
MARVELD 1	CTGCCTTCTGGATCACTATCG	AAGAGCACGGACACGAAGAG
MATN2	GAGGCCGACGAGGAAGAC	TTTTCAAGGCGAGCAAGAG
MATN3	TTCCAGGAAACCTTCTGTGC	CCTTGGCTACACTCACAGTGG
MBOAT7	CTGCTGCTGACGCTGAAG	CTGAAGCCTGAGGCCATTT
MBTPS1	GCTTGTGACCATGAAGCTTGT	TCTTCCCACAGAGCAAAACC
MCAM	AGACGAGCGCATCTTCTTG	AGCTGGATGCGGTACTCCT

MCL1	AAGCCAATGGGCAGGTCT	TGTCCAGTTTCCGAAGCAT
MCTP1	GGCAGAGCTAGAAAATCCAAAAG	AAGCCAAAATAATGTGAGAGAAGAA
MDK	AGTCGCCTCTTAGCGGATG	CCGCCCTTCTTCACCTTATC
MEGF6	GACCCGCTGTCAGTATAGAT	GTAGGAGCCTGGGGTGTTC
MERTK	TCCTCTGCAGACACTATTGAAGTT	TGAAGAAAATTCCTGTTGCTCA
METRNL	GCTCCTAGCCGTCTGCAC	CTCAGGCTCGTGGGTAACTT
MFAP2	AGCAGTGAACGGAGTCACAA	GAGGTAGGCAGCTCTCATGG
MFGE8	TTTGAGCTACTGGGCTGTGA	GGCCGTGATCTGCTTGTC
MMP1	GCTAACCTTTGATGCTATAACTACGA	TTTGTGCGCATGTAGAATCTG
MMP11	GGTGCCCTCTGAGATCGAC	TCACAGGGTCAAACTTCCAGT
MMP14	CTGTCAGGAATGAGGATCTGAA	AGGGGTCACTGGAATGCTC
MMP16	GGGAGATACTGTCGAAGAGCTG	TCTTCCAGTGCGGAGGTATC
MMRN1	GGGCCGAAATGTCAACTAAG	GCTACTCCTCTGCCAACAGC
MMRN2	GCAGAGCTCCAGGACACCT	GGTAGGGGCACCAGTTACG
MPZL2	ATGGGAATCCTGAGCGGTA	ATGTCCCATTGTCGTCGAAC
MST1	TGTGGTGGGTCACAGTGC	GGAGCACTTGGAAGTCATTCA
MTCH1	TACGTGAAGCTGCTCATCCA	TGAAGAAGCTCGGCAGATAGA
MTDH	CACTGGAGATGCTAATACAAATGG	GAAACTGGCTCAGCAGTAGACC
MTUS1	TATGAAGCATTCGTCCAGCA	TTTAAGCCGATTCTCTCGTTCT
MYCT1	GGCCTCAACAGAACTGGATT	GGCTGAGGTTGCTTCGAC
MYOF	GCCAACGTCACAGTTCTCG	CAGCCGCCTCATGTATTTG
NBL1	GTCCATGTGGGAGATTGTGA	GGCAGCTACAGTGCAGGAT
NDST1	CACAAAGGCATCGACTGGTA	AGAAGTCGGAGGTGGTGTTG
NENF	TGCAGACCTCACCCATGAC	TGGCTTGTACACTTTGGTGA
NF2	GGAGACAGCTGACCTGTTGG	CCTTGATGCGCTGCATTT
NGRN	GCCATGGAGCAGATACGG	TCACATCAGTGCTGACATCAAA
NHLRC3	TTTGCATTCGCGTTTTTGT	CACATCCAGCCGGTAAAGA
NID1	CAGTTTTCAGATGAGGGAACG	TGAAGGCCAGTTTCACAGTAGTT
NID2	GGCTGGCTCTTTGCTTTAGA	ATGGGTAAAGGCAGCACCT
NOTCH1	CCTGCAGTGCAACAACCA	TTCCAGGGGTCATTGAAGTT
NOTCH4	GGGTCCTGACTGCCTGAC	GCAGCTGCCATTGTATAGGC
NPC2	CAGTGAAAAGCGAATATCCCTCT	GAGATGAGAAACGATCTGTACTG
NPR3	GTTTGTTGAAGGATTCCACGAT	AGTACTTCATGTAGAGCCAAGACG
NRCAM	AAGAAAAGGAAATTCAGTGTGTGA	CGCTTCTTTTTCGGCATTAT
NR2F1	GCCTCAAAGCCATCGTGCTG	CCTCACGTACTCCTCCAGTG
NRP1	TACCCTGAGAATGGGTGGAC	CGTGACAAAGCGCAGAAG
NRP2	GACAGTGAATCCGCAGACC	GCGTAGTCGGAGGTGAACTT
NT5E	AACCACGTATCCATGTGCATT	ATTGTTGCGTTCATCAATGG
NTN4	TGCACAATAAGAGCGAACCA	TCCTTACATTCGCATTTACCTG
NUCB1	GCTGCTCAAGGCCAAGAT	TTCTGAGGGTCCAGGTGTTC
NXPE3	GTGGACCTGATTGGGTAACTG	GGGACTACCCAAGTTAAACTCCA
ORAI2	TGAGCTTAACGTGCCTATCG	CGGTAATCCATGCCCTTATG
OSTC	TCGAATGTCCCAACCTGAA	TCCGGTGATGAGGAAGTAAGA
OXA1L	TGCCTGATTTTTC CTCTCATC	CTCTGGCAAGTGATTGTGGA
P2RX4	CCTCAAGTCGTGCATTTATGAT	CTCCACTATTTTGCCAAGACG
PAM	TTGCTGGCATGTACCTTATGA	CATGAAATGTCAGAATTCACCAC
PAPLN	AGTTCGACGGAGCGGAGT	TGCAGTTCAGTTCACACTTGTTT
PARP12	GTTTCCAGGCCCGAAGAG	CTGAAAGCCTGGGTCTGG
PCDH12	CAGACACAGATGGCCCAAG	GAGGTCCTCTTCAGGATCCAA
PCYOX1L	GCACATGCAGGACTTCGTC	TCAGTCTCCTCCAGCATGAA
PDGFA	GATGAGGACCTTGGCTTGC	CCAGCCTCTCGATCACCTC
PDGFB	CTGGCATGCAAGTGTGAGAC	CGAATGGTCACCCGAGTTT
PDGFC	CAGCAACAAGGAACAGAACG	TGGGCTGTGAATACTTCCATT
PDGFD	CTGGGAGTGCAACTGTAACG	ACCATGACCGGAAGTCAAAA

PDGFRL	GTCAAGTACCAGCTGCTCTACG	GCCAAGATGGTTGTTGAGG
PEAR1	CCTCTGTGTCCCCTCCTTCT	TCACTGGGGTTGAGAGTTGC
PGF	CTCGGGACGTCTGAGAAAGAT	CAGCAGACAAGGCCCACT
PLA2G12A	GCGTTCATAAGATAGACACGTACC	CGTGGGAAAGGCTTAGATCC
PLAT	AGCTGTGGGGAGCTCAGA	CACAGCGTCCCTTAAATTCAC
PLAU	TTGCTCACCACAACGACATT	GGCAGGCAGATGGTCTGTAT
PLSCR1	CCTCCAGGATATAGTGGCTACC	CTGATTTGGGACAGGAAAGC
PLSCR4	CCACAGCAACCCAGTACCTT	TACCGGACAGGATGGATACC
PLTP	CCACCTACTTTGGGAGCATT	CAGCTTCAATGGGGAGTCA
PLXNA2	GAGATCTACCTGACCCGGCTA	AAGTCGTCCACAAACTTCTGC
PLXND1	CTGCTGTTGCTGCTGCTC	CAGGGCGAAGTTGTTGGT
PMP22	CCACCATGATCCTGTCGAT	CCCTTGGTGAGGGTGAAG
PODXL	TGCAGACACCACTACAGTTGC	GGCTGCTTGTGGTGTTAGGT
PON2	AAAATTCCCAGGACTCCACA	CTTAATTCCCGTGCCCTTG
POSTN	TGTTGCCCTGGTTATATGAGAA	ACATGGTCAATGGGCAAAAC
PPT1	GGAGACCTCCCTGTACACACA	TGAAGATGGTCCCCTTCTGT
PRADC1	GGGCTGGTGTTGTCTCGT	CAAATAATCATGGATACGGAAGC
PRNP	CTGTCCTCCGAGCCAGTC	CCAAGGTTCGCCATAATGA
PROCR	CTAGAGCCCATGTCTTCTTCG	GCCGGAAACTCACAAAGG
PRSS3	TCATTCCCCTCCAGGACTTT	GCAGCTCACTGCTACAAGACC
PSAP	CCTCTCCTCCTCTACCCTCAG	GCAAACGTCCCCATTATCC
PTDSS1	GGTTTGACCCCAAATCTTCTT	TGCCAGATGATCATGAAAAGG
PTPRB	CGGAGCCAGAGAGATGTAACTT	AATTCTCCACTGGATAGACACAGA
PTPRE	AGCAGCAAAGGGTGATGC	CACGGGGATGGGAAAATACT
PTPRF	CCTGCGAAGCTGTATGTGC	CATCACCTCCTGGCTGCT
PTPRM	GCAGCCTTTTACAATTGGTGAT	AGCTTGGAAATAAATTCTGTAGCTTT
PTTG1IP	AAAACCCGTATGCTAGATTTGAA	ACAGGAAGCGTCGGGACT
PTX3	GCGGTGCTAGAGGAGCTG	GGAATAAAATAGCTGTTTCACAA
PVR	ACTGTCACCAGCCTCTGGAT	GAGGCTTCTCAAAGCTCTCG
PVRL2	CGGATCTCCTGGCTCTCAT	AGGGTCCCTGACACCTGAG
PXDN	GGGGAACACCGTGTACTTCA	CTGTCTTCATGCTCAGCTCATT
QPCT	CAGTCCGAGTGCCTCAGC	ACTGGTGCCTTCTGCAATTT
QSOX1	CTGGCTTCCCGACTGTGA	AGCACCAGCCACTGGAAA
RHBDF1	CTGCCAGGAACCATGAGTG	CTTTAGCCAGGGTGGCTTC
RNF144B	AGACAGTGTGCCCTGTTGC	CAGAATTTCAGGTGGCAAGAA
RNPEP	GAAGGAGTTCCTGCATAACCA	CTCACTGCCACCCATCATT
ROBO4	AGAGCGCAGAGCTTGGAG	GCCAGAGGATGGTCTCACTT
RPN1	CCAGAGACAGCCAGATAGTGG	AGCAGCAGGAAGGATGGTC
RPN2	CCTGCGAAGCTGTATGTGC	ATCCAGGCGAGCAACAAG
RSPRY1	TCGAGACAGCAAATTCCTCA	TGGCATTGTACCAAATCAGC
RTN4	GAGCCTGTGATACGCTCCTC	TCTTGACCAGCCGAAATAGTG
S100A10	GGAGTTCCCCTGGATTTTTG	CACTGGTCCAGGTCCTTCAT
S1PR1	CCGCCTCTTCCTGCTAATC	GCAGTTCCAGCCCATGATAG
SCARA3	TGCGGATTCTTTACCTCTTCC	TTCGGAGAGAGAGTCCACTTTT
SCARB2	CAGTTCTATTTCTTCAATGTCACCA	AATATTTGCTTTGTTTCTGAGTTCC
SCARF1	ATGAGCCGTGCTCTCCAG	ACTGCTGTTCGCAGCTCTC
SCCPDH	GCTGCAGACAAAGGGGTTTA	GATCTGCTGGAATGGAGTCAA
SCD	CCTAGAAGCTGAGAAACTGGTGA	ACATCATCAGCAAGCCAGGT
SCD5	CGACGCCAAGGAAGAAATC	ACGACATTCCTCCAGACGAT
SDCBP	TTGGGGACCAAGTACTTCAG	CACCTTGTGCGCTTTATCAG
SDF2	TGCGCTATGGGTCAGGTAG	CCCCCGTATCCTCCAGTAAC
SEC22C	TGATTTTTACCACACCCAAGATT	CCAGTCGCAAGGCTAAACTC
SEC61A1	CAAAGCAGCTGAAGGAGCA	ATGCACAGCCCACCAAAG

SEL1L3	GTGATAGTACGCGCCTGGAT	GAGCAAGTTTTCCTCCCATTT
SEMA3A	GGAGACTTGGTATGATTTAGAAGAGG	TGCTGAAATAGCAGTCGGTTC
SEMA3F	ATGCCCTTCTCAGGGAAGAT	TTCATAGATGGCGTGAAGGTT
SEMA6B	GAGTGTGCAAGAACGACGTG	TCCGGGTACAGAGCAGTTG
SERINC3	GGATTCAGATAATTTTATTGGACTGTT	TAGTGGAAGTGCGGATGCTA
SERPINB2	CATGGAGCATCTCGTCCAG	ACTGCATTGGCTCCCACTT
SERPINE1	AAGGCACCTCTGAGAACTTCA	CCCAGGACTAGGCAGGTG
SFRP1	GCTGGAGCACGAGACCAT	TGGCAGTTCTTGTTGAGCA
SIAE	CTCTGTGATAGAGACTCGCCTT	CAGCCGATAAGCCACAGTC
SLC25A6	TGGGAAAGTCAGGCACAGA	AGACACTGAAGCCCTGGTACA
SLC29A1	CTGACTGTGGTCTTCGAGCA	CGAGGTAGCCGTTGGAGA
SLC35F2	ATTGCTTTGGGATTCCTGTG	GAAGTGGATCACTCTGTATCTTGC
SLC38A1	CCTTCACAAATATCAGAGTAAAGATGA	TGAGGATCACAGCAACAATGA
SLC38A2	CCTATGAAATCTGTACAAAAGATTGG	TTGTGTACCCAATCCAAAACAA
SLC7A11	TGATGGTCCTAAATAGCATGAGTG	GCTGTGAGCTTGCAAAAGGT
SLC7A7	TTGGAGGATTCCTTGCTTTC	CAAAGGTGATGGCAATGATG
SMPDL3A	ACCACCATCCAGAGTCTCTTTC	GGTGACTACAGGCAGTTGATCC
SPARC	TTCCCTGTACACTGGCAGTTC	AATGCTCCATGGGGATGA
SPATA20	CTACTTCTGCAGTGAGGCTGAG	AGGTTGTGGGCTGACACG
SPCS3	CGGGCGAACTCACTGTTC	GACGCTCCTGTCTTTGAAGG
SPNS2	CTGCTTTACGGGATTTCTGG	GGCAGATGAAGATGGCAGAG
SPOCK1	GACAATGACCAGTGGCTGAG	TTCCAGTTTCTGAAATAATCATCG
SPTLC2	ATCTGGATGAGGCTCACAGC	CCCATCATAACATCCACATCC
SRGN	GCACCCTGCTACATTTCCTAA	GCTTCTGCATTCATGACCAAC
SRPX2	AGGGCACAAAATACCATAAAACA	GAAGTCAGCCATCAATTAACTTC
SSR1	TGACATGAGTTGGATTCCTCAG	TCCCACTGATCTCTTCTGTGC
SSR3	ACTCGAAAACTTTCTGAAGCTGA	TTTAAACAGGAGTCAAACCTTTCAT
STAB1	AGCCAGGGGAAGTTGCTT	GCCCAGGAACTGTTGTCAG
STC1	GAGGCGGAGCAGAATGACT	GTTGAGGCAACGAACCACTT
STC2	AGGGTCCCCAAGACAGGA	GTGCTGGATCTCCGCTGT
STOM	TCTTTAGATTGGGTCGCATTTT	TGTCAGTGCATGGCAGAATAA
STT3A	CCACAGAACATTGGCTGGT	TGTCCTTGACAAGCCTCGAT
STT3B	CGTTGGTGGATAATAACACCTG	GAAGACATAGCTTTTCCCACCA
STX12	GGAACGCCTCATGAATGACT	TTTTCCTTTTCAGATACCCTTCTC
STX3	TGAGATTGAGGGACGACACA	TCGTGAAGCTCCTTGATGC
STX4	CTCAGCGGACTACGTGGAAC	CTCGCCTTCTTCTGGTTCTC
STX7	CAAAGGAAAATACAGAAGGATCG	CCTTCTGGAAGTTTGTCAGTGAT
SVEP1	CCATCGTGACCTTCTCGTCC	TGAAGAAGAATTTGCGCGGC
SYNJ2	GGCACCAAAGC GATGAAG	TCCAGAAGGGGTCCTGATAA
SYT11	GGTGACCGTCTTTGTCTGGT	TTGTATGGTGGGTTCTTCTGC
TCN2	TCAAGCTTGGTTACCAGCAGT	GGCAGTCACCGTCATCCT
TCTN1	CACTATGCAATCTGGCTGTAAAC	GCCACGTAATCTGGGAAGC
TEK	GAGAAGCCCCTGAACTGTGA	GCAAATGATGGCCTCTCATAA
TFPI	GCCTGGGCAATATGAACAAT	CCACCTGGAAACCATTCG
TGFB1	ACTACTACGCCAAGGAGGTCAC	TGCTTGAACTTGTCATAGATTTC
TGFBI	CGAGTGCTGTCCTGGATATG	CCCAGGGTCTCGTAAAGGTT
TGFBR2	CAAGGGCAACCTACAGGAGT	ACTGTGGAGGTGAGCAATCC
TGOLN2	GCCACTTCTTTGCATATCTGG	CCAGGACAAAAGCAATGATCT
THBS1	CCATGGAGACCAGCCATC	GCCACAGTTCCTGATGGAG
THSD1	ACATGTGAGTCCGGGGTAGA	TGACCACTCCTTGGACGAA
THSD7A	GCTCAGGTACAGCCTGCAT	TGTCTTACTTGTCGGGAGTGG
TIE1	TTCTTCCTGACTTGCGTGTCT	CACGATACGGTCGTCCTTC
TIMM22	CGACTGCAAAAGAAGTGCTG	GCGAAATTTTTGGCATAGGA
TIMP1	CTGTTGTTGCTGTGGCTGAT	AACTTGGCCCTGATGACG

TIMP2	GAAGAGCCTGAACCACAGGT	CGGGGAGGAGATGTAGCAC
TIMP3	GCTGGAGGTCAACAAGTACCA	CACAGCCCCGTGTACATCT
TINAGL1	CAGATGGAAGGACGCTCAA	TCGATGTCGCACTCATTGAC
TLR4	TTCAAGGTCTGGCTGGTTTAG	CAAACTTTTCCAAGTTTCCTTCA
TM2D2	GGTTCATTTTGGTGGCTGTC	CCCTTTCCAAGCCAATTAAA
TM4SF1	TGTATCTCTGTTTTCTATCCTCTTGG	CATATGCCTCCAAGCACTCC
TM4SF18	TCGGAATTGCTTTTTCTGGA	AATATGGCCCTTGGACAAGAC
TM9SF2	CTGATGACGTGTGCTGTGGT	TTTCCACTTCTCACCTCCAAA
TM9SF3	CAAGAGCCATTCCTTTTGGA	GGCCAAGTATTGTACCAACAAGAT
TMCC3	CCTTAGAGGAGTTTAGGCCAGA	TGGGTTTGTTCACGATGGTA
TMCO3	CATTGTTACCATAGGAATGCTGTC	AACCAAACATTGTAGGCAATCC
TMED10	CGTGAGGAGATTCACAAGGAC	GCCAGCAGAATCTGTGATCTT
TMED2	TACATGGAAGTCCGGGAGAG	CCAAAGGACCACTCTGCTGT
TMEM109	CCTCTACGCCCTGCTGAG	CACCTTGGCCTCGAGTTG
TMEM123	CACAATGACCGTAACCCACA	CAGAATGCATAGTTGTTGTGATTG
TMEM173	CATCCATCCATCCCGTGT	TAGCC CCCAAAGGG TCAC
TMEM184B	TCACCAGTGGCTACCTCTACG	GGTGGCGAAGTAGAAGAGGA
TMEM30A	ATCTCCGGATGTGACACCTT	ACACGTTGCCCTCAAATGAC
TMEM44	GGGAAGACATTTCCCTCCAT	CAATGGCCGAGGCATAGA
TMEM5OA	CAGAATATGCTCAGGGAATGC	AGTCCTTGTGGGAATCATGC
TMX3	CCAAGAACAGATAGAAGAGAGCAA	GCTCCTGCACTGTAGGCACT
TNC	CCGGACCAAAACCATCAGT	GGGATTAATGTCGGAAATGGT
TNFRSF10B	CCACAAAGAATCAGGTACAAAGC	GCTGGAGGTCACCGTCTC
TNFRSF10C	CTTCCCCTTCTGCTGCCTA	ACGGACAGGAACTGGCTCT
TNFRSF10D	CGGAGTGACATCAAGTGCAA	CTGTCTCCTCCGCTGCTG
TNFRSF11A	TCATCGTGGTCTACGTCAGC	CAGGGTCTCCTCCTGCAC
TNFRSF1B	GACACCGTGTGTGACTCCTG	GCTCAAGCACTCGGGAAC
TNFSF10	TGCTGATCGTGATCTTCACAG	TGCTTCAGCTCGTTGGTAAA
TNFSF12	GATCGCAGCCCATTATGAAG	CTCACTGTCCCGTCCACAC
TNFSF15	CAAGGGCACACCTGACAGT	GCCAGGCCTAGTTCATGTTC
TOR2A	AGGCCTGATGGAAGTCCTG	ATGCCACCTGGTTGATCTG
TRAM2	CTGGAATGAGCAGAGTGCAA	TGATGAGCCTGGCTGGTAGT
TSKU	TCTCCCACCTGTGAACTGC	CTGTCGAAAAGGCCGAAG
TSPAN14	CATCGCCATCTCGCTGTT	TCACTGCCTCGATGTCTGA
TSPAN15	CGTGATCATCTGGTTCATGG	CGGGGAAATACCAGCTTTG
TSPAN18	CGTCCGTGAGAACAAGTGTC	CTGAAGATGAAGGCCAGGAT
TSPAN3	CACTGGCATTTGCAGCTATTC	CTGCACAACACGATGCAAG
TSPAN5	ACCCAGTTTGGCTCTTCCTT	AGGAAAGTGTTTTCCCGTAGC
TSPAN6	AGGTGAGCCTGGAGAATTACTTT	CCAGTAGCAATGAGCACGAA
TSPANS	TTCTGTGTTCCTGGGAATTATTTT	CAGCTGGTCTTTGATCCAGTC
UBE2J1	GTTAGAGGGCCCCCAGACT	CAGTACTATCCGCCCGTGA
VAMP3	CTCTGCTGACCCTCTCTCGT	GCAGCAGTTGGACCTGTAGA
VAMP5	GCTGGGAGAACATCCGTTAC	AGGATGATGAGCAGGACACC
VAMP8	ATGATCGTGTGCGGAACC	GCTCCACATTCTGGGTCATAA
VANGL1	CCTCCTCTTCATCCATTACCTG	CACCTGCAGCGTGAACAT
VASH1	GGTCCTGGTGGGAGAGG	AAGCACTCGGTGTGGAC
VEGFA	CAGCACAACAAATGTGAATGC	GGTTCCCGAAACCCTGAG
VEGFB	CTGGCCACCAGAGGAAAGT	CTGGAGCTCCACAGTCAAG
VEGFC	GTGATTATTCCACATGTAATTGGTG	TGCCAGCAACACTACCACAG
VOPP1	GAGCCGGCTTCTTCATCC	GCCTGGTGTAGGACACATTG
VWCE	GCTGAAGGGATGTGTTTTC	CACTCAGGAGAGATGCAGGAC
VWF	GAAATGTGTCAGGAGCGATG	ATCCAGGAGCTGTCCCTCA
WLS	GCGATCTGGGTGTCCATAGT	CCACATGGGTGATAGTGGTG
YIPF5	CTATTCGCAGCAAGGCAGAT	TAAATCTGCCCGGTGTATGG

Mouse primers		
Ccl2	CACTCACCTGCTGCTACTCA	GCTTGGTGACAAAAACTACAGC
Ctsd	GACCTGATCCTCAAAGGCCC	CCATAGTACTGGGCATCCAGG
Gapdh	AGCTTGTCATCAACGGGAAG	TTTGATGTTAGTGGGGTCTCG
Loxl2	CGCTTCCAGACAGAGTTCCT	AGGGTACTGGAGCTGGTAGG
Pear1	TCAGCTACCCTCTTAGCAGAAATC	GGGACAAAGTGGCATTGCAG
