# Supplementary material for: Lung endothelial PEAR1 induces tumor cell dormancy
Source: Mol Cancer. 2025 Nov 3;24:278. doi: 10.1186/s12943-025-02488-3 (PMC12581312; doi:10.1186/s12943-025-02488-3)
Supplement: Supplementary file 2 — Supplementary Material 2. [file 12943_2025_2488_MOESM2_ESM.pdf]

Supplementary figures

to

Endothelial PEAR1 induces tumor cell dormancy

Roquid et al.

Supplementary Figures 1-11

Suppl. Figure 1

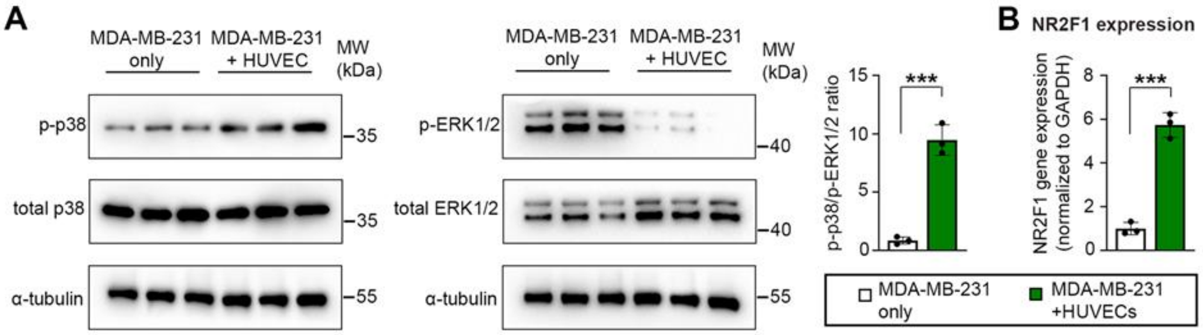

Suppl. Figure 1. MDA-MB-231 tumor cells exhibit increased p-p38/p-ERK1/2 protein ratio and NR2F1 mRNA expression upon co-culture with endothelial cells. (**A**, **B**) Human MDA-MB-231 cells expressing mCherry were cultured alone or together with HUVECs for 96 h. Following the sorting of MDA-MB-231 cells from both set-ups, protein and mRNA were extracted for subsequent analysis. Protein samples were analyzed by immunoblotting using the indicated antibodies. Shown are representative blots and the statistical analysis (A) (n=3 independent experiments). mRNA samples were used for qRT-PCR analysis of *NR2F1* expression. Shown is the statistical analysis (B) (n=3 independent experiments). Shown are mean values ± S.E.M.; ***, P ≤ 0.001 (two-tailed, unpaired t-test with Welch's correction (A); unpaired t-test, two-tailed (B)).

Suppl. Figure 2

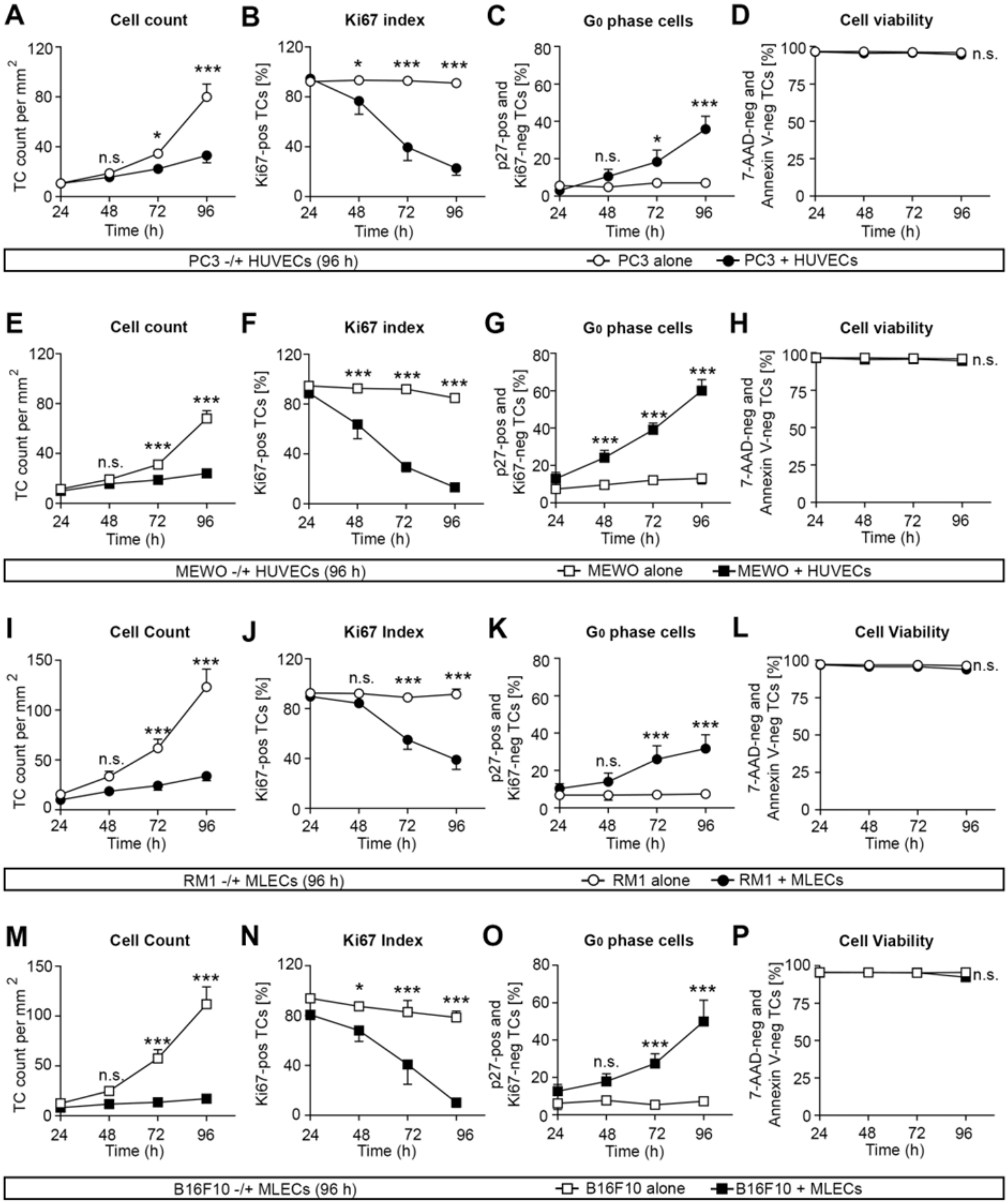

Suppl. Figure 2. Endothelial cells induce dormancy of human and mouse tumor cells *in vitro*. A-C, PC3 tumor cells (A-D) and MEWO tumor cells (E-H) co-expressing mCherry-luciferace and mVenus-$p27K^-$ were cultured alone or together with HUVECs for 96 h. Shown is the quantification of the tumor cell count (A, E), the percentage of Ki67-positive cells (B, F), the percentage of p27-positive and Ki67-negative cells (C, G), and the percentage of alive cells as indicated by 7-AAD and annexin V negativity

(D, H) after 1-4 days of culture (n=3 independent experiments). **I-P**, RM1 tumor cells (I-L) and B16F10 tumor cells (M-P) co-expressing mCherry-luciferace and mVenus-p27K^{-} were cultured alone or together with mouse lung endothelial cells (MLECs) for 4 days. Shown is the quantification of the tumor cell number (I, M), the percentage of Ki67-positive cells (J, N), the percentage of p27-positive and Ki67-negative cells (K, O), and the percentage of alive, 7-AAD-negative and annexin V-negative cells (L, P) after 1-4 days of culture (n=3 independent experiments). Shown are mean values ± S.E.M.; *, $P \leq 0.05$; ***, $P \leq 0.001$; n.s., non-significant (2-way ANOVA with Bonferroni's post-hoc test).

Suppl. Fig. 3

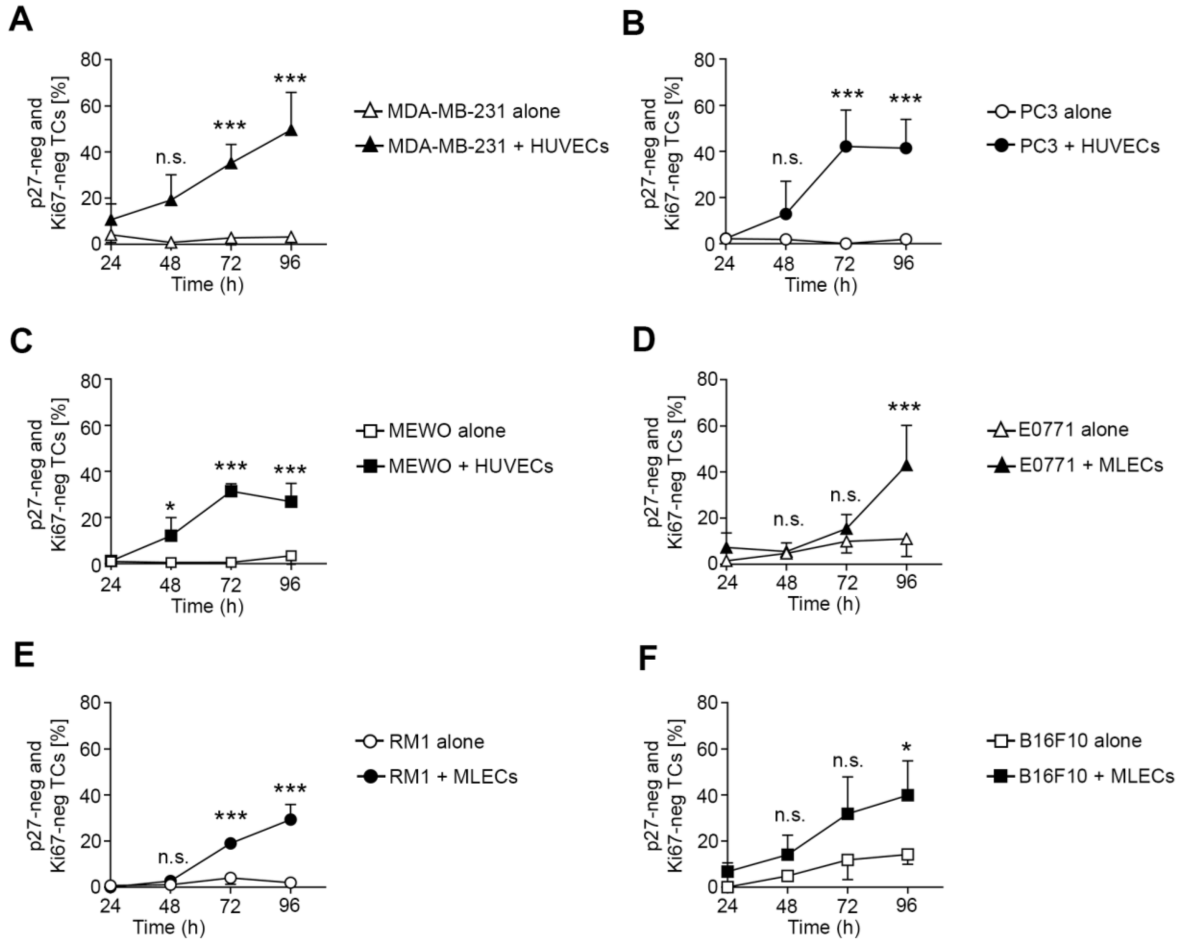

Suppl. Figure 3. Increased proportion of p27-negative and Ki67-negative tumor cells is observed upon co-culture with endothelial cells. A-F, Human and mouse cancer cells co-expressing mCherry-luciferase and mVenus-p27K^{-} were cultured alone or together with HUVECs or MLECs, respectively, for 96 hours in a direct co-culture system. Immunohistochemical staining was performed with Hoechst 33342 and with antibodies directed against mCherry to detect tumor cells (TC), against mVenus to detect the mVenus-p27K^{-} fusion protein (p27) and against Ki67 to detect proliferating cells. Shown are quantifications of the percentage p27-negative and Ki67-negative MDA-MB-231 (A), PC3 (B), MEWO (C), E0771 (D), RM1 (E), and B16F10 (F) during the 96 hr co-culture with endothelial cells (n=3 independent experiments). Shown are mean values ± S.E.M.; *, $P \leq 0.05$; ***, $P \leq 0.001$; n.s., non-significant (2-way ANOVA with Bonferroni's post-hoc test).

Suppl. Figure 4

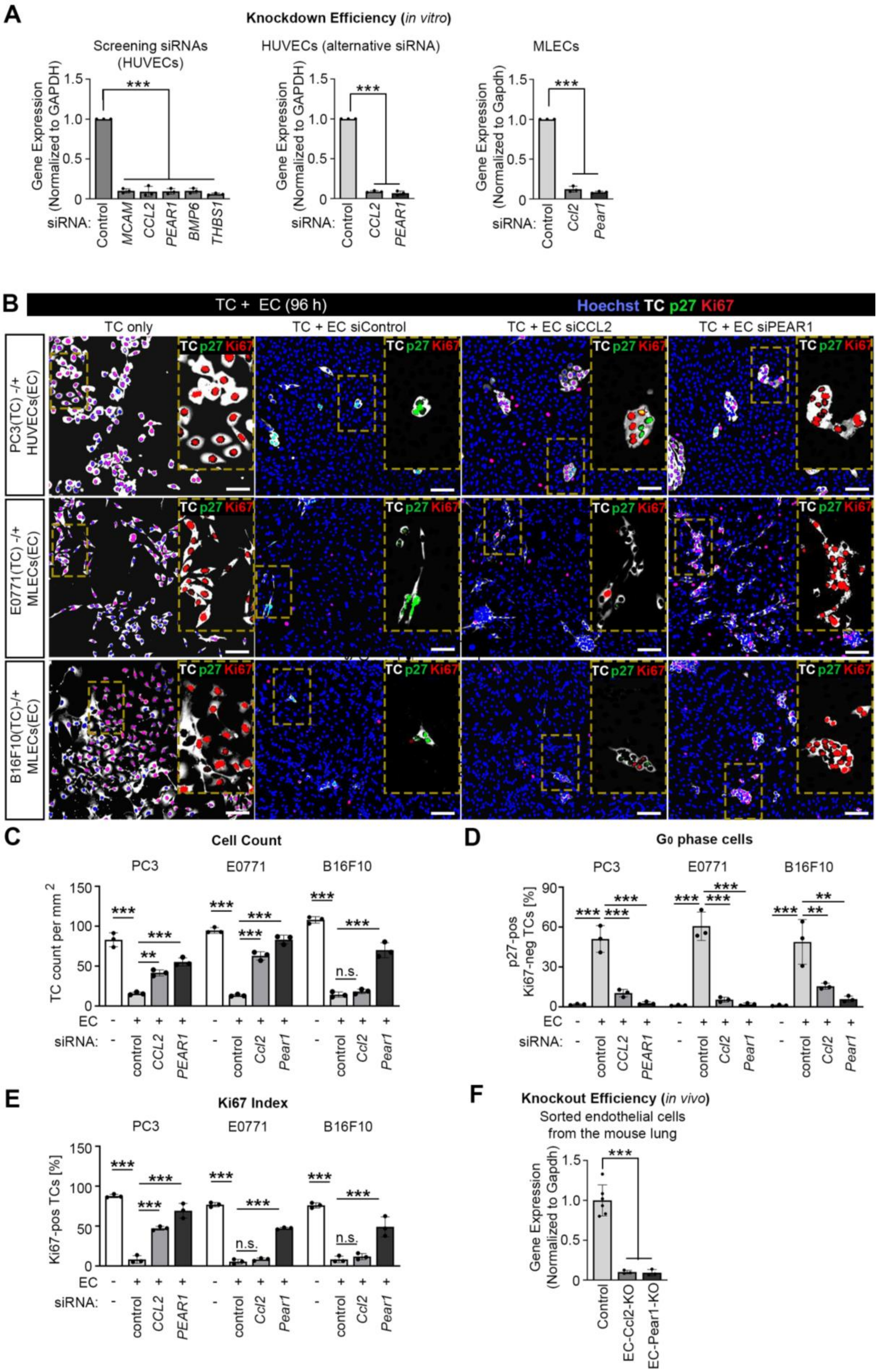

Suppl. Figure 4. Identification of PEAR1 and CCL2 as potential mediators of endothelium-induced tumor cell dormancy. A, Efficency of knock-down of PEAR1, CCL2, MCAM, BMP6 and THBS1 in HUVECs and MLECs (n=3 inependent experiments). **B-E**, PC3 human tumor and E0771 or B16F10 mouse tumor cells co-expressing mCherry-luciferase and mVenus-p27K$^-$ were cultured alone or together with HUVECs or MLECs, respectively, which were transfected with control siRNA or siRNAs directed against *CCL2* or *PEAR1,* and were analyzed 4 days later. Shown in (B) are representative immunohistochemical images stained with Hoechst 33342 and antibodies directed against mCherry (TC), mVenus-p27K$^-$ (p27) and Ki67 with magnified areas indicated by a box with dashed lines. Bar diagrams show the quantification of cell numbers (C), of p27-positive and Ki67-negative cells (E) and of the percentage of Ki67-positive tumor cells (E) (n=3 independent experiments). **F**, Efficiency of knock-out in MLECs isolated from control, EC-Ccl2-KO and EC-Pear1-KO mice (n=6 mice in control; n=3 mice per group for EC-Ccl2-KO and EC-Pear1-KO). Scale bars: 100 μm. Shown are mean values ± S.E.M.; **, P ≤ 0.01; ***, P ≤ 0.001; n.s., non-significant (one-way ANOVA with Tukey's multiple comparison test).

Suppl. Figure 5

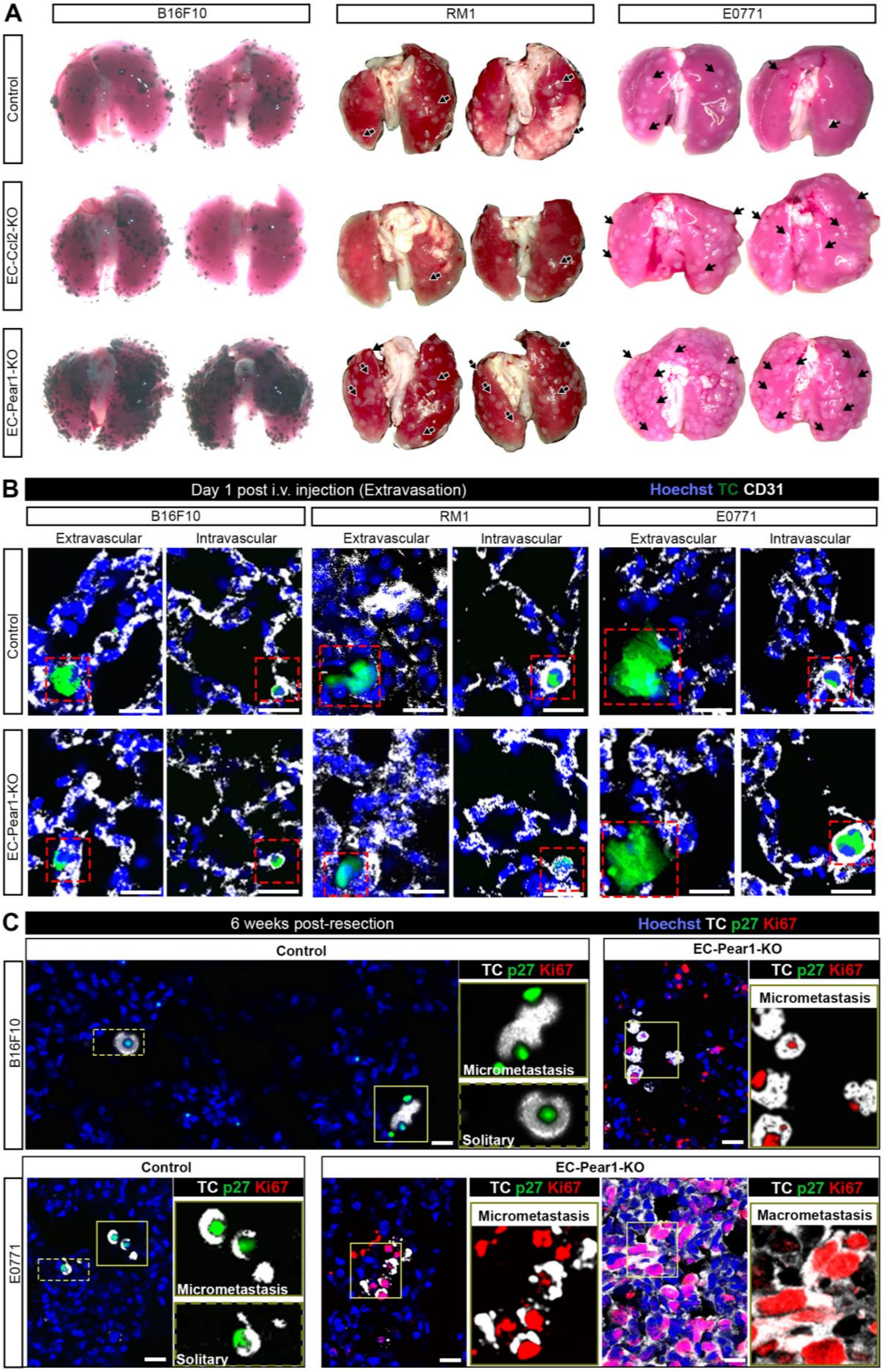

Suppl. Figure 5. Representative images for Figs. 3A and F and Figs. 4I and J. A, Shown are representative images of lung sections from control, EC-Ccl2-KO and EC-Pear1-KO animals 12 days after i.v.-injection of the indicated tumor cells. Arrows indicate metastases. **B**, Shown are representative images of lung sections from control and EC-Pear1-KO animals 1 day after i.v.-injection of the indicated CFSE-loaded tumor cells and after staining with Hoechst 33342, an antibody against CD31 and after visualization of CFSE (TC). Typical examples of extravasated and non-extravasated intravascular tumor cells are shown (scale bar: 20 µm). **C**, Shown are representative images of lung sections 6 weeks after resection of B16F10 and E0771 primary tumors from control mice and EC-Pear1-KO animals stained with Hoechst 33342 and antibodies against mVenus-p27K-, against mCherry to identify tumor cells (TC) and against Ki67. Typical solitary tumor cells, micrometastases (2-8 cells) and a macrometastases (>8 cells are indicated) are shown (scale bar: 20 µm).

Suppl. Figure 6

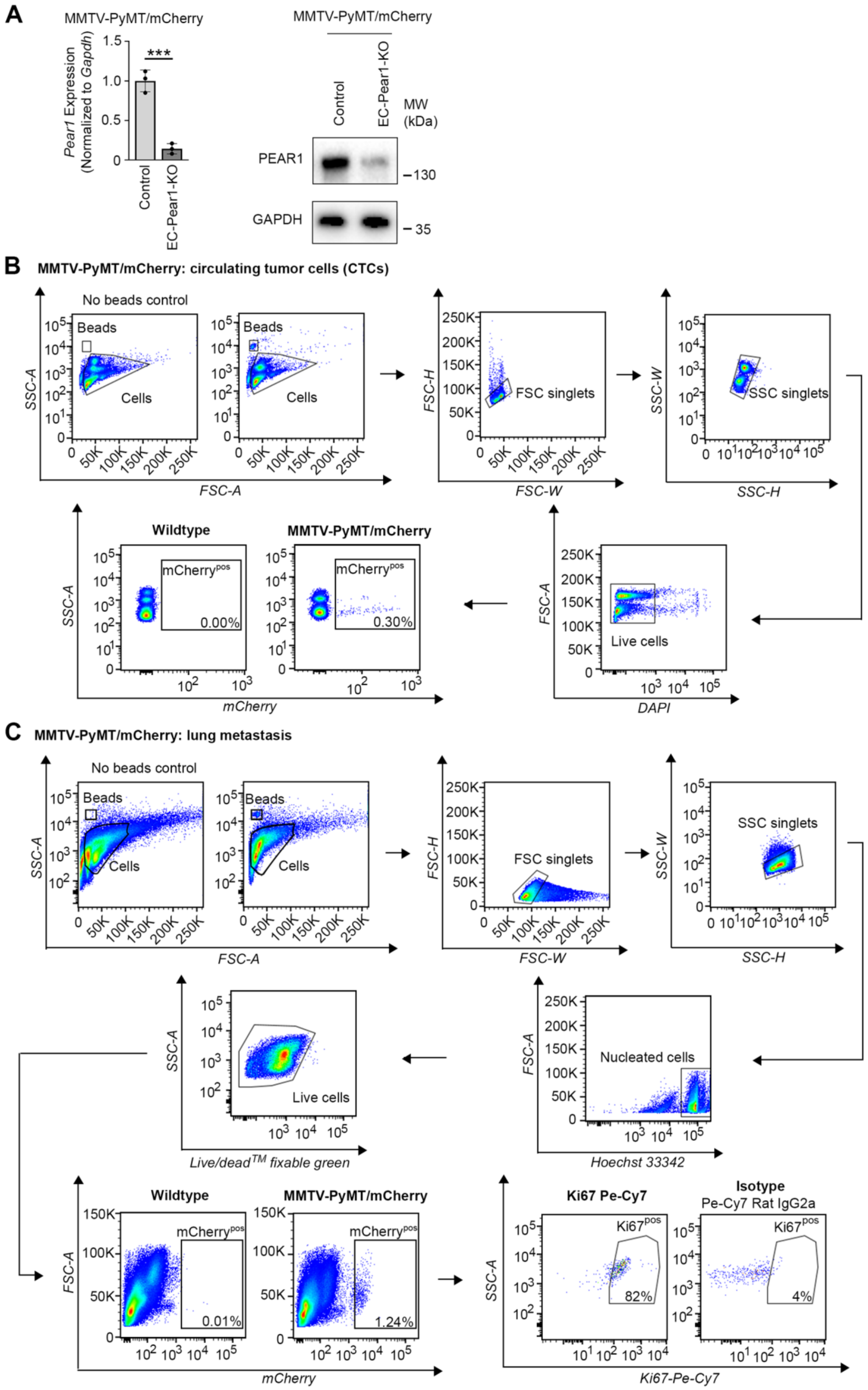

Suppl. Figure 6. Knock-out efficiency and gating strategy used for sorting experiments using MMTV-PyMT/mCherry mice. A, Lungs from control and EC-Pear1-KO mice crossed with MMTV-PyMT/mCherry were digested and endothelial cells were sorted for subsequent extraction of protein and RNA. Efficiency of Pear1 knock-out in MLECs isolated from control and EC-Pear1-KO mice (n=3 mice per group) was determined through qPCR (left panel) and immunoblotting with indicated antibodies (right panel). **B**, Blood samples isolated from MMTV-PyMT/mCherry mice were analyzed for the presence of circulating tumor cells (CTCs) using flow cytometry. Beads and cells were first gated on a forward scatter and side scatter plot. Singlets on both forward and side scatter were then selected, and live cells were gated as DAPI-negative cells. Within the live cell population, CTCs were then selected through the mCherry channel. A wildtype blood sample was used as a biological negative control to gate for mCherry-positive CTCs in the blood. **C**, Lung cell suspensions from MMTV-PyMT/mCherry mice were analyzed for the presence of Ki67-pos and mCherry-pos tumor cells using flow cytometry. Beads and cells were first gated on a forward scatter and side scatter plot. Singlets on both forward and side scatter were then selected, and nucelated cells were gated as Hoechst 33342-positive cells. Within the live cell population (Live/Dead^{TM} fixable green-neg cells), tumor cells were then selected through the mCherry channel. A wildtype lung cell sample was used as a biological negative control. Additionally, mCherry-positive tumor cells were analyzed for Ki67 positivity using the Pe-Cy7 channel. MMTV-PyMT/mCherry lung cell samples stained with the isotype control, Pe-Cy7 Rat IgG2a, were used as a fluorescence minus one (FMO) control to gate for Ki67-positive tumor cells for lung cell suspensions that were stained with Pe-Cy7-conjugated anti-mouse Ki67 antibody. Shown are mean values ± S.E.M.; ***, P ≤ 0.001 (unpaired t-test, two-tailed (A)).

Suppl. Figure 7

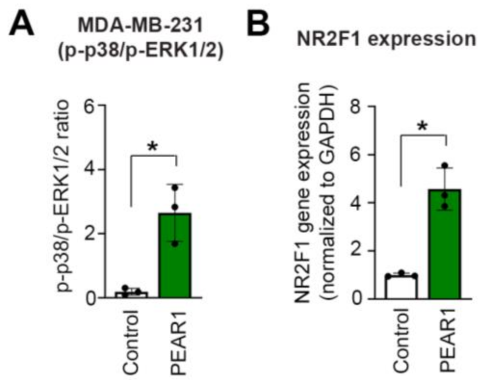

Suppl. Figure 7. MDA-MB-231 tumor cells show increased p-p38/p-ERK1/2 protein ratio and *NR2F1* expression after treatment with the extracellular part of PEAR1. A, **B**, MDA-MB-231 cells were treated daily for 4 days with the extracellular part of PEAR1 immobilized on Ni-NTA beads, followed by protein and mRNA extraction. Protein samples were analyzed by immunoblotting using antibodies against p-p38 and p-ERK1/2. Shown is the quantification and statistical analysis of the ratio of p-p38/p-ERK1/2 (A) (n=3 independent experiments). The mRNA samples were used for qRT-PCR analysis of *NR2F1* expression. Shown is the statistical analysis (B) (n=3 independent experiments). Shown are mean values ± S.E.M.; *, $P \leq 0.05$; **, $P \leq 0.01$; ***, $P \leq 0.001$; n.s., non-significant (two-tailed, unpaired t-test with Welch's correction (A, B)).

Suppl. Figure 8

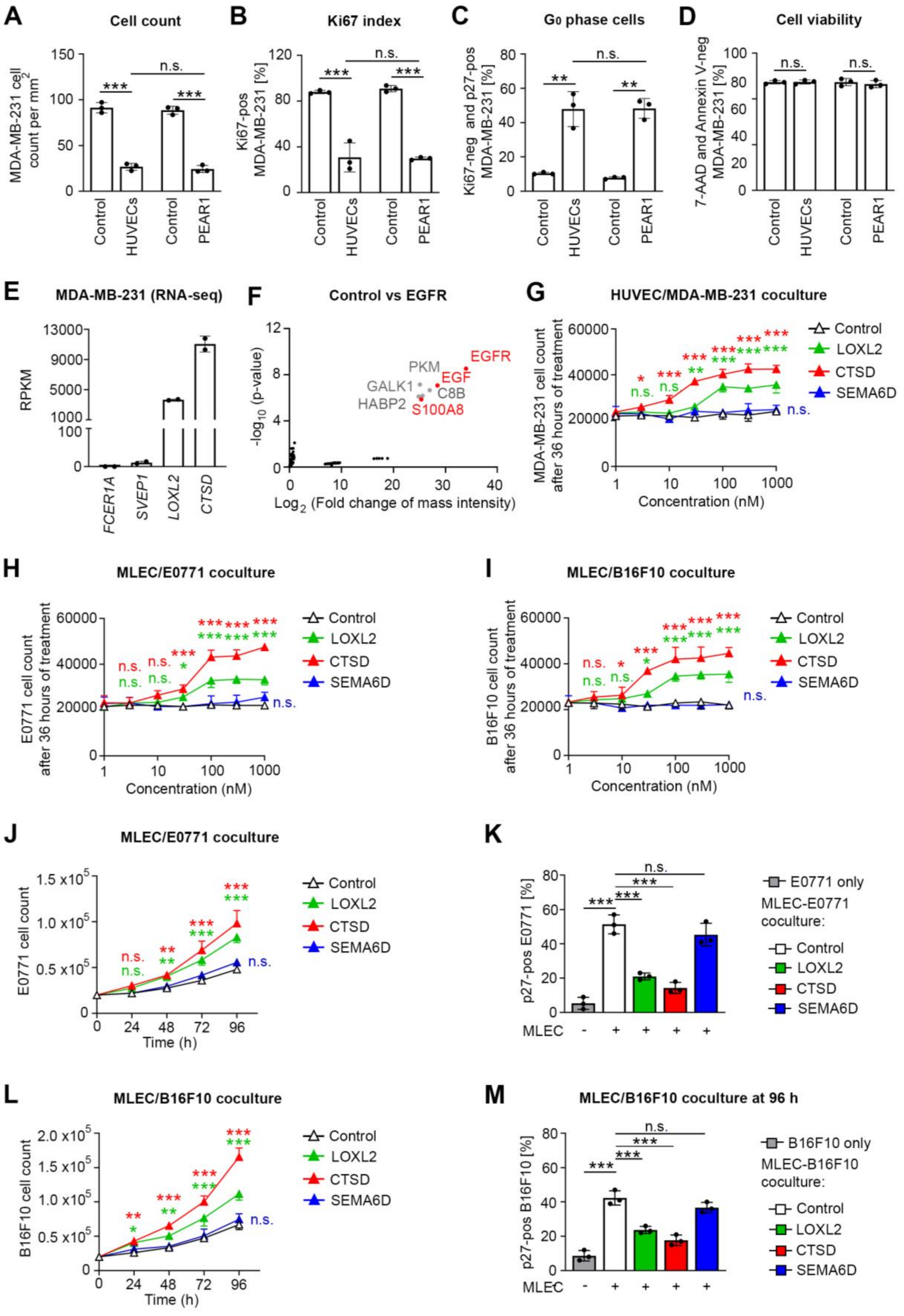

Suppl. Figure 8. PEAR1-induced tumor cell dormancy and role of CTSD. A-D, MDA-MB-231 cells that co-expressed mCherry-luciferase and mVenus-p27K$^-$ were cultured alone (control) or with HUVECs or were alternatively cultured alone and were treated daily with the extracellular part of PEAR1 immobilized on Ni-NTA beads or with control Ni-NTA beads (control). Thereafter, cells were stained with DAPI and with anti-mCherry, anti-mVenus, and anti-Ki67 antibodies. Shown is the quantification of cell number (A), percentage of Ki67-positive (B), p27-positive and Ki67-negative (C) as well as of live cells as indicated by 7-AAD and annexin V negativity (D) after 4 days of culture (n=3 independent experiments). **E**, RNA-seq-based expression of *FCER1A*, *SVEP1*, *LOXL2* and CTSD in MDA-MB-231 breast cancer cells. **F**, Analysis of potential interactions between the recombinant human His-tagged extracellular domain of the EGF receptor (EGFR) and proteins in the supernatants of MDA-MB-231 tumor cells. The plot shows proteins that were specifically pulled down with EGFR immobilized on Ni-NTA beads in comparison to control samples. Proteins only found with EGFR are indicated by red dots, and proteins found with both PEAR1 and EGFR are indicated by grey dots (n=3 independent experiments). **G**, MDA-MB-231 cells co-expressing mCherry-luciferase and mVenus-p27K$^-$ were co-cultured with HUVECs and treated with increasing concentrations of CTSD, LOXL2 and SEMA6D, and cell numbers were quantified by flow cytometry after 36 hours (n=3 independent experiments). **H**, **I**, E0771 or B16F10 mouse tumor cells expressing mCherry-luciferase and mVenusp27K$^-$ were co-cultured with MLECs for 4 days. Cells were treated daily with increasing concentrations of either CTSD, LOXL2 or SEMA6D, and the number of E0771 (H) and B16F10 (I) cells was determined by flow cytometry (n=3 independent experiments). **J-M**, E0771 or B16F10 tumor cells expressing mCherry-luciferase and mVenusp27K$^-$ were co-cultured with MLECs for 4 days in the absence (control) or presence of LOXL2 (100 nM), CTSD (100 nM) or SEMA6D (1000 nM), and cells were counted (J, L). After 4 days, the percentages of p27-positive E0771 (K) and B16F10 (M) cells were evaluated by flow cytometry (n=3 independent experiments). Shown are mean values ± S.E.M.; *, P ≤ 0.05; **, P ≤ 0.01; ***, P ≤ 0.001; n.s., non-significant (2-way ANOVA with Bonferroni's post-hoc test (A-D, G-J, L); multiple two-tailed t-tests, Bayesian moderated, Benjamin, Krieger, and Yekutieli corrected, falso discovery rate = 1% (F); one-way ANOVA with Tukey's multiple comparison test (K, M)).

Suppl. Figure 9

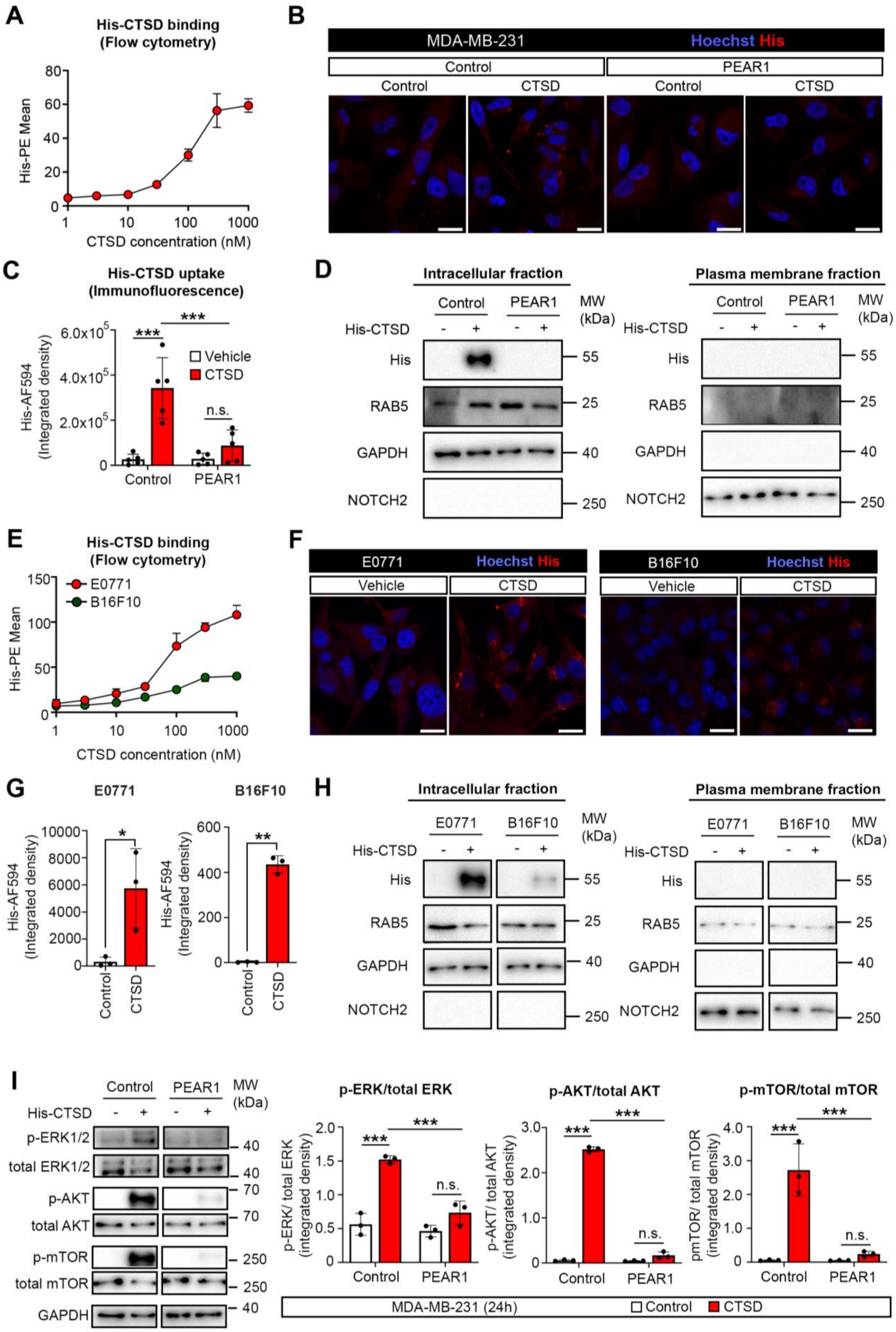

Suppl. Figure 9. Uptake and cellular effects of CTSD. A, MDA-MB-231 cell suspensions were incubated with increasing concentrations of His-tagged recombinant human CTSD for 1 h at 4 °C followed by staining with a PE-conjugated anti-His antibody. Shown is the intensity of PE staining as determined by flow cytometry. **B**, **C**, MDA-MB-231 cells were incubated without (control) or with 100 nM of His-tagged CTSD for 1 hour in the absence or presence of 100 nM PEAR1. Thereafter, cells were permeabilized and stained with Hoechst 33342 and an antibody against the His-tag. Shown is a respresentative of 5 independently performed experiments (B) and the statistical evaluation of the anti-His signal indicating uptake of CTSD (C). **D**, MDA-MB-231 cells were incubated without and with His-tagged CTSD (100 nM) in the absence or presence of the extracellular part of PEAR1 (100 nM) immobilized on streptavidin beads for 1 h. Thereafter, cells were fractionated to obtain the intracellular fraction and the plasma membrane fraction, and cell fractions were separated and analyzed by immunoblotting using antibodies directed against the His-tag (His) and the indicated proteins, which are markers for the intracellular and plasma membrane fractions of cells. Shown is a representative of 3 independently performed experiments. **E**, E0771 or B16F10 cell suspensions were incubated with the indicated concentrations of His-tagged recombinant mouse CTSD for 1 h followed by staining with PE-conjucated anti-His antibody. Shown is the intensity of PE indicating CTSD binding as determined by flow cytometry (n=3 independently performed experiments). **F**, **G**, E0771 or B16F10 cells were incubated in the absence (control) or in the presence of 100 nM of His-tagged CTSD for 1 h. Thereafter, cells were permeabilized and stained with Hoechst 33342 and an anti-His antibody. Shown is a representative of 3 independently performed experiments (F) as well as the statistical analysis of the anti-His signal indicating CTSD update (G). **H**, E0771 or B16F10 cells were incubated in the absence or presence of His-tagged CTSD for 1 hour, and cells were then fractionated into the intracellular and the plasma membrane fraction. Thereafter, proteins were separated and analyzed by immunoblotting using antibodies against the His-tag or against the indicated proteins. Shown is a representative of 3 independently performed experiments. **I**, MDA-MB-231 cells were treated without or with 100 nM CTSD in the absence (control) or presence of 100 nM of the extracellular part of PEAR1. Cells were then analyzed by immunoblotting using the indicated antibodies. Shown is a representative of 3 independently performed immunoblots as well as the statistical evaluation. Scale bars: 25 μm. Shown are mean values ± S.E.M.; *, $P \leq 0.05$; **, P ≤

0.01; ***, P ≤ 0.001; n.s., non-significant (2-way ANOVA with Bonferroni's post-hoc test (C, I); unpaired t-test, two-tailed with Welch's correction (G: E0771); unpaired t-test, two-tailed (G: B16F10)).

Suppl. Figure 10

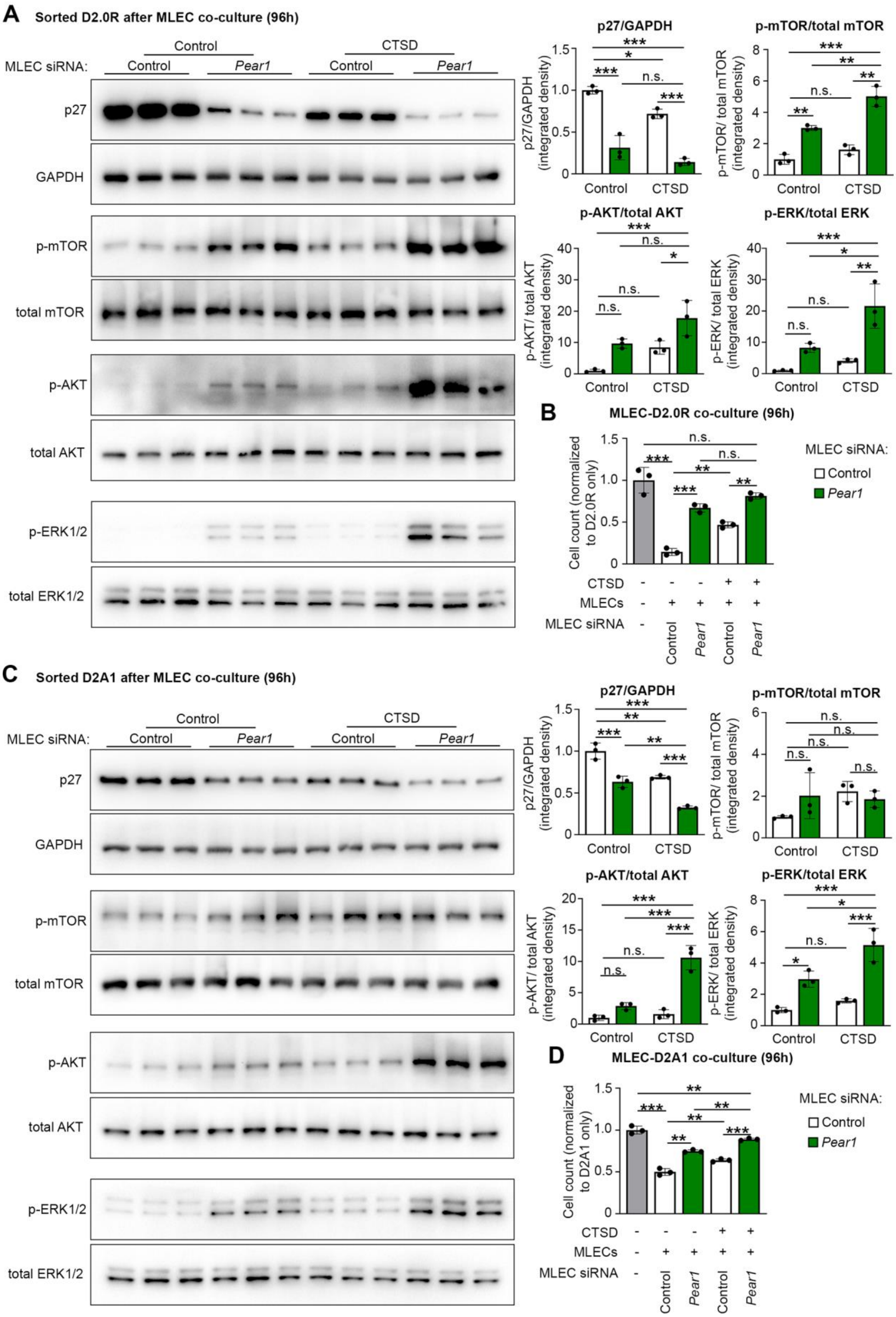

Suppl. Figure 10. Effect of PEAR1 and CTSD on cellular dormancy and downstream signaling markers in D2.0R and D2A1 cells. A-D, D2.0R and D2A1 cells, loaded with CFSE Cell tracker dye, were co-cultured with MLECs that were transfected with either control siRNA or siRNA against *Pear1* and without or with CTSD (100 nM) treatment. After 96 hours, MLECs were stained for CD31, tumor cells ($CFSE^{pos}$) were isolated by FACS, and cells were then processed for Western blotting. Cell count was determined after addition of Accucount counting beads by flow cytometry. Shown are immunoblots of lysates of sorted D2.0R (A) and D2A1 (C) cells stained with the indicated antibodies (left panel) and the corresponding statistical analysis (right panel) as well as the corresponding tumor cell count for each set-up (B, D). Shown are mean values ± S.E.M.; *, P ≤ 0.05; **, P ≤ 0.01; ***, P ≤ 0.001; n.s., non-significant (2-way ANOVA with Bonferroni's post-hoc test (A,C); one-way ANOVA with Tukey's multiple comparison test (B, D).

Suppl. Figure 11

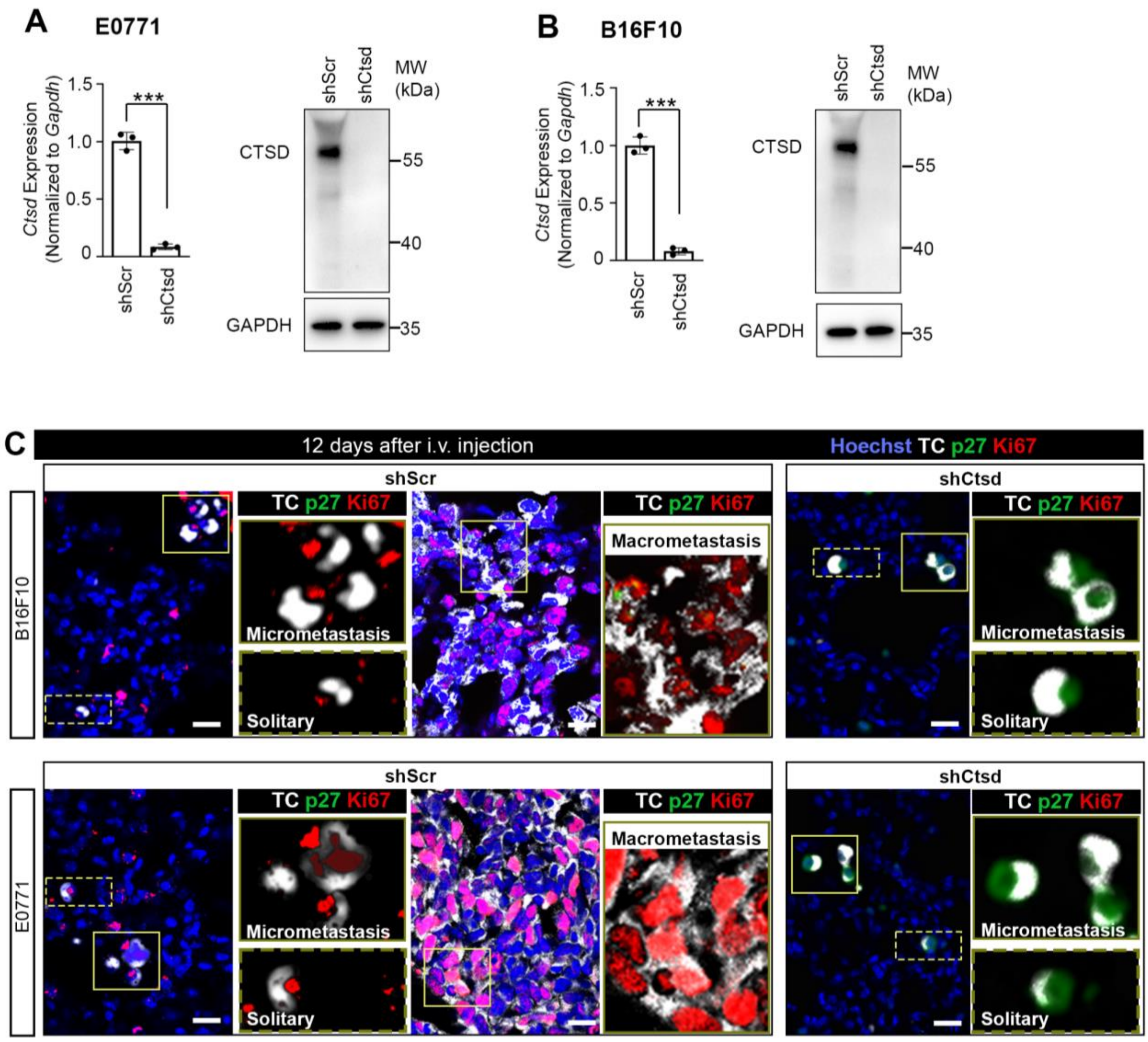

Suppl. Figure 11. Knock-down efficiency of CTSD in mouse tumor cells and representative images of Fig. 7I and J. A, **B**, E0771 (A) and B16F10 cells (B) were infected with lentivirus transducing either scrambled control shRNA (shScr) or an shRNA targeting *Ctsd* (shCtsd). Efficiency of knockdown of Ctsd was evaluated by qPCR (left panels) and immunoblotting with the indicated antibodies (right panels) (n=3 independent experiments). **C**, Shown are representative images of lung sections 12 days after injection of E0771 or B16F10 tumor cells without or with stable knock-down of CTSD (shScr or shCtsd, respectively) co-expressing mCherry-luciferase and mVenusp27K^{-} into mice. Sections were stained with DAPI and with antibodies against mVenus-p27K-, against mCherry to identify tumor cells (TC) and against Ki67. Typical solitary tumor cells, micrometastases (2-8 cells) and a macrometastases (>8 cells) are shown (scale bar: 20 µm). Shown are mean values ± S.E.M.; ***, P ≤ 0.001 (unpaired t-test, two-tailed).
